# Herbal Medicine for the Treatment of Anorexia in Children: A Systematic Review and Meta-Analysis

**DOI:** 10.3389/fphar.2022.839668

**Published:** 2022-04-01

**Authors:** Boram Lee, Chan-Young Kwon, Sun Haeng Lee, Gyu Tae Chang

**Affiliations:** ^1^ KM Science Research Division, Korea Institute of Oriental Medicine, Daejeon, South Korea; ^2^ Department of Oriental Neuropsychiatry, Dong-eui University College of Korean Medicine, Busan, South Korea; ^3^ Department of Korean Pediatrics, College of Korean Medicine, Kyung Hee University, Seoul, South Korea

**Keywords:** herbal medicine, anorexia, children, systematic review, meta-analysis

## Abstract

**Background:** Anorexia is a common obstacle to adequate nutrition in childhood, a critical period for physical growth. East Asian traditional medicine treatment modalities including herbal medicine (HM) a re considered an attractive therapeutic option, especially in East Asian countries. The purpose of this systematic review was to comprehensively examine the efficacy and safety of HM for anorexia in children.

**Methods:** A total of 12 electronic databases from their inception date to June 2021 were searched for randomized controlled trials (RCTs) assessing the efficacy of HM for the treatment of anorexia in children. The primary outcome was an improvement in anorexia clinical symptoms after treatment. In this meta-analysis, continuous and binary outcomes were assessed, and the data are presented as the mean difference or standardized mean difference and risk ratio (RR) with their 95% confidence intervals (CIs). The risk of bias and quality of evidence were assessed using the Cochrane Collaboration’s risk of bias tool and Grading of Recommendations, Assessment, Development, and Evaluations tool.

**Results:** A total of 205 RCTs were included. A comparison of HM with placebo revealed that the total effective rate based on anorexia symptom improvement was significantly higher in the HM group (RR 1.58, 95% CI 1.34, 1.85). In comparison with controls, HM as monotherapy or adjunctive therapy to dietary supplements or conventional medications led to significant improvements in anorexia symptoms, body measurements, levels of blood biomarkers related to gastrointestinal function, and nutrition indices, with a lower recurrence rate of anorexia. No serious adverse events related to HM were reported. The risk of bias of the included studies was generally unclear, and the quality of evidence was generally low to moderate.

**Conclusion:** Our study showed that HM could improve clinical symptoms, some anthropometric outcomes, and some biological markers related to appetite and growth in children with anorexia. However, considering the high risk of bias of the included studies and the heterogeneity of the HMs used, future research should focus on the use of standardized HMs and the implementation of methodologically robust clinical trials.

**Systematic Review Registration**: https://www.crd.york.ac.uk/prosperodisplay_record.php?ID=CRD42021274376, identifier CRD42021274376

## 1 Introduction

Childhood is a critical period for physical growth, and proper nutrition during this period is crucial to ensure normal growth (Martins et al., 2011; Hurley et al., 2016). Anorexia, a disorder with a decreased desire to eat, is a common obstacle to adequate nutrition in children. In the Chatoor Diagnostic Criteria for Feeding Disorder, infantile anorexia can be defined when a child refuses to eat adequate amounts of food for at least 1 month (not caused by an underlying medical illness or traumatic event), does not express hunger and lacks interest in food but shows strong interest in exploration and interaction with caregivers, and shows reduced growth (Levine et al., 2011). According to some studies, poor appetite rather than insufficient food may be a contributing factor to low energy intake by infants and children (Brown et al., 1995; Mukatay et al., 2010; Carter et al., 2019). Therefore, an inappropriately decreased appetite in childhood is considered to have important public health implications.

Anorexia is common in children; according to an observational study of 504 children under 4 years of age in Korea, the prevalence of anorexia was 27% (Liu et al., 2016). Moreover, among these participants, anorexia was closely associated with a low rate and short duration of breastfeeding, starting on solid food at an inappropriate time, less interest in food during mealtime, and an unbalanced diet (Liu et al., 2016). Likewise, according to a questionnaire survey and a case-control study conducted in China, the main risk factors for this condition in children were the late addition of supplementary food, a high frequency of taking snacks and/or drinks, and eating while playing (Liu et al., 2016). Nutritional intervention (Naila et al., 2021), cyproheptadine (Najib et al., 2014; Sant’Anna et al., 2014; Kim et al., 2021), and folic acid (Hatamizadeh et al., 2007; Riahi et al., 2018) can be used to increase appetite and subsequent food intake in this population. In addition, East Asian traditional medicine (EATM) treatment modalities are considered an attractive therapeutic option, especially in East Asian countries.

In Korea, Korean medicine (KM) hospitals are the most common medical institutions where parents of children with anorexia receive counseling for anorexia (68%) in addition to pediatrics (20%) and internal medicine (3%) (Yoon et al., 2003). Around 10% of outpatients in the department of pediatrics in KM hospitals are presented with eating problems, including anorexia (Choi et al., 2010). In China, EATM is widely used for the treatment of anorexia in children, and a recent systematic review reported that pediatric *tuina*, which is a modality of traditional Chinese medicine, is a safe and effective treatment for improving the symptoms of anorexia in children under 14 years of age (Liang et al., 2020).

Herbal medicine (HM) is one of the representative EATM therapies and widely used for the treatment of various diseases. Recently, modern biomedical research methodologies have been used to elucidate the therapeutic effects and underlying mechanisms of HM. The evidence suggests that some HMs can be used to improve anorexia. For example, standardized HMs such as Rikkunshito may improve anorexia by acting as a ghrelin potentiator (Fujitsuka and Uezono, 2014). Therefore, HM can be an attractive medical option in terms of appetite control. Although it is actively used to improve anorexia in children mainly in East Asian countries, the effect and safety of this intervention in children with anorexia have not been systematically and critically reviewed. Therefore, the purpose of this systematic review was to comprehensively examine the efficacy and safety of HM for anorexia in children.

## 2 Methods

### 2.1 Protocol Registration

We registered the protocol of this systematic review with PROSPERO (Registration number: CRD42021274376) and reported the study according to the Preferred Reporting Items for Systematic Reviews and Meta-Analyses 2020 statement (Page et al., 2021).

### 2.2 Inclusion and Exclusion Criteria

#### 2.2.1 Study Design

Only parallel group randomized controlled trials (RCTs) assessing the efficacy of HM for the treatment of anorexia in children were included. We excluded cross-over studies to reduce the risk of potential bias. There were no limitations regarding the publication language of the study. We included not only studies published in journals but also grey literature including degree thesis.

#### 2.2.2 Study Participants

Studies involving children with anorexia were included. Studies involving children with anorexia with known organic causes were excluded. In addition, we excluded studies that did not clearly describe the diagnostic criteria for anorexia. There were no limitations regarding the sex, race, and nationality of the participants.

#### 2.2.3 Treatment and Control Interventions

For treatment interventions, studies involving oral HM based on the EATM theory for the treatment of anorexia in children were included. However, studies in which the individual herb composition was not specified were excluded. For control interventions, studies with placebo HM, no treatment, and active controls such as dietary supplements or conventional medications were included. However, studies using other EATM therapies such as acupuncture as the control were excluded. Studies involving HM combined with other therapies as treatment interventions were included if the other therapies were used equally in both the treatment and the control groups.

#### 2.2.4 Outcome Measures

The primary outcome was an improvement in the clinical symptoms of anorexia after treatment, measured using such as the total effective rate (TER). For assessment with the TER, participants were classified according to the degree of improvement in anorexia symptoms as “cured” (*N1*), “markedly improved” (*N2*), “improved” (*N3*), or “non-responder” after treatment. The TER was calculated using the following formula: *TER = (N1 + N2 + N3)/total sample size*. The secondary outcomes included 1) body measurements such as body weight and body mass index (BMI), 2) gastric emptying time, 3) levels of blood biomarkers related to gastrointestinal function, such as ghrelin and gastrin, 4) nutrition indices, such as levels of serum trace elements, 5) recurrence rate, and 6) incidence of adverse events (AEs) during the treatment period.

### 2.3 Information Sources and Search Strategy

We comprehensively searched the following 12 English, Korean, Chinese, and Japanese electronic databases from their inception date to 3 June 2021: Medline (*via* PubMed), EMBASE (*via* Elsevier), Cochrane Central Register of Controlled Trials, Allied and Complementary Medicine Database (*via* EBSCO), Oriental Medicine Advanced Searching Integrated System, Korean Studies Information Service System, Korean Medical Database, ScienceON, China National Knowledge Infrastructure, Wanfang Data, Chongqing VIP, and CiNii. We additionally reviewed the reference lists of the relevant studies, Google Scholar, and trial registries such as ClinicalTrials.gov to include any potentially relevant studies. We set the search strategy as comprehensively as possible following consultation with specialists in pediatrics and systematic review experts. The detailed search strategies for each database and search results are presented in Supplementary File S1.

### 2.4 Study Selection and Data Extraction

All studies searched from databases and identified from other sources were imported into EndNote 20 (Clarivate Analytics, Philadelphia, PA, United States). After removing duplicates, the titles and abstracts of the articles were reviewed for first inclusion. For the first included studies, the full-texts were retrieved and reviewed for final inclusion.

For the final included studies, we extracted the following information using a standardized, pilot-tested Excel form: basic information (first author’s name and country, publication year, study setting, or funding source), sample size, details of participants, treatment and control intervention, details of the HM used, outcome measures, results, and information for the assessment of the risk of bias.

Study selection and data extraction were independently conducted by two researchers (BL and CYK), and any disagreement was resolved by discussions with other researchers (SHL and GTC). If the data were ambiguous or insufficient, we contacted the authors of the included studies via e-mail if possible.

### 2.5 Risk of Bias Assessment

We evaluated the methodological quality of the included studies using the Cochrane Collaboration’s risk of bias tool including items of random sequence generation, allocation concealment, blinding of participants and personnel, blinding of outcome assessors, completeness of outcome data, selective reporting, and other biases (Higgins et al., 2011). Specifically, for the assessment of other biases, the statistical homogeneity of baseline clinical characteristics such as mean age or sex between the treatment and control groups was determined. Each item was assessed as “low risk”, “unclear risk”, or “high risk”. Two researchers (BL and CYK) independently conducted the risk of bias assessment, and a consensus was reached through discussions with other researchers (SHL and GTC) if there were disagreements.

### 2.6 Data Analysis and Synthesis

Descriptive analysis of the details of the participants, treatment and control interventions, and outcomes of the included studies was conducted. In particular, the meta-analysis was performed using Review Manager software (version 5.4; Cochrane, London, United Kingdom) if two or more studies used the same type of treatment and control intervention with the same outcome measures. Continuous and binary outcomes were assessed, and the data are presented as the mean difference (MD) or standardized mean difference (SMD) and risk ratio (RR) with their 95% confidence intervals (CIs). Heterogeneity between the studies was assessed using both the χ^2^ test and the *I* ^*2*^ statistic; specifically, 50% ≤ *I* ^*2*^ < 75% and *I* ^*2*^ ≥ 75% were considered to indicate substantial and considerable heterogeneity, respectively. A random-effects model was used if the included studies have significant heterogeneity (*I* ^*2*^ ≥ 50%), whereas a fixed-effects model was used if the heterogeneity is not significant or if the number of studies included in the meta-analysis is small because estimates of inter-study variance have poor accuracy (Borenstein et al., 2010). Subgroup analysis was performed depending on whether the participants’ pattern identification was specified. For sensitivity analysis to confirm the robustness of the meta-analysis, we excluded 1) studies with a high risk of bias and 2) outliers that are numerically distant from the rest of the data. If there were sufficient studies (≥10 studies) included in each meta-analysis, we assessed the evidence of publication bias by Egger’s test using Stata/MP software version 16.1 (StataCorp LLC, College Station, TX, United States).

### 2.7 Quality of Evidence Assessment

We graded the quality of evidence of the main findings using the Grading of Recommendations, Assessment, Development, and Evaluations (GRADE) tool (Balshem et al., 2011). Using GRADEpro (https://gradepro.org/), the risk of bias, inconsistency, indirectness, imprecision, and publication bias were evaluated for the main findings, and the final quality of evidence for each finding was presented as “very low”, “low”, “moderate”, or “high”. A researcher (BL) assessed the quality of evidence, and another researcher (SHL) reviewed the results. Any discrepancies were resolved through discussions with other researchers (CYK and GTC).

## 3 Results

### 3.1 Study Selection

**FIGURE 1:**
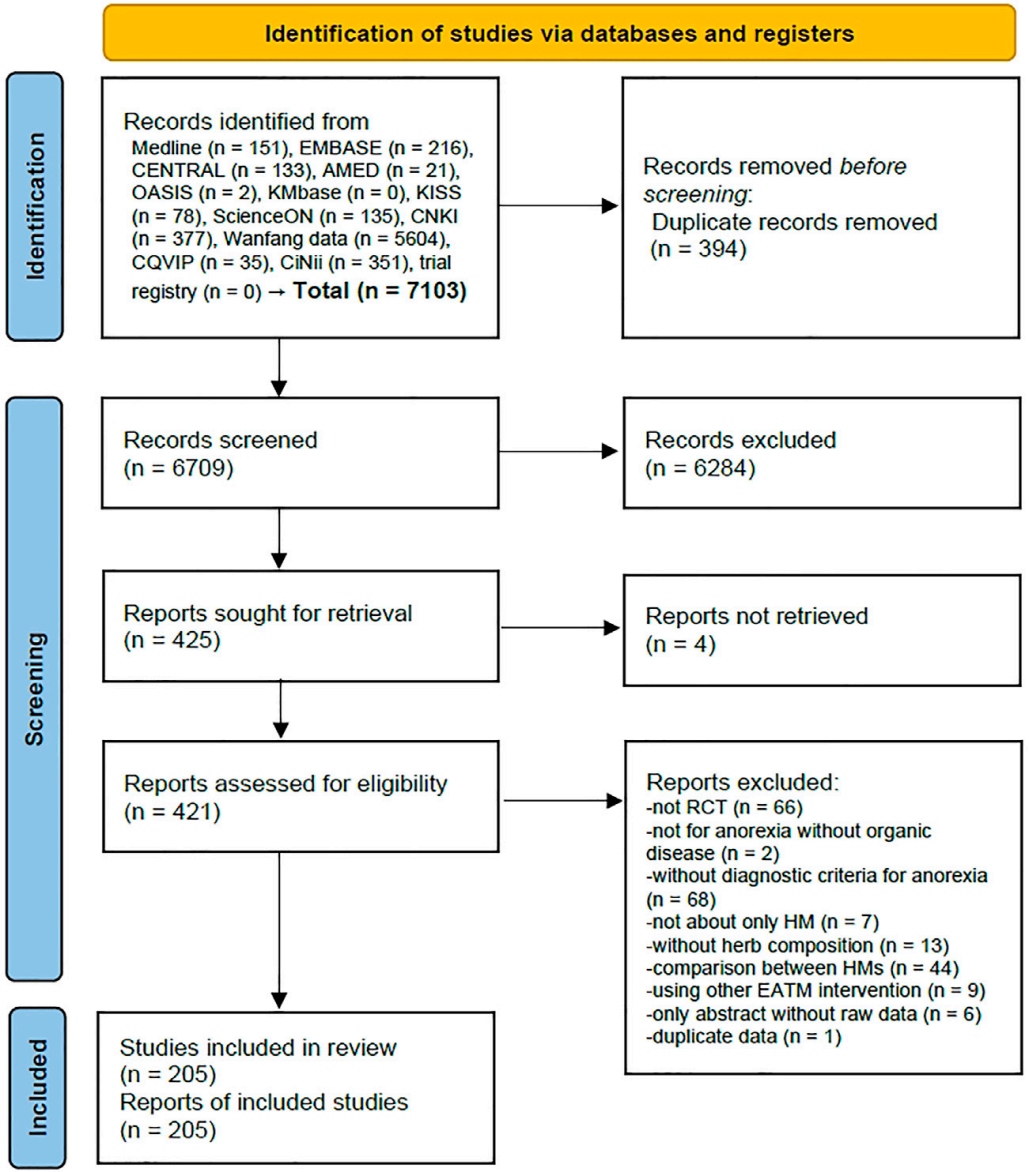
PRISMA 2020 flow diagram. No studies were identified from other sources (Google scholar and citation searching). Abbreviations: AMED, Allied and Complementary Medicine Database; CENTRAL, Cochrane Central Register of Controlled Trials; CNKI, China National Knowledge Infrastructure; CQVIP, Chongqing VIP; EATM, East Asian traditional medicine; HM, herbal medicine; KISS, Koreanstudies Information Service System; KMbase, Korean Medical Database; OASIS, Oriental Medicine Advanced Searching Integrated System; RCT, randomized controlled trial.

A total of 7,103 articles were identified from 12 electronic databases, and there were no additional records identified from the trial registry, Google Scholar, and reference lists of relevant articles. After removing 394 duplicate records using EndNote 20, 6,709 records were screened based on the titles and abstracts, and 425 eligible reports were further examined for retrieval. Among them, it was not possible to secure full-texts for 4 of them, and a full-text review was conducted for the remaining 421 articles. After removing 216 articles for the following reasons: 66 studies were not RCTs, 2 studies did not involve anorexia without organic disease, 68 studies did not have diagnostic criteria for anorexia, 7 studies were not about HM only, 13 studies did not have details on the herb composition, 44 studies were a comparison between HMs, 9 studies used other EATM interventions, 6 studies only had the abstract without raw data, and 1 study contained duplicate data (Supplementary File S2), finally, a total of 205 studies were included (Figure 1 and Supplementary File S3).

### 3.2 Study Characteristics

All included articles were published in Chinese in China from 1990 to 2021, and the study settings were all hospitals. Overall, 3 studies compared HM with placebo, 1 study compared HM with non-treatment, 135 studies compared HM with active control, and 72 studies compared HM plus active control with active control alone. As active controls for the control group, dietary supplements (e.g., zinc gluconate, multi-enzyme, and probiotics) and conventional medications (e.g., domperidone) were used. A total of 101 studies included only participants with anorexia corresponding to a specific pattern identification or mentioned the participants’ pattern identification. Most of the pattern identifications were related to the spleen-stomach. Among them, spleen-stomach qi deficiency was the most common in 28 studies, followed by spleen failing in transportation (22 studies), spleen deficiency with effulgent liver (18 studies), spleen-stomach disharmony (12 studies), and spleen-stomach yin deficiency (8 studies). A total of 23 studies reported that they received approval from the institutional review board before conducting the study, and 93 studies reported that informed consent was obtained from the participants. A total of 21 studies reported the funding sources for their research, and all of them received funding from the government or province (Supplementary File S3).

Various HMs were used in the included studies; among them, Xingpi Yanger granule was the most commonly used in 16 studies. In addition, there was a high frequency of using Shenling Baizhu powder (11 studies), Xiaoer Yanshi decoction (9 studies), and Yigong powder (8 studies). For the HM dosage form, most were used in the decoction form (130 studies), followed by the granule (57 studies) and pill (8 studies) forms. Analysis of the individual herbs constituting the basic HMs revealed that Crataegi Fructus was the most frequently used in 122 studies. Additionally, Atractylodis Rhizoma Alba (121 studies), Poria Sclerotium (113 studies), Citri Unshius Pericarpium (109 studies), Glycyrrhizae Radix (108 studies), and Hordei Fructus Germinatus (100 studies) were widely used herbs. In 50 studies, additional herbs were used in addition to basic HMs according to specific symptoms or pattern identifications. The HM administration period varied from 5 days to 3 months, among which 4 weeks (64 studies) and 2 weeks (42 studies) were the most common. Follow-up after HM administration was performed in 26 studies. The follow-up period varied widely from 1 week to 1 year, of which 6 months were the most frequent in 7 studies (Supplementary File S4).

### 3.3 Risk of Bias Assessment

A total of 57 studies appropriately generated random sequence numbers using randomization methods such as random number tables, and the risk of selection bias was evaluated to be low. The remaining studies did not report the random number generation method; thus, the risk was evaluated as unclear. In the case of performance bias, 3 studies were evaluated as low risk as they used placebo HM in the control group. On the other hand, 9 studies were evaluated as having a high risk of performance bias because single blinding was performed or the blinding of participants and personnel was not possible as the HM was compared with non-treatment. No studies mentioned allocation concealment and blinding of outcome assessors. Although there were missing values, 7 studies did not clearly state the reasons for the missing values or did not perform intention-to-treat analysis; thus, the risk of attrition bias was evaluated as high. A total of 17 studies reported that TER was calculated based on changes in body weight; however, raw data before and after treatment on body weight were not reported. Particularly, in some studies (Yuan, 2010; Yu, 2014), when body weight increased by 0.25 kg or more, it was judged as “markedly improved”, when it increased by less than 0.25 kg, it was judged as “improved”, and when there was no clear improvement, it was defined as “non-responder”. However, pre- and post-treatment analysis data for body weight were not presented, and only the calculated TER value was presented. Therefore, the risk of reporting bias was evaluated as high. In the other biases domain, 11 studies were evaluated as having an unclear risk because they did not mention the baseline homogeneity of the treatment group and the control group. In one study, the total number of the control group and the total number of men and women did not match; thus, the related risk was evaluated as high (Supplementary File S5).

### 3.4 Herbal Medicine Versus Placebo

Three studies compared HM with placebo. In the HM group of all studies, Xiaoer Piweile granule was administered for 4 weeks to anorexic patients with an identified pattern identification indicating spleen failing in transportation. The meta-analysis showed that the TER based on anorexia symptom improvement was significantly higher in the HM group (3 studies, RR 1.58, 95% CI 1.34, 1.85, *I* ^*2*^ = 77%). A study (Lin, 2012) reported that there was no difference in the body weight and serum hemoglobin levels between the two groups (*p* > 0.05, all). There was also no difference in the incidence of AEs between the two groups (2 studies, RR 0.66, 95% CI 0.25, 1.75) (Table 1).

**TABLE 1:** Results of meta-analysis and summary of findings.

Outcomes	Subgroup	No. participants (RCTs)	Anticipated absolute effects (95% CI)		Relative effect (95% CI)	*I* ^*2*^ value (%)	Quality of evidence (GRADE)	Comments
			Risk with control group	Risk with HM group				
HM versus placebo								
TER	Total (PI)	235 (3)	590 per 1,000	932 per 1,000 (790–1,000)	**RR 1.58 (1.34, 1.85)**	77	High	—
AE	Total (PI)	247 (3)	75 per 1,000	48 per 1,000 (18–128)	RR 0.66 (0.25, 1.75)	NA	Moderate	Imprecision (-1)
HM versus active control								
TER	Total	14,097 (130)	729 per 1,000	911 per 1,000 (889–933)	**RR 1.25 (1.22, 1.28)**	54	Low	Risk of bias (-1)
								Publication bias (-1)
—	PI	7,679 (69)	725 per 1,000	913 per 1,000 (884–949)	**RR 1.26 (1.22, 1.31)**	67	Very low	Risk of bias (-1)
								Inconsistency (-1)
								Publication bias (-1)
—	Not PI	6,418 (61)	733 per 1,000	909 per 1,000 (880–931)	**RR 1.24 (1.20, 1.27)**	23	Low	Risk of bias (-1)
								Publication bias (-1)
Food intake (g/day)	Total	395 (3)	—	**MD 7.23 higher (5.24 to 9.22 higher)**	—	96	Moderate	Risk of bias (-1)
—	PI	160 (1)	—	**MD 6.1 higher (4.09 to 8.11 higher)**	—	NA	Moderate	Risk of bias (-1)
—	Not PI	235 (2)	—	**MD 52.91 higher (40.11 to 65.71 higher)**	—	0	Moderate	Risk of bias (-1)
Body weight (kg)	Total	1,112 (11)	—	**MD 0.89 higher (0.23 to 1.54 higher)**	—	79	Very low	Risk of bias (-1)
								Inconsistency (-2)
—	PI	395 (4)	—	MD 0.72 higher (0.42 lower to 1.87 higher)	—	92	Very low	Risk of bias (-1)
								Inconsistency (-2)
								Imprecision (-1)
—	Not PI	717 (7)	—	**MD 0.96 higher (0.48 to 1.45 higher)**	—	0	Moderate	Risk of bias (-1)
Height (cm)	Total (PI)	161 (2)	—	MD 0.4 lower (4.3 lower to 3.51 higher)	—	0	Moderate	Risk of bias (-1)
BMI (kg/cm^2^)	Total	166 (2)	—	**MD 2.03 higher (1.57 to 2.49 higher)**	—	0	Moderate	Risk of bias (-1)
—	PI	94 (1)	—	**MD 1.85 higher (1.2 to 2.5 higher)**	—	NA	Low	Risk of bias (-1)
								Imprecision (-1)
—	Not PI	72 (1)	—	**MD 2.2 higher (1.55 to 2.85 higher)**	—	NA	Low	Risk of bias (-1)
								Imprecision (-1)
Leptin	Total	961 (11)	—	**SMD 1.08 lower (1.98 to 0.18 lower)**	—	97	Very low	Risk of bias (-1)
								Inconsistency (-2)
—	PI	737 (8)	—	SMD 0.33 lower (1.15 lower to 0.49 higher)	—	96	Very low	Risk of bias (-1)
								Inconsistency (-2)
								Imprecision (-1)
—	Not PI	224 (3)	—	**SMD 3.13 lower (4.53 to 1.73 lower)**	—	92	Moderate	Risk of bias (-1)
Gastrin (ng/L)	Total	532 (5)	—	**MD 13.23 higher (11.06 to 15.39 higher)**	—	0	Moderate	Risk of bias (-1)
—	PI	326 (3)	—	**MD 14.85 higher (11.19 to 18.52 higher)**	—	0	Moderate	Risk of bias (-1)
—	Not PI	206 (2)	—	**MD 12.36 higher (9.68 to 15.04 higher)**	—	57	Moderate	Risk of bias (-1)
Motilin (ng/L)	Total (PI)	431 (4)	—	**MD 28.6 higher (19.74 to 37.45 higher)**	—	14	Moderate	Risk of bias (-1)
NPY	Total	762 (8)	—	**SMD 1.02 higher (0.87 to 1.17 higher)**	—	50	Moderate	Risk of bias (-1)
—	PI	476 (5)	—	**SMD 0.9 higher (0.71 to 1.09 higher)**	—	12	Moderate	Risk of bias (-1)
—	Not PI	286 (3)	—	**SMD 1.22 higher (0.97 to 1.48 higher)**	—	64	Moderate	Risk of bias (-1)
Ghrelin	Total	349 (4)	—	**SMD 0.7 higher (0.47 to 0.93 higher)**	—	95	Very low	Risk of bias (-1)
								Inconsistency (-2)
—	PI	265 (3)	—	**SMD 0.44 higher (0.18 to 0.69 higher)**	—	94	Very low	Risk of bias (-1)
								Inconsistency (-2)
—	Not PI	84 (1)	—	**SMD 1.76 higher (1.25 to 2.26 higher)**	—	NA	Low	Risk of bias (-1)
								Imprecision (-1)
Orexin (ng/ml)	Total	146 (2)	—	**MD 1.67 higher (1.58 to 1.75 higher)**	—	99	Moderate	Risk of bias (-1)
—	PI	86 (1)	—	MD 0.18 higher (0.09 lower to 0.45 higher)	—	NA	Low	Risk of bias (-1)
								Imprecision (-1)
—	Not PI	60 (1)	—	**MD 1.84 higher (1.75 to 1.93 higher)**	—	NA	Low	Risk of bias (-1)
								Imprecision (-1)
Serum Zn (umol/L)	Total	1,410 (16)	—	**MD 3.87 higher (2.51 to 5.23 higher)**	—	88	Low	Risk of bias (-1)
								Publication bias (-1)
—	PI	1,129 (13)	—	**MD 3.08 higher (1.77 to 4.38 higher)**	—	83	Low	Risk of bias (-1)
								Publication bias (-1)
—	Not PI	281 (3)	—	MD 7.45 higher (1.06 lower to 15.96 higher)	—	96	Moderate	Risk of bias (-1)
Serum Fe (umol/L)	Total	141 (3)	—	**MD 1.36 higher (0.73 to 1.98 higher)**	—	92	Moderate	Risk of bias (-1)
—	PI	55 (2)	—	**MD 47.89 higher (29.98 to 65.8 higher)**	—	0	Low	Risk of bias (-1)
								Imprecision (-1)
—	Not PI	86 (1)	—	**MD 1.3 higher (0.68 to 1.92 higher)**	—	NA	Low	Risk of bias (-1)
								Imprecision (-1)
Serum Ca (mmol/L)	Total (PI)	245 (4)	—	**MD 0.03 higher (0 to 0.05 higher)**	—	79	Moderate	Risk of bias (-1)
Hb (g/L)	Total	846 (9)	—	**MD 9.78 higher (5.19 to 14.37 higher)**	—	92	Moderate	Risk of bias (-1)
—	PI	526 (5)	—	**MD 6.91 higher (1.34 to 12.48 higher)**	—	95	Moderate	Risk of bias (-1)
—	Not PI	320 (4)	—	**MD 15.37 higher (3.61 to 27.12 higher)**	—	86	Moderate	Risk of bias (-1)
Hair Zn (ppm)	Total	134 (2)	—	**MD 21.79 higher (16.91 to 26.67 higher)**	—	87	Very low	Risk of bias (-1)
								Inconsistency (-2)
—	PI	67 (1)	—	**MD 23.5 higher (18.47 to 28.53 higher)**	—	NA	Low	Risk of bias (-1)
								Imprecision (-1)
—	Not PI	67 (1)	—	MD 5.78 lower (25.99 lower to 14.43 higher)	—	NA	Low	Risk of bias (-1)
								Imprecision (-1)
Recurrence rate	Total	690 (8)	266 per 1,000	40 per 1,000 (24–67)	**RR 0.15 (0.09, 0.25)**	22	Low	Risk of bias (-1)
								Imprecision (-1)
—	PI	191 (3)	200 per 1,000	72 per 1,000 (32–164)	**RR 0.36 (0.16, 0.82)**	0	Low	Risk of bias (-1)
								Imprecision (-1)
—	Not PI	499 (5)	294 per 1,000	29 per 1,000 (15–59)	**RR 0.10 (0.05, 0.20)**	10	Low	Risk of bias (-1)
								Imprecision (-1)
AE	Total	3,059 (29)	10 per 1,000	3 per 1,000 (1–8)	**RR 0.34 (0.14, 0.80)**	0	Low	Risk of bias (-1)
								Imprecision (-1)
	PI	1760 (18)	10 per 1,000	5 per 1,000 (2–15)	RR 0.52 (0.18, 1.47)	14	Low	Risk of bias (-1)
								Imprecision (-1)
	Not PI	1,299 (11)	10 per 1,000	1 per 1,000 (0–8)	**RR 0.14 (0.03, 0.77)**	0	Low	Risk of bias (-1)
								Imprecision (-1)
HM + active control versus active control alone								
TER	Total	6,953 (70)	767 per 1,000	936 per 1,000 (905–959)	**RR 1.22 (1.18, 1.25)**	53	Low	Risk of bias (-1)
								Publication bias (-1)
—	PI	2,870 (28)	775 per 1,000	945 per 1,000 (899–999)	**RR 1.22 (1.16, 1.29)**	74	Low	Risk of bias (-1)
								Publication bias (-1)
—	Not PI	4,083 (42)	761 per 1,000	921 per 1,000 (898–944)	**RR 1.21 (1.18, 1.24)**	0	Low	Risk of bias (-1)
								Publication bias (-1)
Appetite recovery time (day)	Total	324 (4)	—	**MD 2.09 lower (2.44 to 1.75 lower)**	—	83	Moderate	Risk of bias (-1)
—	PI	104 (1)	—	**MD 1.42 lower (1.89 to 0.95 lower)**	—	NA	Moderate	Risk of bias (-1)
—	Not PI	220 (3)	—	**MD 2.88 lower (3.39 to 2.37 lower)**	—	0	Moderate	Risk of bias (-1)
Food intake (g/day)	Total	144 (2)	—	**MD 37.96 higher (23.13 to 52.78 higher)**	—	0	Moderate	Risk of bias (-1)
—	PI	74 (1)	—	**MD 44.1 higher (17.47 to 70.73 higher)**	—	NA	Low	Risk of bias (-1)
								Imprecision (-1)
—	Not PI	70 (1)	—	**MD 35.2 higher (17.35 to 53.05 higher)**	—	NA	Low	Risk of bias (-1)
								Imprecision (-1)
Body weight (kg)	Total	1,185 (13)	—	**MD 1.34 higher (1.03 to 1.66 higher)**	—	58	Moderate	Risk of bias (-1)
—	PI	410 (4)	—	**MD 1.15 higher (0.86 to 1.43 higher)**	—	0	Moderate	Risk of bias (-1)
—	Not PI	775 (9)	—	**MD 1.42 higher (0.98 to 1.85 higher)**	—	69	Moderate	Risk of bias (-1)
BMI (kg/cm^2^)	Total	549 (5)	—	**MD 0.89 higher (0.64 to 1.13 higher)**	—	87	Moderate	Risk of bias (-1)
—	PI	389 (4)	—	**MD 1.53 higher (1.17 to 1.88 higher)**	—	50	Moderate	Risk of bias (-1)
—	Not PI	160 (1)	—	MD 0.3 higher (0.04 lower to 0.64 higher)	—	NA	Moderate	Risk of bias (-1)
Subcutaneous fat thickness (cm)	Total (not PI)	332 (3)	—	**MD 0.09 higher (0.05 to 0.13 higher)**	—	0	Moderate	Risk of bias (-1)
Leptin	Total	803 (8)	—	**SMD 1.49 lower (2.37 to 0.61 lower)**	—	97	Very low	Risk of bias (-1)
								Inconsistency (-2)
—	PI	631 (6)	—	**SMD 1.75 lower (2.93 to 0.57 lower)**	—	97	Very low	Risk of bias (-1)
								Inconsistency (-2)
—	Not PI	172 (2)	—	**SMD 0.74 lower (1.2 to 0.28 lower)**	—	54	Moderate	Risk of bias (-1)
Gastrin (ng/L)	Total	407 (4)	—	**MD 14.75 higher (11.74 to 17.77 higher)**	—	81	Moderate	Risk of bias (-1)
—	PI	325 (3)	—	**MD 13.08 higher (9.95 to 16.21 higher)**	—	0	Moderate	Risk of bias (-1)
—	Not PI	82 (1)	—	**MD 35.88 higher (24.76 to 47 higher)**	—	NA	Low	Risk of bias (-1)
								Imprecision (-1)
Motilin (ng/L)	Total	415 (4)	—	**MD 31.34 higher (24.8 to 37.88 higher)**	—	44	Moderate	Risk of bias (-1)
—	PI	325 (3)	—	**MD 31.66 higher (24.77 to 38.55 higher)**	—	62	Moderate	Risk of bias (-1)
—	Not PI	90 (1)	—	**MD 28.48 higher (7.88 to 49.08 higher)**	—	NA	Low	Risk of bias (-1)
								Imprecision (-1)
NPY	Total	686 (7)	—	**SMD 1.11 higher (0.72 to 1.5 higher)**	—	82	Moderate	Risk of bias (-1)
—	PI	514 (5)	—	**SMD 1.03 higher (0.77 to 1.28 higher)**	—	47	Moderate	Risk of bias (-1)
—	Not PI	172 (2)	—	SMD 1.34 higher (0.4 lower to 3.08 higher)	—	96	Moderate	Risk of bias (-1)
Ghrelin	Total (PI)	358 (3)	—	**SMD 1.35 higher (1.12 to 1.58 higher)**	—	0	Moderate	Risk of bias (-1)
Serum Zn	Total	1,381 (15)	—	**SMD 0.92 higher (0.74 to 1.1 higher)**	—	61	Moderate	Risk of bias (-1)
—	PI	622 (7)	—	**SMD 0.73 higher (0.51 to 0.95 higher)**	—	41	Moderate	Risk of bias (-1)
—	Not PI	759 (8)	—	**SMD 1.09 higher (0.85 to 1.32 higher)**	—	55	Moderate	Risk of bias (-1)
Serum Fe	Total	503 (6)	—	**SMD 1.21 higher (0.72 to 1.7 higher)**	—	84	Moderate	Risk of bias (-1)
—	PI	170 (2)	—	**SMD 1.16 higher (0.69 to 1.63 higher)**	—	51	Moderate	Risk of bias (-1)
—	Not PI	333 (4)	—	**SMD 1.25 higher (0.49 to 2.02 higher)**	—	90	Moderate	Risk of bias (-1)
Serum Ca (mmol/L)	Total	442 (5)	—	**MD 0.17 higher (0.13 to 0.21 higher)**	—	87	Moderate	Risk of bias (-1)
—	PI	302 (3)	—	**MD 0.15 higher (0.1 to 0.21 higher)**	—	79	Moderate	Risk of bias (-1)
—	Not PI	140 (2)	—	**MD 0.2 higher (0.13 to 0.28 higher)**	—	95	Moderate	Risk of bias (-1)
Hb (g/L)	Total	793 (9)	—	**MD 8.47 higher (5.59 to 11.35 higher)**	—	72	Moderate	Risk of bias (-1)
—	PI	144 (2)	—	**MD 8.24 higher (5.74 to 10.74 higher)**	—	0	Moderate	Risk of bias (-1)
—	Not PI	649 (7)	—	**MD 8.51 higher (4.48 to 12.53 higher)**	—	79	Moderate	Risk of bias (-1)
Recurrence rate	Total	450 (6)	342 per 1,000	113 per 1,000 (79–164)	**RR 0.33 (0.23, 0.48)**	0	Low	Risk of bias (-1)
								Imprecision (-1)
—	PI	254 (3)	273 per 1,000	101 per 1,000 (57–175)	**RR 0.37 (0.21, 0.64)**	0	Low	Risk of bias (-1)
								Imprecision (-1)
—	Not PI	196 (3)	444 per 1,000	133 per 1,000 (80–222)	**RR 0.30 (0.18, 0.50)**	0	Low	Risk of bias (-1)
								Imprecision (-1)
AE	Total	2,791 (29)	12 per 1,000	7 per 1,000 (3–15)	RR 0.58 (0.27, 1.24)	8	Low	Risk of bias (-1)
								Imprecision (-1)
—	PI	808 (9)	10 per 1,000	7 per 1,000 (2–31)	RR 0.75 (0.18, 3.14)	NA	Low	Risk of bias (-1)
								Imprecision (-1)
—	Not PI	1983 (20)	14 per 1,000	7 per 1,000 (3–18)	RR 0.53 (0.22, 1.30)	38	Low	Risk of bias (-1)
								Imprecision (-1)

Abbreviations: AE, adverse event; BMI, body mass index; CI, confidence interval; GRADE, grading of recommendations assessment, development, and evaluation; Hb, hemoglobin; HM, herbal medicine; MD, mean difference; NA, not applicable; NPY, neuropeptide Y; PI, pattern identification; RCT, randomized controlled trial; RR, risk ratio; SMD, standardized mean difference; TER, total effective rate.

Bold values mean significant differences between the groups.

### 3.5 Herbal Medicine Versus No Treatment

A study (Lian et al., 2020) compared HM with non-treatment; in the HM group, Tiaozhong decoction was administered for 4 weeks to anorexic patients with an identified pattern identification indicating spleen deficiency with effulgent liver. Lian et al. (Lian et al., 2020) reported that the TER and serum leptin level was significantly higher in the HM group than in the non-treatment group (*p* < 0.05, all).

### 3.6 Herbal Medicine Versus Active Control

A total of 135 studies compared HM with active control, and the meta-analysis revealed that the TER (130 studies, RR 1.25, 95% CI 1.22, 1.28, *I* ^*2*^ = 54%), food intake (3 studies, MD 7.23 g/day, 95% CI 5.24, 9.22, *I* ^*2*^ = 96%), body weight (11 studies, MD 0.89 kg, 95% CI 0.23, 1.54, *I* ^*2*^ = 79%), and BMI (2 studies, MD 2.03, 95% CI 1.57, 2.49, *I* ^*2*^ = 0%) were significantly higher in the HM group after treatment. In addition, both the levels of blood biomarkers related to gastrointestinal function (leptin, gastrin, motilin, neuropeptide Y (NPY), ghrelin, and orexin) and nutrition indices (serum Zn, Fe, and Ca, hemoglobin, and hair Zn) were significantly improved in the HM group compared with the active control group. The recurrence rate of anorexia (8 studies, RR 0.15, 95% CI 0.09, 0.25, *I* ^*2*^ = 2%) and the incidence of AEs (29 studies, RR 0.34, 95% CI 0.14, 0.80, *I* ^*2*^ = 0%) were significantly lower in the HM group. In subgroup analysis based on whether the pattern identification of the participants was specified, the TER, food intake, BMI, levels of gastrin, NPY, ghrelin, serum Fe, and hemoglobin, and recurrence rate showed a consistent HM effect regardless of pattern identification; however, the rest of the outcome indicators did not show a similar effect (Table 1). An evaluation of the risk of publication bias by Egger’s test showed that there was no risk of publication bias in the body weight (*p* = 0.104), serum leptin (*p* = 0.057), and incidence of AEs (*p* = 0.851); however, there was a risk of publication bias in the TER (*p* < 0.001) and serum Zn (*p* = 0.006).

### 3.7 Herbal Medicine Plus Active Control Versus Active Control Alone

A total of 72 studies compared HM plus active control with active control alone, and the symptoms of anorexia measured according to the TER (70 studies, RR 1.22, 95% CI 1.18, 1.25, *I* ^*2*^ = 53%), appetite recovery time (4 studies, MD −2.09 days, 95% CI -2.44, -1.75, *I* ^*2*^ = 83%), and food intake (2 studies, MD 37.96 g/day, 95% CI 23.13, 52.78, *I* ^*2*^ = 0%) and all body measurement indicators including body weight (13 studies, MD 1.34 kg, 95% CI 1.03, 1.66, *I* ^*2*^ = 58%), BMI (5 studies, MD 0.89, 95% CI 0.64, 1.13, *I* ^*2*^ = 87%), and subcutaneous fat thickness (3 studies, MD 0.09 cm, 95% CI 0.05, 0.13, *I* ^*2*^ = 0%) were significantly improved in the HM group compared with the control group. Additionally, both the levels of blood biomarkers related to gastrointestinal function (leptin, gastrin, motilin, NPY, and ghrelin) and nutrition indices (serum Zn, Fe, and Ca and hemoglobin) were significantly improved in the HM group. The recurrence rate of anorexia was significantly lower in the HM group (5 studies, RR 0.33, 95% CI 0.23, 0.48, *I* ^*2*^ = 0%), and the incidence of AEs was not significantly different between the two groups (29 studies, RR 0.58, 95% CI 0.27, 1.24, *I* ^*2*^ = 8%). In subgroup analysis based on whether the pattern identification of the participants was specified, all results except the BMI and NPY level consistently showed the significant effect of HM (Table 1). An evaluation of the risk of publication bias by Egger’s test showed there was no risk of publication bias in the body weight (*p* = 0.05), serum Zn (*p* = 0.812), and incidence of AEs (*p* = 0.779); however, there was a risk of publication bias in the TER (*p* < 0.001).

### 3.8 Quality of Evidence

When HM and placebo were compared, the quality of evidence for the main outcome was moderate to high, and imprecision caused by a wide CI was the reason for the downgrade. When HM and active control or HM plus active control and active control alone were compared, the quality of evidence for the main outcome was very low to moderate. In particular, the risk of bias, inconsistency, imprecision, and publication bias of meta-analysis results were the reasons for the downgrade (Table 1).

## 4 Discussion

### 4.1 Findings of This Review

This systematic review provides the most comprehensive review of HMs for anorexia in children. Based on the results of meta-analysis, there was some clinical evidence showing that HM as monotherapy or adjuvant therapy for children with anorexia could improve the clinical symptoms of anorexia (assessed by TER, appetite recovery time, and food intake), some anthropometric outcomes (including body weight and BMI), and some biological markers related to appetite and growth (including leptin, gastrin, motilin, NPY, ghrelin, orexin, and serum Zn, Fe, and Ca). HM was also found to significantly reduce the recurrence rate of anorexia and incidence of AEs in a head-to-head comparison with active controls such as dietary supplements or conventional medications in this population. However, the methodological quality of the included studies (evaluated as the risk of bias) was not optimal, and information for evaluating the risk of bias in selection bias, performance bias, and detection bias was not well described. Overall, the quality of evidence (evaluated using the GRADE approach) for the findings of the meta-analysis ranged from very low to moderate, with most cases evaluated as moderate.

### 4.2 Clinical Implication

As a standard clinical practice guideline for the diagnosis and management of children with anorexia has not been developed, it is clinically meaningful to analyze the efficacy and safety of HM, which is widely used in Asian countries (Yoon et al., 2003; Choi et al., 2010). Therefore, this review was conducted to summarize the available evidence. The findings of this review highlight the heterogeneity of the HMs used and the lack of high-quality trials, along with the potential of HM therapies in the management of children with anorexia. Specifically, to optimize the management of this condition with HM, the HM prescription strategy should be standardized, and more robust clinical trials should be conducted.

Pattern identification is a unique diagnostic system of EATM, and HM tends to rely on pattern identification rather than a diagnosed disease (Li and Xu, 2015). In other words, even in patients with different diagnosed diseases, if they have the same identified pattern identification, the same or at least a similar HM will likely be prescribed. On the other hand, in patients with the same diagnosed disease, if they have different identified pattern identifications, different HMs will likely be prescribed. Therefore, the standardization of pattern identification can help overcome the barriers of using heterogeneous HMs, thus promoting precision medicine (Li and Xu, 2015). Standardized pattern identification clusters heterogeneous HMs, and this clustering can lead to the standardization of the HM prescription strategy. Around half of the included studies in this systematic review included only participants with anorexia corresponding to a specific pattern identification or mentioned the participants’ pattern identification. In addition, most of them were related to the spleen-stomach. We conducted subgroup analysis based on whether the pattern identification of the participants was specified to investigate statistical and clinical heterogeneity and to ensure that the results are consistent. Although the results of subgroup analysis for the various outcome measures were not consistent and statistical heterogeneity was often unresolved in our study, the authors identified some specific pattern identifications frequently observed in children with anorexia, including spleen-stomach qi deficiency and spleen failing in transportation. It is important to explore the standardization of pattern identification for this condition in further studies such as expert consensus and large prospective cohort studies.

This review identified specific individual herbs that were used frequently, including Crataegi Fructus, Atractylodis Rhizoma Alba, Poria Sclerotium, Citri Unshius Pericarpium, Glycyrrhizae Radix, and Hordei Fructus Germinatus. Among them, the most frequently used herbs including Crataegii Fructus, Atractylodis Rhizoma Alba, Citri Unshius Pericarpium, and Hordei Fructus Germiniatus are effective in promoting digestion and invigorating the stomach. Furthermore, Poria Sclerotium and Glycyrrhizae Radix are the component herbs of Rikkunshito, a well-known herbal ghrelin potentiator (Fujitsuka and Uezono, 2014). However, there are limited studies on the appetite-stimulating mechanisms of these herbs. Considering the potentially beneficial properties of the complex components of the herbs (Wang et al., 2012), the nutritional, psychological, and physiological effects of these herbs for the treatment of anorexia in children should be comprehensively reviewed. The findings could help standardize and optimize HM therapies for this condition in the future.

### 4.3 Limitations and Suggestions for Further Studies

Although we provide the most comprehensive systematic review on anorexia in children for the first time, some limitations should be considered. First, among the included studies, only a few studies compared HM with placebo. In most of the included studies, the risk of performance bias was evaluated to be unclear; thus, the possibility that the appetite-promoting effect of HM may be exaggerated cannot be excluded. In future controlled clinical trials, it is important to minimize the possibility of performance bias, specifically by comparing the effects of HM with those of placebo. Second, all included studies were conducted in China with Chinese subjects. Therefore, the results of this review are not only less generalizable but also vulnerable to the placebo effect because individuals in China may be more likely than those in Western countries to be familiar with EATM treatment modalities such as HM. To overcome the generalization issue, future studies should include other countries such as Korea, Taiwan, and Japan, which have incorporated HM therapies in the national medical system. In addition, it would be ideal to evaluate the participants’ familiarity and expectations for HM in trials and consider them in the interpretation of the results. Third, the diagnostic criteria for anorexia in children used in the included studies were all developed locally in China, and there was no internationally accepted diagnostic standard, which restricts the generalizability of the results for future studies. Therefore, it is recommended that future studies include internationally accepted diagnostic criteria for anorexia, such as infantile anorexia in the Chatoor Diagnostic Criteria for Feeding Disorder (Levine et al., 2011). Fourth, despite our efforts to interpret the heterogeneity through a subgroup analysis, considering the significant clinical heterogeneity among the included studies such as the age of the participants and the HMs used, caution is required when interpreting the meta-analysis results. Finally, the pre-planned sensitivity analysis could not be performed in this review because the included studies had an unclear risk of bias in most domains. This finding highlights the need for better quality clinical trials in the future.

## 5 Conclusion

According to the findings, there was a low to moderate quality of evidence showing that HM as monotherapy or adjuvant therapy for children with anorexia could improve clinical symptoms of anorexia, anthropometric outcomes, and biological markers related to appetite and growth. However, the methodological quality of the included studies was generally poor, and there was heterogeneity in the HMs used. Therefore, future high-quality clinical trials using a placebo as the control and research focusing on the standardization of HM prescription for children with anorexia should be performed.

## Data Availability

The original contributions presented in the study are included in the article/Supplementary Material, further inquiries can be directed to the corresponding author.
