# Supplementary material for: Herbal Medicine for the Treatment of Anorexia in Children: A Systematic Review and Meta-Analysis
Source: Front Pharmacol. 2022 Apr 1;13:839668. doi: 10.3389/fphar.2022.839668 (PMC9012502; doi:10.3389/fphar.2022.839668)
Supplement: Supplementary file 1 [file Table1.DOCX]

**Supplement 1. Search terms used in each database and results**

**MEDLINE via PubMed**

|  | Searches | Results |
| --- | --- | --- |
| #1 | Anorexia[MH] OR Appetite[MH] OR “Food Fussiness”[MH] OR “Feeding and Eating Disorders”[MH] OR anorexi*[TIAB] OR anorectic[TIAB] OR hypophagia[TIAB] OR oligophag*[TIAB] OR appetite*[TIAB] OR orexia[TIAB] OR “insufficient intake”[TIAB] OR “decreased food intake”[TIAB] OR “food fussiness*”[TIAB] OR "food pickiness*"[TIAB] OR “fussy eater*”[TIAB] OR “picky eater*”[TIAB] OR “food refusal”[TIAB] OR “food aversion*”[TIAB] OR “aversion to food”[TIAB] OR “food phobia”[TIAB] OR “food neophobia”[TIAB] OR feeding[TIAB] OR eating[TIAB] OR “avoidant restrictive food intake disorder”[TIAB] OR ARFID[TIAB] | 343,708 |
| #2 | Child[MH] OR Pediatrics[MH] OR Infant[MH] OR Adolescent[MH] OR Minors[MH] OR child*[TIAB] OR pediatric*[TIAB] OR infant[TIAB] OR neonate[TIAB] OR newborn[TIAB] OR adolescent[TIAB] OR baby[TIAB] | 4,226,673 |
| #3 | “Plants, Medicinal”[MH] OR “Drugs, Chinese Herbal”[MH] OR “Medicine, Chinese Traditional”[MH] OR “Medicine, Kampo”[MH] OR “Medicine, Korean Traditional”[MH] OR “Herbal Medicine”[MH] OR “traditional Korean medicine”[TIAB] OR “traditional Chinese medicine”[TIAB] OR “traditional oriental medicine”[TIAB] OR “Kampo medicine”[TIAB] OR herb*[TIAB] OR decoction*[TIAB] OR botanic*[TIAB] | 227,417 |
| #4 | “randomized controlled trial”[PT] OR “controlled clinical trial”[PT] OR randomized[TIAB] OR placebo[TIAB] OR “drug therapy”[SH] OR randomly[TIAB] OR trial[TIAB] OR groups[TIAB] | 5,088,905 |
| #5 | animals[MH] NOT humans[MH] | 4,836,407 |
| #6 | #1 AND #2 AND #3 AND #4 NOT #5 | **151** |

**EMBASE via Elsevier**

|  | Searches | Results |
| --- | --- | --- |
| #1 | anorexia/exp OR appetite/exp OR 'food fussiness'/exp OR 'feeding disorder'/exp OR 'eating disorder'/exp OR 'appetite disorder'/exp OR anorexi*:ab,ti OR anorectic:ab,ti OR hypophagia:ab,ti OR oligophag*:ab,ti OR appetite*:ab,ti OR orexia:ab,ti OR 'insufficient intake':ab,ti OR 'decreased food intake':ab,ti OR 'food fussiness*':ab,ti OR 'food pickiness*':ab,ti OR 'fussy eater*':ab,ti OR 'picky eater*':ab,ti OR 'food refusal':ab,ti OR 'food aversion*':ab,ti OR 'aversion to food':ab,ti OR 'food phobia':ab,ti OR 'food neophobia':ab,ti OR feeding:ab,ti OR eating:ab,ti OR 'avoidant restrictive food intake disorder':ab,ti OR ARFID:ab,ti | 519,825 |
| #2 | child/exp OR pediatrics/exp OR infant/exp OR adolescent/exp OR ‘minor (person)’/exp OR child*:ab,ti OR pediatric*:ab,ti OR infant:ab,ti OR neonate:ab,ti OR newborn:ab,ti OR adolescent:ab,ti OR baby:ab,ti | 4,579,701 |
| #3 | ‘medicinal plant’/exp OR ‘medicinal plant’:ab,ti OR ‘herbaceous agent’/exp OR ‘herbaceous agent’:ab,ti OR ‘chinese medicine’/exp OR ‘chinese medicine’:ab,ti OR ‘kampo medicine’/exp OR ‘kampo medicine’:ab,ti OR ‘kampo medicine (drug)’/exp OR ‘kampo medicine (drug)’:ab,ti OR ‘korean medicine’/exp OR ‘korean medicine’:ab,ti OR ‘herbal medicine’/exp OR ‘herbal medicine’:ab,ti OR ‘oriental medicine’/exp OR ‘oriental medicine’:ab,ti OR herb/exp OR herb*:ab,ti OR decoction*:ab,ti OR botanic*:ab,ti | 457,763 |
| #4 | 'crossover procedure':de OR 'double-blind procedure':de OR 'randomized controlled trial':de OR 'single-blind procedure':de OR (random* OR factorial* OR crossover* OR cross NEXT/1 over* OR placebo* OR doubl* NEAR/1 blind* OR singl* NEAR/1 blind* OR assign* OR allocat* OR volunteer*):de,ab,ti | 2,756,061 |
| #5 | #1 AND #2 AND #3 AND #4 | **216** |

**CENTRAL**

|  | Searches | Results |
| --- | --- | --- |
| #1 | MeSH descriptor: [Anorexia] explode all trees | 401 |
| #2 | MeSH descriptor: [Appetite] explode all trees | 1,496 |
| #3 | MeSH descriptor: [Food Fussiness] explode all trees | 4 |
| #4 | MeSH descriptor: [Feeding and Eating Disorders] explode all trees | 1,690 |
| #5 | (anorexi* OR anorectic OR hypophagia OR oligophag* OR appetite* OR orexia OR “insufficient intake” OR “decreased food intake” OR “food fussiness*” OR "food pickiness*" OR “fussy eater*” OR “picky eater*” OR “food refusal” OR “food aversion*” OR “aversion to food” OR “food phobia” OR “food neophobia” OR feeding OR eating OR “avoidant restrictive food intake disorder” OR ARFID):ti,ab,kw | 47,786 |
| #6 | #1 OR #2 OR #3 OR #4 OR #5 | 47,832 |
| #7 | MeSH descriptor: [Child] explode all trees | 57,420 |
| #8 | MeSH descriptor: [Pediatrics] explode all trees | 696 |
| #9 | MeSH descriptor: [Infant] explode all trees | 32,816 |
| #10 | MeSH descriptor: [Adolescent] explode all trees | 105,819 |
| #11 | MeSH descriptor: [Minors] explode all trees | 10 |
| #12 | (child* OR pediatric* OR infant OR neonate OR newborn OR adolescent OR baby):ti,ab,kw | 284,006 |
| #13 | #7 OR #8 OR #9 OR #10 OR #11 OR #12 | 284,015 |
| #14 | MeSH descriptor: [Plants, Medicinal] explode all trees | 946 |
| #15 | MeSH descriptor: [Drugs, Chinese Herbal] explode all trees | 3,645 |
| #16 | MeSH descriptor: [Medicine, Chinese Traditional] explode all trees | 1,219 |
| #17 | MeSH descriptor: [Medicine, Kampo] explode all trees | 46 |
| #18 | MeSH descriptor: [Medicine, Korean Traditional] explode all trees | 33 |
| #19 | MeSH descriptor: [Herbal Medicine] explode all trees | 63 |
| #20 | (“traditional Korean medicine” OR “traditional Chinese medicine” OR “traditional oriental medicine” OR “Kampo medicine” OR herb* OR decoction* OR botanic*):ti,ab,kw | 18,819 |
| #21 | #14 OR #15 OR #16 OR #17 OR #18 OR #19 OR #20 | 19,970 |
| #22 | (#6 AND #13 AND #21) in Trials | **133** |

**AMED via EBSCO**

|  | Searches | Results |
| --- | --- | --- |
| #1 | Anorexia[SU] OR Appetite[SU] OR “Food Fussiness”[SU] OR “Feeding and Eating Disorders”[SU] OR anorexi*[TX] OR anorectic[TX] OR hypophagia[TX] OR oligophag*[TX] OR appetite*[TX] OR orexia[TX] OR “insufficient intake”[TX] OR “decreased food intake”[TX] OR “food fussiness*”[TX] OR "food pickiness*"[TX] OR “fussy eater*”[TX] OR “picky eater*”[TX] OR “food refusal”[TX] OR “food aversion*”[TX] OR “aversion to food”[TX] OR “food phobia”[TX] OR “food neophobia”[TX] OR feeding[TX] OR eating[TX] OR “avoidant restrictive food intake disorder”[TX] OR ARFID[TX] | 2,791 |
| #2 | Child[SU] OR Pediatrics[SU] OR Infant[SU] OR Adolescent[SU] OR Minors[SU] OR child*[TX] OR pediatric*[TX] OR infant[TX] OR neonate[TX] OR newborn[TX] OR adolescent[TX] OR baby[TX] | 31,853 |
| #3 | “Plants, Medicinal”[SU] OR “Drugs, Chinese Herbal”[SU] OR “Medicine, Chinese Traditional”[SU] OR “Medicine, Kampo”[SU] OR “Medicine, Korean Traditional”[SU] OR “Herbal Medicine”[SU] OR “traditional Korean medicine”[TX] OR “traditional Chinese medicine”[TX] OR “traditional oriental medicine”[TX] OR “Kampo medicine”[TX] OR herb*[TX] OR decoction*[TX] OR botanic*[TX] | 34,563 |
| #4 | #1 AND #2 AND #3 | **21** |

**OASIS**

|  | Searches | Results |
| --- | --- | --- |
| #1 | (식욕\|편식\|음식\|급식\|섭식\|식사\|식이\|섭취) (소아\|아동) (한약\|약초\|본초\|탕\|환\|산) | **2** |

**KMbase**

|  | Searches | Results |
| --- | --- | --- |
| #1 | ((((((([ALL=식욕] OR [ALL=편식]) OR [ALL=음식]) OR [ALL=급식]) OR [ALL=섭식]) OR [ALL=식사]) OR [ALL=식이]) OR [ALL=섭취]) | 10,638 |
| #2 | ([ALL=소아] OR [ALL=아동]) | 19,066 |
| #3 | ((((([ALL=한약] OR [ALL=약초]) OR [ALL=본초]) OR [ALL=탕]) OR [ALL=환]) OR [ALL=산]) | 113,315 |
| #4 | #1 AND #2 AND #3 | **0** |

**KISS**

|  | Searches | Results |
| --- | --- | --- |
| #1 | 제목=(식욕\|편식\|음식\|급식\|섭식\|식사\|식이\|섭취) AND 초록=(소아\|아동) AND 초록=(한약\|약초\|본초\|탕\|환\|산) | **78** |

**ScienceON**

|  | Searches | Results |
| --- | --- | --- |
| #1 | (식욕\|편식\|음식\|급식\|섭식\|식사\|식이\|섭취) (소아\|아동) (한약\|약초\|본초\|탕\|환\|산) | **135** |

**CNKI**

|  | Searches | Results |
| --- | --- | --- |
| #1 | (SU='厌食'+'厌食症'+'恶食'+‘不思食'+‘拒食'+'挑食'+'摄入不足'+'食减'+'食欲'+'食慾'+'食欲缺乏'+'胃口'+'食物恐惧症'+'新食物恐惧症'+'进食障碍'+'避免限制性进食障碍') AND (SU='小儿'+'儿童'+'儿'+'小孩'+'孩子') AND (SU='中医药'+'中医'+'中西医结合'+'中药'+'汤'+'丸'+'散'+'饮'+'颗粒'+'胶囊'+'自拟') AND (SU='随机'+'对照'+'随意'+'试验'+'安慰') | **377** |

**Wanfang data**

|  | Searches | Results |
| --- | --- | --- |
| #1 | (主题:厌食 OR 主题:厌食症 OR 主题:恶食 OR 主题:不思食 OR 主题:拒食 OR 主题:挑食 OR 主题:摄入不足 OR 主题:食减 OR 主题:食欲 OR 主题:食慾 OR 主题:食欲缺乏 OR 主题:胃口 OR 主题:食物恐惧症 OR 主题:新食物恐惧症 OR 主题:进食障碍 OR 主题:避免限制性进食障碍) AND (主题:小儿 OR 主题:儿童 OR 主题:儿 OR 主题:小孩 OR 主题:孩子) AND (主题:中医药 OR 主题:中医 OR 主题:中西医结合 OR 主题:中药 OR 主题:汤 OR 主题:丸 OR 主题:散 OR 主题:饮 OR 主题:颗粒 OR 主题:胶囊 OR 主题:自拟) AND (主题:随机 OR 主题:对照 OR 主题:随意 OR 主题:试验 OR 主题:安慰) | **5,604** |

**VIP.**

|  | Searches | Results |
| --- | --- | --- |
| #1 | (M=(厌食 OR 厌食症 OR 恶食 OR 不思食 OR 拒食 OR 挑食 OR 摄入不足 OR 食减 OR 食欲 OR 食慾 OR 食欲缺乏 OR 胃口 OR 食物恐惧症 OR 新食物恐惧症 OR 进食障碍 OR 避免限制性进食障碍) AND M=(小儿 OR 儿童 OR 儿 OR 小孩 OR 孩子) AND M=(中医药 OR 中医 OR 中西医结合 OR 中药 OR 汤 OR 丸 OR 散 OR 饮 OR 颗粒 OR 胶囊 OR 自拟) AND M=(随机 OR 对照 OR 随意 OR 试验 OR 安慰)) | **35** |

**CiNii**

|  | Searches | Results |
| --- | --- | --- |
| #1 | (拒食 OR 食欲 OR 食慾 OR 無食欲 OR 食欲不振 OR 食欲低下 OR 摂取 OR 摂食 OR 摂食障害) AND (小児 OR 幼子 OR 児子 OR 子 OR 児 OR 児童 OR 幼児 OR 乳兒 OR 子供 OR 思春期の OR 青春の) AND (漢方薬 OR ハーブ OR 散 OR 汤 OR 丸) AND (ランダム化比較試験 OR 対照臨床試験 OR ランダム OR 無作為 OR 対照 OR 試験 OR 偽薬) | **351** |
