# Supplementary material for: Herbal Medicine for the Treatment of Anorexia in Children: A Systematic Review and Meta-Analysis
Source: Front Pharmacol. 2022 Apr 1;13:839668. doi: 10.3389/fphar.2022.839668 (PMC9012502; doi:10.3389/fphar.2022.839668)
Supplement: Supplementary file 2 [file Table2.DOCX]

**Supplement 2. Excluded studies after full-text reviewing**

**1) not RCT: 66**

1. 胡丹, 叶峰. 加味三仙汤加减治疗小儿厌食症效果观察. 中国乡村医药. 2014;21(05):29-30.

2. 邵功利, 林忠嗣, 王继艳. 加味逍遥散治疗小儿肝郁脾虚型厌食症的研究. 中医药学刊. 2006;24(4):667-8.

3. 张雅凤, 林忠嗣, 卞镝, 张好姝, 周淑梅. 加味厌食散治疗肝郁脾虚型厌食患儿临床观察. 中华中医药学刊. 2007;25(5):1030-2.

4. 全文哲. 适贝高儿宝颗粒治疗儿童厌食症60例. 陕西中医. 2004;25(11).

5. 杜德平. 脾胃虚弱型小儿厌食症采用中医保健疗法治疗的疗效观察分析. 健康之友. 2020(1):187-8.

6. 张永生, 冯金花. 四君子汤治疗中虚积滞型小儿厌食症的临床分析. 中国中医药现代远程教育. 2015;13(24):23-4.

7. 强艳, 程毓清. 三种不同疗法治疗儿童厌食症效果分析. 中国临床康复. 2003;7(27):3771-.

8. 耿芳. 醒脾养儿颗粒联合双歧杆菌三联活菌胶囊及补锌疗法治疗小儿厌食症的临床研究. 疾病监测与控制. 2020;14(05):361-3+72.

9. 黄丹. 醒脾养儿颗粒联合双歧杆菌三联活菌胶囊同步补锌疗法治疗98例小儿厌食症的临床研究. 医药论坛杂志. 2019;40(02):143-4.

10. 袁美凤，许华，曾繁冬，何盛琪. 醒脾开胃口服液治疗小儿厌食症的临床研究. 中药新药与临床药理. 1995(02):17-9.

11. 李建平. 逍遥散加减治疗小儿厌食症的疗效. 家庭医药. 2018(7):45.

12. 张明. 莪术四君汤治疗小儿厌食30例：附健脾汤治疗28例对照观察. 浙江中医杂志. 1999;34(7).

13. 罗飞. 自拟厌食方治疗小儿厌食症89例. 安徽中医临床杂志. 2002;14(2):118.

14. 魏冬锋, 兰金. 中西医结合治厌食症脾胃虚弱型临床观察. 实用中医药杂志. 2020;36(1):42-3.

15. 杨桂霞, 杨秀珍. 中西医结合治疗缺锌引起小儿厌食症40例. 陕西中医. 2001;22(5):278-9.

16. 李万田. 中西医结合治疗小儿厌食症. 现代中西医结合杂志. 1994:75-6.

17. 王新秋, 王礼马. 中西医结合治疗小儿厌食症51例. 时珍国医国药. 2004;15(4):256-.

18. 吴玉玲. 中西医结合治疗小儿厌食症68例临床观察. 临床医药文献电子杂志. 2018;5(56):72.

19. 杨明武, 付晓萍. 中西医结合治疗小儿厌食症的临床体会. 中西医结合心血管病电子杂志. 2015(18):42-,4.

20. 白学忠. 中西医结合治疗小儿厌食症的疗效观察. 中西医结合心血管病电子杂志. 2015;3(29):110-1.

21. 董凡诗. 中西药结合治疗小儿厌食症的临床研究. 中国社区医师（医学专业）. 2013;15(1):192.

22. 木阿热木·阿不都克热, 陈淑平. 中西药联合治疗小儿厌食症的疗效观察. 中国社区医师（医学专业）. 2011;13(34):186.

23. 范慧慧. 中医辨证治疗小儿厌食症临床体会. 世界最新医学信息文摘（连续型电子期刊）. 2019;19(18):198-9.

24. 路敬坤. 中医保健疗法治疗脾胃虚弱型小儿厌食症疗效观察. 健康之友. 2019(15):140.

25. 童建霞. 中医药治疗小儿厌食症临床经验探析. 中国保健营养. 2019;29(26):81-2.

26. 吴秀清, 赖柏玉, 范章辉, 李忠冠. 中药和葡萄糖酸锌治疗儿童缺锌性厌食92例. 广东微量元素科学. 2004;11(5):38-9.

27. 赖柏玉, 范纪钦. 中药和葡萄糖酸锌治疗儿童缺锌性厌食100例. 广东微量元素科学. 2003;10(4):45-6.

28. 赵玉屏. 七味白术散与多酶片结合治疗小儿厌食症50例. 中原医刊. 2004;31(23):22-3.

29. 孙升云. 炮穿山甲为主治疗小儿厌食症. 中医杂志. 2002;43(2):95.

30. 张旭松, 张秀萍. 双歧杆菌三联活菌散联合七味白术散治疗儿童厌食症的疗效及对食欲调节因子的影响. 中国中西医结合消化杂志. 2016;24(3):219-20,23.

31. 秦晓莉. 双歧三联活菌胶囊联合葡萄糖酸锌治疗小儿厌食症临床疗效分析. 中国卫生标准管理. 2016;7(10):115-6.

32. 郭明玉, 陈荣坤. 异功散加味治疗小儿厌食症368例. 江西中医药. 2001;32(6):43.

33. 雷后兴. 畲族民间验方消积汤治疗小儿厌食症196例疗效观察. 中国民族医药杂志. 2007;13(3):15-6.

34. 何斌, 张高东. 苏藿三仙汤治疗小儿厌食症78例. 实用中医药杂志. 2002;18(7):18-9.

35. 刘克丽, 谢静, 张丽霞, 张小利, 李红星. 观音散治疗小儿厌食症108例临床观察. 中医药导报. 2007(08):37-9.

36. 沙龙. 调中汤治疗脾胃不和型小儿厌食症44例疗效观察. 健康之路. 2014(2):321-.

37. 孙远岭, 顾菊美, 周纬, 范亚可. 运脾方与锌制剂对照治疗儿童厌食症的临床研究. 中医药学刊. 2004;22(12):2206-7.

38. 王丽君. 龙牡壮骨颗粒治疗小儿厌食症（脾胃虚弱证）临床观察 [硕士]: 湖北中医药大学; 2016.

39. 刘智刚, 屈小会. 健脾消食汤治疗小儿厌食的临床观察. 现代中医药. 2014;34(4):36-7.

40. 王光富, 郑建本. 健脾开胃汤治疗小儿厌食症60例疗效观察. 光明中医. 2007(04):43-4.

41. 叶春. 四君子汤加减治疗肝旺脾虚型小儿厌食症24例. 健康之路. 2013;12(11):505.

42. 张红. 醒脾养儿颗粒治疗小儿厌食症的用药效果评估. 人人健康. 2018(22):238.

43. 张青玲. 醒脾养儿颗粒治疗儿童厌食症疗效观察. 中国当代医药. 2009;16(22):175-.

44. 孙宁. 疏肝健脾法治疗减控体重儿童厌食症的临床研究 [硕士]: 浙江中医药大学; 2015.

45. 孙冉. 消食口服液改善小儿厌食症的临床观察. 中国社区医师（医学专业）. 2013;15(8):193-4.

46. 许洪义, 徐曼曼, 邵梦洁, 朱岩, 于秀文. 小儿健脾和胃散治疗小儿厌食症属脾胃虚弱,气郁化热型临床研究. 中国保健营养. 2019;29(11):65-6.

47. 张新友. 小儿健脾丸治疗小儿厌食症的临床疗效评价. 中国继续医学教育. 2015;7(13):196-7.

48. 张文婷, 张雁冰, 陈志勇, 陈昌斌, 赵红梅, 游洁玉. 小儿扶脾颗粒联合硫酸锌治疗小儿厌食症疗效观察. 中国医师杂志. 2015;17(12):1869-71.

49. 张海妹. 小儿喜食颗粒治疗小儿厌食症60例. 社区医学杂志. 2012;10(17):21-2.

50. 向天利, 杨来. 小儿厌食症应用中药补脾益肾法治疗的效果. 临床医药文献电子杂志. 2020;7(A2):48-9.

51. 高汉媛, 王维红, 王继芳, 李小芹, 史正刚. 小儿开胃增食合剂治疗小儿脾胃不和型厌食症疗效观察. 西部中医药. 2018;31(7):52-5.

52. 祝秀珍. 自拟健脾和胃汤治疗小儿脾胃虚弱型厌食症的临床研究. 临床医药文献电子杂志. 2018;5(53):75.

53. 吴红英. 中西医结合治疗小儿厌食症45例临床观察. 中医儿科杂志. 2015;11(06):61-3.

54. 方芳. 中西医结合治疗儿童厌食症300例临床疗效观察. 健康必读（下旬刊）. 2011(12):345-6.

55. 王芳, 王丛礼, 姜丕英, 洪丽君. 中药进食一号颗粒治疗小儿厌食症50例疗效观察. 中国中西医结合儿科学. 2009;1(4):333-4.

56. 赵桂英, 王祥领, 曲延玲. 厌食冲剂的制备与临床观察. 山东医药工业. 2001;20(1):42-3.

57. 韩冬梅. 温胆枳术汤治疗小儿厌食症（脾胃湿热型）临床观察 [硕士]: 黑龙江中医药大学; 2016.

58. 徐铁华. 温脾养阴法治疗小儿厌食症65例. 四川中医. 2002;20(9):57.

59. 陈怡. 调肝醒脾法治疗小儿厌食症120例. 陕西中医. 2006;27(3):268-9.

60. 张晓春. 运脾醒胃汤治疗小儿厌食症疗效观察. 山西中医. 2014;30(3):15.

61. Chen YQ, Fan ZM. Analysis of treatment effect of infantile anorexia with spleen and stomach regulating decoction. China and Foreign Medical Treatment. 2017;36(13):180-2.

62. Zhu Q. The clicinal research on mixture feier for the treatment of child anorexia with the type disorder of the stomach-spleen: Fujian University of Traditional Chinese Medicine; 2009.

63. Zhu Q, Yao W, Xu P. Observation of curative effect of feier mixtures on child anorexia of disharmony between spleen and stomach type. Journal of Shandong University of Traditional Chinese Medicine. 2015;39(2):127-9.

64. Zeng Y, Deng L. Observation on therapeutic effect of Jianpi Xiaoji granules on infantile anorexia. Liaoning Journal of Traditional Chinese Medicine. 2003;30(7):544-5.

65. 刘惠武. 中药治疗小儿厌食症100例. 内蒙古中医药. 2001;20(1):4.

66. 杜恒, 徐和祥. 龙牡壮骨颗粒对厌食症患儿微量元素的影响. 世界中医药. 2016;11(1):91-2.

**2) not for anorexia without organic disease: 2**

1. 杨春. 运脾和胃汤治疗小儿脾胃不和型厌食症疗效. 内蒙古中医药. 2017;36(14):7.

2. Wang J. Therapeutic effect of "Spleen Appetizer" on children with HP Infection-associated anorexia. Systems Medicine. 2018;3(24):21-3.

**3) without diagnostic criteria for anorexia: 68**

1. 张茵. 健脾消积汤治疗小儿厌食症的临床疗效观察. 中国医药指南. 2019;17(24):187-8.

2. 王宏海. 健脾和胃汤治疗小儿厌食疗效观察. 东方药膳. 2020(16):215.

3. 陈利玲. 健脾丸治疗小儿厌食症的临床疗效分析. 光明中医. 2016;31(13):1851-2.

4. 蒋盛花. 健脾丸治疗小儿厌食症的临床研究. 基层医学论坛. 2017;21(31):4385-6.

5. 丛树杰. 健脾养胃颗粒治疗小儿厌食症的效果及安全性分析. 中国社区医师. 2020;36(19):107-8.

6. 李蓉. 健胃消食口服液联合双歧杆菌四联活菌片及赖氨葡锌颗粒治疗厌食症患儿的疗效分析. 黑龙江中医药. 2021;50(01):100-1.

7. 薄其温, 宁宝华, 刘桂兰. 适贝高治疗小儿厌食症126例. 山东中医杂志. 2004;23(11):695-6.

8. 金艳艳. 杞枣口服液治疗厌食症患儿的临床观察. 光明中医. 2018;33(8):1126-8.

9. 邵金玲. 白苓健脾颗粒与锌制剂对照冶疗儿童厌食症的临床分析. 东方食疗与保健. 2015(8):220-.

10. 廉洁. 肥儿疳积颗粒治疗小儿厌食症疗效观察. 中国医药指南. 2009;7(16):92-3.

11. 曾凌云. 肥儿合剂治疗脾胃不和型小儿厌食症的临床研究. 中医临床研究. 2011;3(22):49-50.

12. 李丽嫱, 马惠芳, 王俊怡, 王娜, 孙晓北. 四缝穴治疗小儿厌食症的临床评价. 2005年国际针灸技法及临床应用学术研讨会论文集; 北京2005. p. 173-8.

13. 李欣, 张巍, 代运磊. 醒脾消食汤治疗小儿厌食症临床疗效及不良反应率分析. 药品评价. 2019;16(5):25-7.

14. 李怀祯. 醒脾消食汤治疗小儿厌食症临床疗效观察. 母婴世界. 2019(21):124.

15. 秦钰. 醒脾养儿颗粒在治疗小儿厌食中的应用研究. 中外医学研究. 2013;11(04):39-40.

16. 马春霞. 醒脾养儿颗粒治疗小儿厌食症的临床疗效观察. 中国处方药. 2017;15(11):107-8.

17. 李桂秀. 醒脾养儿颗粒治疗小儿厌食症的临床观察. 临床医药文献电子杂志. 2017;4(82):16137-8.

18. 施金凤, 张富汉. 醒脾养儿颗粒治疗小儿厌食症疗效观察. 首都食品与医药. 2018;25(9):103.

19. 吴小巍. 醒脾养儿颗粒治疗小儿厌食症疗效观察. 健康大视野. 2020(9):109,7.

20. 张薇, 苏公典. 醒脾养儿颗粒治疗小儿厌食疗效观察. 中国医学创新. 2009;6(24):47.

21. 范娟华. 醒脾养儿颗粒治疗儿童厌食症200例. 临床医药文献电子杂志. 2020;7(10):24-5.

22. 周礼双. 醒脾养儿颗粒治疗儿童厌食症的临床分析. 中国保健营养（中旬刊）. 2013(10):520-1.

23. 李明珍. 醒脾养儿颗粒治疗儿童厌食症临床分析. 健康必读（中旬刊）. 2013;12(8):201-.

24. 吴跃南. 醒脾养儿颗粒对厌食症患儿血清胃泌素 、血浆胃动素及神经肽Y水平的影响. 大家健康（上旬版）. 2017;11(12):40-1.

25. 黄晋芬, 冯金娟. 消食醒脾开胃散治疗小儿厌食症83例疗效观察. 中医儿科杂志. 2007(06):26-7.

26. 姜畅. 小儿健脾丸治疗小儿厌食症的临床分析. 内蒙古中医药. 2018;37(04):24-5.

27. 虞志华. 小儿健脾丸治疗小儿厌食症的临床效果观察. 吉林医学. 2011;32(33):7069.

28. 侯崇远. 小儿健脾丸治疗小儿厌食症的临床效果观察. 中国中医药咨讯. 2012;4(5):254.

29. 刘彬. 小儿健脾丸治疗小儿厌食症的临床疗效评价. 大家健康（上旬版）. 2016;10(1):36-7.

30. 张红春. 小儿健胃消食口服液联合葡萄糖酸锌治疗小儿厌食症的疗效分析. 临床医药文献电子杂志. 2016;3(7):1339-,42.

31. 段讲用. 小儿扶脾颗粒联合硫酸锌治疗小儿厌食症的效果与安全性观察. 河南医学研究. 2016;25(08):1475-6.

32. 程亮. 小儿厌食症应用醒脾养儿颗粒的临床治疗效果分析. 当代医学. 2017;23(24):145-7.

33. 陈秋雨. 小儿厌食症应用醒脾养儿颗粒的临床疗效观察. 中国实用医药. 2016;11(15):213-4.

34. 祝咏梅. 逍遥散加减治疗厌食症患儿的效果观察. 中国民康医学. 2019;31(14):118-20.

35. 蔡筱璐. 自拟“开胃饼”治疗小儿厌食临床观察. 广西中医学院学报. 1999;0(1).

36. 许锦华, 王锦平. 自拟健脾益气汤治疗小儿厌食症的临床效果研究. 基层医学论坛. 2020;24(31):4559-60.

37. 舒仕菊. 自拟健脾益气汤治疗小儿厌食症的临床效果评价. 母婴世界. 2020(32):127.

38. 吕勤. 自拟健脾益气汤治疗小儿厌食症疗效及预后观察. 中国实用医药. 2019;14(23):127-8.

39. 闫杰锋. 自拟厌食饼治疗小儿厌食症360例. 中国保健营养（下旬刊）. 2013;23(1):371-2.

40. 李俊梅. 自拟运脾和胃汤治疗小儿厌食症疗效观察. 医学信息. 2014(25):474-.

41. 李云英. 中西医结合治疗小儿脾虚气滞型厌食症的疗效观察. 中西医结合心血管病电子杂志. 2015(13):20-,2.

42. 胡淑德. 中西医结合治疗小儿厌食症40例临床观察. 中医杂志. 1997(01):41-2.

43. 仝伟, 闫奇. 中西医结合治疗小儿厌食症86例. 四川中医. 2003;21(10):79-80.

44. 陈文林. 中西医结合治疗小儿厌食症482例. 现代中医药. 2008;28(2):39-40.

45. 崔桂杰, 曾苓. 中西医结合治疗小儿厌食症的疗效观察. 中西医结合心血管病电子杂志. 2016;4(9):126-7.

46. 张晓, 曹瑾芬, 宋翠领. 中西医结合治疗小儿厌食症的疗效观察. 临床医药文献电子杂志. 2016;3(6):1045,7.

47. 方占荣, 周建辉. 中西医结合治疗小儿厌食症临床疗效观察. 中国中医药咨讯. 2011;3(20):479,354.

48. 李湘辉. 中西医结合治疗小儿厌食症疗效观察. 现代中西医结合杂志. 2012;21(14):1525-6.

49. 吴杰妍. 中西医结合治疗小儿厌食症疗效观察. 吉林中医药. 2008;28(8):587-8.

50. 陈朝晖. 中西医结合治疗小儿厌食症疗效观察. 中外医疗. 2010;29(15):56,8.

51. 郑春玲. 中医辨证治疗小儿厌食症的临床观察. 糖尿病天地. 2018;15(7):38.

52. 常岩. 中医辨证治疗小儿厌食症疗效观察. 临床医药文献电子杂志. 2017;4(24):4696.

53. 余惠平, 张邦道, 周峰, 马新超. 中医药辨治小儿厌食243例. 国际传统医药大会论文摘要汇编; 中国北京2000.

54. 张杰, 燕飞妮. 中医药治疗小儿厌食症研究. 母婴世界. 2021(2):65.

55. 李海枫. 中医辩证治疗小儿厌食症的临床疗效观察. 健康大视野. 2019(11):114.

56. 卢桢婉. 香砂平胃汤加减治疗小儿脾胃虚弱型厌食症的临床研究. 云南中医中药杂志. 2019;40(04):47-9.

57. 李原. 乐食健儿宝治疗厌食症113例. 陕西中医. 2001;22(5):278-9.

58. 者桂莲. 儿童厌食及其临床治疗分析. 吉林医学. 2010;31(9):1182.

59. 李智. 儿童厌食症临床治疗100例观察. 中国卫生产业. 2014(19):179-80.

60. 崔海燕. 参苓白术散加减联合多酶片治疗小儿厌食症临床研究. 亚太传统医药. 2013;9(07):183-4.

61. 李伟峰. 参苓白术散联合多酶片治疗小儿厌食症的临床研究. 河南医学研究. 2015;24(10):92-3.

62. 车颖鸿. 参苓白术散联合多酶片治疗小儿厌食症疗效评价. 医学信息. 2016;29(2):281-2.

63. 郑珊, 雷碧华. 泻黄散治疗小儿厌食症湿热内结型30例临床疗效观察. 中医儿科杂志. 2006;2(2):37-9.

64. 孙凤平, 崔伟锋, 葛国岚, 韩雪. 补中助长颗粒治疗学龄前ISS伴厌食症的疗效观察. 时珍国医国药. 2017;28(5):1151-3.

65. 张云. 观察小儿康颗粒治疗小儿厌食症的临床效果. 中国保健营养. 2016;26(25):144-.

66. 柳春玲, 周黎黎. 运脾颗粒治疗脾虚型小儿厌食症60例临床观察. 甘肃科技. 2015;31(09):124-5.

67. Wang SC, You RD. [Clinical and experimental study on treatment of anorexy in children with the activating spleen prescription]. Zhong Xi Yi Jie He Za Zhi. 1991;11(2):75-8, 67.

68. Jiang Y, Zhang DL. To study the clinical effect of Chinese medicine for invigorating the spleen and kidney in treating infantile anorexia. World Latest Medicine Information. 2018;18(5):139.

**4) not about only HM: 7**

1. 李君君, 肖诏玮, 施志强, 原丹, 沈聪, 马榕花, et al. 福州清明草糕治疗小儿肝旺脾虚型厌食症的临床研究. 医药前沿. 2015;5(27):342-3.

2. 郭倩. 脾胃虚弱型小儿厌食症应用中医保健疗法治疗的有效性. 中国保健营养. 2018;28(19):293.

3. 冯国辉, 杨士珍, 郝海英, 闫翠环. 地锦草合剂足浴治疗小儿厌食60例临床观察. 河北中医药学报. 2012;27(1):21-2.

4. 王利然. 妈咪爱联合三黄屏风膏治疗小儿厌食症的临床研究. 中国中医药科技. 2018;25(5):626-8.

5. 徐仁斌. 贝飞达胶囊联合甘草锌治疗小儿厌食症疗效观察. 海峡药学. 2012;24(2):169-70.

6. 邱静, 王练军, 凤小荣. 复方消化酶胶囊与肥儿合剂对小儿厌食症的疗效及对血清LEP及CCK水平的影响. 现代消化及介入诊疗. 2019;24(1):68-70,3.

7. Pang J. Xingpi Yanger granules combined with domperidone in the treatment of 60 cases of infantile anorexia. China Practical Medicine. 2012;7(18):188-9.

**5) without herb composition: 13**

1. 张强. 捏脊疗法治疗脾胃气虚型小儿厌食症的临床观察 [硕士]: 黑龙江中医药大学; 2013.

2. 王国杰, 张强, 张迪. 捏脊疗法治疗小儿厌食症疗效观察. 中医儿科杂志. 2013;9(01):64-5.

3. 秦莉, 肖向丽. 四磨汤联合双歧杆菌四联活菌片治疗小儿厌食症. 吉林中医药. 2019;39(9):1205-7.

4. 李博, 李维军. 醒脾消食汤治疗小儿厌食症的临床疗效评价. 中国现代药物应用. 2016;10(9):22-3.

5. 万娟. 醒脾养儿颗粒治疗小儿厌食症的临床观察. 医药前沿. 2018;8(13):24-5.

6. 张金玺, 袁国卿. 小儿厌食颗粒对厌食小儿血浆酪神经肽和血清瘦素水平的影响. 时珍国医国药. 2013;24(6):1444-5.

7. 邹敏, 祖敏, 李莉. 中西医结合治疗小儿厌食症的疗效观察. 江西医药. 2014(7):635-6.

8. 李银梅. 中西医结合治疗小儿厌食症疗效观察. 康颐. 2021(1):246.

9. 沙新奂, 马勇. 中西医结合治疗小儿厌食症疗效观察. 中西医结合心血管病电子杂志. 2016;4(19):170,2.

10. 杨中原, 于凤琴, 姜凯. 儿宝颗粒辅助锌硒宝治疗厌食症儿童的效果观察. 实用中西医结合临床. 2018;18(11):42-4.

11. 赵波. 复合凝乳酶治疗儿童厌食和食欲不振的临床疗效观察. 中国现代药物应用. 2012;6(2):63-4.

12. 张粤蓝. 贝飞达胶囊和葡萄糖酸锌联合儿童厌食症的疗效分析. 齐齐哈尔医学院学报. 2013;34(05):715-6.

13. Yang WB, Tang SF, Ma J, Zhang YQ, Shao TW, Xiong C. Linical efficacy of children's compound Jinneijin chewable tablets combined with saccharomyces boulardii powder in the treatment of infantile anorexia. Chinese Health Care. 2021;39(5):3-5.

**6) comparison between HMs: 44**

1. 杨菁华, 杨淑娟, 孙健. 加味异功散治疗小儿厌食症临床疗效观察. 医药前沿. 2018;8(34):355.

2. 陈凤媚, 马书鸽, 张晓莹, 何田田. 肝旺脾虚型小儿厌食的中医治疗体会. 中华中医药学会儿科分会第三十一次学术大会论文汇编; 中国云南昆明2014.

3. 豆玉凤, 史艳平. 健脾消积汤治疗小儿厌食症临床研究. 陕西中医. 2016;37(7):812-3.

4. 芦小慧. 健脾助运方剂治疗小儿厌食症的疗效机制分析. 大家健康（下旬版）. 2016;10(8):43-4.

5. 田洪英, 于田田. 健脾冲剂治疗脾胃虚弱型小儿厌食症的临床研究. 国际中医中药杂志. 2010;32(5):406-8.

6. 范晓妮. 健脾和胃汤治疗脾胃虚弱型小儿厌食症的临床效果分析. 当代医药论丛. 2020;18(17):157-8.

7. 郭萍, 许智芳. 健脾和胃汤治疗小儿脾胃虚弱型厌食症的临床研究. 中国保健营养. 2018;28(35):126.

8. 赵玉敏. 健脾和胃汤对厌食症患儿中医证候及体内微量元素水平的影响. 光明中医. 2017;32(21):3069-71.

9. 钟成梁, 谢杰, 胡思源. 健儿厌食康颗粒治疗小儿厌食脾虚食滞证临床观察. 中国中医药信息杂志. 2006(03):73-4.

10. 罗世惠. 芪斛楂颗粒治疗小儿厌食症疗效观察. 首届中华中医儿科高等教育论坛暨2009年度全国中医儿科学术交流大会论文集; 深圳2009. p. 216.

11. 胡思源, 马融, 刘海沛. 金橘开胃颗粒剂治疗小儿厌食脾失健运证临床研究. 中国中医药信息杂志. 2003;10(7):16-7,78.

12. 张福全. 蒙药查干乌日勒治疗小儿厌食症的临床观察. 中国民族民间医药. 2013;22(7):121-.

13. 赵春玲. 保儿增食液治疗小儿厌食症的临床研究. 广东省五届四次中医儿科学术会议论文集; 广东从化2005. p. 51-4.

14. 李陈, 张凡, 陈淑芬, 谢红, 沈漪萍, 陈秀敏. 三九免煎颗粒治疗脾胃不和型小儿厌食症的临床研究. 湖北中医杂志. 2008;30(7):35-6.

15. 李秀育, editor 三术汤治疗小儿厌食合并HP感染. 第二十三届全国中西医结合消化系统疾病学术会议暨2011消化系统疾病诊治进展学习班; 2011; 中国山西太原.

16. 方英莲, 林爱顺, 李炳植. 醒脾养儿颗粒治疗小儿厌食症临床疗效观察68例. 中国保健营养. 2020;30(33):3,5.

17. 赵霞, 张亚梅, 王亚茹, 惠明刚. 醒脾养儿颗粒治疗小儿厌食疗效观察. 中国中西医结合儿科学. 2010;2(3):277-8.

18. 徐克国. 小健脾汤加减治疗小儿厌食症49例. 中国民间疗法. 2018;26(13):24-5.

19. 蒋梦霞. 消食口服液治疗小儿脾胃不和型厌食症的临床及实验研究 [硕士]: 湖北中医药大学; 2015.

20. 赵爱利. 小儿康颗粒治疗小儿厌食症临床研究. 中外健康文摘. 2012;9(19):452-.

21. 潘玉霞. 小儿厌食颗粒联合布拉氏酵母菌治疗小儿厌食症的临床疗效分析. 东方药膳. 2020(3):36.

22. 陈倩, 马融, 胡思源, 魏小维. 小儿开胃增食颗粒治疗小儿厌食脾失健运证的临床观察. 天津中医药. 2010;27(05):367-8.

23. 赵越郡, 马融, 胡思源, 魏小维, 吕玉霞, 丁樱, et al. 小儿开胃增食颗粒治疗小儿厌食脾失健运证Ⅲ期临床观察. 山西中医. 2011;27(12):12-3+5.

24. 林美萍, 张杰. 小儿肠胃康颗粒治疗小儿厌食症. 河南中医. 2004;24(12):47-.

25. 王静怡. 五谷运脾消疳汤治疗小儿厌食临床观察. 光明中医. 2020;35(22):3508-10.

26. 陈梦麟. 王氏保赤丸治疗小儿厌食症86例. 北京中医. 2000;19(4):62.

27. 王柏婧, 李亚冬. 自拟"万青汤"合针挑四缝穴治疗小儿厌食症50例. 中国民族民间医药杂志. 2001(1):17-8.

28. 廖世忠. 自拟健脾和胃汤治疗小儿脾胃虚弱型厌食症的临床疗效及对体内微量元素水平的影响. 内蒙古中医药. 2017;36(18):16-7.

29. 李景新. 自拟健脾和胃汤治疗小儿脾胃虚弱型厌食症的临床疗效对体内微量元素水平的影响分析. 家有孕宝. 2020;2(17):59.

30. 乔威. 自拟健胃消食汤治疗小儿厌食症126例临床观察. 当代医学. 2016;22(17):153-,4.

31. 韩谨. 自拟醒脾和胃汤治疗小儿厌食症42例临床观察. 四川中医. 2002;20(9):55-6.

32. 庄海燕. 自拟养阴食疗方治疗小儿脾胃阴虚型厌食的临床疗效观察 [硕士]: 福建中医药大学; 2017.

33. 林向韶, 叶剑, 丁吴女. 中西医结合治疗小儿厌食症60例. 浙江中医杂志. 2012;47(11):806.

34. 纪明春. 中西医结合治疗小儿厌食疗效观察. 内蒙古中医药. 2012;31(14):25-6.

35. 张立秋, 李恒, 宋长艳, 李童波. 乐食冲剂治疗小儿厌食60例临床观察. 第24届全国中医儿科学术研讨会、中医药高等教育儿科教学研讨会、儿科名中医讲习班论文汇编; 中国福建厦门2007.

36. 刘丽平. 厌食合剂治疗小儿厌食症30例疗效观察. 中国中西医结合儿科学. 2013;5(3):245-6.

37. 张华静, 胡思源. 宝贝开胃颗粒剂治疗脾失健运型小儿厌食121例临床研究. 实用中医内科杂志. 2007(08):56-7.

38. 孙秋玲. 开胃进食汤超微颗粒治疗小儿厌食症的临床疗效. 海峡药学. 2018;30(12):191-2.

39. 曹建雄. 开胃进食汤超微颗粒治疗小儿厌食症临床疗效及其抗厌食作用之研究 [博士]: 湖南中医药大学(湖南省中医药研究院); 2007.

40. 张文静. 拟健脾和胃汤治疗小儿脾胃虚弱型厌食疗效观察. 健康前沿. 2019;28(2):228.

41. 孙晓虹, 田洪英, 孙聪玲, 吴金勇. 补脾益肾法治疗小儿厌食症临床研究. 中国实用医药. 2010;5(35):116-7.

42. 王海涛. 运脾利胆方和西药在脾胃不和型厌食中的疗效对比观察. 中西医结合研究. 2016;8(1):46-,56.

43. 黄冬梅. 运脾平肝法治疗小儿厌食脾虚肝旺证的临床研究 [硕士]: 南京中医药大学; 2014.

44. Wang Y, Zhong DD, Ji XH, Cheng YB, Gao XD, Zheng YM, et al. Multi-center randomized double-blind controlled study on children's anorexia (spleen-stomach disharmony) treated with Child Compound Endothelium Corneum. Zhongguo Zhongyao Zazhi. 2021;46(9):2298-303.

**7) using other TCM intervention: 9**

1. 陈英芳, 耿少怡, 孙桂芳, 王海燕. 健儿增食汤联合推拿治疗小儿厌食症80例临床观察. 河北中医. 2005(09):668.

2. 陈淑芬. 杞枣口服液联合推拿治疗脾胃虚弱型小儿厌食症60例. 中国药业. 2015(22):179-80.

3. 杜洪煊. “培土调枢”推拿法治疗小儿厌食症的临床研究 [硕士]: 广州中医药大学; 2018.

4. 吴青春. 中西医结合治疗小儿厌食症30例. 中国中医药现代远程教育. 2015;13(08):38-9.

5. 程春颖. 中西医结合治疗小儿厌食症40例疗效观察. 湖南中医杂志. 2016;32(5):73-4.

6. 郭芳. 中医辨证疗法配合经皮给药治疗小儿厌食症的效果观察. 医药前沿. 2017;7(12):340-1.

7. 张艳. 中医保健疗法治疗脾胃虚弱型厌食症患儿的疗效观察. 母婴世界. 2020(3):101.

8. 闫保瑞. 中医保健疗法治疗脾胃虚弱型厌食症患儿的疗效观察. 中国民康医学. 2018;30(22):77-8.

9. 李惠. 探讨中医保健疗法治疗脾胃虚弱型小儿厌食症的疗效. 中华养生保健. 2020;38(4):29-31.

**8) only abstract without raw data: 6**

1. 豆玉凤, 史艳平, editors. 健脾消积汤治疗小儿厌食症临床观察. 第十一届全国中西医结合基础理论学术研讨会; 2015; 中国宁夏回族自治区银川.

2. 史艳平, 豆玉凤. 健脾消积汤治疗小儿厌食症临床观察. 庆祝中国当代儿科杂志创刊15周年大会暨当代儿科论坛论文集; 长沙2013. p. 315-.

3. 俞虹, editor 扶土抑木汤治小儿用脾虚厌食症66例疗效分析. 中西医结合第九次全国儿科学术会议; 2000; 中国广西北海.

4. 方芳, 罗振芳, 肖芳, 黄志娟. 中西医结合治疗小儿厌食症120例临床疗效观察. 第二届国际妇幼保健学术会议暨2006全国妇幼保健学术大会论文集; 中国北京2006.

5. 张金虎, 支晓艳. 厌食汤治疗小儿厌食213例. 中华中医药学会儿科分会第三十次学术大会论文汇编; 中国山东济南2013.

6. Wang Y. Brief introduction to clinical treatment and observation of 100 cases of infantile anorexia. China Health Vision. 2013;21(5):408.

**9) duplicate data: 1**

1. Lin LM, Liu JZ, Xiang XX. Chinical research on Xiaoer Piweile in treating children anorexia of spleen-dysfunction in fransportation. Hubei Journal of Traditional Chinese Medicine. 2013;35(11):9-10.
