# Supplementary material for: Herbal Medicine for the Treatment of Anorexia in Children: A Systematic Review and Meta-Analysis
Source: Front Pharmacol. 2022 Apr 1;13:839668. doi: 10.3389/fphar.2022.839668 (PMC9012502; doi:10.3389/fphar.2022.839668)
Supplement: Supplementary file 3 [file Table5.DOCX]

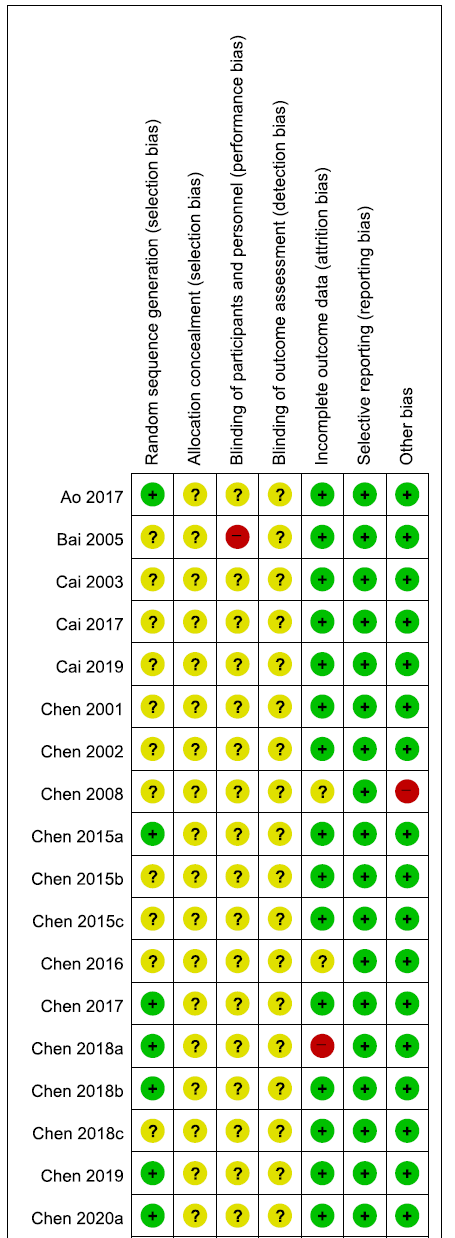

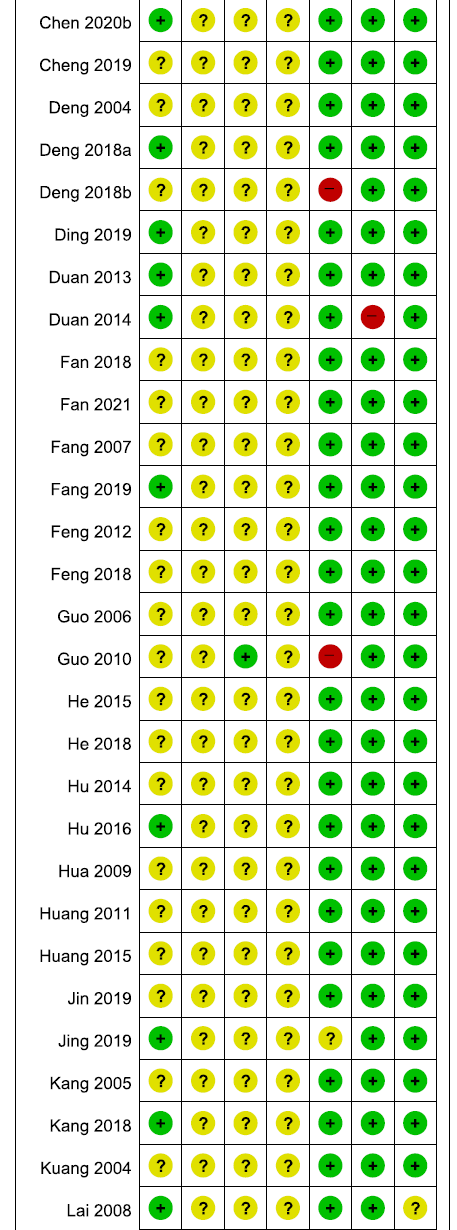


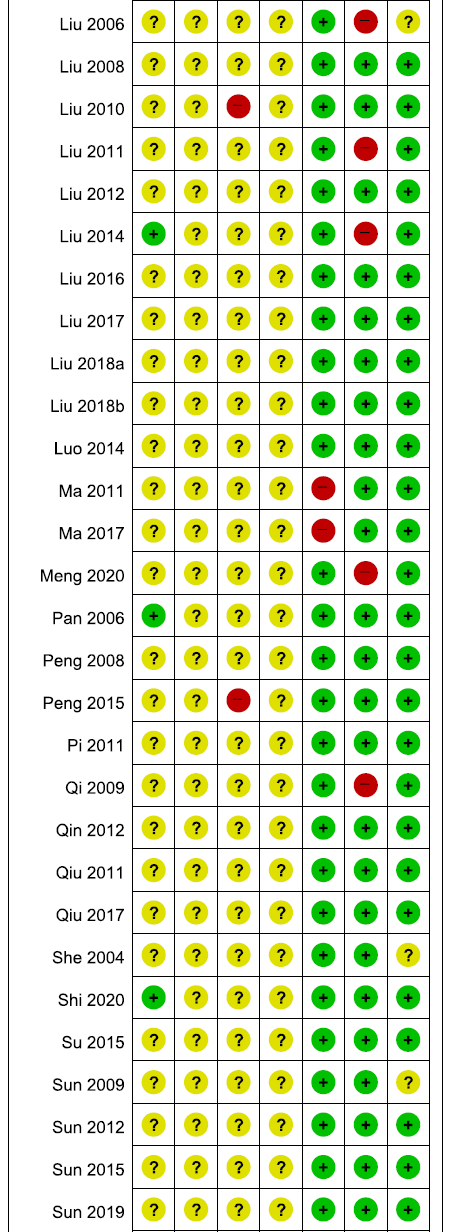

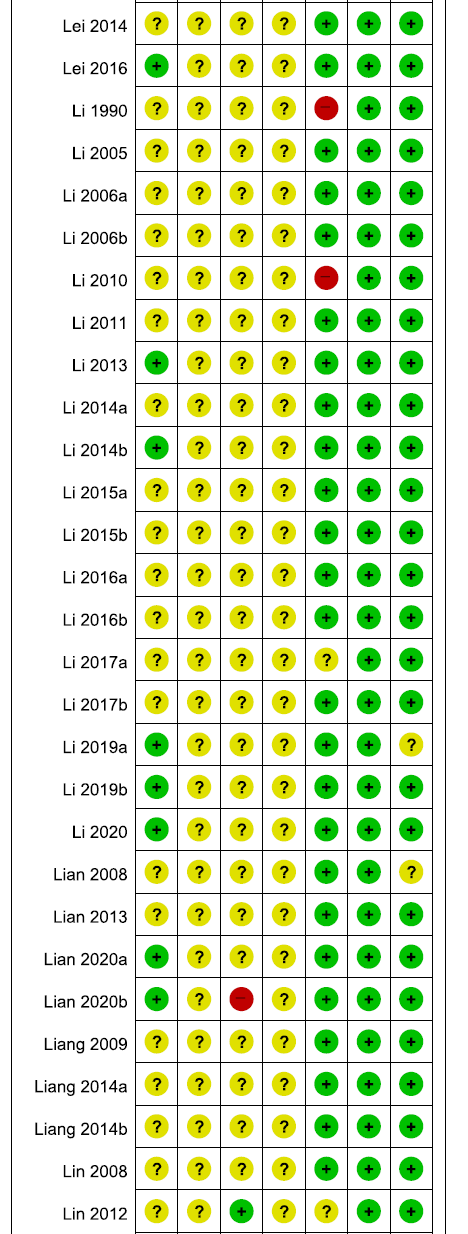


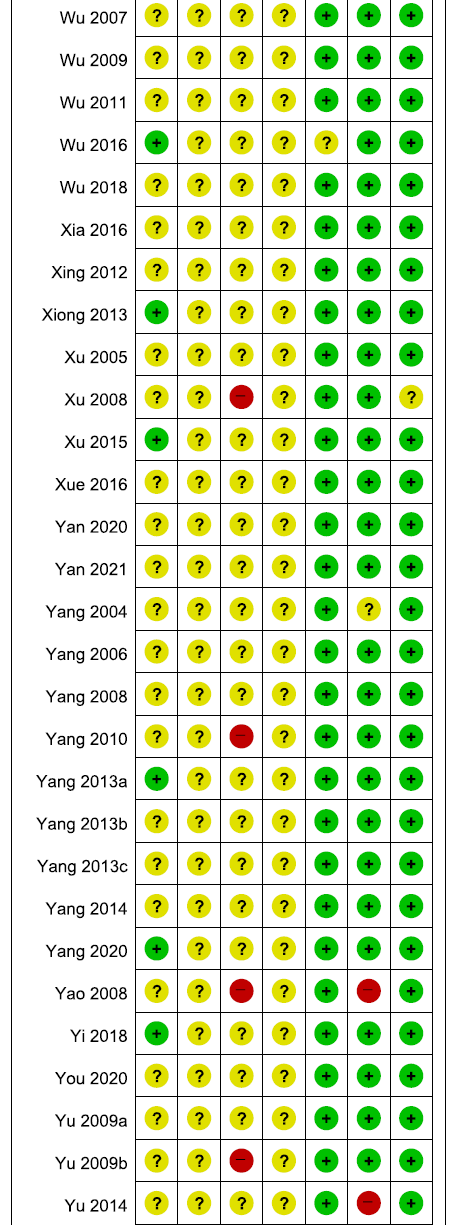

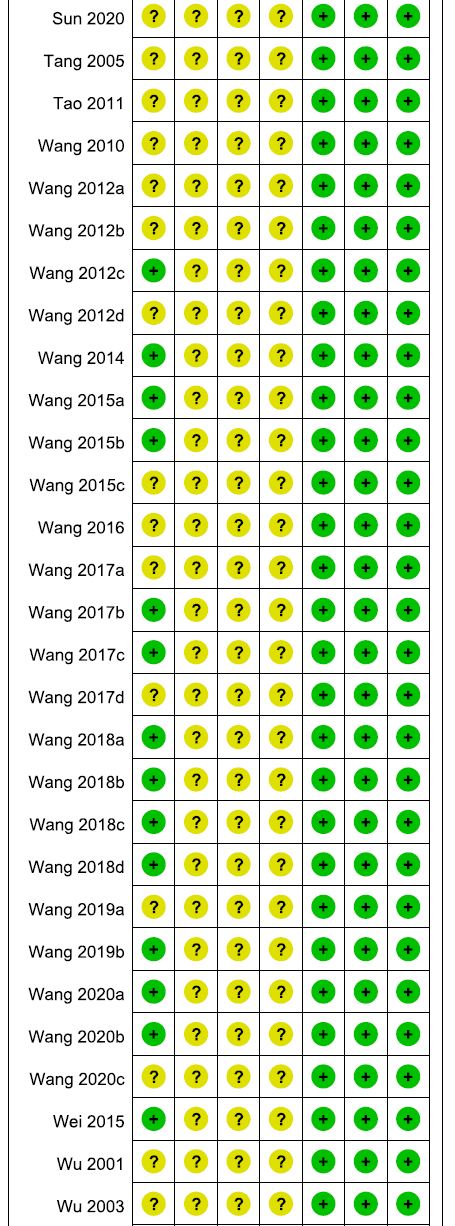


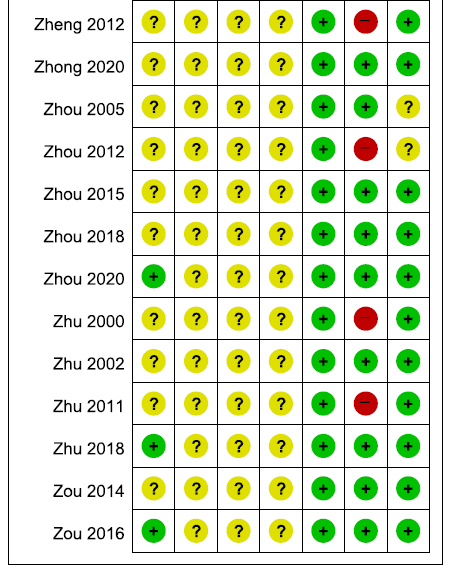

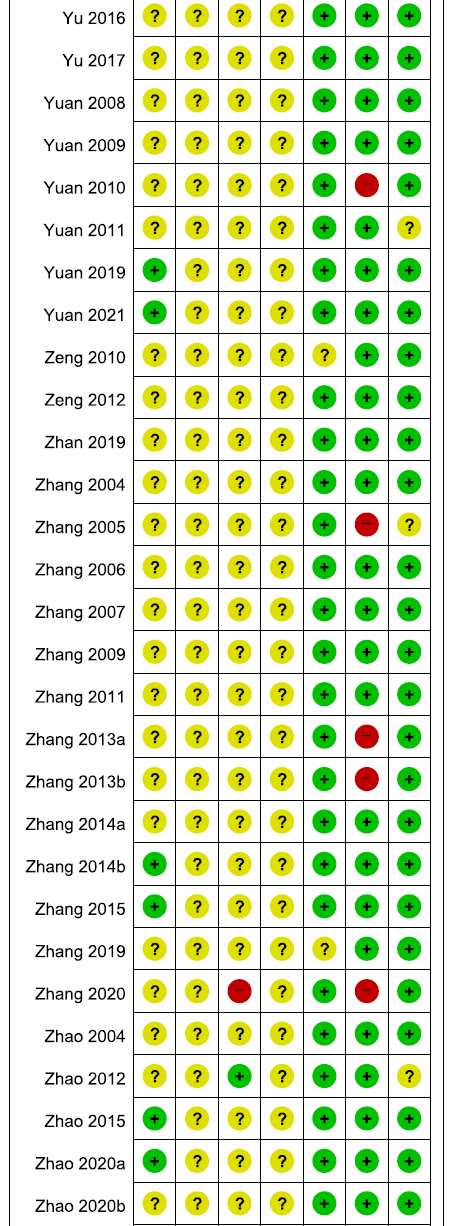


**Supplement 5.** Risk of bias summary for all included studies.

Note. Low, unclear, and high risk, respectively, are represented with the following symbols: “+”, “?”, and “-”.
