# Supplementary material for: Herbal Medicine for the Treatment of Anorexia in Children: A Systematic Review and Meta-Analysis
Source: Front Pharmacol. 2022 Apr 1;13:839668. doi: 10.3389/fphar.2022.839668 (PMC9012502; doi:10.3389/fphar.2022.839668)
Supplement: Supplementary file 4 [file Table3.DOCX]

Supplement 3. General clinical characteristics of the included studies

| **Study ID** | **Sample size (TG:CG)** | **Mean age or range (yr)** | **Anorexia disease period** | **Diagnostic criteria** | **Pattern identification** | **TG** | **CG** | **Outcome** | **Adverse events** |
| --- | --- | --- | --- | --- | --- | --- | --- | --- | --- |
| Ao 2017 | 100(50:50) | TG: 4.25 ± 1.23 CG: 4.56 ± 1.54 | NR | 《Pediatrics》 | spleen failing in transportation | HM + CG | Zinc Gluconate | 1. TER 2. Weight change (kg) 3. Feeding situation (mL/d) 4. Sleep condition (hour/d) 5. intermittent abdominal pain (cases) | NR |
| Bai 2005 | 240(120:120) | 2~14 | 3mo~3yr | 《Practical Pediatrics》  《Pediatrics of Chinese Medicine》 | spleen deficiency food accumulation | HM + Zinc Gluconate | Multi-enzyme + Domperidone  + Zinc Gluconate | 1. TER 2. Treatment period | NR |
| Cai 2003 | 108(66:42) | 1~7 | ≧1mo | 《Criteria for Diagnosis and Efficacy of TCM Diseases and Syndromes》 | spleen deficiency | HM | Multi-enzyme + Zinc Citrate | 1. TER | TG: None CG: NR |
| Cai 2017 | 100(50:50) | TG: 6.07 ± 2.15 CG: 6.12 ± 2.04 | TG: 6.51 ± 1.35wk CG: 6.58 ± 1.31wk | 《Practical Pediatrics》 | NR | HM + CG | Probiotics  (Quadruple Bifidobacterium Live Bacteria)  + Zinc Gluconate | 1. TER 2. TER (TCM symptom score) 3. Recurrence rate | N.S TG: constipation 2, vomiting 1 CG: constipation 1, nausea 1, vomiting 2 |
| Cai 2019 | 59(30:29) | TG: 4.07 ± 2.80 CG: 4.04 ± 2.45 | TG: 2.53 ± 1.62mo CG: 2.84 ± 1.51mo | 《Zhu Futang Practical Pediatrics》  《Criteria for Diagnosis and Efficacy of TCM Diseases and Syndromes》 | NR | HM + CG | Five Vitamins  + Lysine | 1. TER 2. Serum Zn (μmol/L) 3. Serum Fe (μmol/L) 4. Hemoglobin (g/L) 5. TCM symptom score | None |
| Chen 2001 | 74(38:36) | TG: 3.6 CG: 3.7 | NR | 《Criteria for Diagnosis and Efficacy of TCM Diseases and Syndromes》 | spleen deficiency | HM | Pepsin mixture  + Zinc Gluconate | 1. TER | NR |
| Chen 2002 | 300(162:138) | TG: 4.2 CG: 4.6 | 2wk~6mo | 《Practical Pediatrics》 | NR | HM | Vitamin B complex  + Zinc Gluconate | 1. TER 2. Symptom score | NR |
| Chen 2008 | 62(30:32) | TG: 6.53 ± 0.78 CG: 6.18 ± 0.45 | TG: 38 ± 13.24mo CG: 36 ± 11.21mo | 《Pediatrics of Chinese Medicine》 | NR | HM | Domperidone | 1. TER 2. Gastric half emptying time (min) 3. Gastric antrum contraction frequency (times/hour) | NR |
| Chen 2015a | 110(58:52) | TG: 5.58 ± 2.13 CG: 5.44 ± 2.53 | TG: 5.44 ± 1.97mo CG: 6.1 ± 1.98mo | 《Diagnostic Criteria of Anorexia in Children》 | spleen-stomach qi deficiency | HM | Pepsin mixture | 1. TER 2. Serum gastrin (ng/L) 3. Plasma motilin (ng/L) 4. NPY (ng/L) | None |
| Chen 2015b | 60(30:30) | TG: 1~6 CG: 1~6 | NR | 《Criteria for Diagnosis and Efficacy of TCM Diseases and Syndromes》 | spleen-stomach qi deficiency | HM | Zinc Gluconate | 1. TER 2. Weight (kg) 3. Height (cm) 4. Serum Zn (μmol/L) | NR |
| Chen 2015c | 60(30:30) | TG: 4.34 ± 2.25  CG: 4.564 ± 2.27 | TG: 6.89 ± 2.04mo  CG: 6.45 ± 2.27mo | 《Guiding Principles for Clinical Research of New Chinese Medicines》 | NR | HM + CG | Multi-enzyme + Pepsin  + Domperidone + Zinc Gluconate | 1. TER 2. Serum Zn (μmol/L) 3. Salivary Amylase (U/L) 4. Urine D-xylose excretion (μmol/L) | None |
| Chen 2016 | 156(78:78) | 4.5 | 3mo~5yr | 《Diagnosis and Curative Effect Criteria of Pediatric Diseases and Syndromes of Traditional Chinese Medicine》 | spleen failing in transportation, spleen-stomach qi deficiency, stomach yin deficiency | HM + CG | Zinc Sulfate | 1. TER | NR |
| Chen 2017 | 120(60:60) | TG: 8.54 ± 0.52  CG: 8.38 ± 0.49 | TG: 5.72 ± 1.28mo CG: 5.32 ± 1.37mo | 《Criteria for Diagnosis and Curative Effect of Traditional Chinese Medicine Symptoms》 《Guiding Principles for Clinical Research of New Chinese Medicines》 《Practical Pediatrics》 | spleen-stomach qi deficiency | HM + CG | Pepsin mixture | 1. TER 2. Serum gastrin (ng/L) 3. Serum motilin (ng/L) 4. Serum NPY (ng/L) 5. Serum Zn (μmol/L) 6. Selenium (mg/ml) 7. TCM symptom score | NR |
| Chen 2018a | 64(32:32)→ 60(30:30) | TG: 5 CG: 5 | TG: 3  CG: 2 (no unit) | 《Practical Pediatrics》 《Criteria for Diagnosis and Efficacy of TCM Diseases and Syndromes》 | spleen-stomach qi deficiency | HM + CG | Multi-enzyme | 1. TER 2. TCM symptom score | None |
| Chen 2018b | 100(50:50) | TG: 4.36 ± 0.66 CG: 4.35 ± 0.65 | TG: 6.18 ± 0.62mo CG: 6.26 ± 0.50mo | 《Practical Pediatrics》 《Guiding Principles for Clinical Research of New Chinese Medicines》 | qi deficiency, yin deficiency, yang deficiency, phlegm-dampness | HM + CG | Zinc Gluconate | 1. TER 2. Total score of anorexia 3. Main symptom score (loss of appetite, decreased food intake) 4. Secondary symptom score 5. Urine D-xylose excretion rate  6. Recurrence rate 7. PedsQL 4.0 | NR |
| Chen 2018c | 90(45:45) | TG: 4.08 ± 1.39 CG: 4.17 ± 1.26 | TG: 66.91 ± 0.70d CG: 67.10 ± 0.30d | 《Practical Pediatrics》 《Guiding Principles for Clinical Research of New Chinese Medicines》 | spleen-stomach qi deficiency,  spleen-stomach yin deficiency | HM + CG | Domperidone  + Vitamin | 1. TER 2. Serum Zn (μmol/L) 3. Serum Fe (mmol/L) 4. Serum Ca (mmol/L) | NR |
| Chen 2019 | 80(40:40) | TG: 6.46 ± 2.60 CG: 6.28 ± 2.68 | TG: 7.94 ± 2.68mo CG: 7.64 ± 2.42mo | 《Criteria for Diagnosis and Curative Effect of TCM Diseases》 | liver-spleen disharmony | HM + CG | Zinc Gluconate  + Domperidone  + Multi-vitamin | 1. Serum Zn (g/L) 2. Weight (kg) 3. TER | TG: nausea 1, vomiting 1, diarrhea 1 CG: nausea 1, vomiting 1, diarrhea 2 |
| Chen 2020a | 102(51:51) | TG: 5.8 ± 1.1 CG: 6.1 ± 1.2 | TG: 8.5 ± 1.6mo CG: 8.9 ± 1.7mo | 《Zhu Futang Practical Pediatrics》 | NR | HM + CG | Probiotics (Bifidobacterium Triple Live Bacteria) | 1. TER 2. Weight (kg) 3. Hemoglobin (g/L) 4. Main symptom score (loss of appetite) | NR |
| Chen 2020b | 80(40:40) | TG: 7.2 ± 3.2 CG: 7.6 ± 3.2 | TG: 8.13 ± 4.62mo CG: 8.27 ± 4.83mo | 《Zhu Futang Practical Pediatrics》 《Guidelines for Diagnosis and Treatment of Common Diseases in Pediatrics of Traditional Chinese Medicine》 | spleen deficiency with effulgent liver | HM + CG | Probiotics (Bifidobacterium Lactobacillus Triple Live Bacteria) | 1. TER 2. Weight gain (kg) (after 6mo) 3. Serum Zn (mg/L) 4. Serum Fe (mg/L) 5. TCM symptom score 6. Main symptom score (loss of appetite) 7. Serum Mg (mg/L) 8. Serum Cu (mg/L) 9. IgM (g/L) 10. IgG (g/L) 11. IgA (g/L) 12. Number of respiratory infections (after 6mo) 13. Number of gastrointestinal infections (after 6mo) | NR |
| Cheng 2019 | 86(43:43) | TG: 5.12 ± 0.53 CG: 5.24 ± 0.61 | TG: 14.56 ± 2.53d CG: 14.12 ± 2.41d | 《Zhu Futang Practical Pediatrics》 | NR | HM  + Ca + Zinc Gluconate | Probiotics  (Bifidobacterium Tetravaccine) + Ca  + Zinc Gluconate | 1. TER | NR |
| Deng 2004 | 150(80:70) | TG: 2.8 ± 1.03 CG: 3.5 ± 1.01 | TG: 1.08 ± 0.33yr CG: 1.01 ± 0.31yr | 《Criteria for Diagnosis and Curative Effect of TCM Diseases》 《Practical Pediatrics》 | spleen failing in transportation | HM | Pepsin mixture + Yeast | 1. TER | NR |
| Deng 2018a | 60(30:30) | TG: 5.4 ± 3.4 CG: 5.9 ± 3.3 | TG: 14.5 ± 19.5mo CG: 14.7 ± 18.8mo | 《Zhu Futang Practical Pediatrics》 《Technical Guidelines for Clinical Trial Design and Evaluation of New Chinese Medicines for Anorexia in Children》 | spleen deficiency with effulgent liver | HM | Probiotics (Bacillus Subtilis and Enterococcus Faecium Live Bacteria) | 1. TER 2. Main symptom score (loss of appetite, decreased food intake) | None |
| Deng 2018b | 64(32:32)→ 60(30:30) | TG: 1~14 CG: 1~14 | TG: <1yr (8 cases), 1~2yr (9 cases), ≧2yr (13 cases) CG: <1yr (8 cases), 1~2yr (9 cases), ≧2yr (13 cases) | 《Zhu Futang Practical Pediatrics》 《Technical Guidelines for Clinical Trial Design and Evaluation of New Chinese Medicines for Anorexia in Children》 | spleen deficiency with effulgent liver | HM | Probiotics (Bacillus Subtilis and Enterococcus Faecium Live Bacteria) | 1. TER 2. Leptin 3. Ghrelin 4. Serum Zn 5. Serum Fe 6. Serum Ca 7. TER (TCM symptom score) 8. Recurrence rate 9. Main symptom score (loss of appetite, decreased food intake) 10. Secondary symptom score (color of face, stool) | NR |
| Ding 2019 | 80(40:40) | TG: 6.2 ± 0.6 CG: 6.4 ± 0.7 | TG: 6.2 ± 0.4mo CG: 6.1 ± 0.5mo | 《Standards for Diagnosis and Treatment of Infantile Anorexia》 | NR | HM | Zinc Gluconate | 1. TER 2. Serum Leptin (ng/L) 3. Plasma NPY (ng/L) | NR |
| Duan 2013 | 60(30:30) | TG: 8.25 (3-13) CG: 8.25 (3-13) | TG: 1~12mo CG: 1~12mo | 《Zhu Futang Practical Pediatrics》 | NR | HM | Domperidone | 1. TER 2. Urine D-xylose excretion rate (%) | NR |
| Duan 2014 | 60(30:30) | TG: 8.25 CG: 8.25 | >2mo | 《Criteria for Diagnosis and Efficacy of TCM Diseases and Syndromes》 《Pediatrics of Integrated Traditional Chinese and Western Medicine》 《Zhu Futang Practical Pediatrics》 《Guiding Principles for Clinical Research of New Chinese Medicines》 | spleen-stomach disharmony | HM | Domperidone | 1. TER 2. Serum Zn (μmol/L) 3. Urine D-xylose excretion | None |
| Fan 2018 | 300(150:150) | TG: 3.1 ± 1.5 CG: 2.9 ± 1.7 | TG: 12.3 ± 3.4mo CG: 11.7 ± 2.8mo | 《Guiding Principles for Clinical Research of New Chinese Medicines》 | spleen deficiency with effulgent liver | HM + CG | Zinc Sulfate + Lactobacillus | 1. TER 2. Leptin (ng/ml) 3. Human growth hormone releasing peptide (ng/ml) 4. TCM symptom score | NR |
| Fan 2021 | 120(40:40:40) | TG1: 3.60 ± 1.22 TG2: 3.65 ± 1.10 CG: 3.83 ± 1.45 | TG1: 8.63 ± 3.21wk TG2: 8.85 ± 3.30wk CG: 8.70 ± 3.32wk | 《Zhu Futang Practical Pediatrics》 《Pediatrics of Chinese Medicine》 | NR | TG1: HM TG2: TG1 + Acupoint herbal patching | Probiotics  (Bacillus Subtilis and Enterococcus Double Live Bacteria) | 1. TER 2. Weight gain (kg) 3. Symptom and sign score | None |
| Fang 2007 | 67(35:32) | TG: 5.6 CG: 4.8 | TG: 7mo CG: 6mo | 《TCM Disease Diagnosis and Treatment Standard》 《Standards for Diagnosis and Treatment of Infantile Anorexia》 | NR | HM | Zinc Gluconate | 1. TER 2. Hair Zn (μg/g) 3. Hair Fe (μg/g) 4. Hair Cu (μg/g) 5. Hair Ca (μg/g) | TG: None CG: NR |
| Fang 2019 | 240(80:80:80) | TG1: 4.78 ± 1.43 TG2: 4.38 ± 1.65 CG: 4.18 ± 1.87 | TG1: 7.32 ± 3.13mo TG2: 7.51 ± 3.66mo CG: 7.91 ± 3.46mo | 《Wolfson Diagnostic Criteria》 《Criteria for Diagnosis and Efficacy of TCM Diseases and Syndromes》 | spleen-stomach qi deficiency | TG1: HM + CG TG2: HM | Probiotics (Saccharomyces Boulardii) | 1. TER 2. Weight (kg) 3. Height (cm) 4. Leptin (ng/L) 5. Ghrelin (ng/L) 6. Serum substance P (ng/L) 7. Total improvement rate | None |
| Feng 2012 | 86(43:43) | TG: 4.1 ± 1.26 CG: 4.2 ± 1.23 | NR | 《Diagnosis and Curative Effect Criteria of Pediatric Diseases and Syndromes of Traditional Chinese Medicine》 | spleen failing in transportation with spleen qi depletion, spleen failing in transportation with stomach yin deficiency | HM + CG | Multi-enzyme | 1. TER 2. Improvement rate of main symptoms 2-1. anorexia 2-2. constipation 2-3. intermittent abdominal pain 2-4. weight gain 3. TCM symptom score | None |
| Feng 2018 | 60(30:30) | TG: 3.2 CG: 3.6 | TG: 7.2mo CG: 5.3mo | 《Criteria for Diagnosis and Efficacy of TCM Diseases and Syndromes》 《Practical Pediatrics》 | spleen-stomach qi deficiency | HM | Zinc Gluconate | 1. TER 2. Weight (kg) 3. Height (cm) 4. Zn (μmol/L) | NR |
| Guo 2006 | 180(100:80) | TG: 4 CG: 4.3 | TG: 1.2yr CG: 1.3yr | 《Pediatrics of Chinese Medicine》 | spleen-stomach disharmony (TG1), spleen-stomach qi deficiency (TG2), spleen-stomach dural deficiency of qi and yin (TG3) | TG1: HM TG2: HM + Beiqi Injection TG3: HM + Shengmai injection | Zinc Gluconate | 1. TER | NR |
| Guo 2010 | 72(36:36)→ 63(30:33) | TG: 4.332 ± 1.715 CG: 3.806 ± 1.783 | TG: 33.667 ± 16.629 CG: 26.194 ± 18.769 (no unit) | 《Practical Pediatrics》 《Pediatrics of Chinese Medicine》 《Guiding Principles for Clinical Research of New Chinese Medicines》 《Criteria for Diagnosis and Efficacy of TCM Diseases and Syndromes》 | spleen failing in transportation | HM | Placebo | 1. TER 2. Effective rate of main symptoms (appetite, food intake) | None |
| He 2015 | 74(37:37) | TG: 6.53 ± 0.82 CG: 6.37 ± 0.75 | TG: 36.05 ± 12.76mo CG: 34.02 ± 13.50mo | 《Zhu Futang Practical Pediatrics》 《Pediatrics of Chinese Medicine》 | spleen-stomach qi deficiency | HM + CG | Domperidone | 1. TER 2. BMI 3. TCM symptom score 4. Food intake (g) | NR |
| He 2018 | 86(43:43) | TG: 4.5 ± 0.6 CG: 4.4 ± 0.8 | TG: 3.6 ± 1.1mo CG: 3.5 ± 1.0mo | 《Practical Pediatrics》 《Pediatrics of Chinese Medicine》 | NR | HM | Zinc Gluconate | 1. TER | NR |
| Hu 2014 | 81(42:39) | TG: 4.2 ± 1.8 CG: 3.8 ± 1.4 | TG: 7.2 ± 1.2mo CG: 7.8 ± 1.0mo | 《Research Progress of Anorexia in Children》 《Criteria for Diagnosis and Efficacy of TCM Diseases and Syndromes》 | spleen deficiency with accumulation | HM | Domperidone | 1. TER 2. Ghrelin (ng/L) 3. Leptin (ng/L) 4. Weight (kg) 4-1. Weight 3mo after treatment 4-2. Weight 6mo after treatment | None |
| Hu 2016 | 130(65:65) | TG: 5.8 ± 1.9 CG: 5.4 ± 2.1 | TG: 1.9 ± 0.5yr CG: 1.7 ± 0.3yr | 《Zhu Futang Practical Pediatrics》 《Pediatrics of Chinese Medicine》 | spleen-stomach qi deficiency | HM  + Ca + Zinc Gluconate | Probiotics (Bifidobacterium Tetravaccine) + Ca  + Zinc Gluconate | 1. TER 2. Weight gain (kg) 3. Serum gastrin (ng/L) 4. Plasma motilin (ng/L) 5. Plasma NPY (ng/L) 6. Increment of hemoglobin (g/L) 7. Increment of total protein (g/L) | NR |
| Hua 2009 | 89(52:37) | 5.74 | 6.23mo | 《Guiding Principles for Clinical Research of New Chinese Medicines》 | NR | HM | 5 types of Vitamin  + Multi-enzyme | 1. TER 2. Urine D-xylose excretion rate | NR |
| Huang 2011 | 124(62:62) | TG: 6.1 CG: 6.5 | TG: 6.5mo CG: 6.1mo | 《Criteria for Diagnosis and Efficacy of TCM Diseases and Syndromes》 | NR | HM | Zinc Gluconate  + Vitamin B complex | 1. TER | TG: None CG: NR |
| Huang 2015 | 50(25:25) | 6mo~14yr | NR | 《Zhu Futang Practical Pediatrics》 | NR | HM + CG | Zinc Gluconate | 1. TER | None |
| Jin 2019 | 74(37:37) | TG: 3.98 ± 1.09 CG: 3.51 ± 1.37 | TG: 3.43 ± 1.21mo CG: 4.01 ± 1.32mo | 《Zhu Futang Practical Pediatrics》 | NR | HM + CG | Probiotics (Bacillus Subtilis Dual Live Bacteria) | 1. TER 2. Weight (kg) 3. Appetite recovery time (d) 4. Abdominal pain and distension disappear time (d) | None |
| Jing 2019 | 244(166:78)→ 243(165:78) | TG: 5.49 ± 3.18  CG: 5.19 ± 3.12 | TG: 79.48 ± 10.62d  CG: 69.64 ± 8.26d | 《Zhu Futang Practical Pediatrics》 《Guidelines for Diagnosis and Treatment of Common Diseases in Pediatrics of Traditional Chinese Medicine》 | spleen-stomach qi deficiency | HM | Pepsin mixture | 1. TER 2. Main symptom score (food, spirit, body) 3. Secondary symptom score (color of face, lips, and nails, abdominal symptom, vomiting, stool, urine, color of tongue and pulse) | None |
| Kang 2005 | 256(156:100) | TG: 4.5 CG: 4.7 | TG: 2.7yr CG: 2.8yr | 《Criteria for Diagnosis and Curative Effect of TCM Diseases》 | inward retain of worm toxin with effulgent heart-liver fire, spleen deficiency with stomach heat | HM | Multi-enzyme | 1. TER | NR |
| Kang 2018 | 92(46:46) | TG: 3.41 ± 0.92 CG: 3.26 ± 0.90 | TG: 7.05 ± 2.04mo CG: 7.18 ± 2.06mo | 《Guiding Principles for Clinical Research of New Chinese Medicines》 《Diagnosis and treatment of infantile anorexia》 | spleen-stomach weakness | HM + CG | Probiotics  (Bifidobacterium Tetravaccine) + Iron-containing preparations + Vitamin A | 1. TER 2. Ghrelin (pg/ml) 3. Leptin (pg/ml) 4. NPY (pg/ml) | None |
| Kuang 2004 | 108(66:42) | 1~7 | ≧1mo | 《Criteria for Diagnosis and Efficacy of TCM Diseases and Syndromes》 | spleen deficiency | HM | Multi-enzyme + Multi-vitamin | 1. TER | TG: None CG: NR |
| Lai 2008 | 79(40:39) | 3.84 ± 1.15 | 4.93 ± 2.55mo | 《Criteria for Diagnosis and Curative Effect of TCM Diseases》 | NR | HM | Medilac-Vita®  (Combined Bacillus Subtilis and Enterococcus Faecium Granules with Multi-vitamines) | 1. TER 2. Weight (kg) 3. Hemoglobin (g/L) 4. RBC (10^12^/L) 5. TCM symptom score | NR |
| Lei 2014 | 150(75:75) | TG: 5.8 ± 1.4 CG: 5.9 ± 1.7 | TG: 4.1 ± 0.6mo CG: 4.3 ± 0.8mo | 《Criteria for Diagnosis and Curative Effect of TCM Diseases》 | middle deficiency and stagnation | HM + CG | Lysine  + Inositol  + Vitamin B12 | 1. TER 2. TER (TCM symptom score) | NR |
| Lei 2016 | 60(30:30) | TG: 7.7 ± 2.5 CG: 8.1 ± 3.0 | NR | 《Guiding Principles for Clinical Research of New Chinese Medicines》 | NR | HM | Medilac-Vita®  (Combined Bacillus Subtilis and Enterococcus Faecium Granules with Multi-vitamines) | 1. TER 2. Serum Leptin (ng/ml) 3. Serum Orexin (ng/ml) 4. Main symptom score | NR |
| Li 1990 | 101(58:43) | 1.5~10 | 0.5~9yr | 《Diagnosis and Curative Effect Criteria of Pediatric Diseases and Syndromes of Traditional Chinese Medicine》 | spleen deficiency | HM | 0.2% Zinc Sulphate | 1. TER 2. Weight gain (kg) 3. Height increase (cm) 4. Hemoglobin (g) 5. Improvement rate of systemic symptoms (hair, abdominal pain, dyschezia, tongue shape, tongue fur, pulse) 6. Improvement rate of hyperhidrosis 7. Prevention rate of getting cold 8. Hair Zn (ppm) 9. Urine D-xylose absorption rate | TG: None CG: abdominal pain 1,  nausea, vomiting and hemoglobin decreased 2 |
| Li 2005 | 120(60:60) | TG: 5.2  CG: 5.7 | TG: 6mo CG: 5mo | 《Diagnosis and Curative Effect Standard of TCM Internal and External Gynecology and Pediatric Diseases》 | NR | HM | Zinc Gluconate | 1. TER 2. Number of people whose appetite recovered within every 10d 2-1. Number of people whose appetite recovered within 10d 2-2. Number of people whose appetite recovered between within 20d 2-3. Number of people whose appetite recovered within 30d | TG: None CG: NR |
| Li 2006a | 63(38:25) | 1~13.5 | ≧1mo | 《TCM Syndrome Diagnosis and Curative Effect Standard》 | NR | HM | Multi-enzyme  + Zinc Gluconate | 1. TER | TG: None CG: NR |
| Li 2006b | 85(45:40) | TG: 1~10 CG: 1.5~10 | 38.5d (15d~3mo) | 《TCM Disease Treatment Standard》 《Practical Pediatrics》 | NR | HM | Pepsin mixture + Yeast tablet | 1. TER 2. Long-term effective rate 3. Recurrence rate | NR |
| Li 2010 | 200(100:100)→ 180(100:80) | TG: 2.5 CG: 2.6 | 13d (10d~1mo) | 《Practical Pediatrics》 《Criteria for Diagnosis and Efficacy of TCM Syndromes》 | spleen-stomach disharmony, spleen-stomach qi deficiency,  spleen-stomach yin deficiency,  spleen deficiency with effulgent liver | HM | Yeast tablet | 1. TER | NR |
| Li 2011 | 71(36:35) | 1~8 | >1mo | 《Diagnostic Criteria of Anorexia in Children》 | NR | HM | Domperidone  + Multi-enzyme | 1. TER | None |
| Li 2013 | 42(21:21) | TG: 5.21 ± 1.22 CG: 5.43 ± 1.37 | TG: 1.07 ± 0.23mo CG: 1.03 ± 0.32mo | 《Criteria for Diagnosis and Efficacy of TCM Diseases and Syndromes》 | NR | HM + CG | Multi-enzyme | 1. TER 2. Weight gain (kg) 3. Increment of food intake (g) | None |
| Li 2014a | 120(69:51) | TG: 4.29 ± 2.34 CG: 4.75 ± 1.58 | TG: 6.01 ± 4.03mo CG: 6.45 ± 3.24mo | 《Pediatric Abortion》 《Pediatric Abortion Syndrome》 | NR | HM | Domperidone  + Multi-enzyme + Compound Vitamin B | 1. Cure rate  1-1. Cure rate after 7d of treatment 1-2. Cure rate after 14d of treatment 1-3. Cure rate after 1mo of treatment 2. Recurrence rate | None |
| Li 2014b | 178(90:88) | TG: 4.32 ± 2.68 CG: 4.56 ± 2.82 | NR | 《Guiding Principles for Clinical Research of New Chinese Medicines》 | spleen-stomach disharmony, spleen-stomach qi deficiency, spleen-stomach yin deficiency | HM | Zinc Gluconate | 1. TER 2. Serum Zn (μmol/L) | NR |
| Li 2015a | 50(25:25) | TG: 6.72 ± 1.93 CG: 6.24 ± 3.13 | NR | 《Criteria for Diagnosis and Curative Effect of TCM Diseases》 《Guiding Principles for Clinical Research of New Chinese Medicines》 | spleen-stomach qi deficiency | HM | Zinc Gluconate  + Domperidone  + Probiotics (Saccharomyces boulardii) | 1. TER  2. Hemoglobin (change) 3. RBC (change) 4. Hematocrit (change) 5. Serum ferritin (change) 6. 25-Hydroxyvitamin D (change) | NR |
| Li 2015b | 64(33:31) | TG: 1~6 CG: 1~6 | TG: 2.5 ± 0.5yr  CG: 2.5 ± 0.5yr | 《Pediatrics of Chinese Medicine》 | spleen-stomach disharmony, spleen-stomach qi deficiency, spleen-stomach yin deficiency | HM | Zinc Gluconate | 1. TER | NR |
| Li 2016a | 123(62:61) | TG: 4.9 ± 1.57 CG: 4.6 ± 1.62 | TG: 29.8 ± 6.7d CG: 32.3 ± 5.9d | 《Criteria for Diagnosis and Efficacy of TCM Diseases and Syndromes》 《Pediatrics of Chinese Medicine》 | NR | HM | Domperidone | 1. TER 2. Weight (kg) 3. Serum Zn (μg/dL) 4. Hemoglobin (g/L) 5. Food intake (g/d) | N.S TG: None CG: mild nausea and vomiting 2 |
| Li 2016b | 86(43:43) | TG: 1~8 CG: 1~8 | TG: 7.18 ± 2.36mo CG: 7.09 ± 2.15mo | 《Guiding Principles for Clinical Research of New Chinese Medicines》 | NR | HM | Multi-enzyme | 1. TER 2. Serum Zn (μmol/L) 3. Serum Fe (μmol/L) 4. Serum Cu (μmol/L) 5. Serum Mg (mmol/L) 6. Serum IgA (g/L) 7. Serum IgG (g/L) 8. Serum IgM (g/L) | NR |
| Li 2017a | 214(106:108)→ 213(106:107) | TG: 4.89 ± 2.39 CG: 4.94 ± 2.52 | TG: 1.08 ± 0.70yr CG: 1.13 ± 0.71yr | 《Zhu Futang Practical Pediatrics》 《Guiding Principles for Clinical Research of New Chinese Medicines》 《Criteria for Diagnosis and Efficacy of TCM Diseases and Syndromes》 《Pediatrics of Integrated Traditional Chinese and Western Medicine》 | NR | HM | Probiotics (Bifidobacterium Lactobacillus Triple Live Bacteria) | 1. TER 2. Weight (kg) 3. Weight gain (kg) 4. Urine D-xylose excretion | None |
| Li 2017b | 120(60:60) | 6.7 | 1yr | 《Criteria for Diagnosis and Efficacy of TCM Diseases and Syndromes》 | NR | HM + CG | Probiotics (Bifidobacterium Triple Live Bacteria) + Zinc Sulfate | 1. TER | NR |
| Li 2019a | 66(33:33) | TG: 4.61 ± 1.08 CG: 4.73 ± 1.12 | NR | 《Technical Guidelines for Clinical Trial Design and Evaluation of New Chinese Medicines for Anorexia in Children》 | NR | HM + CG | Probiotics (Bifidobacterium Triple Viable Bacteria)  + Zinc Gluconate | 1. TER 2. Weight (kg) (after 6mo) 3. Recurrence rate | NR |
| Li 2019b | 97(49:48) | TG: 3.69 ± 0.45 CG: 3.73 ± 0.43 | TG: 4.71 ± 0.46mo CG: 4.65 ± 0.43mo | 《Zhu Futang Practical Pediatrics》 《Criteria for Diagnosis and Efficacy of TCM Diseases and Syndromes》 | spleen-stomach dampness-heat | HM + CG | Probiotics (Bifidobacterium Tetravaccine) + Cisapride + Zinc Gluconate | 1. TER 2. Leptin (μg/L) 3. Gastrin (ng/L) 4. Motilin (ng/L) 5. NPY (ng/L) 6. Serum albumin (mg/L) 7. Serum fibronectin (mg/L) | NR |
| Li 2020 | 80(40:40) | TG: 1~6 CG: 1~6 | NR | 《Guidelines for Diagnosis and Treatment of Common Diseases in Pediatrics of Traditional Chinese Medicine》 | spleen-stomach disharmony | HM | Compound Digestive Enzyme | 1. TER 2. TCM symptom score | NR |
| Lian 2008 | 80(40:40) | 4.5 | NR | 《Pediatrics of Chinese Medicine》 | NR | HM | Zinc Gluconate | 1. TER 2. Effective rate of every wk 2-1. 1st wk 2-2. 2nd wk 2-3. 3-4th wk | NR |
| Lian 2013 | 80(40:40) | TG: 1~12 CG: 1~12 | TG: 18.0 ± 0.85d CG: 17.5 ± 0.78d | 《Guiding Principles for Clinical Research of New Chinese Medicines》 | spleen deficiency with effulgent liver | HM | Zinc Gluconate | 1. TER 2. Effective rate of every wk 2-1. 1st wk 2-2. 2nd wk 2-3. 3rd wk | TG: vomiting 1 (disappeared after being instructed to properly feed the medicine.) CG: None |
| Lian 2020a | 80(40:40) | TG: 8.28 ± 0.55 CG: 8.31 ± 0.36 | NR | 《Guiding Principles for Clinical Research of New TCM Drugs》 《Pediatrics of Chinese Medicine》 《Zhu Futang Practical Pediatrics》 | spleen deficiency with effulgent liver | HM | Zinc Gluconate | 1. TER 2. Serum Zn (μmol/L) 3. VIP (pg/L) 4. TCM symptom score 5. Normal rate of electro-gastrogram | NR |
| Lian 2020b | 120(40:40:40) | TG: 8.25 ± 0.47 CG1: 8.41 ± 0.56 CG2: 8.35 ± 0.46 | TG: 2wk~6mo CG1: 2wk~6mo CG2: 2wk~6mo | 《Zhu Futang Practical Pediatrics》 《Guiding Principles for Clinical Research of New Chinese Medicines》 《Pediatrics of Chinese Medicine》 | spleen deficiency with effulgent liver | HM | CG1: No treatment (health education) CG2: Zinc Gluconate | 1. TER 2. Leptin (μg/L) 3. NPY (ng/L) 4. VIP (ng/L) 5. TCM symptom score | NR |
| Liang 2009 | 158(108:50) | TG: 4.1 CG: 4.2 | TG: 1.1yr CG: 1.1yr | 《Pediatrics of Chinese Medicine》 | NR | HM + CG | Zinc Gluconate | 1. TER 2. Hemoglobin recovery rate in people with below-normal hemoglobin levels | NR |
| Liang 2014a | 70(35:35) | TG: 3.8 ± 1.37 CG: 4.1 ± 1.38 | TG: 4.3 ± 3.42mo CG: 5.4 ± 6.17mo | 《Diagnosis and Curative Effect Criteria of Pediatric Diseases and Syndromes of Traditional Chinese Medicine》 | NR | HM | Zinc Gluconate | 1. TER | NR |
| Liang 2014b | 120(60:60) | 5~15 | NR | 《Criteria for Diagnosis and Efficacy of TCM Diseases and Syndromes》 《Practical Pediatrics》 | spleen deficiency with effulgent liver | HM | Probiotics (Bacillus Subtilis and Enterococcus Double Live Bacteria Multi-dimensional) + Zinc Gluconate | 1. TER 2. Main symptom score (loss of appetite, decreased food intake) | NR |
| Lin 2008 | 60(30:30) | TG: 4.6 CG: 5.3 | TG: 12mo CG: 10mo | 《Criteria for Diagnosis and Efficacy of TCM Diseases and Syndromes》 | spleen failing in transportation | HM | Vitamin B complex  + Multi-enzyme + Zinc or Ca agent | 1. TER 2. Serum Zn (μmol/L) 3. Hemoglobin increase (g/L) 4. Serum Ca (mmol/L) 5. RBC increase (x10^2^/L) 6. Urine amylase (U) | NR |
| Lin 2012 | 36(18:18)→ 35(18:17) | TG: 4.493 ± 1.596 CG: 4.477 ± 1.708 | TG: 1.772 ± 1.627 CG: 1.610 ± 1.379 (no unit) | 《Guiding Principles for Clinical Research of New Chinese Medicines》  《Pediatrics of Chinese Medicine》 | spleen failing in transportation | HM | Placebo | 1. TER 2. Weight (kg) 3. Weight gain (kg) 4. Hemoglobin (g/L) 5. Effective rate of improving food intake 6. Effective rate of improving TCM symptom 7. Main symptom score  8. TCM symptom score 9. Urine D-xylose excretion 10. Urine D-xylose excretion change 11. ALT (U/L) 12. BUN (mmol/L) 13. Creatinine (ummol/L) | None |
| Liu 2006 | 105(60:45) | TG: 1~6 CG: 1~6 | NR | 《Diagnosis and Curative Effect Criteria of Pediatric Diseases and Syndromes of Traditional Chinese Medicine》 | NR | HM | Zinc syrup | 1. TER 2. Hair Zn | TG: None CG: NR |
| Liu 2008 | 70(35:35) | TG: 2~12 CG: 2~12 | TG: 8mo CG: 7.7mo | 《Criteria for Diagnosis and Efficacy of TCM Diseases and Syndromes》 | NR | HM + CG | Multi-enzyme  + Zinc Gluconate | 1. TER 2. Weight (kg) 3. Hemoglobin (g/L) 4. Food intake (g) | NR |
| Liu 2010 | 100(50:50) | TG: 1~14 CG: 1~14 | NR | 《Zhu Futang Practical Pediatrics》 《Criteria for Diagnosis and Efficacy of TCM Diseases and Syndromes》 | spleen deficiency with effulgent liver | HM | Zinc Gluconate | 1. TER 2. Serum Zn (mg/L) 3. Improvement of appetite 4. Improvement of food intake | NR |
| Liu 2011 | 156(80:76) | TG: 4.52 ± 0.83 CG: 4.38 ± 0.76 | TG: 36 ± 12.77mo CG: 34 ± 13.51mo | 《Pediatrics of Chinese Medicine》 | NR | HM | Pepsin mixture | 1. TER 2. Height 3. Weight  4. Hemoglobin 5. Thickness of subcutaneous fat 6. RBC 7. Total protein | None |
| Liu 2012 | 180(100:80) | TG: 4.5 CG: 4.3 | TG: mean 1.4yr CG: 2.5mo~2.1yr | 《Pediatrics of Chinese Medicine》 | spleen-stomach disharmony, spleen-stomach qi deficiency, spleen-stomach yin deficiency | HM | Zinc Gluconate | 1. TER | NR |
| Liu 2014 | 200(150:50) | TG: 1~6 CG: 1~6 | TG: 12.6 ± 5.2wk CG: 11.9 ± 5.5wk | 《Zhu Futang Practical Pediatrics》 《Criteria for Diagnosis and Efficacy of TCM Diseases and Syndromes》 | NR | HM | Probiotics (Bifidobacterium Lactobacillus Triple Live Bacteria) + Domperidone | 1. TER 2. Appetite score 3. Food intake score 4. TCM symptom score | TG: 0 CG: skin rash 1, dizziness 1 |
| Liu 2016 | 60(30:30) | TG: 4.94 ± 1.27 CG: 4.89 ± 1.30 | TG: 2.96 ± 1.72mo CG: 2.73 ± 1.50mo | 《Practical Pediatrics》 《Pediatrics of Chinese Medicine》 | spleen failing in transportation | HM | Zinc Gluconate | 1. TER 2. TCM symptom score 3. Individual symptom score 4. Serum Zn (μg/mL) 5. Hemoglobin (g/L) | None |
| Liu 2017 | 106(53:53) | TG: 4.11 ± 0.82 CG: 4.23 ± 0.67 | TG: 6.5 ± 1.5mo CG: 6.6 ± 1.8mo | 《Zhu Futang Practical Pediatrics》 《Criteria for Diagnosis and Efficacy of TCM Diseases and Syndromes》 《Guiding Principles for Clinical Research of New Chinese Medicines》 | spleen failing in transportation with spleen qi depletion | HM + CG | Probiotics (Saccharomyces boulardii) + 0.2% Zinc Sulfate + Ferric Amine Citrate  + Multi-vitamin | 1. TER 2. Main symptom score (food refusal, constipation, intermittent abdominal pain, weight loss) 3. TCM symptom score 4. Serum leptin (μg/L) 5. Serum ghrelin (μg/L) 6. Serum NPY (μg/L) | None |
| Liu 2018a | 60(30:30) | TG: 6.02 ± 5.24 CG: 6.47 ± 5.19 | NR | 《Standards for Diagnosis and Treatment of Infantile Anorexia》 《Pediatrics of Chinese Medicine》 | NR | HM | Medilac-Vita | 1. Food intake symptom score  2. Height (cm) 3. Weight (kg) 4. Serum orexin-A (ng/L) 5. Serum gastrin (ng/L) 6. Serum NPY (ng/L) | None |
| Liu 2018b | 120(60:60) | TG: 6.5 ± 2.0 CG: 6.3 ± 1.5 | TG: 5.2 ± 1.8mo CG: 5.4 ± 2.0mo | 《Pediatrics of Chinese Medicine》 | spleen deficiency with effulgent liver, spleen-stomach qi deficiency, spleen-stomach disharmony | HM | Domperidone | 1. TER 2. Appetite recovery time (cases) | NR |
| Luo 2014 | 80(40:40) | 1~12 | 2~12mo | 《Pediatrics of Chinese Medicine》 | spleen deficiency with effulgent liver | HM | Zinc Gluconate | 1. TER | NR |
| Ma 2011 | 196(98:98) | TG: 2~12 CG: 2~12 | TG: 5.5 ± 1.5mo CG: 5.2 ± 1.4mo | 《Criteria for Diagnosis and Efficacy of TCM Diseases and Syndromes》 《Pediatrics of Chinese Medicine》 | spleen-stomach qi deficiency | HM | Five Vitamins  + Lysine syrup | 1. TER 2. Serum Zn (umol/L) | NR |
| Ma 2017 | 70(35:35)→65(33:32) | TG: 5.36 ± 3.277 CG: 4.72 ± 3.275 | TG: <1yr (9 cases), 1~2yr (10 cases), ≥2yr (14 cases) CG: <1yr (8 cases), 1~2yr (10 cases), ≥2yr (14 cases) | 《Zhu Futang Practical Pediatrics》 《Criteria for Diagnosis and Efficacy of TCM Diseases and Syndromes》 | spleen-stomach yin deficiency | HM | Probiotics (Bifidobacterium Quadruple Viable Bacteria) | 1. TER 2. TER (TCM symptom score) 3. TCM symptom score 4. Recurrence rate  5. Serum Zn (umol/L) 6. Serum Fe (umol/L) 7. Serum Ca (umol/L) | NR |
| Meng 2020 | 60(20:20:20) | TG1: 7.4 ± 1.3 TG2: 6.8 ± 1.0 CG: 6.0 ± 1.6 | TG1: median 4.5yr TG2: median 5yr  CG: median 4yr | 《Zhu Futang Practical Pediatrics》 | deficiency-excess complex | TG1: HM + CG TG2: HM | Probiotics tablets (Bacillus Coagulans Live Bacteria) | 1. TER 2. TCM symptom score 3. Weight (kg) | None |
| Pan 2006 | 80(40:40) | 3.2 | 3wk~2mo: 26 cases, 2~6mo: 34 cases, >6mo: 20 cases | 《Criteria for Diagnosis and Efficacy of TCM Diseases and Syndromes》 | spleen deficiency with effulgent liver | HM | Probiotics tablets (Bifidobacterium Lactobacillus Triple Live Bacteria) | 1. TER 2. Weight gain (kg) 3. RBC increase 4. Hemoglobin increase (g/L) 5. Appetite recovery time (cases) | NR |
| Peng 2008 | 80(40:40) | TG: <1 (5 cases), 1~3 (12 cases), 3~7 (15 cases), 7~11 (8 cases)  CG: 2~10 | TG: 1mo~2yr | 《Pediatrics of Chinese Medicine》 | NR | HM | Multi-enzyme tablets | 1. TER | NR |
| Peng 2015 | 90(60:30) | 1~14 | TG: 5.68 ± 0.983wk CG: 5.97 ± 1.402wk | 《Zhu Futang Practical Pediatrics》 《Guiding Principles for Clinical Research of New Chinese Medicines》 | spleen failing in transportation | HM | Zinc Sulfate | 1. TER 2. TER (TCM symptom score) 3. TCM symptom score 4. Urine D-xylose excretion rate (g/L) | None |
| Pi 2011 | 60(30:30) | TG: 5.6 CG: 5.8 | TG: 6.5mo CG: 5.5mo | 《Criteria for Diagnosis and Therapeutic Effect of Internal and External Gynecological Diseases in Traditional Chinese Medicine》 | NR | HM | Zinc Gluconate | 1. TER 2. Appetite recovery time (cases) | NR |
| Qi 2009 | 83(43:40) | TG: 6  CG: 6 | TG: 15d~2yr CG: 30d~2yr | 《Criteria for Diagnosis and Efficacy of TCM Diseases and Syndromes》 | spleen-stomach weakness, damage to yin of spleen-stomach | HM | Zinc Gluconate | 1. TER | NR |
| Qin 2012 | 80(30:25:25) | TG: 1~6 CG1: 1~5.5 CG2: 1.5~6 | NR | 《Practical Pediatrics》 《Pediatrics of Chinese Medicine》 | NR | HM | CG1: Zinc Gluconate CG2: Pepsin | 1. TER | NR |
| Qiu 2011 | 130(65:65) | 7.5 | 12.5yr | 《Criteria for Diagnosis and Efficacy of TCM Diseases and Syndromes》 | spleen failing in transportation | HM | Multi-enzyme + Compound Vitamin B + Zinc or Calcium agents | 1. TER 2. Serum Ca (mmol/L) 3. Serum Zn (μmol/L) 4. Urine amylase | NR |
| Qiu 2017 | 135(68:67) | TG: 6.6 ± 2.7 CG: 6.4 ± 2.7 | TG: 5.7 ± 2.5mo CG: 5.7 ± 2.5mo | 《Zhu Futang Practical Pediatrics》 《Pediatrics of Chinese Medicine》 | spleen-stomach disharmony, spleen-stomach qi deficiency, spleen deficiency with effulgent liver | HM | Domperidone | 1. TER 2. TCM symptom score | NR |
| She 2004 | 209(131:78) | TG: <3 (44 cases), 3-6 (53 cases), >7 (34 cases) CG: <3 (27 cases), 3-6 (31 cases), >7 (20 cases) | NR | 《Criteria for Diagnosis and Curative Effect of TCM Diseases》 | spleen-stomach qi deficiency | HM | Lactobacillus | 1. Severity of the spleen and stomach qi 2. TER 3. Urine D-xylose excretion rate (%) 4. Urine amylase activity | NR |
| Shi 2020 | 104(52:52) | TG: 6.08 ± 1.72 CG: 6.02 ± 1.79 | TG: 5.89 ± 1.11mo CG: 5.88 ± 1.14mo | 《Guidelines for Diagnosis and Treatment of Common Diseases in Pediatrics of Traditional Chinese Medicine》 | spleen failing in transportation | HM | Probiotics  (Bifidobacterium Quadruple Viable Bacteria) | 1. Serum motilin (ng/L) 2. Substance P (pg/ml) 3. Hemoglobin (g/L) 4. TCM symptom score 5. TER | NR |
| Su 2015 | 221(111:110) | TG: 4.21 ± 1.32 CG: 4.25 ± 2.06 | TG: 7.92 ± 3.21mo CG: 7.88 ± 2.56mo | 《Diagnostic Criteria for Curative Effect of Traditional Chinese and Western Medicine》 | NR | HM + CG | Multi-enzyme or Pepsin mixture  + Domperidone  + Zinc Gluconate | 1. TER | NR |
| Sun 2009 | 300(200:50:50) | 1~8 | 2.5mo~3yr | 《Pediatrics of Chinese Medicine》 《Criteria for Diagnosis and Efficacy of TCM Diseases and Syndromes》 | spleen failing in transportation, spleen-stomach qi deficiency, stomach yin deficiency | TG1: HM TG2: HM | Pepsin mixture | 1. TER | NR |
| Sun 2012 | 150(78:72) | TG: 4.2 ± 0.8 CG: 4.1 ± 0.7 | TG: 8.5 ± 1.3mo CG: 8.2 ± 1.1mo | 《Zhu Futang Practical Pediatrics》  《Criteria for Diagnosis and Efficacy of TCM Diseases and Syndromes》 | NR | HM + CG | Zinc Gluconate  + Domperidone | 1. TER | None |
| Sun 2015 | 60(30:30) | TG: 10.47 ± 1.55 CG: 10.40 ± 1.40 | more than 2 wk | 《Zhu Futang Practical Pediatrics》 《Guiding Principles for Clinical Research of New Chinese Medicines》 | liver depression and spleen deficiency | HM | Zinc Gluconate | 1. TER 2. Weight gain 3. Average time to regain ideal weight (d) 4. TCM symptom score | NR |
| Sun 2019 | 80(40:40) | TG: 4.34 ± 1.32 CG: 4.50 ± 1.42 | TG: 5.09 ± 2.76mo CG: 5.25 ± 2.90mo | 《Practical Pediatrics》 | NR | HM + CG | Lysine Vitamin B12 | 1. TER 2. Serum Fe (μmol/L) 3. Serum Ca (mmol/L) 4. Serum Zn (μmol/L) 5. Hemoglobin (g/L) 6. Serum amylase (U/L) 7. Serum ferritin (μg/L) | N.S TG: diarrhea 1, nausea 1, skin rash 1 CG: dizziness 1, nausea 1 |
| Sun 2020 | 92(46:46) | TG: 4.5 ± 1.2 CG: 4.1 ± 1.0 | TG: 5.2 ± 1.5mo CG: 5.5 ± 1.4mo | 《Traditional Chinese Medicine Treatment of Infantile Anorexia》 | NR | HM + CG | Domperidone  + Calcium Gluconate | 1. TER 2. Symptom improvement time (abdominal pain, abdominal distension, constipation) (d) 3. Serum Zn (μmol/L) 4. Weight (kg) | None |
| Tang 2005 | 137(67:35:35) | 1~8 | more than 3mo | 《Standards for Diagnosis and Treatment of Infantile Anorexia》 | NR | TG1: HM + CG TG2: HM | Probiotics (Bifidobacterium Lactobacillus Triple Live Bacteria) | 1. TER | NR |
| Tao 2011 | 99(51:24:24) | NR | more than 6mo | 《Criteria for Diagnosis and Efficacy of TCM Diseases and Syndromes》 | NR | TG1: HM + CG TG2: HM | Zinc Gluconate | 1. TER | NR |
| Wang 2010 | 100(50:50) | 1~8 | 3~11mo | 《Pediatrics of Chinese Medicine》 | spleen deficiency with effulgent liver | HM | Zinc Sulfate | 1. TER | NR |
| Wang 2012a | 80(40:40) | TG: 5.7 CG: 6.9 | NR | 《Zhu Futang Practical Pediatrics》 《Criteria for curative effect of TCM Internal and External Gynecological Diseases》 | spleen yin deficiency | HM | Zinc Gluconate | 1. TER 2. Weight gain (kg) 3. TCM symptom score | NR |
| Wang 2012b | 60(30:30) | TG: 3.53 ± 1.80 CG: 3.43 ± 1.63 | TG: 2~8wk (14 cases), 8~14wk (12 cases), 14~20wk (4 cases) CG: 2~8wk (11 cases), 8~14wk (14 cases), 14~20wk (5 cases) | 《Criteria for Diagnosis and Efficacy of TCM Diseases and Syndromes》 《Zhu Futang Practical Pediatrics》 | spleen deficiency | HM | Compound four vitamins and ferrous fumarate powder | 1. TER 2. Hemoglobin 3. Main symptom score 4. Secondary symptom score 5. TCM symptom score | None |
| Wang 2012c | 94(54:40) | TG: 4.1 ± 1.1 CG: 4.2 ± 1.4 | more than 2wk | 《Zhu Futang Practical Pediatrics》 《Criteria for Diagnosis and Efficacy of TCM Syndromes》 | spleen-stomach qi deficiency | HM | Pepsin  + Domperidone | 1. TER 2. BMI 3. Serum leptin (μg/L) | NR |
| Wang 2012d | 120(60:60) | TG: 4.2  CG: 4.1 | TG: 7.8mo CG: 8.5mo | 《Criteria for Diagnosis and Efficacy of TCM Diseases and Syndromes》 | spleen-stomach weakness | HM | Multivitamin B  + Multi-enzyme, etc | 1. TER | NR |
| Wang 2014 | 60(30:30)→59(30:29) | TG: 1~6 CG: 1~6 | more than 3mo | 《Zhu Futang Practical Pediatrics》 《Pediatrics of Chinese Medicine》 《Guiding Principles for Clinical Research of New Chinese Medicines》 | spleen deficiency with dumpness-heat | HM | Probiotics (Bifidobacterium Quadruple Viable Bacteria) + Zinc Gluconate | 1. TER 2. TER (TCM symptom score) 3. TCM symptom score | None |
| Wang 2015a | 114(58:56) | TG: 3.58 ± 1.08 CG: 3.69 ± 1.12 | TG: 1.45 ± 0.42yr CG: 1.49 ± 0.48yr | 《Zhu Futang Practical Pediatrics》 | NR | HM + CG | Lactase  + Pepsin solution | 1. TER 2. TCM main symptom score 3. Serum zinc (μmol/L) 4. Hemoglobin (g/L) | None |
| Wang 2015b | 120(60:60) | TG: 4.8 ± 0.6 CG: 4.9 ± 0.7 | TG: 8.3 ± 0.9mo CG: 8.1 ± 0.8mo | 《Zhu Futang Practical Pediatrics》 《Guiding Principles for Clinical Research of New Chinese Medicines》 | spleen-stomach disharmony | HM + CG | Multi-enzyme | 1. TER 2. TCM symptom score 3. Weight 4. BMI 5. Plasma CCK-8 (ng/L) 6. Plasma β-endorphin (ng/L) | NR |
| Wang 2015c | 92(46:46) | TG: 6.54 ± 4.68 CG: 7.37 ± 4.23 | TG: 69.6 ± 7.3d CG: 71.1 ± 6.2d | 《Guiding Principles for Clinical Research of New Chinese Medicines》 | NR | HM | Multi-enzyme | 1. TER 2. Recurrence rate | TG: None CG: dry stool 2 |
| Wang 2016 | 80(40:40) | TG: 3.5 ± 0.9 CG: 3.3 ± 1.1 | TG: 60 ± 10d CG: 65 ± 5d | 《Zhu Futang Practical Pediatrics》 《Pediatrics of Chinese Medicine》 | spleen deficiency | HM | Domperidone | 1. TER | NR |
| Wang 2017a | 96(48:48) | TG: 4.15 ± 0.67 CG: 4.21 ± 0.65 | TG: 8.03 ± 0.99mo CG: 8.17 ± 0.96mo | 《Zhu Futang Practical Pediatrics》 《Guiding Principles for Clinical Research of New Chinese Medicines》 | spleen-stomach qi deficiency | HM + CG | Multi-enzyme | 1. TER 2. Weight 3. BMI 4. TCM symptom score 5. Plasma β-endorphin (ng/L)  6. Leptin (ng/L) | NR |
| Wang 2017b | 86(43:43) | TG: 5.06 ± 1.31 CG: 5.11 ± 1.25 | TG: 2.29 ± 0.64yr CG: 2.12 ± 0.54yr | 《Zhu Futang Practical Pediatrics》 | NR | HM  + Ca + Zinc Gluconate | Probiotics (Bifidobacterium Quadruple Live Bacteria)  + Ca  + Zinc Gluconate | 1. TER 2. Weight | During the treatment, both groups of children had adverse reactions, such as vomiting and diarrhea. (No numerical data) |
| Wang 2017c | 126(63:63) | TG: 4.31 ± 1.52 CG: 3.94 ± 1.47 | TG: 3.85 ± 1.38wk CG: 3.26 ± 1.74wk | 《Practical Pediatrics》 《Pediatrics of Chinese Medicine》 | spleen failing in transportation | HM | Domperidone | 1. TER 2. Blood lead (ug/L) 3. Serum leptin (pg/ml) 4. NPY (pg/ml) | N.S TG: nausea 2, abdominal pain 1 CG: vomiting 3, abdominal pain 2 |
| Wang 2017d | 154(77:77) | TG: 4.1 ± 1.3 CG: 4.2 ± 1.6 | TG: 1.6 ± 0.5yr CG: 1.4 ± 0.3yr | 《Criteria for Diagnosis and Efficacy of TCM Diseases and Syndromes》 | NR | HM + CG | Probiotics (Bifidobacterium Triple Live Bacteria) + Multi-enzyme | 1. TER 2. TCM symptom score | None |
| Wang 2018a | 68(34:34) | TG: 4.22 ± 1.23 CG: 3.91 ± 1.07 | TG: 3.93 ± 1.13mo CG: 4.54 ± 1.42mo | 《Criteria for Diagnosis and Efficacy of TCM Diseases and Syndromes》 《Practical Pediatrics》 | NR | HM + CG | Probiotics (Bifidobacterium Triple Live Bacteria) | 1. TER 2. Weight 3. Appetite recovery time (d) 4. Abdominal pain and bloating disappear time (d) | NR |
| Wang 2018b | 280(140:140) | TG: 4.7 ± 0.7 CG: 4.6 ± 0.7 | TG: 35.8 ± 11.2mo CG: 35.6 ± 11.3mo | 《Pediatrics of Chinese Medicine》 | NR | HM | Pepsin mixture | 1. TER 2. Weight gain (kg) 3. Hemoglobin increase (g/dl) | NR |
| Wang 2018c | 82(41:41) | TG: 4.42 ± 1.19 CG: 3.89 ± 1.47 | TG: 4.77 ± 1.05mo CG: 5.17 ± 1.31mo | 《Zhu Futang Practical Pediatrics》 | NR | HM + CG | Probiotics (Bifidobacterium Triple Live Bacteria) | 1. TER 2. Abdominal subcutaneous fat thickness (cm) | None |
| Wang 2018d | 86(43:43) | TG: 3.37 ± 1.75 CG: 3.20 ± 1.88 | TG: 8.65 ± 3.14mo CG: 9.19 ± 3.43mo | 《Zhu Futang Practical Pediatrics》 《Technical Guidelines for Clinical Trial Design and Evaluation of New Chinese Medicines for Anorexia in Children》 | spleen-stomach dampness-heat | HM | Probiotics (Bifidobacterium Quadruple Viable Bacteria) + Cisapride  + Zinc Gluconate | 1. TCM symptom score 2. Motilin (ng/L) 3. Gastrin (ng/L) 4. NPY (ng/L) 5. Albumin (mg/L) 6. Fibronectin (mg/L) 7. Leptin (μg/L) | NR |
| Wang 2019a | 74(37:37) | TG: 5.3 ± 1.9 CG: 5.4 ± 2.1 | TG: 9.4 ± 2.8mo CG: 9.5 ± 2.6mo | 《Zhu Futang Practical Pediatrics》 《Pediatrics of Chinese Medicine》 | NR | HM + CG | Calcium  + Zinc Gluconate | 1. TER 2. Treatment satisfaction of children's family members | NR |
| Wang 2019b | 134(67:67) | TG: 6.23 ± 1.02 CG: 6.54 ± 1.18 | TG: 8.31 ± 1.56mo CG: 8.59 ± 1.24mo | 《Standards for Diagnosis and Treatment of Infantile Anorexia》 《Guidelines for Diagnosis and Treatment of Common Diseases in Pediatrics of Traditional Chinese Medicine》 | NR | HM + CG | Multi-enzyme  + Zinc Gluconate | 1. TER 2. Serum Fe (μg/L) 3. Serum Zn (μg/L) 4. Serum Cu (μg/L) 5. Hemoglobin (g/L) | None |
| Wang 2020a | 104(52:52) | TG: 7.72 ± 2.35 CG: 7.64 ± 2.37 | TG: 2.98 ± 0.91mo CG: 3.02 ± 0.89mo | 《Zhu Futang Practical Pediatrics》 《Guiding Principles for Clinical Research of New Chinese Medicines》 | spleen-stomach dampness-heat | HM + CG | Zinc Gluconate  + Vitamin B Complex | 1. TER 2. TCM symptom score 3. Appetite recovery time (d) 4. Food intake recovery time (d) 5. Hemoglobin (g/L) 6. Serum Zn (mmol/L) 7. Serum Ca (mmol/L) | None |
| Wang 2020b | 86(43:43) | TG: 3.85 ± 2.46 CG: 3.46 ± 2.1 | more than 2mo | 《Zhu Futang Practical Pediatrics》 《Criteria for Diagnosis and Efficacy of TCM Diseases and Syndromes》 | spleen-stomach yin deficiency | HM | Probiotics (Bacillus Subtilis Dual Live Bacteria) | 1. TER 2. Leptin (μg/L) 3. Orexin (ng/ml) 4. Serum Zn (μmol/L) | NR |
| Wang 2020c | 104(52:52) | TG: 4.51 ± 1.01 CG: 4.03 ± 1.42 | NR | 《Zhu Futang Practical Pediatrics》 《Criteria for Diagnosis and Efficacy of TCM Diseases and Syndromes》 | NR | HM + CG | Multi-enzyme + Probiotics (Quadruple Bifidobacterium Live Bacteria) | 1. TER | NR |
| Wei 2015 | 108(55:53) | TG: 4.26 ± 1.31 CG: 4.51 ± 1.63 | TG: 3.56 ± 1.07yr CG: 3.78 ± 1.19yr | 《Zhu Futang Practical Pediatrics》 | NR | HM + CG | Domperidone | 1. TER 2. Comparison of clinical symptom relief time (abdominal distension, abdominal pain, constipation) (d) 3. Recurrence rate | TG: diarrhea 1 CG: diarrhea 3, stomachache 2, vomiting 1, skin rash 1 |
| Wu 2001 | 144(68:40:36) | NR | 2mo~5yr | 《Criteria for Diagnosis and Efficacy of TCM Diseases and Syndromes》 | NR | HM | CG1: Vitamin B Complex liquid CG2: Zinc Gluconate | 1. TER | NR |
| Wu 2003 | 128(68:60) | TG: 4.1 CG: 4.3 | TG: 2.9mo CG: 2.8mo | 《Criteria for Diagnosis and Efficacy of TCM Diseases and Syndromes》 | NR | HM | Zinc Gluconate | 1. TER 2. Recurrence rate | NR |
| Wu 2007 | 112(56:56) | 5.92 | 6.23mo | 《Guiding Principles of Clinical Research on the Treatment of Infantile Anorexia by New Chinese Herbal Medicines》 | NR | HM | Multi-enzyme tablets  + Zinc Gluconate | 1. TER 2. Food intake (g/d) 3. Urine D-xylose excretion rate | None |
| Wu 2009 | 156(80:76) | TG: 4.52 ± 0.83 CG: 4.38 ± 0.76 | TG: 36 ± 12.77mo CG: 34 ± 13.51mo | 《Practical Pediatrics》 《Pediatrics of Chinese Medicine》 | NR | HM + CG | Zinc Gluconate  + Medilac-Vita | 1. TER | NR |
| Wu 2011 | 40(20:20) | TG: 1~6 CG: 1~6 | NR | 《Guiding Principles for Clinical Research of New Chinese Medicines》 | spleen deficiency food accumulation | HM | Lactase  + Domperidone | 1. TER | None |
| Wu 2016 | 500(125:125:125:125) | TG1: 3.5 ± 2.3 TG2: 3.3 ± 2.2 TG3: 3.7 ± 2.0 CG: 3.7 ± 2.3 | 2mo~4yr | 《Practical Pediatrics》 《Pediatrics of Chinese Medicine》 | spleen failing in transportation | TG1: Chuna TG2: HM TG3: Chuna + HM | Zinc Sulfate  + Yeast Flakes | 1. TER 2. Hemoglobin (g/L) 3. Serum Zn (μmol/L) 4. TCM syndrome score | None |
| Wu 2018 | 180(90:90) | TG: 5.7 ± 1.1 CG: 6.1 ± 0.6 | TG: 7.3 ± 0.6mo CG: 8.0 ± 1.3mo | 《Zhu Futang Practical Pediatrics》 《Pediatrics of Chinese Medicine》 | NR | HM + CG | Lysine Vitamin B12 | 1. TER 2. Weight | None |
| Xia 2016 | 150(90:60) | TG: 1~12 CG: 1~11 | TG: 1.5yr CG: 1.6yr | 《Zhu Futang Practical Pediatrics》 《Criteria for Diagnosis and Efficacy of TCM Diseases and Syndromes》 | NR | HM | Medilac-Vita  + Lysine Vitamin B12 | 1. TER | NR |
| Xing 2012 | 90(60:30) | 1~14 | TG: 5.68 ± 0.98mo CG: 5.97 ± 1.40mo | 《Zhu Futang Practical Pediatrics》 《Guiding Principles for Clinical Research of New Chinese Medicines》 | spleen failing in transportation | HM | Zinc Sulfate | 1. TER 2. TCM symptom score 3. Urine D-xylose excretion (g/L)  4. TER (TCM syndrome score) | None |
| Xiong 2013 | 241(131:110) | TG: 3.83 ± 0.69 CG: 3.67 ± 0.52 | TG: 8 ± 2.57mo CG: 9 ± 3.24mo | 《Pediatrics of Chinese Medicine》 | spleen failing in transportation, spleen-stomach qi deficiency | HM | Lactobacillus + Zinc Gluconate  + Medilac-Vita, etc. | 1. TER | NR |
| Xu 2005 | 120(64:56) | TG: 2.6 ± 1.5 CG: 2.5 ± 1.6 | TG: 3.5 ± 0.9mo CG: 3.8 ± 0.9mo | 《Criteria for Diagnosis and Curative Effect of TCM Diseases》 | spleen deficiency with effulgent liver, spleen deficiency with dampness encumbrance, dual deficiency of the lung-spleen | HM | Lactase  + Multi-enzyme + Saccharated Yeast  + Zinc Gluconate | 1. TER | NR |
| Xu 2008 | 160(120:40) | TG: ≥1 CG: ≥1 | TG: 4wk~1yr CG: NR | 《Diagnosis and Curative Effect Criteria of Pediatric Diseases and Syndromes of Traditional Chinese Medicine》 《Pediatrics of Chinese Medicine》 | spleen failing in transportation, spleen-stomach qi deficiency | HM | Medilac-Vita | 1. TER 2. Weight 3. Food intake (g/d) | TG: None CG: NR |
| Xu 2015 | 66(33:33) | TG: 4.9 ± 0.7 CG: 5.1 ± 0.6 | TG: 5.8 ± 0.8mo CG: 6.0 ± 0.9mo | 《Zhu Futang Practical Pediatrics》 《Pediatrics of Chinese Medicine》 | spleen failing in transportation | HM + CG | Probiotics (Bifidobacterium Triple Viable Bacteria) + Zinc Gluconate | 1. TER 2. Recurrence rate | NR |
| Xue 2016 | 88(44:44) | TG: 4.7 ± 1.5 CG: 4.5 ± 1.6 | TG: 7.7 ± 3.1mo CG: 7.8 ± 3.3mo | 《Diagnosis and Treatment of Feeding Disorders in Infants, Toddlers, and Young Children》 《Standards for Diagnosis and Treatment of Infantile Anorexia》 | NR | HM + CG | Zinc Gluconate  + Medilac-Vita | 1. TER | NR |
| Yan 2020 | 62(31:31) | TG: 2.4 ± 0.3 CG: 2.3 ± 0.5 | NR | 《Criteria for Diagnosis and Curative Effect of TCM Diseases》 | NR | HM + CG | Probiotics (Saccharomyces Boulardii)  + Vitamins | 1. TER 2. TCM symptom score | NR |
| Yan 2021 | 60(30:30) | TG: 5.5 ± 1.0 CG: 5.1 ± 0.8 | TG: 8.4 ± 1.6mo CG: 7.8 ± 1.2mo | 《Pediatrics》 | NR | HM + CG | Zinc Gluconate | 1. TER 2. Feeding (ml/d) 3. Weight gain (kg) 4. Intermittent abdominal pain (cases, %) 5. Sleep quality (h/d) | NR |
| Yang 2004 | 98(52:46) | 6mo~8yr | NR | 《Standards for Diagnosis and Treatment of Infantile Anorexia》 | NR | HM | Multi-enzyme  + Yeast | 1. TER 2. Curative effect of anorexia complications | NR |
| Yang 2006 | 98(52:46) | 8mo~9yr | NR | 《Criteria for Diagnosis and Curative Effect of TCM Diseases》 | NR | HM | Pepsin mixture | 1. TER 2. Curative effect of anorexia complications | NR |
| Yang 2008 | 150(100:50) | TG: 2.5 ± 1.02 CG: 1~6.5 | TG: 1.10 ± 0.521yr CG: 1.08 ± 0.56yr | 《Criteria for Diagnosis and Therapeutic Effect of Women and Children's Diseases in Traditional Chinese Medicine》 | NR | HM | Multi-vitamin  + Multi-enzyme | 1. TER | NR |
| Yang 2010 | 86(50:36) | TG: 4.23 ± 0.95 CG: 4.81 ± 0.75 | NR | 《Guiding Principles for Clinical Research of New Chinese Medicines》 《Criteria for Diagnosis and Efficacy of TCM Diseases and Syndromes》 | spleen-stomach dampness-heat | HM + CG | Zinc Gluconate  + Multi-vitamine B | 1. TER | NR |
| Yang 2013a | 62(31:31) | TG: 3.1 ± 1.5 CG: 3.4 ± 1.7 | NR | 《Guiding Principles for Clinical Research of New Chinese Medicines》 《Criteria for Diagnosis and Curative Effect of TCM Diseases》 | spleen-stomach disharmony | HM | Compound digestive enzyme capsule | 1. TER 2. Serum Zn (umol/L) 3. Salivary amylase (U/L) 4. D-xylose absorption test (umol/L) | NR |
| Yang 2013b | 60(30:30) | TG: 8mo~14yr CG: 10mo~14yr | >2wk | 《Guiding Principles for Clinical Research of New Chinese Medicines》 | NR | HM + CG | Probiotics  (Bifidus Triple Viable Bacteria) | 1. TER | NR |
| Yang 2013c | 120(80:40) | TG: 4.8 CG: 4.6 | TG: 4wk~1yr CG: 4wk~1yr | 《Criteria for Diagnosis and Curative Effect of TCM Diseases》 | NR | HM | Routine western medicine | 1. TER | NR |
| Yang 2014 | 72(36:36) | 5.23 ± 2.35 | 4.32 ± 1.55mo | 《Practical Pediatrics》 | NR | HM | Zinc Gluconate | 1. TER 2. Weight (kg) 3. BMI 4. Hemoglobin (g/L)  5. Serum Zn (mg/L) | None |
| Yang 2020 | 82(41:41) | TG: 6.4 ± 1.2 CG: 6.2 ± 1.3 | TG: 0.7 ± 0.3yr CG: 0.8 ± 0.3yr | 《Zhu Futang Practical Pediatrics》 《Pediatrics of Chinese Medicine》 | spleen deficiency food accumulation | HM + CG | Probiotics (Bifidobacterium Quadruple Live Bacteria) | 1. TER | NR |
| Yao 2008 | 90(60:30) | TG: 6.57 ± 0.752 CG: 6.74 ± 0.551 | ≥2wk | 《Practical Pediatrics》  《Guiding Principles for Clinical Research of New Chinese Medicines》 | NR | HM | Zinc Gluconate | 1. TER | NR |
| Yi 2018 | 90(45:45) | TG: 4.30 ± 1.23 CG: 4.08 ± 1.28 | TG: 11.05 ± 2.39mo CG: 10.38 ± 2.42mo | 《Practical Pediatrics》 | NR | HM + CG | Lactobacillus + 0.2% Zinc Sulfate  + Vitamin A  + Vitamin B1  + Vitamin B2 | 1. TER 2. Subcutaneous fat thickness (cm) 3. Hemoglobin (g/L) 4. Salivary Zn (umol/L) 5. TCM symptom score 6. NPY (ng/L)  7. Motilin (ng/L) 8. Leptin (ng/mL) | None |
| You 2020 | 60(30:30) | TG: 3.45 ± 1.23 CG: 3.77 ± 1.52 | TG: 3.2 ± 1.5mo CG: 4.2 ± 1.8mo | 《Pediatrics of Integrated Traditional Chinese and Western Medicine》 | NR | HM + CG | Psychotherapy  + Nutrition Counseling  + Physical Exercise | 1. TER 2. TCM symptom score 3. Serum Zn (mg/L) 4. Serum Fe (mg/L) 5. Serum Ca (mg/L) | NR |
| Yu 2009a | 110(68:42) | NR | ≥1mo | 《Criteria for Diagnosis and Efficacy of TCM Diseases and Syndromes》 | NR | HM | Zinc Gluconate | 1. TER | NR |
| Yu 2009b | 100(50:50) | TG: 6.5 CG: 7.2 | NR | 《Criteria for Diagnosis and Efficacy of TCM Diseases and Syndromes》 《Practical Pediatrics》  《Guiding Principles for Clinical Research of New Chinese Medicines》 | spleen-stomach dampness-heat | HM | Zinc Gluconate | 1. TER 2. TCM symptom score 3. Weight change (kg) | NR |
| Yu 2014 | 100(50:50) | TG: 5.2 CG: 5.7 | ≥1mo | 《Criteria for Diagnosis and Efficacy of TCM Diseases and Syndromes》 | NR | HM | Lactase  + Multi-enzyme | 1. TER | NR |
| Yu 2016 | 240(80:80:80) | 1~12 | 0.8 ± 0.2yr | 《Zhu Futang Practical Pediatrics》 《Diagnosis and Curative Effect Criteria of Pediatric Diseases and Syndromes of Traditional Chinese Medicine》 | NR | TG1: HM + CG TG2: HM +CG + Seabuckthorn dry emulsion | Probiotics  (Bacillus Subtilis Dual Live Bacteria)  + Domperidone | 1. TER 2. BMI 3. Serum Zn (umol/L) 4. Subcutaneous fat thickness (cm) | NR |
| Yu 2017 | 84(42:42) | 1~12 | TG: 6.4 ± 0.7mo CG: 6.3 ± 0.6mo | 《Criteria for Diagnosis and Efficacy of TCM Diseases and Syndromes》 《Standards for Diagnosis and Treatment of Infantile Anorexia》 | NR | HM | Zinc Gluconate | 1. TER 2. Weight (kg) 3. Serum Ghrelin (ng/L) 4. Serum Leptin (ng/L) | NR |
| Yuan 2008 | 140(78:62) | NR | NR | 《Criteria for Diagnosis and Efficacy of TCM Diseases and Syndromes》 | NR | HM  + Zinc Gluconate  + Multi-vitamin B tablets | Gastric enzyme mixture or Multi-enzyme  + Zinc Gluconate  + Multi-vitamin B | 1. TER | NR |
| Yuan 2009 | 120(60:60) | 6mo~11yr | 1~18mo | 《Criteria for Diagnosis and Efficacy of TCM Diseases and Syndromes》 | liver depression and spleen deficiency | HM | Zinc Gluconate | 1. TER 2. TER (TCM symptom score) | NR |
| Yuan 2010 | 200(100:100) | TG: 5.2 CG: 5.7 | ≥1mo | 《Criteria for Diagnosis and Efficacy of TCM Diseases and Syndromes》 | NR | HM | Lactobacillus  + Domperidone | 1. TER | NR |
| Yuan 2011 | 76(20:36:20) | 1~12 | 0.5~24mo | 《Zhu Futang Practical Pediatrics》 《Criteria for Diagnosis and Curative Effect of TCM Diseases》 | NR | TG1: HM TG2: HM + CG | Probiotics (Bifido Triple Viable Bacteria) | 1. TER 2. TCM symptom score | NR |
| Yuan 2019 | 80(40:40) | TG: 6.8 ± 0.48 CG: 6.78 ± 0.39 | TG: 13.02 ± 0.72mo CG: 13.15 ± 0.69mo | 《Zhu Futang Practical Pediatrics》 《Criteria for Diagnosis and Efficacy of TCM Diseases and Syndromes》 《Pediatrics of Chinese Medicine》 | food accumulation | HM | Domperidone | 1. TER 2. Onset time of effective treatment 3. TER for symptom | NR |
| Yuan 2021 | 108(54:54) | TG: 6.28 ± 1.52 CG: 5.84 ± 1.35 | TG: 5.83 ± 1.37mo CG: 6.06 ± 1.51mo | 《Zhu Futang Practical Pediatrics》 《Guiding Principles for Clinical Research of New Chinese Medicines》 | spleen-stomach yin deficiency | HM + CG | Probiotics  (Bacillus Subtilis Dual Live Bacteria) | 1. TER 2. TCM symptom score 3. Motilin (ng/L)  4. Gastrin (pg/ml)  5. Somatostatin (ng/L)  6. Ca2+ (mmol/L)  7. Zn2+ (umol/L)  8. Recurrence rate | NR |
| Zeng 2010 | 56(28:28)→54(28:26) | 1~6 | ≥3mo | 《Zhu Futang Practical Pediatrics》 《Pediatrics of Chinese Medicine》 | liver depression and spleen deficiency | HM | Multi-enzyme | 1. TER 2. Main symptom (grade, %) 3. Secondary symptom (grade, %) 4. TER (secondary symptom) 5. Weight gain (range) | NR |
| Zeng 2012 | 60(30:30) | 1~6 | 15-40d | 《Pediatrics of Chinese Medicine》 《Clinical Treatment of Pediatric Disease with Traditional Chinese Medicine》 | NR | HM + CG | Multi-enzyme + Vitamin B | 1. TER | NR |
| Zhan 2019 | 100(50:50) | TG: 3.60 ± 0.53 CG: 3.51 ± 0.60 | TG: 7.93 ± 1.31mo CG: 8.12 ± 1.23mo | 《Practical Pediatrics》 《Guidelines for Diagnosis and Treatment of Common Diseases in Pediatrics of Traditional Chinese Medicine》 | spleen failing in transportation | HM + CG | Bacillus Licheniformis + Pediatric Compound Lysine | 1. TER 2. TCM symptom score | NR |
| Zhang 2004 | 106(56:50) | TG: 4.3 CG: 4.5 | TG: 3.1mo CG: 3.2mo | 《Criteria for Diagnosis and Efficacy of TCM Diseases and Syndromes》 | NR | HM | Zinc Gluconate | 1. TER | NR |
| Zhang 2005 | 128(88:40) | TG: 1~8 CG: 1~8 | TG: 2~10mo CG: 2~10mo | 《Pediatrics of Chinese Medicine》 | NR | HM + CG | Zinc Gluconate | 1. TER 2. Average effective time | None |
| Zhang 2006 | 80(40:40) | 4.96 | 1mo~5yr | 《Guiding Principles for Clinical Research of New Chinese Medicines》 | NR | HM | Zinc Calcium Gluconate | 1. TER 2. Food intake loss 3. Weight loss 4. TCM symptom score 5. Serum Zn (by age) | NR |
| Zhang 2007 | 80(40:40) | TG: 4.2 CG: 4.1 | TG: 8mo CG: 10.5mo | 《Criteria for Diagnosis and Efficacy of TCM Diseases and Syndromes》 | NR | HM | Vitamin B Complex  + Lactic Acid Bacteria, etc | 1. TER | NR |
| Zhang 2009 | 67(35:32) | TG: 5.6 CG: 4.8 | TG: 7mo CG: 6mo | 《Criteria for Diagnosis and Efficacy of TCM Diseases and Syndromes》 《Standards for Diagnosis and Treatment of Infantile Anorexia》 | NR | HM | Zinc Gluconate | 1. Urine amylase activity (u) 2. Urine d-xylose metabolism rate (%) | NR |
| Zhang 2011 | 128(64:64) | 1~14 | ≥3mo | 《Zhu Futang Practical Pediatrics》 | NR | HM + CG | Probiotics (Bifidobacterium Lactobacillus Triple Live Bacteria) | 1. TER | None |
| Zhang 2013a | 100(60:40) | TG: 5.4 CG: 5.1 | TG: 6mo CG: 5mo | 《Guiding Principles for Clinical Research of New Chinese Medicines》 | NR | HM + CG | Zinc Sulfate | 1. TER | NR |
| Zhang 2013b | 120(80:40) | TG: 3.88 CG: 3.85 | TG: 6mo CG: 5.5mo | 《Criteria for Diagnosis and Efficacy of TCM Diseases and Syndromes》 《Zhu Futang Practical Pediatrics》 | NR | HM | Zinc Gluconate  + Vitamin B, B6, D + Niacin + L-lysine Hydrochloride + Ferrous Fumarate + Calcium Hydrogen phosphate | 1. TER | NR |
| Zhang 2014a | 66(40:26) | 2~10 | ≥2wk | 《Practical Pediatrics》 《Department of Medical Administration, State Administration of Traditional Chinese Medicine》 | NR | HM | Calcium Zinc Gluconate | 1. TER | NR |
| Zhang 2014b | 80(40:40) | TG: 8.6 ± 1.2 CG: 8.5 ± 1.3 | TG: 1.1 ± 0.3yr CG: 1.2 ± 0.4yr | 《Standards for Diagnosis and Treatment of Infantile Anorexia》 | NR | HM + CG | Zinc Gluconate or Dry Yeast flakes  + Domperidone (if necessary) | 1. Total treatment time (d) 2. Clinical symptom improvement time (d) 3. TER | None |
| Zhang 2015 | 40(21:19) | TG: 4.2 ± 1.1 CG: 4.4 ± 1.3 | TG: 1.1 ± 0.39mo CG: 1.1 ± 0.96mo | 《Zhu Futang Practical Pediatrics》 《Pediatrics of Chinese Medicine》 | spleen failing in transportation | HM + CG | Zinc Gluconate | 1. TER 2. TCM symptom score  3. Weight gain (kg) 4. Serum Zn (ug/dl) 5. Hemoglobin (g/L) | NR |
| Zhang 2019 | 70(35:35)→66(33:33) | 1~14 | ≥1mo | 《Zhu Futang Practical Pediatrics》 《Technical Guidelines for Clinical Trial Design and Evaluation of New Chinese Medicines for Children with Anorexia》 《Guidelines for Diagnosis and Treatment of Common Diseases in Pediatrics of Traditional Chinese Medicine》 | spleen deficiency with effulgent liver | HM | Probiotics  (Quadruple Bifidobacterium Live Bacteria) | 1. TER 2. TCM symptom score  3. TER (TCM symptom score) 4. Recurrence rate (after 4 wk) 5. Serum NPY (no unit) | NR |
| Zhang 2020 | 86(43:43) | TG: 3.02 ± 1.12 CG: 2.85 ± 1.06 | NR | 《Practical Pediatrics》 《Criteria for Diagnosis and Curative Effect of TCM Diseases》 | NR | HM | Cisapride  + Zinc Gluconate  + Probiotics  (Quadruple Bifidobacterium Live Bacteria) | 1. TER | NR |
| Zhao 2004 | 114(68:46) | 2~12 | >1mo | 《Criteria for Diagnosis and Curative Effect of TCM Diseases》 | NR | HM | Multi-enzyme + Vitamin B6  + Zinc Citrate | 1. TER | NR |
| Zhao 2012 | 144(72:72)→137(70:67) | NR | NR | 《Practical Pediatrics》 《Pediatrics of Chinese Medicine》 | spleen failing in transportation | HM | Placebo | 1. TER (Anorexia) 2. TER (Food intake) 3. TER 4. TER (TCM symptom score) | N.S TG: 6 CG: 9 |
| Zhao 2015 | 50(25:25) | TG: 4.2 ± 1.7 CG: 4.5 ± 1.8 | TG: 8.4 ± 0.1mo CG: 8.4 ± 0.2mo | 《Practical Pediatrics》 《Guiding Principles for Clinical Research of New Chinese Medicines》 | spleen failing in transportation | HM | Zinc Gluconate | 1. TER | None |
| Zhao 2020a | 146(73:73) | TG: 5.37 ± 2.25 CG: 5.63 ± 2.12 | NR | 《Pediatrics》 | NR | HM | Probiotics  (Bacillus Subtilis Dual Live Bacteria) | 1. TER 2. Food intake symptom score 3. NPY (ng/L) 4. Serum gastrin (ng/L) | NR |
| Zhao 2020b | 99(50:49) | TG: 4.01 ± 0.43 CG: 3.62 ± 0.82 | TG: 10.48 ± 2.14mo CG: 9.98 ± 2.71mo | 《Zhu Futang Practical Pediatrics》 《Guiding Principles for Clinical Research of New Chinese Medicines》 | spleen-stomach qi deficiency | HM + CG | Multi-enzyme | 1. TER 2. BMI 3. TCM symptom score 4. Serum NPY (ng/L) 5. Serum β-EP (pmol/L) | NR |
| Zheng 2012 | 181(NR)→176(55:63:58) | TG1: 4~13 TG2: 3~14 CG: 3~13 | NR | 《Guiding Principles for Clinical Research of New Chinese Medicines》 《Pediatrics of Chinese Medicine》 | spleen-stomach qi deficiency | TG1: HM1 TG2: HM2 | Lifestyle modification | 1. TER | NR |
| Zhong 2020 | 80(40:40) | TG: 2.79 ± 0.51 CG: 2.61 ± 0.57 | TG: 1.25 ± 0.89mo CG: 1.32 ± 0.26mo | 《Criteria for Diagnosis and Efficacy of TCM Diseases and Syndromes》 | spleen failing in transportation | HM + CG | Routine care  (rest, anti-allergic, vitamin supplementation) + Probiotics (Saccharomyces Boulardii) | 1. TCM symptom score | NR |
| Zhou 2005 | 46(25:21) | 3.5 | 1mo~3yr | 《Criteria for Diagnosis and Efficacy of TCM Diseases and Syndromes》 | NR | HM | Probiotics (Bacillus Subtilis Dual Live Bacteria) + Multi-vitamin | 1. TER 2. TCM symptom score 3. RBC count 4. Hemoglobin (g/L) | NR |
| Zhou 2012 | 86(43:43) | TG: 4.2 ± 0.7 CG: 4.4 ± 0.5 | TG: 3.8 ± 1.1mo CG: 3.9 ± 0.8mo | 《Evaluation Criteria for Efficacy of Anorexia in Children》 | NR | HM | Zinc Gluconate | 1. TER 2. Recurrence rate | NR |
| Zhou 2015 | 80(40:40) | TG: 3.4 ± 1.2 CG: 3.3 ± 1.4 | TG: 11.2 ± 3.6mo CG: 10.8 ± 3.3mo | 《Guiding Principles for Clinical Research of New Chinese Medicines》 《Criteria for Diagnosis and Efficacy of TCM Diseases and Syndromes》 | spleen deficiency with effulgent liver | HM + CG | Lactobacillus + Zinc Sulfate syrup  + Iron-containing preparations  (Ferric Amine Citrate)  + Vitamin A, B1, B2, etc. | 1. TER 2. TCM symptom score 3. Serum somatotropin-releasing hormone (ng/mL) 4. Serum leptin (ng/mL) | None |
| Zhou 2018 | 60(30:30) | 1~14 | NR | 《Zhu Futang Practical Pediatrics》 《Criteria for Diagnosis and Curative Effect of TCM Diseases》 《Guiding Principles for Clinical Research of New Chinese Medicines》 | spleen yin deficiency | HM | Zinc Gluconate  + Probiotics (Bifidobacteria Triple Viable Bacteria) | 1. TER 2. TCM symptom score 3. TER (TCM symptom score) | NR |
| Zhou 2020 | 82(41:41) | TG: 4.27 ± 0.97 CG: 4.21 ± 0.84 | TG: 4.17 ± 1.13wk CG: 3.84 ± 1.27wk | 《Differential Diagnosis of Pediatric Symptoms》 《TCM Diagnosis and Treatment Routine》 | NR | HM + CG | Zinc Gluconate | 1. TCM symptom score 2. Serum Leptin (ug/L) 3. Serum NPY (ug/L) 4. Serum Gastrin (pg/mg) 5. Serum Neurotensin (ng/L) | NR |
| Zhu 2000 | 72(48:24) | TG: 3.5 CG: 4 | TG: 6mo CG: 7mo | 《Criteria for Diagnosis and Curative Effect of TCM Diseases》 | spleen-stomach disharmony | HM | Yeast tablet | 1. TER | NR |
| Zhu 2002 | 72(39:33) | TG: 1.5~13 CG: 1.5~13 | TG: 2mo~5yr CG: 2mo~5yr | 《Criteria for Diagnosis and Curative Effect of TCM Diseases》 | NR | HM | Zinc Gluconate | 1. TER 2. Hemoglobin (g/L) | NR |
| Zhu 2011 | 120(86:34) | NR | NR | 《Criteria for Diagnosis and Curative Effect of TCM Diseases》 | NR | HM | Lactase  + Vitamin B1, etc | 1. TER | NR |
| Zhu 2018 | 78(39:39) | TG: 4.03 ± 1.11 CG: 3.67 ± 0.98 | TG: 2.36 ± 1.73mo CG: 2.74 ± 1.65mo | 《Zhu Futang Practical Pediatrics》 | NR | HM + CG | Clostridium Butyricum + Zinc + Dietary adjustment | 1. TER 2. Weight (kg) 3. Appetite recovery time (d) 4. Abdominal pain and bloating disappear time (d) | NR |
| Zou 2014 | 60(30:30) | TG: 4.6 ± 1.0 CG: 4.8 ± 1.0 | NR | 《Zhu Futang Practical Pediatrics》 《Pediatrics of Chinese Medicine》 | NR | HM | Multi-enzyme  + Zinc Gluconate | 1. TER | NR |
| Zou 2016 | 78(40:38) | TG: 4.2 ± 1.8 CG: 4.4 ± 1.3 | TG: 7.5 ± 1.1mo CG: 7.0 ± 1.4mo | 《Practical Pediatrics》 《Criteria for Diagnosis and Curative Effect of TCM Diseases》 《Guiding Principles for Clinical Research of New Chinese Medicines》 | NR | HM + CG | Zinc Selenium | 1. TER 2. Weight (kg) | None |

Abbreviations. ALT, alanine aminotransferase; BMI, body mass index; BUN, blood urea nitrogen; CCK, cholecystokinin-octapeptide; CG, control group; HM, herbal medicine; NPY, neuropeptide Y; NR, not recorded; N.S, non-significant; PedsQL, pediatric quality of life inventory; RBC, red blood cell; TCM, traditional Chinese medicine; TER, total effective rate; TG, treatment group; VIP, vasoactive intestinal peptide.
