# Supplementary material for: Herbal Medicine for the Treatment of Anorexia in Children: A Systematic Review and Meta-Analysis
Source: Front Pharmacol. 2022 Apr 1;13:839668. doi: 10.3389/fphar.2022.839668 (PMC9012502; doi:10.3389/fphar.2022.839668)
Supplement: Supplementary file 5 [file Table4.DOCX]

Supplement 4. Details of herbal medicine used in each study

| **Study ID** | **Herbal medicine name** | **Dosage form** | **Main herb components (per day)** | **Additional components (per day)** | **Manufacturer** | **Administration period** | **Follow-up period** |
| --- | --- | --- | --- | --- | --- | --- | --- |
| Ao 2017 | Xiaoer Yanshi decoction | Decoction | Dioscorea batatas Dacne. [Dioscoreaceae; Dioscoreae Rhizoma] 8 g, Gallus gallus var. domesticus Brisson [Phasianidae; Galli Stomachichum Corium], Crataegus pinnatifida Bge [Rosaceae; Crataegii Fructus], Dolichos lablab L. [Leguminosae; Lablab Semen] 6 g, Adenophora triphylla var. japonica Hara [Campanulaceae; Adenophorae Radix], Prunus mume Sieb. et Zucc [Rosaceae; Mume Fructus], Paeonia lactiflora Pall. [Paeoniaceae; Paeoniae Radix Alba] 5 g, Glycyrrhiza uralensis Fisch. [Leguminosae; Glycyrrhizae Radix] 3 g | None | Not applicable | 12 weeks | None |
| Bai 2005 | Xiaoer Yanshi decoction | Decoction | Crataegus pinnatifida Bge [Rosaceae; Crataegii Fructus], Triticum aestivum L. [Gramineae; Massa Medicata Fermentata], Hordeum vulgare L. [Gramineae; Hordei Fructus Germiniatus], Corydalis ternata Nakai [Papaveraceae; Corydalis (Tuber) Rhizoma], Paeonia lactiflora Pall. [Paeoniaceae; Paeoniae Radix Alba], Codonopsis pilosulae (Fr.) Nannf. [Campanulaceae; Codonopsis Pilosulae Radix], Poria cocos (Schw.) Wolf [Polyporaceae; Poria(Hoelen)] 8 g, Atractylodes macrocepha-la Koidz [Asteraceae; Atractylodis Rhizoma Alba], Raphanus sativus var. hortensis for. acanthiformis Makino [Brassicaceae; Raphani Semen], Aucklandia lappa Decne [Asteraceae; Aucklandiae Radix], Glycyrrhiza uralensis Fisch. [Leguminosae; Glycyrrhizae Radix] 5 g, Amomum villosum Lour. [Zingiberaceae; Amomi Fuctus] 3 g | None | Not applicable | TG: mean 21 days CG: mean 32 days | None |
| Cai 2003 | Rénshēn diào pí sàn | Granule | Panax ginseng C. A. Mey. [Araliaceae; Ginseng Radix], Poria cocos (Schw.) Wolf [Polyporaceae; Poria(Hoelen)], Dioscorea batatas Dacne. [Dioscoreaceae; Dioscoreae Rhizoma], Nelumbo nucifera Gaertner [Nymphaceae; Nelumbinis Semen], Euryale ferox Salisb. [Nymphaceae; Euryales Semen], Hordeum vulgare L. [Gramineae; Hordei Fructus Germiniatus], Gallus gallus var. domesticus Brisson [Phasianidae; Galli Stomachichum Corium], Dendrobium loddigesii Rolfe. [Orchidaceae; Denbrobii Herba], Atractylodes macrocepha-la Koidz [Asteraceae; Atractylodis Rhizoma Alba], Cocculus sarmentosus (Lour.) Diels. | None | Not applicable | 28-42 days | None |
| Cai 2017 | Xiāo shí jiàn pí héjì | Decoction | Atractylodes macrocepha-la Koidz [Asteraceae; Atractylodis Rhizoma Alba], Amomum villosum Lour. [Zingiberaceae; Amomi Fuctus], Dioscorea batatas Dacne. [Dioscoreaceae; Dioscoreae Rhizoma], Magnolia officinalis Rehder et Wilson [Magnoliaceae; Magnoliae Cortex], Gallus gallus var. domesticus Brisson [Phasianidae; Galli Stomachichum Corium] 14 g, Aucklandia lappa Decne [Asteraceae; Aucklandiae Radix], Forsythia suspensa (Thunb.) Vahl [Oleaceae; Forsythiae Fructus], Citrus unshiu Markovich [Rutaceae; Citri Unshius Pericarpium], Poncirus trifoliata Rafin. [Rutaceae; Aurantii Immaturus Fructus], Raphanus sativus var. hortensis for. acanthiformis Makino [Brassicaceae; Raphani Semen], Crataegus pinnatifida Bge [Rosaceae; Crataegii Fructus], Triticum aestivum L. [Gramineae; Massa Medicata Fermentata], Hordeum vulgare L. [Gramineae; Hordei Fructus Germiniatus], Pinellia ternata (Thunb.) Breit. [Araceae; Pinelliae Rhizoma] 9 g | None | Not applicable | 4 weeks | 1, 3, 6 month |
| Cai 2019 | Qū mài zhǐ shù tāng | Decoction | Atractylodes lancea (Thunb.) DC. [Asteraceae; Atractylodis Rhizoma] 15 g, Poncirus trifoliata Rafin. [Rutaceae; Aurantii Immaturus Fructus], Triticum aestivum L. [Gramineae; Massa Medicata Fermentata], Hordeum vulgare L. [Gramineae; Hordei Fructus Germiniatus] 10 g | - Abdominal distention, abdominal pain: Aucklandia lappa Decne [Asteraceae; Aucklandiae Radix], Citrus unshiu Markovich [Rutaceae; Citri Unshius Pericarpium] - Sloppy stool: Dioscorea batatas Dacne. [Dioscoreaceae; Dioscoreae Rhizoma], Pueraria thunbergiana Benth. [Leguminosae; Puerariae Radix]  - Constipation: Cannabis sativa L. [Cannabinaceae; Cannabis Fructus], Raphanus sativus var. hortensis for. acanthiformis Makino [Brassicaceae; Raphani Semen]  - Night anxiety, profuse sweating: Cryptotympana pustulata Fabricius [Cicadidae; Cicadae Periostracum], Zizyphus jujuba Mill [Rhamnaceae; Zizyphi Spinosae Semen] | Not applicable | 30 days | None |
| Chen 2001 | Jiàn pí kāi wèi tāng | Decoction | Poria cocos (Schw.) Wolf [Polyporaceae; Poria(Hoelen)], Astragalus membranaceus Bunge [Leguminosae; Astragali Radix], Crataegus pinnatifida Bge [Rosaceae; Crataegii Fructus] 10~15 g, Atractylodes lancea (Thunb.) DC. [Asteraceae; Atractylodis Rhizoma], Gallus gallus var. domesticus Brisson [Phasianidae; Galli Stomachichum Corium] 6~10 g, Citrus reticulata Blanco [Rutaceae; Citri Rubrum Exocarpium] 4~10 g, Scolopendra subspinipes mutilans L. Koch [Scolopendridae; Scolopendra Corpus] 1 | - Dampness imbalance: Agastache rugosa (Fisch. et Meyer) O. Kuntze [Labiatae; Agastachis Herba], Eupatorium chinese for. tripartitum H. Hara [Asteraceae; Eupatorii Herba] - Obvious abdominal distension: Raphanus sativus var. hortensis for. acanthiformis Makino [Brassicaceae; Raphani Semen], Citrus aurantium L. [Rutaceae; Aurantii Fructus Pericarpium] - Sloppy stool: Crataegus pinnatifida Bge [Rosaceae; Crataegii Fructus] - Obvious yin deficiency: Trichosanthes kirilowii Maxim. [Cucurbitaceae; Trichosanthis Fructus], Dendrobium loddigesii Rolfe. [Orchidaceae; Denbrobii Herba], Picrorrhiza scrophulariiflora Pennell [Scrophulariaceae; Picrorrhozae Rhizoma] - Hyperhidrosis: Triticum aestivum L. [Gramineae; Tritici Cimmatri Semen], Ostrea gigas Thunb. [Ostreidae; Ostreae Concha] | Not applicable | 7 days | None |
| Chen 2002 | Lè shí héjì | Decoction | Paederia scandens var. scandens (Lour.) Merr. [Rubiaceae; Paederiae Herba] 15 g, Codonopsis pilosulae (Fr.) Nannf. [Campanulaceae; Codonopsis Pilosulae Radix], Atractylodes macrocepha-la Koidz [Asteraceae; Atractylodis Rhizoma Alba], Lindera srtychnifolium [Lauraceae; Linderae Radix], Curcuma zedoaria Rocs. [Zingiberaceae; Zedoariae Rhizoma], Dendrobium loddigesii Rolfe. [Orchidaceae; Denbrobii Herba], Crataegus pinnatifida Bge [Rosaceae; Crataegii Fructus], Raphanus sativus var. hortensis for. acanthiformis Makino [Brassicaceae; Raphani Semen], Triticum aestivum L. [Gramineae; Massa Medicata Fermentata] 10 g, Aucklandia lappa Decne [Asteraceae; Aucklandiae Radix] 6 g | None | Sānjiǔ jítuán | 2 weeks | None |
| Chen 2008 | Kaiwei decoction | Decoction | Atractylodes macrocepha-la Koidz [Asteraceae; Atractylodis Rhizoma Alba], Atractylodes lancea (Thunb.) DC. [Asteraceae; Atractylodis Rhizoma], Poria cocos (Schw.) Wolf [Polyporaceae; Poria(Hoelen)], Curcuma zedoaria Rocs. [Zingiberaceae; Zedoariae Rhizoma], Gallus gallus var. domesticus Brisson [Phasianidae; Galli Stomachichum Corium], Oryza sativa L. [Gramineae; Oryzae Fructus Germinatus], Coix lachryma-jobi var. ma-yeun (Roman.) Stapf [Gramineae; Coicis Semen] 10 g, Poncirus trifoliata Rafin. [Rutaceae; Aurantii Immaturus Fructus], Citrus unshiu Markovich [Rutaceae; Citri Unshius Pericarpium]6 g | None | Not applicable | 3 weeks | 2 week |
| Chen 2015a | Xingpi Yanger granule | Granule | Gerbera piloselloides (Linn. ) Cass., Pittosporum glabratum Lindl., Emilia sonchifolia (L.) DC., Valeriana jatamansi Jones | None | NR | 4 weeks | None |
| Chen 2015b | Yigong powder | Decoction | Codonopsis pilosulae (Fr.) Nannf. [Campanulaceae; Codonopsis Pilosulae Radix], Atractylodes macrocepha-la Koidz [Asteraceae; Atractylodis Rhizoma Alba], Poria cocos (Schw.) Wolf [Polyporaceae; Poria(Hoelen)] 10 g, Citrus unshiu Markovich [Rutaceae; Citri Unshius Pericarpium]8 g, Gallus gallus var. domesticus Brisson [Phasianidae; Galli Stomachichum Corium], Hordeum vulgare L. [Gramineae; Hordei Fructus Germiniatus], Glycyrrhiza uralensis Fisch. [Leguminosae; Glycyrrhizae Radix] 5 g | - Abdominal distension: Magnolia officinalis Rehder et Wilson [Magnoliaceae; Magnoliae Cortex], Aucklandia lappa Decne [Asteraceae; Aucklandiae Radix]  - Sloopy or Undigested stool: Amomum villosum Lour. [Zingiberaceae; Amomi Fuctus], Scutellaria baicalensis Georgi [Labiatae; Scutellariae Radix], Dolichos lablab L. [Leguminosae; Lablab Semen] | Not applicable | 30 days | None |
| Chen 2015c | Jianpi decoction | Decoction | Huò pú, Atractylodes macrocepha-la Koidz [Asteraceae; Atractylodis Rhizoma Alba] 15 g, Codonopsis pilosulae (Fr.) Nannf. [Campanulaceae; Codonopsis Pilosulae Radix], Citrus unshiu Markovich [Rutaceae; Citri Unshius Pericarpium], Triticum aestivum L. [Gramineae; Massa Medicata Fermentata], Crataegus pinnatifida Bge [Rosaceae; Crataegii Fructus] 10 g, Citrus aurantium L. [Rutaceae; Aurantii Fructus Pericarpium] 8 g, Glycyrrhiza uralensis Fisch. [Leguminosae; Glycyrrhizae Radix] 3 g | None | Not applicable | 4 weeks | None |
| Chen 2016 | Qi Pi Yin | Decoction | Dioscorea batatas Dacne. [Dioscoreaceae; Dioscoreae Rhizoma], Atractylodes macrocepha-la Koidz [Asteraceae; Atractylodis Rhizoma Alba], Agastache rugosa (Fisch. et Meyer) O. Kuntze [Labiatae; Agastachis Herba], Crataegus pinnatifida Bge [Rosaceae; Crataegii Fructus], Triticum aestivum L. [Gramineae; Massa Medicata Fermentata], Hordeum vulgare L. [Gramineae; Hordei Fructus Germiniatus], Citrus unshiu Markovich [Rutaceae; Citri Unshius Pericarpium], Gallus gallus var. domesticus Brisson [Phasianidae; Galli Stomachichum Corium] 10 g, Pinellia ternata (Thunb.) Breit. [Araceae; Pinelliae Rhizoma], Poria cocos (Schw.) Wolf [Polyporaceae; Poria(Hoelen)] 5 g, Aucklandia lappa Decne [Asteraceae; Aucklandiae Radix] 3 g | - Vomiting: Phyllostachys nigra var. henonis (Bean.) Stapf [Gramineae; Bambusae Caulis In Taeniam] 10 g - Abdominal distension: Amomum villosum Lour. [Zingiberaceae; Amomi Fuctus] 5 g  - Dampness predominance: Atractylodes lancea (Thunb.) DC. [Asteraceae; Atractylodis Rhizoma] 10 g - Spleen-stomach heat: Coptis deltoidea C.Y. Cheng et Hsiao [Ranunculaceae; Coptidis Rhizoma] 2 g, Gentiana scabra Bunge [Gentianaceae; Gentianae Radix] 3 g - Spleen-stomach qi deficiency: Codonopsis pilosulae (Fr.) Nannf. [Campanulaceae; Codonopsis Pilosulae Radix] 8 g - Stomach yang deficiency: Polygonatum odoratum var. pluriflorum Ohwi [Liliaceae; Polygonati Dodrati Rhizoma] 6 g - Spontaneous sweating: Astragalus membranaceus Bunge [Leguminosae; Astragali Radix] 10 g, Saposhnikovia divaricata Schiskin [Apiaceae; Saposhnikovia Radix] 5 g - Night sweating: Triticum aestivum L. [Gramineae; Tritici Cimmatri Semen] 12 g - Depressed mood: Curcuma aromatica Salisb. [Zingiberaceae; Curcumae Radix] 10 g, Selaginella tamariscina (Beauv.) Spring [Selaginellaceae; Selaginelliae Herba] 8 g | Not applicable | 1 month | None |
| Chen 2017 | Feier mixture | Decoction | Pinellia ternata (Thunb.) Breit. [Araceae; Pinelliae Rhizoma], Hordeum vulgare L. [Gramineae; Hordei Fructus Germiniatus] 15 g, Agastache rugosa (Fisch. et Meyer) O. Kuntze [Labiatae; Agastachis Herba], Poria cocos (Schw.) Wolf [Polyporaceae; Poria(Hoelen)], Crataegus pinnatifida Bge [Rosaceae; Crataegii Fructus], Scutellaria baicalensis Georgi [Labiatae; Scutellariae Radix], Citrus unshiu Markovich [Rutaceae; Citri Unshius Pericarpium], Atractylodes lancea (Thunb.) DC. [Asteraceae; Atractylodis Rhizoma], Triticum aestivum L. [Gramineae; Massa Medicata Fermentata] 10 g, Magnolia officinalis Rehder et Wilson [Magnoliaceae; Magnoliae Cortex] 6 g, Glycyrrhiza uralensis Fisch. [Leguminosae; Glycyrrhizae Radix] 3 g | None | Not applicable | 4 weeks | None |
| Chen 2018a | Xiaoer Yanshi decoction | Decoction | Coix lachryma-jobi var. ma-yeun (Roman.) Stapf [Gramineae; Coicis Semen], Hordeum vulgare L. [Gramineae; Hordei Fructus Germiniatus] 15 g, Poria cocos (Schw.) Wolf [Polyporaceae; Poria(Hoelen)] 12 g, Triticum aestivum L. [Gramineae; Massa Medicata Fermentata], Astragalus membranaceus Bunge [Leguminosae; Astragali Radix] 10 g, Codonopsis pilosulae (Fr.) Nannf. [Campanulaceae; Codonopsis Pilosulae Radix], Atractylodes macrocepha-la Koidz [Asteraceae; Atractylodis Rhizoma Alba], Selaginella tamariscina (Beauv.) Spring [Selaginellaceae; Selaginelliae Herba], Forsythia suspensa (Thunb.) Vahl [Oleaceae; Forsythiae Fructus] 9 g, Citrus unshiu Markovich [Rutaceae; Citri Unshius Pericarpium], Curcuma aromatica Salisb. [Zingiberaceae; Curcumae Radix], Gallus gallus var. domesticus Brisson [Phasianidae; Galli Stomachichum Corium] 6 g | None | Not applicable | 1 month | None |
| Chen 2018b | Triangular traditional Chinese medicine | Granule | - Qi deficiency: Astragalus membranaceus Bunge [Leguminosae; Astragali Radix], Atractylodes macrocepha-la Koidz [Asteraceae; Atractylodis Rhizoma Alba], Saposhnikovia divaricata Schiskin [Apiaceae; Saposhnikovia Radix] 5 g - Yin deficiency: Atractylodes macrocepha-la Koidz [Asteraceae; Atractylodis Rhizoma Alba] 5 g, Panax ginseng C. A. Mey. [Araliaceae; Ginseng Radix] 2.5 g, Zingiber officinale Rosc. [Zingiberaceae; Zingiberis Rhizoma Siccus] 1.5 g - Yang deficiency: Scrophularia buergeriana Miq. [Scrophulariaceae; Scrophulariae Radix], Rhemannia glutinosa (Gaertner) Liboschitz [Scrophulariaceae; Rehmanniae Radix], Liriope platyphylla Wang et Tang [Liliaceae; Liriopes Radix] 5 g - Phlegm-dampness: Poria cocos (Schw.) Wolf [Polyporaceae; Poria(Hoelen)] 5 g, Pinellia ternata (Thunb.) Breit. [Araceae; Pinelliae Rhizoma] 4.5 g, Citrus unshiu Markovich [Rutaceae; Citri Unshius Pericarpium]3 g | None | Not applicable | 28 days | 4 week |
| Chen 2018c | Jiàn pí hé wèi tāng | Decoction | Atractylodes macrocepha-la Koidz [Asteraceae; Atractylodis Rhizoma Alba] 15 g, Codonopsis pilosulae (Fr.) Nannf. [Campanulaceae; Codonopsis Pilosulae Radix], Poria cocos (Schw.) Wolf [Polyporaceae; Poria(Hoelen)], Crataegus pinnatifida Bge [Rosaceae; Crataegii Fructus], Amomum villosum Lour. [Zingiberaceae; Amomi Fuctus], Bupleurum falcatum Linne [Apiaceae; Bupleuri Radix] 10 g, Glycyrrhiza uralensis Fisch. [Leguminosae; Glycyrrhizae Radix], Citrus unshiu Markovich [Rutaceae; Citri Unshius Pericarpium], Hordeum vulgare L. [Gramineae; Hordei Fructus Germiniatus], Dioscorea batatas Dacne. [Dioscoreaceae; Dioscoreae Rhizoma] 6 g | - Severe yin deficiency: Liriope platyphylla Wang et Tang [Liliaceae; Liriopes Radix], Dendrobium loddigesii Rolfe. [Orchidaceae; Denbrobii Herba] 10 g - Severe qi deficiency: Astragalus membranaceus Bunge [Leguminosae; Astragali Radix], Panax ginseng C. A. Mey. [Araliaceae; Ginseng Radix] 10 g - Night sweating: Schizandra chinensis (Turcz.) Baill. [Magnoliaceae; Schizandrae Fructus] 10 g | Not applicable | 28 days | None |
| Chen 2019 | Shugan Jianpi decoction | Decoction | Bupleurum falcatum Linne [Apiaceae; Bupleuri Radix], Paeonia lactiflora Pall. [Paeoniaceae; Paeoniae Radix Alba], Citrus unshiu Markovich [Rutaceae; Citri Unshius Pericarpium], Citrus aurantium L. [Rutaceae; Aurantii Fructus Pericarpium] 10 g, Curcuma aromatica Salisb. [Zingiberaceae; Curcumae Radix] 12 g, Codonopsis pilosulae (Fr.) Nannf. [Campanulaceae; Codonopsis Pilosulae Radix], Atractylodes macrocepha-la Koidz [Asteraceae; Atractylodis Rhizoma Alba], Crataegus pinnatifida Bge [Rosaceae; Crataegii Fructus] 15 g, Amomum villosum Lour. [Zingiberaceae; Amomi Fuctus], Glycyrrhiza uralensis Fisch. [Leguminosae; Glycyrrhizae Radix] 6 g | - Obvious abdominal distension: Raphanus sativus var. hortensis for. acanthiformis Makino [Brassicaceae; Raphani Semen], Magnolia officinalis Rehder et Wilson [Magnoliaceae; Magnoliae Cortex] 12 g - Obvious upper abdominal pain: Corydalis ternata Nakai [Papaveraceae; Corydalis (Tuber) Rhizoma], Curcuma longa L. [Zingiberaceae; Curcumae Longae Rhizoma] 10 g -Obvious acid reflux, heartburn: Sepia esculenta Hoyle [Sepiolidae; Sepiae Os] 15 g, Coptis deltoidea C.Y. Cheng et Hsiao [Ranunculaceae; Coptidis Rhizoma] 3 g, Evodia rutaecarpa (Juss.) Benth. [Rutaceae; Evodiae Fructus] 3 g -Obvious belching: Calystegia japonica (Thunb.) Chois. [Convolvulaceae; Calystegiae Flos] 10 g, Magnetite 30 g - Limb weakness、lack of strength、shortage of qi, unwilling to talk: Pseudostellaria augustifolia Y.N.Lee [Caryophyllaceae; Pseudostellariae Radix] 30 g, Poria cocos (Schw.) Wolf [Polyporaceae; Poria(Hoelen)] 15 g - Depressed mood or agitation: Cyperus rotundus L. [Cyperaceae; Cyperi Rhizoma], Albizia julibrissin Durazz. [Leguminosae; Albizziae Cortex], Lilium lancifolium Thunb. [Liliaceae; Lili Bulbus] 10 g | Not applicable | 6 weeks | None |
| Chen 2020a | Yunpi Xiaoshi decoction | Decoction | Atractylodes lancea (Thunb.) DC. [Asteraceae; Atractylodis Rhizoma], Dioscorea batatas Dacne. [Dioscoreaceae; Dioscoreae Rhizoma], Crataegus pinnatifida Bge [Rosaceae; Crataegii Fructus], Triticum aestivum L. [Gramineae; Massa Medicata Fermentata], Hordeum vulgare L. [Gramineae; Hordei Fructus Germiniatus] 10 g, Agastache rugosa (Fisch. et Meyer) O. Kuntze [Labiatae; Agastachis Herba] 9 g, Raphanus sativus var. hortensis for. acanthiformis Makino [Brassicaceae; Raphani Semen], Gallus gallus var. domesticus Brisson [Phasianidae; Galli Stomachichum Corium], Citrus unshiu Markovich [Rutaceae; Citri Unshius Pericarpium], Citrus aurantium L. [Rutaceae; Aurantii Fructus Pericarpium], Amomum villosum Lour. [Zingiberaceae; Amomi Fuctus], Poria cocos (Schw.) Wolf [Polyporaceae; Poria(Hoelen)], Triticum aestivum L. [Gramineae; Massa Medicata Fermentata] 6 g, Glycyrrhiza uralensis Fisch. [Leguminosae; Glycyrrhizae Radix] 3 g | None | Not applicable | 4 weeks | None |
| Chen 2020b | Yànshí líng kēlì | Granule | Glehnia littoralis Fr. Schm. [Apiaceae; Glehniae Radix], Gallus gallus var. domesticus Brisson [Phasianidae; Galli Stomachichum Corium], Triticum aestivum L. [Gramineae; Massa Medicata Fermentata], Hordeum vulgare L. [Gramineae; Hordei Fructus Germiniatus], Crataegus pinnatifida Bge [Rosaceae; Crataegii Fructus] 10 g, Androsace umbellata (Lour.) Merr. [Primulacae; Stellariae Radix], Paeonia lactiflora Pall. [Paeoniaceae; Paeoniae Radix Alba] 8 g, Aucklandia lappa Decne [Asteraceae; Aucklandiae Radix], Curcuma aromatica Salisb. [Zingiberaceae; Curcumae Radix], Selaginella tamariscina (Beauv.) Spring [Selaginellaceae; Selaginelliae Herba], Ziziphus jujuba, Cryptotympana pustulata Fabricius [Cicadidae; Cicadae Periostracum] 6 g, Glycyrrhiza uralensis Fisch. [Leguminosae; Glycyrrhizae Radix] 3 g | - Abdominal pain: Corydalis ternata Nakai [Papaveraceae; Corydalis (Tuber) Rhizoma] 10 g, Citrus aurantium L. [Rutaceae; Aurantii Fructus Pericarpium], Lindera srtychnifolium [Lauraceae; Linderae Radix] 8 g - Constipation: Raphanus sativus var. hortensis for. acanthiformis Makino [Brassicaceae; Raphani Semen], Prunus humilis Bunge [Rosaceae; Pruni Humilis Semen] 10 g - Diarrhea: remove Thread jujube, add Atractylodes lancea (Thunb.) DC. [Asteraceae; Atractylodis Rhizoma] 10 g, Amomum villosum Lour. [Zingiberaceae; Amomi Fuctus], Sesamum indicum L. [Pedaliaceae; Sesami Oleum] 8 g | Nèiménggǔ huì fēng yào yè yǒuxiàn gōngsī | 4 weeks | 6 month |
| Cheng 2019 | Xingpi Yanger granule | Granule | Gerbera piloselloides (Linn. ) Cass., Pittosporum glabratum Lindl., Emilia sonchifolia (L.) DC., Valeriana jatamansi Jones | None | NR | 8 weeks | None |
| Deng 2004 | Yunpi Xiaoshi decoction | Decoction | Poria cocos (Schw.) Wolf [Polyporaceae; Poria(Hoelen)] 10 g, Crataegus pinnatifida Bge [Rosaceae; Crataegii Fructus], Dioscorea batatas Dacne. [Dioscoreaceae; Dioscoreae Rhizoma] 8 g, Atractylodes lancea (Thunb.) DC. [Asteraceae; Atractylodis Rhizoma], Triticum aestivum L. [Gramineae; Massa Medicata Fermentata], Aucklandia lappa Decne [Asteraceae; Aucklandiae Radix], Gallus gallus var. domesticus Brisson [Phasianidae; Galli Stomachichum Corium] 6 g, Glycyrrhiza uralensis Fisch. [Leguminosae; Glycyrrhizae Radix], Citrus unshiu Markovich [Rutaceae; Citri Unshius Pericarpium]3 g | - Liver effulgence: Xiàngyá sī, Dú jiǎo jīn 6 g - Dampness stagnation: Dolichos lablab L. [Leguminosae; Lablab Semen], Coix lachryma-jobi var. ma-yeun (Roman.) Stapf [Gramineae; Coicis Semen] 8 g - Qi deficiency: Pseudostellaria augustifolia Y.N.Lee [Caryophyllaceae; Pseudostellariae Radix], Atractylodes macrocepha-la Koidz [Asteraceae; Atractylodis Rhizoma Alba] 6 g | Not applicable | 2 weeks | None |
| Deng 2018a | Tiaogan Lipi granule | Granule | Atractylodes lancea (Thunb.) DC. [Asteraceae; Atractylodis Rhizoma], Atractylodes macrocepha-la Koidz [Asteraceae; Atractylodis Rhizoma Alba], Bupleurum falcatum Linne [Apiaceae; Bupleuri Radix], Paeonia lactiflora Pall. [Paeoniaceae; Paeoniae Radix Alba], Poria cocos (Schw.) Wolf [Polyporaceae; Poria(Hoelen)], Citrus unshiu Markovich [Rutaceae; Citri Unshius Pericarpium], Astagalus membranaceus Bunge [Leguminosae; Astragali Radix], Dolichos lablab L. [Leguminosae; Lablab Semen], Forsythia suspensa (Thunb.) Vahl [Oleaceae; Forsythiae Fructus], Crataegus pinnatifida Bge [Rosaceae; Crataegii Fructus], Citrus aurantium L. [Rutaceae; Aurantii Fructus Pericarpium], Selaginella tamariscina (Beauv.) Spring [Selaginellaceae; Selaginelliae Herba], Uncaria rhynchophylla (Miq.) Jacks. [Rubiaceae; Uncariae Ramulus cum Uncis] 10 g, Aucklandia lappa Decne [Asteraceae; Aucklandiae Radix] 6 g, Syzygium aromaticum Merr et Perry [Myrtaceac; Syzygii Flos] 3 g | None | Not applicable | 4 weeks | None |
| Deng 2018b | Tiaogan Lipi granule | Granule | Atractylodes lancea (Thunb.) DC. [Asteraceae; Atractylodis Rhizoma], Atractylodes macrocepha-la Koidz [Asteraceae; Atractylodis Rhizoma Alba], Bupleurum falcatum Linne [Apiaceae; Bupleuri Radix], Paeonia lactiflora Pall. [Paeoniaceae; Paeoniae Radix Alba], Poria cocos (Schw.) Wolf [Polyporaceae; Poria(Hoelen)], Citrus unshiu Markovich [Rutaceae; Citri Unshius Pericarpium], Astagalus membranaceus Bunge [Leguminosae; Astragali Radix], Dolichos lablab L. [Leguminosae; Lablab Semen], Forsythia suspensa (Thunb.) Vahl [Oleaceae; Forsythiae Fructus], Crataegus pinnatifida Bge [Rosaceae; Crataegii Fructus], Citrus aurantium L. [Rutaceae; Aurantii Fructus Pericarpium], Selaginella tamariscina (Beauv.) Spring [Selaginellaceae; Selaginelliae Herba], Uncaria rhynchophylla (Miq.) Jacks. [Rubiaceae; Uncariae Ramulus cum Uncis] 10 g, Aucklandia lappa Decne [Asteraceae; Aucklandiae Radix] 6 g, Syzygium aromaticum Merr et Perry [Myrtaceac; Syzygii Flos] 3 g | None | Not applicable | 4 weeks | 1 month |
| Ding 2019 | Xiaoer Yanshi granule | Granule | Crataegus pinnatifida Bge [Rosaceae; Crataegii Fructus], Zingiber officinale Rosc. [Zingiberaceae; Zingiberis Rhizoma Siccus], Panax ginseng C. A. Mey. [Araliaceae; Ginseng Radix], Amomum villosum Lour. [Zingiberaceae; Amomi Fuctus], Dioscorea batatas Dacne. [Dioscoreaceae; Dioscoreae Rhizoma] etc | None | Nèiménggǔ huì fēng yào yè yǒuxiàn gōngsī | 14 days | None |
| Duan 2013 | Yunpi Hewei decoction | Decoction | Poncirus trifoliata Rafin. [Rutaceae; Aurantii Immaturus Fructus], Triticum aestivum L. [Gramineae; Massa Medicata Fermentata], Atractylodes lancea (Thunb.) DC. [Asteraceae; Atractylodis Rhizoma], Amomum villosum Lour. [Zingiberaceae; Amomi Fuctus], Aucklandia lappa Decne [Asteraceae; Aucklandiae Radix], Pseudostellaria augustifolia Y.N.Lee [Caryophyllaceae; Pseudostellariae Radix], Poria cocos (Schw.) Wolf [Polyporaceae; Poria(Hoelen)], Magnolia officinalis Rehder et Wilson [Magnoliaceae; Magnoliae Cortex] 5-8 g, Gallus gallus var. domesticus Brisson [Phasianidae; Galli Stomachichum Corium], Citrus unshiu Markovich [Rutaceae; Citri Unshius Pericarpium]5-15 g, Hordeum vulgare L. [Gramineae; Hordei Fructus Germiniatus] 10-15 g, Chaenomeles sinensis Koehne [Rosaceae; Chaenomelis Fuctus] 5-10 g, Glycyrrhiza uralensis Fisch. [Leguminosae; Glycyrrhizae Radix] 3-6 g | None | Not applicable | 7 days | None |
| Duan 2014 | Yunpi Hewei decoction | Decoction | Hordeum vulgare L. [Gramineae; Hordei Fructus Germiniatus] 10-15 g, Gallus gallus var. domesticus Brisson [Phasianidae; Galli Stomachichum Corium], Citrus unshiu Markovich [Rutaceae; Citri Unshius Pericarpium]5-15 g, Chaenomeles sinensis Koehne [Rosaceae; Chaenomelis Fuctus] 5-10 g, Poncirus trifoliata Rafin. [Rutaceae; Aurantii Immaturus Fructus], Triticum aestivum L. [Gramineae; Massa Medicata Fermentata], Atractylodes lancea (Thunb.) DC. [Asteraceae; Atractylodis Rhizoma], Amomum villosum Lour. [Zingiberaceae; Amomi Fuctus], Aucklandia lappa Decne [Asteraceae; Aucklandiae Radix], Pseudostellaria augustifolia Y.N.Lee [Caryophyllaceae; Pseudostellariae Radix], Magnolia officinalis Rehder et Wilson [Magnoliaceae; Magnoliae Cortex], Poria cocos (Schw.) Wolf [Polyporaceae; Poria(Hoelen)] 5-8 g, Glycyrrhiza uralensis Fisch. [Leguminosae; Glycyrrhizae Radix] 3-6 g | None | Not applicable | 7 days | None |
| Fan 2018 | Sijunzi decoction | Decoction | Crataegus pinnatifida Bge [Rosaceae; Crataegii Fructus], Triticum aestivum L. [Gramineae; Massa Medicata Fermentata], Hordeum vulgare L. [Gramineae; Hordei Fructus Germiniatus] 3-8 g, Codonopsis pilosulae (Fr.) Nannf. [Campanulaceae; Codonopsis Pilosulae Radix], Atractylodes macrocepha-la Koidz [Asteraceae; Atractylodis Rhizoma Alba], Poncirus trifoliata Rafin. [Rutaceae; Aurantii Immaturus Fructus], Gallus gallus var. domesticus Brisson [Phasianidae; Galli Stomachichum Corium] 3-6 g, Poria cocos (Schw.) Wolf [Polyporaceae; Poria(Hoelen)] 3-5 g, Paeonia lactiflora Pall. [Paeoniaceae; Paeoniae Radix Alba] 2-5 g, Citrus unshiu Markovich [Rutaceae; Citri Unshius Pericarpium]2-4 g, Bupleurum falcatum Linne [Apiaceae; Bupleuri Radix], Glycyrrhiza uralensis Fisch. [Leguminosae; Glycyrrhizae Radix] 1-3 g | - Hiccups frequently: Zingiber officinale Rosc. [Zingiberaceae; Zingiberis Rhizoma Recens] 3-5 g, Raphanus sativus var. hortensis for. acanthiformis Makino [Brassicaceae; Raphani Semen] 3-6 g - Diarrhea: Amomum villosum Lour. [Zingiberaceae; Amomi Fuctus] 1-3 g, Zingiber officinale Rosc. [Zingiberaceae; Zingiberis Rhizoma Siccus] 3-5 g - Constipation: Rheum palmatum L. [Polygonaceae; Rhei Rhizoma] 2-4 g - Abdominal pain with parasitic disease: Quisqualis indica L. [Combretaceae; Quisqualis Fructus] 1-3 g Aleo vera L. [Liliaceae; Aloe] 1 g - Effulgent liver fire, thirst, red tongue: Scutellaria baicalensis Georgi [Labiatae; Scutellariae Radix], Gardenia jasminoides var. grandiflora (Lour.) Nakai [Rubiaceae; Gardeniae Fructus] 3-5 g  - Night cry, easy to startle: Cryptotympana pustulata Fabricius [Cicadidae; Cicadae Periostracum] 1-3 g - Dampness obstruction in the middle energizer: Pinellia ternata (Thunb.) Breit. [Araceae; Pinelliae Rhizoma] 1-2 g, Agastache rugosa (Fisch. et Meyer) O. Kuntze [Labiatae; Agastachis Herba] 1-3 g | Not applicable | 4 weeks | None |
| Fan 2021 | TG1: Kaiwei decoction TG2: TG1 + Acupoint Application of Traditional Chinese Medicine | Decoction | Hordeum vulgare L. [Gramineae; Hordei Fructus Germiniatus], Oryza sativa L. [Gramineae; Oryzae Semen Germinatus] 10 g, Pseudostellaria augustifolia Y.N.Lee [Caryophyllaceae; Pseudostellariae Radix], Poria cocos (Schw.) Wolf [Polyporaceae; Poria(Hoelen)], Atractylodes macrocepha-la Koidz [Asteraceae; Atractylodis Rhizoma Alba], Paeonia lactiflora Pall. [Paeoniaceae; Paeoniae Radix Alba], Uncaria sinensis (Oliv.) Havil. [Rubiaceae; Uncariae Ramulus et Uncus], Tadehagi triquetrum (L.) Ohashi, Bù zhā yè, Raphanus sativus var. hortensis for. acanthiformis Makino [Brassicaceae; Raphani Semen] 6 g, Crataegus pinnatifida Bge [Rosaceae; Crataegii Fructus] 5 g, Glycyrrhiza uralensis Fisch. [Leguminosae; Glycyrrhizae Radix] 3 g (cf. TG2: Syzygium aromaticum Merr et Perry [Myrtaceac; Syzygii Flos], Evodia rutaecarpa (Juss.) Benth. [Rutaceae; Evodiae Fructus], Magnolia officinalis Rehder et Wilson [Magnoliaceae; Magnoliae Cortex], Atractylodes macrocepha-la Koidz [Asteraceae; Atractylodis Rhizoma Alba], Agastache rugosa (Fisch. et Meyer) O. Kuntze [Labiatae; Agastachis Herba] 15 g (-> attach at Shen Que, Zhong Wan, Tianshu) | None | Not applicable | 14 days | 1 month |
| Fang 2007 | Qīngrè huà shī yǎng yīn kēlì | Granule | Artemisiae apiacea Hance {Asteraceae; Artemisiae Apiaceae Herba], Coix lachryma-jobi var. ma-yeun (Roman.) Stapf [Gramineae; Coicis Semen], Atractylodes lancea (Thunb.) DC. [Asteraceae; Atractylodis Rhizoma], Fritillaria thunbergii Miq. [Liliaceae; Fritillariae Thunbergii Bulbus], Paeonia lactiflora Pall. [Paeoniaceae; Paeoniae Radix Rubra], Raphanus sativus var. hortensis for. acanthiformis Makino [Brassicaceae; Raphani Semen], Hordeum vulgare L. [Gramineae; Hordei Fructus Germiniatus] | None | Not applicable | 15 days | None |
| Fang 2019 | Xiaoer Yanshi granule | Granule | Panax ginseng C. A. Mey. [Araliaceae; Ginseng Radix], Crataegus pinnatifida Bge [Rosaceae; Crataegii Fructus], Areca catechu L. [Arecaceae; Arecae Semen], Dioscorea batatas Dacne. [Dioscoreaceae; Dioscoreae Rhizoma], Atractylodes macrocepha-la Koidz [Asteraceae; Atractylodis Rhizoma Alba], Picrorrhiza scrophulariiflora Pennell [Scrophulariaceae; Picrorrhozae Rhizoma], Zingiber officinale Rosc. [Zingiberaceae; Zingiberis Rhizoma Siccus], Amomum villosum Lour. [Zingiberaceae; Amomi Fuctus] etc | None | Nèiménggǔ huì fēng yào yè yǒuxiàn gōngsī | 8 weeks | 6 month |
| Feng 2012 | Shenling Baizhu powder | Decoction | - Spleen failing in transportation with spleen qi depletion Poria cocos (Schw.) Wolf [Polyporaceae; Poria(Hoelen)], Atractylodes lancea (Thunb.) DC. [Asteraceae; Atractylodis Rhizoma] 12 g, Codonopsis pilosulae (Fr.) Nannf. [Campanulaceae; Codonopsis Pilosulae Radix], Atractylodes macrocepha-la Koidz [Asteraceae; Atractylodis Rhizoma Alba] 10 g, Magnolia officinalis Rehder et Wilson [Magnoliaceae; Magnoliae Cortex], Agastache rugosa (Fisch. et Meyer) O. Kuntze [Labiatae; Agastachis Herba] 9 g, Glycyrrhiza uralensis Fisch. [Leguminosae; Glycyrrhizae Radix], Saposhnikovia divaricata Schiskin [Apiaceae; Saposhnikovia Radix] 4 g - Spleen failing in transportation with stomach yin deficiency Liriope platyphylla Wang et Tang [Liliaceae; Liriopes Radix] 12 g, Codonopsis pilosulae (Fr.) Nannf. [Campanulaceae; Codonopsis Pilosulae Radix], Atractylodes lancea (Thunb.) DC. [Asteraceae; Atractylodis Rhizoma] 10 g, Rehmannia glutinosa var. purpurea (Makino) Makino et Nemoto [Scrophulariaceae; Rehmanniae Radix], Polygonatum odoratum var. pluriflorum Ohwi [Liliaceae; Polygonati Dodrati Rhizoma], Dendrobium loddigesii Rolfe. [Orchidaceae; Denbrobii Herba] 9 g, Dioscorea batatas Dacne. [Dioscoreaceae; Dioscoreae Rhizoma], Gallus gallus var. domesticus Brisson [Phasianidae; Galli Stomachichum Corium] 6 g，Glycyrrhiza uralensis Fisch. [Leguminosae; Glycyrrhizae Radix] 4 g | None | Not applicable | 14 days | None |
| Feng 2018 | Yigong powder | Decoction | Codonopsis pilosulae (Fr.) Nannf. [Campanulaceae; Codonopsis Pilosulae Radix], Poria cocos (Schw.) Wolf [Polyporaceae; Poria(Hoelen)] 10 g, Atractylodes macrocepha-la Koidz [Asteraceae; Atractylodis Rhizoma Alba], Citrus unshiu Markovich [Rutaceae; Citri Unshius Pericarpium], Hordeum vulgare L. [Gramineae; Hordei Fructus Germiniatus] 8 g, Citrus aurantium L. [Rutaceae; Aurantii Fructus Pericarpium], Amomum villosum Lour. [Zingiberaceae; Amomi Fuctus], Gardenia jasminoides var. grandiflora (Lour.) Nakai [Rubiaceae; Gardeniae Fructus] 6 g, Glycyrrhiza uralensis Fisch. [Leguminosae; Glycyrrhizae Radix] 4 g | - Abdominal distension: Magnolia officinalis Rehder et Wilson [Magnoliaceae; Magnoliae Cortex], Aucklandia lappa Decne [Asteraceae; Aucklandiae Radix]  - Unformed stool: Astragalus membranaceus Bunge [Leguminosae; Astragali Radix], Dolichos lablab L. [Leguminosae; Lablab Semen] | Not applicable | 30 days | None |
| Guo 2006 | Xiaodaoyin granule | Granule | Agastache rugosa (Fisch. et Meyer) O. Kuntze [Labiatae; Agastachis Herba], Perilla frutescens var. crispa (Thunb.) Decne. [Labiatae; Perillae Caulis], Citrus aurantium L. [Rutaceae; Aurantii Fructus Pericarpium], Triticum aestivum L. [Gramineae; Massa Medicata Fermentata], Oryza sativa L. [Gramineae; Oryzae Fructus Germinatus], Hordeum vulgare L. [Gramineae; Hordei Fructus Germiniatus], Raphanus sativus var. hortensis for. acanthiformis Makino [Brassicaceae; Raphani Semen], Crataegus pinnatifida Bge [Rosaceae; Crataegii Fructus], Scutellaria baicalensis Georgi [Labiatae; Scutellariae Radix], Atractylodes macrocepha-la Koidz [Asteraceae; Atractylodis Rhizoma Alba], Schizonepeta tenuifolia (Benth.) Briq. [Labiatae; Schizonepeta Spica], Glycyrrhiza uralensis Fisch. [Leguminosae; Glycyrrhizae Radix]  (1.6 g/pack) | None | - Běi qí zhùshè yè: Shànghǎi fú dá zhìyào yǒuxiàn gōngsī - Shēng mài zhùshè yè: Sìchuān chuān dà huáxī yào yè gǔfèn yǒuxiàn gōngsī | 10-30 days | None |
| Guo 2010 | Xiaoer Piweile granule | Granule | Dioscorea batatas Dacne. [Dioscoreaceae; Dioscoreae Rhizoma], Gallus gallus var. domesticus Brisson [Phasianidae; Galli Stomachichum Corium], Myristica fragrans Houtt. [Myristicaceae; Myristicae Semen], Rheum palmatum L. [Polygonaceae; Rhei Rhizoma], Hordeum vulgare L. [Gramineae; Hordei Fructus Germiniatus] | None | Kūnmíng bāng yǔ zhìyào yǒuxiàn gōngsī shēngchǎn | 4 weeks | None |
| He 2015 | Hé wèi jìnshí yǐn | Decoction | Triticum aestivum L. [Gramineae; Massa Medicata Fermentata], Crataegus pinnatifida Bge [Rosaceae; Crataegii Fructus], Hordeum vulgare L. [Gramineae; Hordei Fructus Germiniatus] 15 g, Poria cocos (Schw.) Wolf [Polyporaceae; Poria(Hoelen)] 8 g, Pinellia ternata (Thunb.) Breit. [Araceae; Pinelliae Rhizoma] 6 g, Citrus unshiu Markovich [Rutaceae; Citri Unshius Pericarpium]5 g, Glycyrrhiza uralensis Fisch. [Leguminosae; Glycyrrhizae Radix] 4 g, Aucklandia lappa Decne [Asteraceae; Aucklandiae Radix] 2.5 g | - Food retention in the stomach: increase Crataegus pinnatifida Bge [Rosaceae; Crataegii Fructus], Hordeum vulgare L. [Gramineae; Hordei Fructus Germiniatus], Triticum aestivum L. [Gramineae; Massa Medicata Fermentata], add Gallus gallus var. domesticus Brisson [Phasianidae; Galli Stomachichum Corium]  - Spleen failing in transportation: Poria cocos (Schw.) Wolf [Polyporaceae; Poria(Hoelen)] Atractylodes macrocepha-la Koidz [Asteraceae; Atractylodis Rhizoma Alba]  - Qi deficiency: Dioscorea batatas Dacne. [Dioscoreaceae; Dioscoreae Rhizoma], Pseudostellaria augustifolia Y.N.Lee [Caryophyllaceae; Pseudostellariae Radix]  - External contraction: Zizyphus jujuba ver. inermis Rehder [Rhamnaceae; Zizyphi Fructus], Zingiber officinale Rosc. [Zingiberaceae; Zingiberis Rhizoma Recens]  - Stomach yin deficiency: Prunus mume Sieb. et Zucc [Rosaceae; Mume Fructus], Chaenomeles sinensis Koehne [Rosaceae; Chaenomelis Fuctus] | Not applicable | 14 days | None |
| He 2018 | Jianpi Xiaoshi decoction | Decoction | Astragalus membranaceus Bunge [Leguminosae; Astragali Radix], Crataegus pinnatifida Bge [Rosaceae; Crataegii Fructus], Hordeum vulgare L. [Gramineae; Hordei Fructus Germiniatus], Triticum aestivum L. [Gramineae; Massa Medicata Fermentata] 15 g, Codonopsis pilosulae (Fr.) Nannf. [Campanulaceae; Codonopsis Pilosulae Radix], Magnolia officinalis Rehder et Wilson [Magnoliaceae; Magnoliae Cortex], Raphanus sativus var. hortensis for. acanthiformis Makino [Brassicaceae; Raphani Semen] 10 g，Atractylodes macrocepha-la Koidz [Asteraceae; Atractylodis Rhizoma Alba], Poncirus trifoliata Rafin. [Rutaceae; Aurantii Immaturus Fructus], Amomum villosum Lour. [Zingiberaceae; Amomi Fuctus], Forsythia suspensa (Thunb.) Vahl [Oleaceae; Forsythiae Fructus], Lonicera japonica Thunb. [Caprifoliaceae; Lonicerae Flos], Citrus unshiu Markovich [Rutaceae; Citri Unshius Pericarpium], Dioscorea batatas Dacne. [Dioscoreaceae; Dioscoreae Rhizoma] 5 g | - Dry mouth, red tongue: Coptis deltoidea C.Y. Cheng et Hsiao [Ranunculaceae; Coptidis Rhizoma] 3 g - Thirst: Dendrobium loddigesii Rolfe. [Orchidaceae; Denbrobii Herba] 5 g  - Constipation: Cannabis sativa L. [Cannabinaceae; Cannabis Fructus] 3 g | Not applicable | 4 weeks | None |
| Hu 2014 | Xiaoer Yanshi granule | Granule | Panax ginseng C. A. Mey. [Araliaceae; Ginseng Radix], Dioscorea batatas Dacne. [Dioscoreaceae; Dioscoreae Rhizoma], Atractylodes macrocepha-la Koidz [Asteraceae; Atractylodis Rhizoma Alba], Crataegus pinnatifida Bge [Rosaceae; Crataegii Fructus], Areca catechu L. [Arecaceae; Arecae Semen], Zingiber officinale Rosc. [Zingiberaceae; Zingiberis Rhizoma Siccus], Picrorrhiza scrophulariiflora Pennell [Scrophulariaceae; Picrorrhozae Rhizoma], Amomum villosum Lour. [Zingiberaceae; Amomi Fuctus] etc. | None | Nèiménggǔ huì fēng yào yè yǒuxiàn gōngsī | 4 weeks | 3, 6 month |
| Hu 2016 | Xingpi Yanger granule | Granule | Gerbera piloselloides (Linn. ) Cass., Pittosporum glabratum Lindl., Emilia sonchifolia (L.) DC., Valeriana jatamansi Jones | None | Guìzhōu jiàn xìng yào yè yǒuxiàn gōngsī | 8 weeks | None |
| Hua 2009 | Píng wèi sàn | Decoction | Atractylodes lancea (Thunb.) DC. [Asteraceae; Atractylodis Rhizoma], Atractylodes macrocepha-la Koidz [Asteraceae; Atractylodis Rhizoma Alba], Oryza sativa L. [Gramineae; Oryzae Fructus Germinatus], Hordeum vulgare L. [Gramineae; Hordei Fructus Germiniatus], Gallus gallus var. domesticus Brisson [Phasianidae; Galli Stomachichum Corium] 10 g, Magnolia officinalis Rehder et Wilson [Magnoliaceae; Magnoliae Cortex], Citrus unshiu Markovich [Rutaceae; Citri Unshius Pericarpium]8 g, Glycyrrhiza uralensis Fisch. [Leguminosae; Glycyrrhizae Radix] 3 g | - Spleen-stomach deficiency, lack of strength , sloppy stool: Dioscorea batatas Dacne. [Dioscoreaceae; Dioscoreae Rhizoma], Codonopsis pilosulae (Fr.) Nannf. [Campanulaceae; Codonopsis Pilosulae Radix] 10 g - Stomach yang deficiency with red tongue, dry mouth, dry boung stool: Dendrobium loddigesii Rolfe. [Orchidaceae; Denbrobii Herba], Prunus mume Sieb. et Zucc [Rosaceae; Mume Fructus] 10 g - Liver-spleen disharmony with vexation, easy to cry, night anxiety: Curcuma aromatica Salisb. [Zingiberaceae; Curcumae Radix], Paeonia lactiflora Pall. [Paeoniaceae; Paeoniae Radix Alba] 10 g | Not applicable | 4 weeks | None |
| Huang 2011 | Yigong powder | Decoction | Codonopsis pilosulae (Fr.) Nannf. [Campanulaceae; Codonopsis Pilosulae Radix], Poria cocos (Schw.) Wolf [Polyporaceae; Poria(Hoelen)] 10 g，Atractylodes macrocepha-la Koidz [Asteraceae; Atractylodis Rhizoma Alba], Citrus unshiu Markovich [Rutaceae; Citri Unshius Pericarpium], Hordeum vulgare L. [Gramineae; Hordei Fructus Germiniatus], Oryza sativa L. [Gramineae; Oryzae Fructus Germinatus] 8 g, Citrus aurantium L. [Rutaceae; Aurantii Fructus Pericarpium], Amomum villosum Lour. [Zingiberaceae; Amomi Fuctus], Gardenia jasminoides var. grandiflora (Lour.) Nakai [Rubiaceae; Gardeniae Fructus] 6 gGlycyrrhiza uralensis Fisch. [Leguminosae; Glycyrrhizae Radix] 4 g | None | Not applicable | 7 days | None |
| Huang 2015 | Xingpi Yanger granule | Granule | Gerbera piloselloides (Linn. ) Cass., Pittosporum glabratum Lindl., Emilia sonchifolia (L.) DC., Valeriana jatamansi Jones | None | Guìzhōu jiàn xìng yào yè yǒuxiàn gōngsī | TG: 2-4 weeks CG: 4 weeks | None |
| Jin 2019 | Children's compound Jineijin chewable tablet | Tablet | Gallus gallus var. domesticus Brisson [Phasianidae; Galli Stomachichum Corium], Triticum aestivum L. [Gramineae; Massa Medicata Fermentata] | None | Hénán tài lóng yào yè gǔfèn yǒuxiàn gōngsī | 7 weeks | NR |
| Jing 2019 | Jianwei Xiaoshi decoction | Decoction | Pseudostellaria augustifolia Y.N.Lee [Caryophyllaceae; Pseudostellariae Radix], Citrus unshiu Markovich [Rutaceae; Citri Unshius Pericarpium], Dioscorea batatas Dacne. [Dioscoreaceae; Dioscoreae Rhizoma], Hordeum vulgare L. [Gramineae; Hordei Fructus Germiniatus], Crataegus pinnatifida Bge [Rosaceae; Crataegii Fructus] | None | Jiāngsū jì chuān yào yè jítuán yǒuxiàn gōngsī | 2 weeks | None |
| Kang 2005 | Féi er kǒufú yè | Decoction | Bupleurum falcatum Linne [Apiaceae; Bupleuri Radix], Ulmus macrocarpa Hance [Ulmaceae; Ulmi Pasta Semen], Quisqualis indica L. [Combretaceae; Quisqualis Fuctus], Akebia quinata Decne. [Lardizabalaceae; Akebiae Caulis], Lophatherum gracile Brongn. [Gramineae; Lophatheri Herba], Androsace umbellata (Lour.) Merr. [Primulacae; Stellariae Radix], Chrysomyia megacephala Fabricius [Calliphoridae; Chrysomyiae], Picrorrhiza scrophulariiflora Pennell [Scrophulariaceae; Picrorrhozae Rhizoma], Triticum aestivum L. [Gramineae; Massa Medicata Fermentata], Hordeum vulgare L. [Gramineae; Hordei Fructus Germiniatus], Areca catechu L. [Arecaceae; Arecae Semen], Aucklandia lappa Decne [Asteraceae; Aucklandiae Radix], Amomum krabanh Pierre ex Cagnep [Zingiberaceae; Amomi Rotundus Fructus] | None | Not applicable | 2 weeks | None |
| Kang 2018 | Shenling Baizhu powder | Decoction | Poria cocos (Schw.) Wolf [Polyporaceae; Poria(Hoelen)], Atractylodes lancea (Thunb.) DC. [Asteraceae; Atractylodis Rhizoma] 12 g, Codonopsis pilosulae (Fr.) Nannf. [Campanulaceae; Codonopsis Pilosulae Radix], Atractylodes macrocepha-la Koidz [Asteraceae; Atractylodis Rhizoma Alba] 10 g, Agastache rugosa (Fisch. et Meyer) O. Kuntze [Labiatae; Agastachis Herba], Magnolia officinalis Rehder et Wilson [Magnoliaceae; Magnoliae Cortex] 9 g Glycyrrhiza uralensis Fisch. [Leguminosae; Glycyrrhizae Radix] 6 g, Saposhnikovia divaricata Schiskin [Apiaceae; Saposhnikovia Radix] 3 g | None | Not applicable | 14 days | None |
| Kuang 2004 | Jianpi pill | Pill | Panax ginseng C. A. Mey. [Araliaceae; Ginseng Radix], Poria cocos (Schw.) Wolf [Polyporaceae; Poria(Hoelen)], Dioscorea batatas Dacne. [Dioscoreaceae; Dioscoreae Rhizoma], Aucklandia lappa Decne [Asteraceae; Aucklandiae Radix], Myristica fragrans Houtt. [Myristicaceae; Myristicae Semen], Coptis deltoidea C.Y. Cheng et Hsiao [Ranunculaceae; Coptidis Rhizoma], Hordeum vulgare L. [Gramineae; Hordei Fructus Germiniatus], Triticum aestivum L. [Gramineae; Massa Medicata Fermentata], Citrus unshiu Markovich [Rutaceae; Citri Unshius Pericarpium], Atractylodes macrocepha-la Koidz [Asteraceae; Atractylodis Rhizoma Alba], Crataegus pinnatifida Bge [Rosaceae; Crataegii Fructus], Glycyrrhiza uralensis Fisch. [Leguminosae; Glycyrrhizae Radix] | None | Not applicable | 28-42 days | None |
| Lai 2008 | Shenling Baizhu powder | Decoction | Hordeum vulgare L. [Gramineae; Hordei Fructus Germiniatus], Oryza sativa L. [Gramineae; Oryzae Fructus Germinatus] 15 g, Poria cocos (Schw.) Wolf [Polyporaceae; Poria(Hoelen)] 10 g, Codonopsis pilosulae (Fr.) Nannf. [Campanulaceae; Codonopsis Pilosulae Radix], Atractylodes macrocepha-la Koidz [Asteraceae; Atractylodis Rhizoma Alba], Dioscorea batatas Dacne. [Dioscoreaceae; Dioscoreae Rhizoma], Citrus unshiu Markovich [Rutaceae; Citri Unshius Pericarpium], Gallus gallus var. domesticus Brisson [Phasianidae; Galli Stomachichum Corium], Crataegus pinnatifida Bge [Rosaceae; Crataegii Fructus], Triticum aestivum L. [Gramineae; Massa Medicata Fermentata] 6 g, Atractylodes lancea (Thunb.) DC. [Asteraceae; Atractylodis Rhizoma] 5 g, Glycyrrhiza uralensis Fisch. [Leguminosae; Glycyrrhizae Radix] 4 g | None | Not applicable | 2 months | None |
| Lei 2014 | Sijunzi decoction | Decoction | Panax ginseng C. A. Mey. [Araliaceae; Ginseng Radix], Poria cocos (Schw.) Wolf [Polyporaceae; Poria(Hoelen)] 10 g, Citrus unshiu Markovich [Rutaceae; Citri Unshius Pericarpium], Dioscorea batatas Dacne. [Dioscoreaceae; Dioscoreae Rhizoma] 5-10 g, Crataegus pinnatifida Bge [Rosaceae; Crataegii Fructus], Hordeum vulgare L. [Gramineae; Hordei Fructus Germiniatus] 5-15 g, Atractylodes macrocepha-la Koidz [Asteraceae; Atractylodis Rhizoma Alba] 5 g, Glycyrrhiza uralensis Fisch. [Leguminosae; Glycyrrhizae Radix] 3 g | None | Not applicable | NR | None |
| Lei 2016 | Xingpi Xiaoshi decoction | Decoction | Pseudostellaria augustifolia Y.N.Lee [Caryophyllaceae; Pseudostellariae Radix], Poncirus trifoliata Rafin. [Rutaceae; Aurantii Immaturus Fructus], Tadehagi triquetrum (L.) Ohashi, Gallus gallus var. domesticus Brisson [Phasianidae; Galli Stomachichum Corium], Poria cocos (Schw.) Wolf [Polyporaceae; Poria(Hoelen)], Bù zhā yè, Crataegus pinnatifida Bge [Rosaceae; Crataegii Fructus] 10 g, Amomum villosum Lour. [Zingiberaceae; Amomi Fuctus], Citrus unshiu Markovich [Rutaceae; Citri Unshius Pericarpium]5 g | None | Not applicable | 4 weeks | None |
| Li 1990 | Yigong powder | Granule | Astragalus membranaceus Bunge [Leguminosae; Astragali Radix], Atractylodes macrocepha-la Koidz [Asteraceae; Atractylodis Rhizoma Alba], Poria cocos (Schw.) Wolf [Polyporaceae; Poria(Hoelen)], Polygonatum sibiricum Redoute [Liliaceae; Polygonati Rhizoma], Citrus unshiu Markovich [Rutaceae; Citri Unshius Pericarpium], Baphicacanthus cusia (Nees) Bremek [Acanthaceae; Indigo Pulcerata Levis], Gallus gallus var. domesticus Brisson [Phasianidae; Galli Stomachichum Corium], Glycyrrhiza uralensis Fisch. [Leguminosae; Glycyrrhizae Radix]  (3:3:3:3:2:2:1:1 ratio, 3g/pack) | None | Běijīng dì liù zhìyào chǎng | 3 months | None |
| Li 2005 | Xingpi Xiaoshi decoction | Decoction | Pseudostellaria augustifolia Y.N.Lee [Caryophyllaceae; Pseudostellariae Radix], Bù zhā yè, Citrus unshiu Markovich [Rutaceae; Citri Unshius Pericarpium], Gallus gallus var. domesticus Brisson [Phasianidae; Galli Stomachichum Corium], Striga asiatica (L.) O. Kuntze, Poncirus trifoliata Rafin. [Rutaceae; Aurantii Immaturus Fructus], Amomum villosum Lour. [Zingiberaceae; Amomi Fuctus], Crataegus pinnatifida Bge [Rosaceae; Crataegii Fructus] 3~6 g, Triticum aestivum L. [Gramineae; Massa Medicata Fermentata], Codonopsis pilosulae (Fr.) Nannf. [Campanulaceae; Codonopsis Pilosulae Radix], Hordeum vulgare L. [Gramineae; Hordei Fructus Germiniatus] 5~10 g | None | Not applicable | 1-2 months | None |
| Li 2006a | Yangwei Zengye decoction | Decoction | Citrus unshiu Markovich [Rutaceae; Citri Unshius Pericarpium]15 g, Pseudostellaria augustifolia Y.N.Lee [Caryophyllaceae; Pseudostellariae Radix], Rehmannia glutinosa var. purpurea (Makino) Makino et Nemoto [Scrophulariaceae; Rehmanniae Radix], Adenophora triphylla var. japonica Hara [Campanulaceae; Adenophorae Radix], Dendrobium loddigesii Rolfe. [Orchidaceae; Denbrobii Herba], Prunus mume Sieb. et Zucc [Rosaceae; Mume Fructus], Atractylodes macrocepha-la Koidz [Asteraceae; Atractylodis Rhizoma Alba], Selaginella tamariscina (Beauv.) Spring [Selaginellaceae; Selaginelliae Herba], Gallus gallus var. domesticus Brisson [Phasianidae; Galli Stomachichum Corium], Crataegus pinnatifida Bge [Rosaceae; Crataegii Fructus], Hordeum vulgare L. [Gramineae; Hordei Fructus Germiniatus], Glycyrrhiza uralensis Fisch. [Leguminosae; Glycyrrhizae Radix] 10 g, Picrorrhiza scrophulariiflora Pennell [Scrophulariaceae; Picrorrhozae Rhizoma], Poncirus trifoliata Rafin. [Rutaceae; Aurantii Immaturus Fructus] 6 g | None | Not applicable | 1 month | 1, 2, 3, 4 week |
| Li 2006b | Líng guì lóng mǔ kēlì | Granule | Gazella subgutturosa Guld. [Bovidae; Gazellae Cornu], Cinnamomum cassia Blume [Lauraceae; Cinnamomi Ramulus], Paeonia lactiflora Pall. [Paeoniaceae; Paeoniae Radix Alba], Atractylodes macrocepha-la Koidz [Asteraceae; Atractylodis Rhizoma Alba], Elephas Species [Elephantidae; Fossilia Ossis Mastodi], Ostrea gigas Thunb. [Ostreidae; Ostreae Concha], Crataegus pinnatifida Bge [Rosaceae; Crataegii Fructus], Triticum aestivum L. [Gramineae; Massa Medicata Fermentata], Hordeum vulgare L. [Gramineae; Hordei Fructus Germiniatus] | None | Not applicable | 2 weeks | 6 month |
| Li 2010 | Bǎobǎo xǐ shí tángjiāng | Decoction | Atractylodes macrocepha-la Koidz [Asteraceae; Atractylodis Rhizoma Alba] 10 g, Pharbitis nil Chois. [Convolvulaceae; Pharbitidis Semen], Pseudostellaria augustifolia Y.N.Lee [Caryophyllaceae; Pseudostellariae Radix], Citrus unshiu Markovich [Rutaceae; Citri Unshius Pericarpium], Areca catechu L. [Arecaceae; Arecae Pericarpium], Triticum aestivum L. [Gramineae; Massa Medicata Fermentata] 6 g, Areca catechu L. [Arecaceae; Arecae Semen], Gallus gallus var. domesticus Brisson [Phasianidae; Galli Stomachichum Corium], Crataegus pinnatifida Bge [Rosaceae; Crataegii Fructus], Prunus mume Sieb. et Zucc [Rosaceae; Mume Fructus], Scirpus flaviatilis (Torr.) A. Gray [Sparganiaceae; Scirpi Rhizoma], Curcuma zedoaria Rocs. [Zingiberaceae; Zedoariae Rhizoma], Aucklandia lappa Decne [Asteraceae; Aucklandiae Radix], Mentha arvensis var. piperascens Makinv. [Labiatae; Menthae Herba], Cyperus rotundus L. [Cyperaceae; Cyperi Rhizoma] 3 g, Apis mellifera L. [Apidae; Mel] 15 g | None | Not applicable | 5 days | None |
| Li 2011 | Báilíngjiàn pí kēlì | Granule | Crataegus pinnatifida Bge [Rosaceae; Crataegii Fructus], Atractylodes macrocepha-la Koidz [Asteraceae; Atractylodis Rhizoma Alba], Poria cocos (Schw.) Wolf [Polyporaceae; Poria(Hoelen)] | None | Not applicable | 1 month | None |
| Li 2013 | Wangshi Baochi pill | Pill | Rheum palmatum L. [Polygonaceae; Rhei Rhizoma], Coptis deltoidea C.Y. Cheng et Hsiao [Ranunculaceae; Coptidis Rhizoma], Croton tiglium L. [Euphorbiaceae; Crotonis Seminis Pulvis], Zingiber officinale Rosc. [Zingiberaceae; Zingiberis Rhizoma Recens] etc | None | Jīnghuá zhìyào jítuán gǔfèn yǒuxiàn gōngsī | 90 days | None |
| Li 2014a | Shugan Jianpi Huayu decoction | Decoction | Triticum aestivum L. [Gramineae; Massa Medicata Fermentata], Crataegus pinnatifida Bge [Rosaceae; Crataegii Fructus], Dolichos lablab L. [Leguminosae; Lablab Semen], Poria cocos (Schw.) Wolf [Polyporaceae; Poria(Hoelen)] 10 g, Bupleurum falcatum Linne [Apiaceae; Bupleuri Radix], Cyperus rotundus L. [Cyperaceae; Cyperi Rhizoma], Amomum villosum Lour. [Zingiberaceae; Amomi Fuctus], Selaginella tamariscina (Beauv.) Spring [Selaginellaceae; Selaginelliae Herba], Atractylodes macrocepha-la Koidz [Asteraceae; Atractylodis Rhizoma Alba], Scirpus flaviatilis (Torr.) A. Gray [Sparganiaceae; Scirpi Rhizoma], Curcuma zedoaria Rocs. [Zingiberaceae; Zedoariae Rhizoma] 6 g | - Spleen-stomach deficiency: Codonopsis pilosulae (Fr.) Nannf. [Campanulaceae; Codonopsis Pilosulae Radix], Citrus unshiu Markovich [Rutaceae; Citri Unshius Pericarpium]6 g - Dampness: Agastache rugosa (Fisch. et Meyer) O. Kuntze [Labiatae; Agastachis Herba], Eupatorium chinese for. tripartitum H. Hara [Asteraceae; Eupatorii Herba] 6 g - Liver qi depression: Curcuma aromatica Salisb. [Zingiberaceae; Curcumae Radix], Paeonia lactiflora Pall. [Paeoniaceae; Paeoniae Radix Alba] 6 g - Food stagnation: Areca catechu L. [Arecaceae; Arecae Semen], Citrus aurantium L. [Rutaceae; Aurantii Fructus Pericarpium] 6 g - Blood stasis: Paeonia lactiflora Pall. [Paeoniaceae; Paeoniae Radix Rubra] Prunus percisa (L.) Batsch [Rosaceae; Persicae Semen] 6 g | Not applicable | 52 days | 20 day |
| Li 2014b | spleen-stomach disharmony: Qū mài zhǐ shù wán  spleen-stomach qi deficiency: Liujunzi decoction spleen-stomach yin deficiency: Yangwei Zengye decoction | Decoction | - Spleen-stomach disharmony: Triticum aestivum L. [Gramineae; Massa Medicata Fermentata], Crataegus pinnatifida Bge [Rosaceae; Crataegii Fructus], Hordeum vulgare L. [Gramineae; Hordei Fructus Germiniatus], Atractylodes lancea (Thunb.) DC. [Asteraceae; Atractylodis Rhizoma], Citrus unshiu Markovich [Rutaceae; Citri Unshius Pericarpium], Gallus gallus var. domesticus Brisson [Phasianidae; Galli Stomachichum Corium], Pinellia ternata (Thunb.) Breit. [Araceae; Pinelliae Rhizoma], Poria cocos (Schw.) Wolf [Polyporaceae; Poria(Hoelen)], Glycyrrhiza uralensis Fisch. [Leguminosae; Glycyrrhizae Radix] - Spleen-stomach qi deficiency: Codonopsis pilosulae (Fr.) Nannf. [Campanulaceae; Codonopsis Pilosulae Radix], Atractylodes macrocepha-la Koidz [Asteraceae; Atractylodis Rhizoma Alba], Poria cocos (Schw.) Wolf [Polyporaceae; Poria(Hoelen)], Citrus unshiu Markovich [Rutaceae; Citri Unshius Pericarpium], Astragalus membranaceus Bunge [Leguminosae; Astragali Radix], Saposhnikovia divaricata Schiskin [Apiaceae; Saposhnikovia Radix], Dioscorea batatas Dacne. [Dioscoreaceae; Dioscoreae Rhizoma], Crataegus pinnatifida Bge [Rosaceae; Crataegii Fructus], Triticum aestivum L. [Gramineae; Massa Medicata Fermentata] - Spleen-stomach yin deficiency: Dendrobium loddigesii Rolfe. [Orchidaceae; Denbrobii Herba], Prunus mume Sieb. et Zucc [Rosaceae; Mume Fructus], Glehnia littoralis Fr. Schm. [Apiaceae; Glehniae Radix], Glycyrrhiza uralensis Fisch. [Leguminosae; Glycyrrhizae Radix], Paeonia lactiflora Pall. [Paeoniaceae; Paeoniae Radix Alba], Polygonatum odoratum var. pluriflorum Ohwi [Liliaceae; Polygonati Dodrati Rhizoma], Liriope platyphylla Wang et Tang [Liliaceae; Liriopes Radix], Dioscorea batatas Dacne. [Dioscoreaceae; Dioscoreae Rhizoma], Hordeum vulgare L. [Gramineae; Hordei Fructus Germiniatus], Oryza sativa L. [Gramineae; Oryzae Fructus Germinatus] | None | Not applicable | 3 months | None |
| Li 2015a | Yigong powder | Decoction | Poria cocos (Schw.) Wolf [Polyporaceae; Poria(Hoelen)] 15 g, Pseudostellaria augustifolia Y.N.Lee [Caryophyllaceae; Pseudostellariae Radix] 10 g, Atractylodes macrocepha-la Koidz [Asteraceae; Atractylodis Rhizoma Alba], Atractylodes lancea (Thunb.) DC. [Asteraceae; Atractylodis Rhizoma] 5-10 g, Citrus unshiu Markovich [Rutaceae; Citri Unshius Pericarpium], Glycyrrhiza uralensis Fisch. [Leguminosae; Glycyrrhizae Radix] 6 g | None | Not applicable | 14 days | None |
| Li 2015b | spleen-stomach disharmony: Qū mài zhǐ shù wán spleen-stomach qi deficiency: Qǐ pí wán spleen-stomach yin deficiency: yì wèi tāng | Decoction | - Spleen-stomach disharmony: Atractylodes macrocepha-la Koidz [Asteraceae; Atractylodis Rhizoma Alba], Triticum aestivum L. [Gramineae; Massa Medicata Fermentata], Hordeum vulgare L. [Gramineae; Hordei Fructus Germiniatus] 8 g, Pinellia ternata (Thunb.) Breit. [Araceae; Pinelliae Rhizoma], Citrus unshiu Markovich [Rutaceae; Citri Unshius Pericarpium], Poncirus trifoliata Rafin. [Rutaceae; Aurantii Immaturus Fructus] 4 g - Spleen-stomach qi deficiency: Panax ginseng C. A. Mey. [Araliaceae; Ginseng Radix], Atractylodes macrocepha-la Koidz [Asteraceae; Atractylodis Rhizoma Alba], Poria cocos (Schw.) Wolf [Polyporaceae; Poria(Hoelen)], Dioscorea batatas Dacne. [Dioscoreaceae; Dioscoreae Rhizoma], Nelumbo nucifera Gaertner [Nymphaceae; Nelumbinis Semen] 10 g, Citrus unshiu Markovich [Rutaceae; Citri Unshius Pericarpium], Alisma orientalis (Sam) Juzep [Alismataceae; Alismatis Rhizoma], Crataegus pinnatifida Bge [Rosaceae; Crataegii Fructus], Triticum aestivum L. [Gramineae; Massa Medicata Fermentata], Hordeum vulgare L. [Gramineae; Hordei Fructus Germiniatus], Glycyrrhiza uralensis Fisch. [Leguminosae; Glycyrrhizae Radix] 5 g  - Spleen-stomach yin deficiency: Glehnia littoralis Fr. Schm. [Apiaceae; Glehniae Radix], Dendrobium loddigesii Rolfe. [Orchidaceae; Denbrobii Herba], Liriope platyphylla Wang et Tang [Liliaceae; Liriopes Radix], Rehmannia glutinosa var. purpurea (Makino) Makino et Nemoto [Scrophulariaceae; Rehmanniae Radix], Polygonatum odoratum var. pluriflorum Ohwi [Liliaceae; Polygonati Dodrati Rhizoma] 10 g, Hordeum vulgare L. [Gramineae; Hordei Fructus Germiniatus], Oryza sativa L. [Gramineae; Oryzae Fructus Germinatus] 8 g, Dioscorea batatas Dacne. [Dioscoreaceae; Dioscoreae Rhizoma], Nelumbo nucifera Gaertner [Nymphaceae; Nelumbinis Semen], Prunus mume Sieb. et Zucc [Rosaceae; Mume Fructus] 6 g | None | Not applicable | 1 month | None |
| Li 2016a | Jianbaoling granule | Granule | Tremella fuciformis Berk., Dioscorea batatas Dacne. [Dioscoreaceae; Dioscoreae Rhizoma], Poria cocos (Schw.) Wolf [Polyporaceae; Poria(Hoelen)], Crataegus pinnatifida Bge [Rosaceae; Crataegii Fructus] | None | Xī'ān rén rén yào yè yǒuxiàn gōngsī | 8 weeks | None |
| Li 2016b | Jianbaoling granule | Granule | Tremella fuciformis Berk., Dioscorea batatas Dacne. [Dioscoreaceae; Dioscoreae Rhizoma], Poria cocos (Schw.) Wolf [Polyporaceae; Poria(Hoelen)], Crataegus pinnatifida Bge [Rosaceae; Crataegii Fructus] | None | Xī'ān rén rén yào yè yǒuxiàn gōngsī | 6 weeks | None |
| Li 2017a | Xiao'er Xiaozhi kēlì | Granule | Mangifera indica L.[M.austroyunnanensis Hu], Crataegus pinnatifida Bge [Rosaceae; Crataegii Fructus], Citrus aurantium L. [Rutaceae; Aurantii Fructus Pericarpium], Artemisia capillaris Thunb. [Asteraceae; Artemisiae Capillaris Herba], Glycyrrhiza uralensis Fisch. [Leguminosae; Glycyrrhizae Radix] 등 | None | Not applicable | 2 weeks | 2 week |
| Li 2017b | Liujunzi decoction | Decoction | Dioscorea batatas Dacne. [Dioscoreaceae; Dioscoreae Rhizoma] 12 g, Triticum aestivum L. [Gramineae; Massa Medicata Fermentata], Crataegus pinnatifida Bge [Rosaceae; Crataegii Fructus] 10 g, Pseudostellaria augustifolia Y.N.Lee [Caryophyllaceae; Pseudostellariae Radix], Poria cocos (Schw.) Wolf [Polyporaceae; Poria(Hoelen)], Atractylodes macrocepha-la Koidz [Asteraceae; Atractylodis Rhizoma Alba] 9 g, Citrus unshiu Markovich [Rutaceae; Citri Unshius Pericarpium], Gallus gallus var. domesticus Brisson [Phasianidae; Galli Stomachichum Corium], Pinellia ternata (Thunb.) Breit. [Araceae; Pinelliae Rhizoma] 6 g, Glycyrrhiza uralensis Fisch. [Leguminosae; Glycyrrhizae Radix] 3 g | None | Not applicable | 1 month | None |
| Li 2019a | Xingpi Yanger granule | Granule | Gerbera piloselloides (Linn. ) Cass., Pittosporum glabratum Lindl., Emilia sonchifolia (L.) DC., Valeriana jatamansi Jones | None | Guìzhōu jiàn xìng yào yè yǒuxiàn gōngsī | 8 weeks | 6 month |
| Li 2019b | Jianpi decoction | Decoction | Poria cocos (Schw.) Wolf [Polyporaceae; Poria(Hoelen)] 9 g, Atractylodes macrocepha-la Koidz [Asteraceae; Atractylodis Rhizoma Alba], Artemisia capillaris Thunb. [Asteraceae; Artemisiae Capillaris Herba], Gallus gallus var. domesticus Brisson [Phasianidae; Galli Stomachichum Corium], Coix lachryma-jobi var. ma-yeun (Roman.) Stapf [Gramineae; Coicis Semen] 6 g, Picrorrhiza scrophulariiflora Pennell [Scrophulariaceae; Picrorrhozae Rhizoma], Chrysomyia megacephala Fabricius [Calliphoridae; Chrysomyiae], Codonopsis pilosulae (Fr.) Nannf. [Campanulaceae; Codonopsis Pilosulae Radix], Crataegus pinnatifida Bge [Rosaceae; Crataegii Fructus], Glycyrrhiza uralensis Fisch. [Leguminosae; Glycyrrhizae Radix] 3 g | None | Not applicable | 14 days | None |
| Li 2020 | Yanshi decoction | Decoction | Hordeum vulgare L. [Gramineae; Hordei Fructus Germiniatus] 15 g, Crataegus pinnatifida Bge [Rosaceae; Crataegii Fructus], Triticum aestivum L. [Gramineae; Massa Medicata Fermentata], Atractylodes lancea (Thunb.) DC. [Asteraceae; Atractylodis Rhizoma], Scutellaria baicalensis Georgi [Labiatae; Scutellariae Radix], Citrus unshiu Markovich [Rutaceae; Citri Unshius Pericarpium], Poria cocos (Schw.) Wolf [Polyporaceae; Poria(Hoelen)], Agastache rugosa (Fisch. et Meyer) O. Kuntze [Labiatae; Agastachis Herba] 10 g, Pinellia ternata (Thunb.) Breit. [Araceae; Pinelliae Rhizoma] 9 g, Magnolia officinalis Rehder et Wilson [Magnoliaceae; Magnoliae Cortex] 6 g Glycyrrhiza uralensis Fisch. [Leguminosae; Glycyrrhizae Radix] 3 g | None | Not applicable | 4 weeks | None |
| Lian 2008 | Tiaozhong yin | Decoction | Bupleurum falcatum Linne [Apiaceae; Bupleuri Radix], Poncirus trifoliata Rafin. [Rutaceae; Aurantii Immaturus Fructus], Paeonia lactiflora Pallas [Paeoniaceae; Paeoniae Radix], Poria cocos (Schw.) Wolf [Polyporaceae; Poria(Hoelen)], Corydalis ternata Nakai [Papaveraceae; Corydalis (Tuber) Rhizoma], Atractylodes macrocepha-la Koidz [Asteraceae; Atractylodis Rhizoma Alba], Crataegus pinnatifida Bge [Rosaceae; Crataegii Fructus], Triticum aestivum L. [Gramineae; Massa Medicata Fermentata] | None | Not applicable | NR | None |
| Lian 2013 | Sini powder | Decoction | Bupleurum falcatum Linne [Apiaceae; Bupleuri Radix], Paeonia lactiflora Pallas [Paeoniaceae; Paeoniae Radix] 9 g, Poncirus trifoliata Rafin. [Rutaceae; Aurantii Immaturus Fructus], Poria cocos (Schw.) Wolf [Polyporaceae; Poria(Hoelen)], Corydalis ternata Nakai [Papaveraceae; Corydalis (Tuber) Rhizoma], Atractylodes macrocepha-la Koidz [Asteraceae; Atractylodis Rhizoma Alba], Crataegus pinnatifida Bge [Rosaceae; Crataegii Fructus], Triticum aestivum L. [Gramineae; Massa Medicata Fermentata], Amomum villosum Lour. [Zingiberaceae; Amomi Fuctus] 6 g, Glycyrrhiza uralensis Fisch. [Leguminosae; Glycyrrhizae Radix] 4 g | None | Not applicable | 2-4 weeks | None |
| Lian 2020a | Tiaozhong yin | Decoction | Atractylodes macrocepha-la Koidz [Asteraceae; Atractylodis Rhizoma Alba], Paeonia lactiflora Pall. [Paeoniaceae; Paeoniae Radix Alba], Poria cocos (Schw.) Wolf [Polyporaceae; Poria(Hoelen)] 9 g, Pseudostellaria augustifolia Y.N.Lee [Caryophyllaceae; Pseudostellariae Radix], Bupleurum falcatum Linne [Apiaceae; Bupleuri Radix], Citrus unshiu Markovich [Rutaceae; Citri Unshius Pericarpium], Platycodon grandiflorum (Jacq.) A. DC. [Campanulaceae; Platycodi Radix], Glycyrrhiza uralensis Fisch. [Leguminosae; Glycyrrhizae Radix], Citrus aurantium L. [Rutaceae; Aurantii Fructus Pericarpium] 6 g | None | Not applicable | 8 weeks | None |
| Lian 2020b | Tiaozhong decoction | Decoction | Atractylodes macrocepha-la Koidz [Asteraceae; Atractylodis Rhizoma Alba], Paeonia lactiflora Pall. [Paeoniaceae; Paeoniae Radix Alba], Poria cocos (Schw.) Wolf [Polyporaceae; Poria(Hoelen)] 9 g, Pseudostellaria augustifolia Y.N.Lee [Caryophyllaceae; Pseudostellariae Radix], Bupleurum falcatum Linne [Apiaceae; Bupleuri Radix], Citrus unshiu Markovich [Rutaceae; Citri Unshius Pericarpium], Platycodon grandiflorum (Jacq.) A. DC. [Campanulaceae; Platycodi Radix], Glycyrrhiza uralensis Fisch. [Leguminosae; Glycyrrhizae Radix], Citrus aurantium L. [Rutaceae; Aurantii Fructus Pericarpium] 6 g | None | Not applicable | 4 weeks | None |
| Liang 2009 | Xiǎo'ér jiàn wèi tāng | Decoction | Citrus unshiu Markovich [Rutaceae; Citri Unshius Pericarpium], Atractylodes lancea (Thunb.) DC. [Asteraceae; Atractylodis Rhizoma], Magnolia officinalis Rehder et Wilson [Magnoliaceae; Magnoliae Cortex], Agastache rugosa (Fisch. et Meyer) O. Kuntze [Labiatae; Agastachis Herba], Crataegus pinnatifida Bge [Rosaceae; Crataegii Fructus], Triticum aestivum L. [Gramineae; Massa Medicata Fermentata], Gallus gallus var. domesticus Brisson [Phasianidae; Galli Stomachichum Corium], Amomum villosum Lour. [Zingiberaceae; Amomi Fuctus], Citrus aurantium L. [Rutaceae; Aurantii Fructus Pericarpium], Perilla frutescens var. crispa (Thunb.) Decne. [Labiatae; Perillae Caulis], Selaginella tamariscina (Beauv.) Spring [Selaginellaceae; Selaginelliae Herba] | - Constipation: Trichosanthes kirilowii Maxim. [Cucurbitaceae; Trichosanthis Fructus], Zhì jūn - Bitter taste in the mouth: Coptis deltoidea C.Y. Cheng et Hsiao [Ranunculaceae; Coptidis Rhizoma]  - Sloppy stool: Poria cocos (Schw.) Wolf [Polyporaceae; Poria(Hoelen)], Coix lachryma-jobi var. ma-yeun (Roman.) Stapf [Gramineae; Coicis Semen]  - Frequent gastrointestinal cramps: Corydalis ternata Nakai [Papaveraceae; Corydalis (Tuber) Rhizoma], Uncaria rhynchophylla (Miq.) Jacks. [Rubiaceae; Uncariae Ramulus cum Uncis]  - Obvious elching: Aquilaria agallocha Roxb. [Thymelaceae; Aquilariae Resinatum Lignum] Citrus aurantium L. [Rutaceae; Aurantii Fructus Pericarpium] | Not applicable | 2 weeks | None |
| Liang 2014a | Hé wèi liáo gān kēlì | Granule | Citrus unshiu Markovich [Rutaceae; Citrii Unshiu Immaturi Pericarpium], Bupleurum falcatum Linne [Apiaceae; Bupleuri Radix], Paeonia lactiflora Pall. [Paeoniaceae; Paeoniae Radix Alba], Atractylodes macrocepha-la Koidz [Asteraceae; Atractylodis Rhizoma Alba], Poria cocos (Schw.) Wolf [Polyporaceae; Poria(Hoelen)], Glycyrrhiza uralensis Fisch. [Leguminosae; Glycyrrhizae Radix], Nelumbo nucifera Gaertner [Nymphaceae; Nelumbinis Semen], Euryale ferox Salisb. [Nymphaceae; Euryales Semen], Crataegus pinnatifida Bge [Rosaceae; Crataegii Fructus], Hordeum vulgare L. [Gramineae; Hordei Fructus Germiniatus], Quisqualis indica L. [Combretaceae; Quisqualis Fructus], Areca catechu L. [Arecaceae; Arecae Semen], Prunus mume Sieb. et Zucc [Rosaceae; Mume Fructus], Glehnia littoralis Fr. Schm. [Apiaceae; Glehniae Radix], Liriope platyphylla Wang et Tang [Liliaceae; Liriopes Radix], Picrorrhiza scrophulariiflora Pennell [Scrophulariaceae; Picrorrhozae Rhizoma] | None | Kūnmíng zhōngyào chǎng yǒuxiàn gōngsī | 4 weeks | None |
| Liang 2014b | Cāng sháo xiāoshí fāng | Decoction | Triticum aestivum L. [Gramineae; Massa Medicata Fermentata] 15 g, Paeonia lactiflora Pall. [Paeoniaceae; Paeoniae Radix Alba], Gallus gallus var. domesticus Brisson [Phasianidae; Galli Stomachichum Corium], Dioscorea batatas Dacne. [Dioscoreaceae; Dioscoreae Rhizoma], Crataegus pinnatifida Bge [Rosaceae; Crataegii Fructus], Forsythia suspensa (Thunb.) Vahl [Oleaceae; Forsythiae Fructus] 10 g, Atractylodes lancea (Thunb.) DC. [Asteraceae; Atractylodis Rhizoma], Citrus unshiu Markovich [Rutaceae; Citri Unshius Pericarpium], Raphanus sativus var. hortensis for. acanthiformis Makino [Brassicaceae; Raphani Semen] 9 g | None | Not applicable | 2 weeks | None |
| Lin 2008 | Xǐngpí jiàn wèi héjì | Decoction | Crataegus pinnatifida Bge [Rosaceae; Crataegii Fructus] 12 g, Poria cocos (Schw.) Wolf [Polyporaceae; Poria(Hoelen)], Scutellaria baicalensis Georgi [Labiatae; Scutellariae Radix], Triticum aestivum L. [Gramineae; Massa Medicata Fermentata] 10 g, Atractylodes lancea (Thunb.) DC. [Asteraceae; Atractylodis Rhizoma] 8 g, Agastache rugosa (Fisch. et Meyer) O. Kuntze [Labiatae; Agastachis Herba], Codonopsis pilosulae (Fr.) Nannf. [Campanulaceae; Codonopsis Pilosulae Radix] 6 g, Magnolia officinalis Rehder et Wilson [Magnoliaceae; Magnoliae Cortex] 5 g, Glycyrrhiza uralensis Fisch. [Leguminosae; Glycyrrhizae Radix] 3 g, Rheum palmatum L. [Polygonaceae; Rhei Rhizoma] 2 g | None | Not applicable | 2 weeks | None |
| Lin 2012 | Xiaoer Piweile granule | Granule | Dioscorea batatas Dacne. [Dioscoreaceae; Dioscoreae Rhizoma], Gallus gallus var. domesticus Brisson [Phasianidae; Galli Stomachichum Corium], Rheum palmatum L. [Polygonaceae; Rhei Rhizoma], Amomum krabanh Pierre ex Cagnep [Zingiberaceae; Amomi Rotundus Fructus], Hordeum vulgare L. [Gramineae; Hordei Fructus Germiniatus] | None | Kūnmíng bāng yǔ zhìyào yǒuxiàn gōngsī | 4 weeks | None |
| Liu 2006 | Yanshi powder | Decoction | Poria cocos (Schw.) Wolf [Polyporaceae; Poria(Hoelen)], Codonopsis pilosulae (Fr.) Nannf. [Campanulaceae; Codonopsis Pilosulae Radix], Citrus unshiu Markovich [Rutaceae; Citri Unshius Pericarpium]5 g, Atractylodes macrocepha-la Koidz [Asteraceae; Atractylodis Rhizoma Alba], Crataegus pinnatifida Bge [Rosaceae; Crataegii Fructus], Triticum aestivum L. [Gramineae; Massa Medicata Fermentata], Hordeum vulgare L. [Gramineae; Hordei Fructus Germiniatus] 4 g, Glycyrrhiza uralensis Fisch. [Leguminosae; Glycyrrhizae Radix] 3 g, Gallus gallus var. domesticus Brisson [Phasianidae; Galli Stomachichum Corium] 2 g | None | Not applicable | 14 days | None |
| Liu 2008 | Wangshi Baochi pill | Pill | Rheum palmatum L. [Polygonaceae; Rhei Rhizoma], Coptis deltoidea C.Y. Cheng et Hsiao [Ranunculaceae; Coptidis Rhizoma], Croton tiglium L. [Euphorbiaceae; Crotonis Semen], Fritillaria cirrhosa D. Don. [Liliaceae; Fritillariae Cirrhosae Bulbus], Arisaema amurenxe Maximowicz [Araceae; Arisaematis Rhizoma], Eleocharis dulcis (Burm. F.) Trin. [Cyperaceae; Eleocharitis Tuber], Zingiber officinale Rosc. [Zingiberaceae; Zingiberis Rhizoma Recens] | None | Not applicable | 1 month | 0.5 year |
| Liu 2010 | Miǎn jiān kēlì | Granule | Paeonia lactiflora Pall. [Paeoniaceae; Paeoniae Radix Alba], Codonopsis pilosulae (Fr.) Nannf. [Campanulaceae; Codonopsis Pilosulae Radix], Atractylodes macrocepha-la Koidz [Asteraceae; Atractylodis Rhizoma Alba], Dioscorea batatas Dacne. [Dioscoreaceae; Dioscoreae Rhizoma], Hordeum vulgare L. [Gramineae; Hordei Fructus Germiniatus], Crataegus pinnatifida Bge [Rosaceae; Crataegii Fructus], Dendrobium loddigesii Rolfe. [Orchidaceae; Denbrobii Herba] 10 g, Bupleurum falcatum Linne [Apiaceae; Bupleuri Radix], Citrus unshiu Markovich [Rutaceae; Citri Unshius Pericarpium], Poncirus trifoliata Rafin. [Rutaceae; Aurantii Immaturus Fructus], Triticum aestivum L. [Gramineae; Massa Medicata Fermentata] 6 g, Amomum villosum Lour. [Zingiberaceae; Amomi Fuctus] 3 g | None | Guǎngdōng yīfāng zhìyào chǎng | 30 days | 3 month |
| Liu 2011 | Xingpi Yanger granule | Granule | Gerbera piloselloides (Linn. ) Cass., Pittosporum glabratum Lindl., Emilia sonchifolia (L.) DC., Valeriana jatamansi Jones | None | Guìzhōu jiàn xìng yào yè yǒuxiàn gōngsī | TG: 14-28 days CG: 4 weeks | None |
| Liu 2012 | Qí hú zhā kēlì | Granule | Astragalus membranaceus Bunge [Leguminosae; Astragali Radix], Dioscorea batatas Dacne. [Dioscoreaceae; Dioscoreae Rhizoma], Glycyrrhiza uralensis Fisch. [Leguminosae; Glycyrrhizae Radix], Poria cocos (Schw.) Wolf [Polyporaceae; Poria(Hoelen)], Dendrobium loddigesii Rolfe. [Orchidaceae; Denbrobii Herba], Hordeum vulgare L. [Gramineae; Hordei Fructus Germiniatus], Crataegus pinnatifida Bge [Rosaceae; Crataegii Fructus], Chaenomeles sinensis Koehne [Rosaceae; Chaenomelis Fuctus], Codonopsis pilosulae (Fr.) Nannf. [Campanulaceae; Codonopsis Pilosulae Radix], Citrus unshiu Markovich [Rutaceae; Citri Unshius Pericarpium], Atractylodes macrocepha-la Koidz [Asteraceae; Atractylodis Rhizoma Alba] | None | Guìzhōu hóng qí yào yè guóyào | 21 days | None |
| Liu 2014 | Yuepi Leshi decoction | Decoction | Pseudostellaria augustifolia Y.N.Lee [Caryophyllaceae; Pseudostellariae Radix], Hordeum vulgare L. [Gramineae; Hordei Fructus Germiniatus] 15 g, Atractylodes macrocepha-la Koidz [Asteraceae; Atractylodis Rhizoma Alba], Bupleurum falcatum Linne [Apiaceae; Bupleuri Radix], Prunus mume Sieb. et Zucc [Rosaceae; Mume Fructus], Crataegus pinnatifida Bge [Rosaceae; Crataegii Fructus], Gallus gallus var. domesticus Brisson [Phasianidae; Galli Stomachichum Corium], Raphanus sativus var. hortensis for. acanthiformis Makino [Brassicaceae; Raphani Semen], Paederia scandens var. scandens (Lour.) Merr. [Rubiaceae; Paederiae Herba], Bù zhā yè, Artemisia anomala S. Moore [Asteraceae; Artemisiae Anomalae Herba] 10 g | - Obvious Qi deficiency: Wǔzhǐ máotáo 30 g, Dioscorea batatas Dacne. [Dioscoreaceae; Dioscoreae Rhizoma] 15 g - Profuse sweating: Triticum aestivum L. [Gramineae; Tritici Cimmatri Semen], Oryza sativa var. glutinosa Matsum. [Gramineae; Oryzae Radix] 15 g - Spleen deficiency and loose stools: Dolichos lablab L. [Leguminosae; Lablab Semen] 20 g, Atractylodes lancea (Thunb.) DC. [Asteraceae; Atractylodis Rhizoma] 10 g, Ligusticum chuanxiong Hort [Apiaceae; Ligustici Rhizoma] 5 g - Significant heat and humid: Huǒtàn mǔ 20 g, Gardenia jasminoides var. grandiflora (Lour.) Nakai [Rubiaceae; Gardeniae Fructus] 10 g - Obvious fire-heat: remove Atractylodes macrocepha-la Koidz [Asteraceae; Atractylodis Rhizoma Alba], add Trichosanthes kirilowii Maxim. [Cucurbitaceae; Trichosanthis Fructus] 10 g, Forsythia suspensa (Thunb.) Vahl [Oleaceae; Forsythiae Fructus], Taraxacum platycarpum H. Dahlsi [Asteraceae; Taraxci Herba] 15 g - Food stagnation: Crataegus pinnatifida Bge [Rosaceae; Crataegii Fructus], Gallus gallus var. domesticus Brisson [Phasianidae; Galli Stomachichum Corium], Raphanus sativus var. hortensis for. acanthiformis Makino [Brassicaceae; Raphani Semen] 20 g, Poncirus trifoliata Rafin. [Rutaceae; Aurantii Immaturus Fructus] 10 g - Phlegm and dampness: Citrus reticulata Blanco [Rutaceae; Citri Rubrum Exocarpium], Pinellia ternata (Thunb.) Breit. [Araceae; Pinelliae Rhizoma], Fritillaria thunbergii Miq. [Liliaceae; Fritillariae Thunbergii Bulbus] 10 g - Insufficient yin: Dendrobium loddigesii Rolfe. [Orchidaceae; Denbrobii Herba], Polygonatum sibiricum Redoute [Liliaceae; Polygonati Rhizoma] 10 g, Glehnia littoralis Fr. Schm. [Apiaceae; Glehniae Radix] 15 g - Night anxiety, night cry: Uncaria rhynchophylla (Miq.) Jacks. [Rubiaceae; Uncariae Ramulus cum Uncis] 15 g, Cryptotympana pustulata Fabricius [Cicadidae; Cicadae Periostracum] 5 g, Elephas Species [Elephantidae; Fossilia Ossis Mastodi] 30 g - Bloating, abdominal pain: Areca catechu L. [Arecaceae; Arecae Semen], Poncirus trifoliata Rafin. [Rutaceae; Aurantii Immaturus Fructus] 10 g, Paeonia lactiflora Pall. [Paeoniaceae; Paeoniae Radix Alba] 15 g - Constipation: Trichosanthes kirilowii Maxim. [Cucurbitaceae; Trichosanthis Semen] 20 g, Prunus humilis Bunge [Rosaceae; Pruni Humilis Semen] 15 g, Rheum palmatum L. [Polygonaceae; Rhei Rhizoma] 5 g | Not applicable | 4 weeks | 4 week |
| Liu 2016 | Yunpi Zengshi decoction | Granule | Atractylodes lancea (Thunb.) DC. [Asteraceae; Atractylodis Rhizoma], Poria cocos (Schw.) Wolf [Polyporaceae; Poria(Hoelen)], Raphanus sativus var. hortensis for. acanthiformis Makino [Brassicaceae; Raphani Semen], Crataegus pinnatifida Bge [Rosaceae; Crataegii Fructus], Hordeum vulgare L. [Gramineae; Hordei Fructus Germiniatus], Triticum aestivum L. [Gramineae; Massa Medicata Fermentata], Saposhnikovia divaricata Schiskin [Apiaceae; Saposhnikovia Radix] 6~10 g, Dioscorea batatas Dacne. [Dioscoreaceae; Dioscoreae Rhizoma], Dolichos lablab L. [Leguminosae; Lablab Semen], Gallus gallus var. domesticus Brisson [Phasianidae; Galli Stomachichum Corium] 8~12 g, Coix lachryma-jobi var. ma-yeun (Roman.) Stapf [Gramineae; Coicis Semen] 9~12 g, Citrus unshiu Markovich [Rutaceae; Citri Unshius Pericarpium]6~9 g, Forsythia suspensa (Thunb.) Vahl [Oleaceae; Forsythiae Fructus] 6~8 g, Amomum villosum Lour. [Zingiberaceae; Amomi Fuctus], Glycyrrhiza uralensis Fisch. [Leguminosae; Glycyrrhizae Radix] 3~6 g | - White slimy fur: remove Dolichos lablab L. [Leguminosae; Lablab Semen], Dioscorea batatas Dacne. [Dioscoreaceae; Dioscoreae Rhizoma], add Magnolia officinalis Rehder et Wilson [Magnoliaceae; Magnoliae Cortex] - Visit in summer, dampness, slimy fur, sticky slimy stool: Talcum - Abdominal distension, abdominal pain: Corydalis ternata Nakai [Papaveraceae; Corydalis (Tuber) Rhizoma] - Belching, nausea: Pinellia ternata (Thunb.) Breit. [Araceae; Pinelliae Rhizoma], Phyllostachys nigra var. henonis (Bean.) Stapf [Gramineae; Bambusae Caulis In Taeniam] - Dry stool: add Cannabis sativa L. [Cannabinaceae; Cannabis Fructus], Rheum palmatum L. [Polygonaceae; Rhei Rhizoma] | Not applicable | 30 days | None |
| Liu 2017 | Shenling Baizhu powder | Decoction | Poria cocos (Schw.) Wolf [Polyporaceae; Poria(Hoelen)], Atractylodes lancea (Thunb.) DC. [Asteraceae; Atractylodis Rhizoma] 12g, Codonopsis pilosulae (Fr.) Nannf. [Campanulaceae; Codonopsis Pilosulae Radix], Atractylodes macrocepha-la Koidz [Asteraceae; Atractylodis Rhizoma Alba] 10 g, Agastache rugosa (Fisch. et Meyer) O. Kuntze [Labiatae; Agastachis Herba], Magnolia officinalis Rehder et Wilson [Magnoliaceae; Magnoliae Cortex] 9 g, Glycyrrhiza uralensis Fisch. [Leguminosae; Glycyrrhizae Radix] 6 g, Saposhnikovia divaricata Schiskin [Apiaceae; Saposhnikovia Radix] 3 g | None | Not applicable | 12 weeks | None |
| Liu 2018a | Xingpi Xiaoshi decoction | Decoction | Pseudostellaria augustifolia Y.N.Lee [Caryophyllaceae; Pseudostellariae Radix], Poncirus trifoliata Rafin. [Rutaceae; Aurantii Immaturus Fructus], Gallus gallus var. domesticus Brisson [Phasianidae; Galli Stomachichum Corium], Tadehagi triquetrum (L.) Ohashi, Bù zhā yè, Crataegus pinnatifida Bge [Rosaceae; Crataegii Fructus], Poria cocos (Schw.) Wolf [Polyporaceae; Poria(Hoelen)] 10 g, Amomum villosum Lour. [Zingiberaceae; Amomi Fuctus], Citrus unshiu Markovich [Rutaceae; Citri Unshius Pericarpium]5 g | None | Not applicable | 4 weeks | None |
| Liu 2018b | Jianpi Xiaoji decoction | Decoction | Poria cocos (Schw.) Wolf [Polyporaceae; Poria(Hoelen)], Codonopsis pilosulae (Fr.) Nannf. [Campanulaceae; Codonopsis Pilosulae Radix], Atractylodes macrocepha-la Koidz [Asteraceae; Atractylodis Rhizoma Alba], Triticum aestivum L. [Gramineae; Massa Medicata Fermentata], Hordeum vulgare L. [Gramineae; Hordei Fructus Germiniatus] 10 g, Citrus aurantium L. [Rutaceae; Aurantii Fructus Pericarpium], Paeonia lactiflora Pall. [Paeoniaceae; Paeoniae Radix Alba], Bupleurum falcatum Linne [Apiaceae; Bupleuri Radix] 6 g, Glycyrrhiza uralensis Fisch. [Leguminosae; Glycyrrhizae Radix], Amomum villosum Lour. [Zingiberaceae; Amomi Fuctus] 3 g, Rheum palmatum L. [Polygonaceae; Rhei Rhizoma] 2 g | None | Not applicable | 2 weeks | None |
| Luo 2014 | Shuāng píng sàn | Decoction | Astragalus membranaceus Bunge [Leguminosae; Astragali Radix], Bupleurum falcatum Linne [Apiaceae; Bupleuri Radix], Codonopsis pilosulae (Fr.) Nannf. [Campanulaceae; Codonopsis Pilosulae Radix], Atractylodes macrocepha-la Koidz [Asteraceae; Atractylodis Rhizoma Alba], Hordeum vulgare L. [Gramineae; Hordei Fructus Germiniatus] 10 g, Poncirus trifoliata Rafin. [Rutaceae; Aurantii Immaturus Fructus], Saposhnikovia divaricata Schiskin [Apiaceae; Saposhnikovia Radix], Paeonia lactiflora Pall. [Paeoniaceae; Paeoniae Radix Alba], Glycyrrhiza uralensis Fisch. [Leguminosae; Glycyrrhizae Radix] 6 g | - Pale complexion, pale lips: Astagalus membranaceus Bunge [Leguminosae; Astragali Radix], Equus asinus L. [Equidae; Asini Gelatinum], Zizyphus jujuba ver. inermis Rehder [Rhamnaceae; Zizyphi Fructus] - Night anxiety, Irritation, easy to startle, palm and plantar fever: remove Bupleurum falcatum Linne [Apiaceae; Bupleuri Radix], add Androsace umbellata (Lour.) Merr. [Primulacae; Stellariae Radix], Coptis deltoidea C.Y. Cheng et Hsiao [Ranunculaceae; Coptidis Rhizoma], Artemisiae apiacea Hance [Asteraceae; Artemisiae Apiaceae Herba] - Thick slimy fur, bad fetid mouth odor: remove Atractylodes macrocepha-la Koidz [Asteraceae; Atractylodis Rhizoma Alba], add Atractylodes lancea (Thunb.) DC. [Asteraceae; Atractylodis Rhizoma], Raphanus sativus var. hortensis for. acanthiformis Makino [Brassicaceae; Raphani Semen], Magnolia officinalis Rehder et Wilson [Magnoliaceae; Magnoliae Cortex] - Dry mouth, red tongue, less fur or no fur: Trichosanthes kirilowii Maxim. [Cucurbitaceae; Trichosanthis Fructus], Dolichos lablab L. [Leguminosae; Lablab Semen], Coix lachryma-jobi var. ma-yeun (Roman.) Stapf [Gramineae; Coicis Semen] | Not applicable | 4 weeks | None |
| Ma 2011 | Zhuangerling decoction | Decoction | Codonopsis pilosulae (Fr.) Nannf. [Campanulaceae; Codonopsis Pilosulae Radix], Poria cocos (Schw.) Wolf [Polyporaceae; Poria(Hoelen)], Atractylodes macrocepha-la Koidz [Asteraceae; Atractylodis Rhizoma Alba], Glycyrrhiza uralensis Fisch. [Leguminosae; Glycyrrhizae Radix], Citrus unshiu Markovich [Rutaceae; Citri Unshius Pericarpium], Agastache rugosa (Fisch. et Meyer) O. Kuntze [Labiatae; Agastachis Herba], Magnolia officinalis Rehder et Wilson [Magnoliaceae; Magnoliae Cortex], Gallus gallus var. domesticus Brisson [Phasianidae; Galli Stomachichum Corium], Triticum aestivum L. [Gramineae; Massa Medicata Fermentata], Crataegus pinnatifida Bge [Rosaceae; Crataegii Fructus] | None | Shǎnxī shěng yán'ān shì zhōng yīyuàn tígōng | 4 weeks | None |
| Ma 2017 | Yangyin Yunpi granule | Granule | Dioscorea batatas Dacne. [Dioscoreaceae; Dioscoreae Rhizoma] 10~15 g, Dendrobium loddigesii Rolfe. [Orchidaceae; Denbrobii Herba], Pseudostellaria augustifolia Y.N.Lee [Caryophyllaceae; Pseudostellariae Radix], Paeonia lactiflora Pall. [Paeoniaceae; Paeoniae Radix Alba], Poria cocos (Schw.) Wolf [Polyporaceae; Poria(Hoelen)], Atractylodes macrocepha-la Koidz [Asteraceae; Atractylodis Rhizoma Alba], Scutellaria baicalensis Georgi [Labiatae; Scutellariae Radix], Crataegus pinnatifida Bge [Rosaceae; Crataegii Fructus], Pueraria thunbergiana Benth. [Leguminosae; Puerariae Radix] 6~10 g, Aucklandia lappa Decne [Asteraceae; Aucklandiae Radix] 4~6 g, Syzygium aromaticum Merr et Perry [Myrtaceac; Syzygii Flos] 2~3 g | - Excessive sweating: Triticum aestivum L. [Gramineae; Tritici Cimmatri Semen], Ostrea gigas Thunb. [Ostreidae; Ostreae Concha] - Impatient temperament: Bupleurum falcatum Linne [Apiaceae; Bupleuri Radix], Curcuma aromatica Salisb. [Zingiberaceae; Curcumae Radix] - Dampness-heat obstruction: Coptis deltoidea C.Y. Cheng et Hsiao [Ranunculaceae; Coptidis Rhizoma], Coix lachryma-jobi var. ma-yeun (Roman.) Stapf [Gramineae; Coicis Semen] - Food accumulation: Gallus gallus var. domesticus Brisson [Phasianidae; Galli Stomachichum Corium], Crataegus pinnatifida Bge [Rosaceae; Crataegii Fructus], Triticum aestivum L. [Gramineae; Massa Medicata Fermentata], Hordeum vulgare L. [Gramineae; Hordei Fructus Germiniatus] - Dry stool: Trichosanthes kirilowii Maxim. [Cucurbitaceae; Trichosanthis Fructus], Rheum palmatum L. [Polygonaceae; Rhei Rhizoma] | Běijīng kāngrén táng yào yè yǒuxiàn gōngsī tígōng pèifāng kēlì | 4 weeks | 1 month |
| Meng 2020 | Tiaopi Hezhong granule | Granule | Agastache rugosa (Fisch. et Meyer) O. Kuntze [Labiatae; Agastachis Herba], Atractylodes macrocepha-la Koidz [Asteraceae; Atractylodis Rhizoma Alba], Gardenia jasminoides var. grandiflora (Lour.) Nakai [Rubiaceae; Gardeniae Fructus], Phyllostachys nigra var. henonis (Bean.) Stapf [Gramineae; Bambusae Caulis In Taeniam], Citrus unshiu Markovich [Rutaceae; Citri Unshius Pericarpium], Perilla frutescens var. acuta Kudo [Labiatae; Perilliae Semen], Picrorrhiza scrophulariiflora Pennell [Scrophulariaceae; Picrorrhozae Rhizoma], Selaginella tamariscina (Beauv.) Spring [Selaginellaceae; Selaginelliae Herba], Poria cocos (Schw.) Wolf [Polyporaceae; Poria(Hoelen)], Prunus percisa (L.) Batsch [Rosaceae; Persicae Semen], Gallus gallus var. domesticus Brisson [Phasianidae; Galli Stomachichum Corium], Glycyrrhiza uralensis Fisch. [Leguminosae; Glycyrrhizae Radix] | None | Jìn yào zhì zì  (AZ200802-81) | 8 weeks | None |
| Pan 2006 | Èr jīn qǐ pí yǐn | Decoction | Striga asiatica (L.) O. Kuntze 15 g, Bupleurum falcatum Linne [Apiaceae; Bupleuri Radix], Paeonia lactiflora Pall. [Paeoniaceae; Paeoniae Radix Alba], Gallus gallus var. domesticus Brisson [Phasianidae; Galli Stomachichum Corium], Poria cocos (Schw.) Wolf [Polyporaceae; Poria(Hoelen)] 5 g, Codonopsis pilosulae (Fr.) Nannf. [Campanulaceae; Codonopsis Pilosulae Radix], Atractylodes macrocepha-la Koidz [Asteraceae; Atractylodis Rhizoma Alba], Dioscorea batatas Dacne. [Dioscoreaceae; Dioscoreae Rhizoma], Hordeum vulgare L. [Gramineae; Hordei Fructus Germiniatus], Bù zhā yè 10 g, Citrus unshiu Markovich [Rutaceae; Citri Unshius Pericarpium], Glycyrrhiza uralensis Fisch. [Leguminosae; Glycyrrhizae Radix] 3 g | None | Not applicable | 4 weeks | None |
| Peng 2008 | Qīwèi báizhú sàn | Decoction | Codonopsis pilosulae (Fr.) Nannf. [Campanulaceae; Codonopsis Pilosulae Radix], Poria cocos (Schw.) Wolf [Polyporaceae; Poria(Hoelen)], Atractylodes macrocepha-la Koidz [Asteraceae; Atractylodis Rhizoma Alba], Agastache rugosa (Fisch. et Meyer) O. Kuntze [Labiatae; Agastachis Herba], Pueraria thunbergiana Benth. [Leguminosae; Puerariae Radix], Gallus gallus var. domesticus Brisson [Phasianidae; Galli Stomachichum Corium] 10 g, Aucklandia lappa Decne [Asteraceae; Aucklandiae Radix], Amomum villosum Lour. [Zingiberaceae; Amomi Fuctus], Glycyrrhiza uralensis Fisch. [Leguminosae; Glycyrrhizae Radix] 5 g | - Repeated common cold: Astragalus membranaceus Bunge [Leguminosae; Astragali Radix] 10 g - Abdominal pain with parasitic diseases: Areca catechu L. [Arecaceae; Arecae Semen] 10 g - Loose stool: Huáishān 10 g - Constipation: Raphanus sativus var. hortensis for. acanthiformis Makino [Brassicaceae; Raphani Semen] 10 g | Not applicable | 2 weeks | None |
| Peng 2015 | Yunpi powder | Decoction | Atractylodes lancea (Thunb.) DC. [Asteraceae; Atractylodis Rhizoma], Atractylodes macrocepha-la Koidz [Asteraceae; Atractylodis Rhizoma Alba], Poria cocos (Schw.) Wolf [Polyporaceae; Poria(Hoelen)] 9 g, Dioscorea batatas Dacne. [Dioscoreaceae; Dioscoreae Rhizoma], Coix lachryma-jobi var. ma-yeun (Roman.) Stapf [Gramineae; Coicis Semen], Citrus unshiu Markovich [Rutaceae; Citri Unshius Pericarpium], Citrus aurantium L. [Rutaceae; Aurantii Fructus Pericarpium], Triticum aestivum L. [Gramineae; Massa Medicata Fermentata], Glycyrrhiza uralensis Fisch. [Leguminosae; Glycyrrhizae Radix] 6 g | None | Not applicable | 15 days | None |
| Pi 2011 | Chàng zhōng yǐn | Decoction | Pseudostellaria augustifolia Y.N.Lee [Caryophyllaceae; Pseudostellariae Radix], Citrus unshiu Markovich [Rutaceae; Citri Unshius Pericarpium], Gallus gallus var. domesticus Brisson [Phasianidae; Galli Stomachichum Corium], Poncirus trifoliata Rafin. [Rutaceae; Aurantii Immaturus Fructus], Amomum villosum Lour. [Zingiberaceae; Amomi Fuctus], Crataegus pinnatifida Bge [Rosaceae; Crataegii Fructus] 3~6 g, Triticum aestivum L. [Gramineae; Massa Medicata Fermentata], Hordeum vulgare L. [Gramineae; Hordei Fructus Germiniatus] 5~10 g | None | Not applicable | 1-2 months | None |
| Qi 2009 | Jiàn pí kāiwèi sàn or Yǎng yīn kāiwèi sàn | Decoction | - Jiàn pí kāiwèi sàn: Hordeum vulgare L. [Gramineae; Hordei Fructus Germiniatus] 10 g, Paeonia lactiflora Pall. [Paeoniaceae; Paeoniae Radix Alba] 9 g, Glycyrrhiza uralensis Fisch. [Leguminosae; Glycyrrhizae Radix], Selaginella tamariscina (Beauv.) Spring [Selaginellaceae; Selaginelliae Herba], Citrus medica var. medica 6 g, Benincasa cerifera Savi [Cucurbitaceae; Benincasae Semen] 3 g, Nelumbo nucifera Gaertner [Nymphaceae; Nelumbinis Foluim] 4 g - Yǎng yīn kāiwèi sàn: Liriope platyphylla Wang et Tang [Liliaceae; Liriopes Radix], Polygonatum odoratum var. pluriflorum Ohwi [Liliaceae; Polygonati Dodrati Rhizoma], Adenophora triphylla var. japonica Hara [Campanulaceae; Adenophorae Radix], Nelumbo nucifera Gaertner [Nymphaceae; Nelumbinis Foluim] 10 g, Dendrobium loddigesii Rolfe. [Orchidaceae; Denbrobii Herba], Hordeum vulgare L. [Gramineae; Hordei Fructus Germiniatus] 7.5 g, Paeonia suffruticosa Andrews [Ranunculaceae; Moutan Cortex] 4 g | None | Not applicable | 4 weeks | None |
| Qin 2012 | Xingpi Yanger granule | Granule | Emilia sonchifolia (L.) DC., Gerbera piloselloides (Linn. ) Cass., Pittosporum glabratum Lindl., Valeriana jatamansi Jones | None | NR | 15 days | None |
| Qiu 2011 | Jiàn pí hé wèi tāng | Decoction | Panax ginseng C. A. Mey. [Araliaceae; Ginseng Radix], Atractylodes macrocepha-la Koidz [Asteraceae; Atractylodis Rhizoma Alba], Amomum villosum Lour. [Zingiberaceae; Amomi Fuctus], Magnolia officinalis Rehder et Wilson [Magnoliaceae; Magnoliae Cortex], Poria cocos (Schw.) Wolf [Polyporaceae; Poria(Hoelen)], Crataegus pinnatifida Bge [Rosaceae; Crataegii Fructus], Triticum aestivum L. [Gramineae; Massa Medicata Fermentata] 5 g, Raphanus sativus var. hortensis for. acanthiformis Makino [Brassicaceae; Raphani Semen], Glycyrrhiza uralensis Fisch. [Leguminosae; Glycyrrhizae Radix] 2 g | None | Not applicable | 2 weeks | None |
| Qiu 2017 | Jianpi Xiaoji decoction | Decoction | Codonopsis pilosulae (Fr.) Nannf. [Campanulaceae; Codonopsis Pilosulae Radix], Atractylodes macrocepha-la Koidz [Asteraceae; Atractylodis Rhizoma Alba], Poria cocos (Schw.) Wolf [Polyporaceae; Poria(Hoelen)], Dioscorea batatas Dacne. [Dioscoreaceae; Dioscoreae Rhizoma], Amomum villosum Lour. [Zingiberaceae; Amomi Fuctus], Crataegus pinnatifida Bge [Rosaceae; Crataegii Fructus], Triticum aestivum L. [Gramineae; Massa Medicata Fermentata] | None | Not applicable | 4 weeks | None |
| She 2004 | Shenling Baizhu powder | Decoction | Codonopsis pilosulae (Fr.) Nannf. [Campanulaceae; Codonopsis Pilosulae Radix], Astragalus membranaceus Bunge [Leguminosae; Astragali Radix] 12 g, Atractylodes macrocepha-la Koidz [Asteraceae; Atractylodis Rhizoma Alba], Dolichos lablab L. [Leguminosae; Lablab Semen] 10 g, Hordeum vulgare L. [Gramineae; Hordei Fructus Germiniatus], Coix lachryma-jobi var. ma-yeun (Roman.) Stapf [Gramineae; Coicis Semen] 15 g, Gallus gallus var. domesticus Brisson [Phasianidae; Galli Stomachichum Corium] 8 g, Amomum villosum Lour. [Zingiberaceae; Amomi Fuctus] 3 g | None | Not applicable | 2 weeks | None |
| Shi 2020 | Wangshi Baochi pill | Pill | Rheum palmatum L. [Polygonaceae; Rhei Rhizoma], Coptis deltoidea C.Y. Cheng et Hsiao [Ranunculaceae; Coptidis Rhizoma], Croton tiglium L. [Euphorbiaceae; Crotonis Semen], Fritillaria cirrhosa D. Don. [Liliaceae; Fritillariae Cirrhosae Bulbus], Arisaema amurenxe Maximowicz [Araceae; Arisaematis Rhizoma], Zingiber officinale Rosc. [Zingiberaceae; Zingiberis Rhizoma Siccus] | None | Jīnghuá zhìyào jítuán gǔfèn yǒuxiàn gōngsī | 2 weeks | None |
| Su 2015 | Shenling Baizhu powder | Decoction | Hordeum vulgare L. [Gramineae; Hordei Fructus Germiniatus], Oryza sativa L. [Gramineae; Oryzae Fructus Germinatus] 14 g, Poria cocos (Schw.) Wolf [Polyporaceae; Poria(Hoelen)] 10 g, Crataegus pinnatifida Bge [Rosaceae; Crataegii Fructus] 9 g, Forsythia suspensa (Thunb.) Vahl [Oleaceae; Forsythiae Fructus] 8 g, Codonopsis pilosulae (Fr.) Nannf. [Campanulaceae; Codonopsis Pilosulae Radix], Citrus unshiu Markovich [Rutaceae; Citri Unshius Pericarpium], Gallus gallus var. domesticus Brisson [Phasianidae; Galli Stomachichum Corium], Atractylodes macrocepha-la Koidz [Asteraceae; Atractylodis Rhizoma Alba] 7 g, Atractylodes lancea (Thunb.) DC. [Asteraceae; Atractylodis Rhizoma] 6 g, Dioscorea batatas Dacne. [Dioscoreaceae; Dioscoreae Rhizoma], Triticum aestivum L. [Gramineae; Massa Medicata Fermentata], Glycyrrhiza uralensis Fisch. [Leguminosae; Glycyrrhizae Radix] 5 g | None | Not applicable | 2 weeks | None |
| Sun 2009 | TG1: Baobaole decoction TG2 : Erbao granula | TG1: Decoction TG2: Granule | - Baobaole decoction: Atractylodes lancea (Thunb.) DC. [Asteraceae; Atractylodis Rhizoma], Crataegus pinnatifida Bge [Rosaceae; Crataegii Fructus], Astragalus membranaceus Bunge [Leguminosae; Astragali Radix], Codonopsis pilosulae (Fr.) Nannf. [Campanulaceae; Codonopsis Pilosulae Radix], Citrus unshiu Markovich [Rutaceae; Citri Unshius Pericarpium]10 g, Paeonia lactiflora Pall. [Paeoniaceae; Paeoniae Radix Alba], Cassia tora L. [Leguminosae; Cassiae Semen], Elephas Species [Elephantidae; Fossilia Ossis Mastodi], Ostrea gigas Thunb. [Ostreidae; Ostreae Concha] 20 g - Erbao granula: Atractylodes lancea (Thunb.) DC. [Asteraceae; Atractylodis Rhizoma], Citrus unshiu Markovich [Rutaceae; Citri Unshius Pericarpium], Gallus gallus var. domesticus Brisson [Phasianidae; Galli Stomachichum Corium], Crataegus pinnatifida Bge [Rosaceae; Crataegii Fructus] | None | Not applicable | 2 weeks | None |
| Sun 2012 | Yigong powder | Decoction | Atractylodes macrocepha-la Koidz [Asteraceae; Atractylodis Rhizoma Alba], Crataegus pinnatifida Bge [Rosaceae; Crataegii Fructus], Triticum aestivum L. [Gramineae; Massa Medicata Fermentata], Hordeum vulgare L. [Gramineae; Hordei Fructus Germiniatus], Gallus gallus var. domesticus Brisson [Phasianidae; Galli Stomachichum Corium] 9 g, Poria cocos (Schw.) Wolf [Polyporaceae; Poria(Hoelen)], Pseudostellaria augustifolia Y.N.Lee [Caryophyllaceae; Pseudostellariae Radix], Glycyrrhiza uralensis Fisch. [Leguminosae; Glycyrrhizae Radix], Citrus unshiu Markovich [Rutaceae; Citri Unshius Pericarpium]6 g | - Spleen-liver disharmony: Agastache rugosa (Fisch. et Meyer) O. Kuntze [Labiatae; Agastachis Herba] 9 g, Raphanus sativus var. hortensis for. acanthiformis Makino [Brassicaceae; Raphani Semen] 6 g - Spleen stomach qi deficiency: Dioscorea batatas Dacne. [Dioscoreaceae; Dioscoreae Rhizoma] 9 g, Codonopsis pilosulae (Fr.) Nannf. [Campanulaceae; Codonopsis Pilosulae Radix] 6 g - Spleen stomach yin deficiency: Rehmannia glutinosa var. purpurea (Makino) Makino et Nemoto [Scrophulariaceae; Rehmanniae Radix] 9 g, Dendrobium loddigesii Rolfe. [Orchidaceae; Denbrobii Herba] 6 g - Effulgent liver and spleen deficiency: Baphicacanthus cusia (Nees) Bremek [Acanthaceae; Indigo Pulcerata Levis], Syzygium aromaticum Merr et Perry [Myrtaceac; Syzygii Flos] 6 g | Not applicable | 45 days | None |
| Sun 2015 | Xiāo yáo sàn | Decoction | Bupleurum falcatum Linne [Apiaceae; Bupleuri Radix], Paeonia lactiflora Pall. [Paeoniaceae; Paeoniae Radix Alba], Atractylodes macrocepha-la Koidz [Asteraceae; Atractylodis Rhizoma Alba] 9 g, Astagalus membranaceus Bunge [Leguminosae; Astragali Radix], Poria cocos (Schw.) Wolf [Polyporaceae; Poria(Hoelen)], Paeonia suffruticosa Andrews [Ranunculaceae; Moutan Cortex] 6 g, Gardenia jasminoides var. grandiflora (Lour.) Nakai [Rubiaceae; Gardeniae Fructus], Glycyrrhiza uralensis Fisch. [Leguminosae; Glycyrrhizae Radix], Mentha arvensis var. piperascens Makinv. [Labiatae; Menthae Herba] 3 g, Zingiber officinale Rosc. [Zingiberaceae; Zingiberis Rhizoma Recens] 3 | - Liver depression: increase Bupleurum falcatum Linne [Apiaceae; Bupleuri Radix], add Aucklandia lappa Decne [Asteraceae; Aucklandiae Radix], Citrus unshiu Markovich [Rutaceae; Citri Unshius Pericarpium]6 g - Obvious liver fire: increase Gardenia jasminoides var. grandiflora (Lour.) Nakai [Rubiaceae; Gardeniae Fructus], add Phellodendron amurense Rupr. [Rutaceae; Phellodenderi Cortex] 6 g, Cassia tora L. [Leguminosae; Cassiae Semen] 9 g  - Yin deficiency: remove Bupleurum falcatum Linne [Apiaceae; Bupleuri Radix], add Artemisia capillaris Thunb. [Asteraceae; Artemisiae Capillaris Herba] 12 g, Polygonatum odoratum var. pluriflorum Ohwi [Liliaceae; Polygonati Odorati Rhizoma], Dendrobium loddigesii Rolfe. [Orchidaceae; Denbrobii Herba] 6 g - Qi deficiency: Astragalus membranaceus Bunge [Leguminosae; Astragali Radix] 12 g, Codonopsis pilosulae (Fr.) Nannf. [Campanulaceae; Codonopsis Pilosulae Radix] 9 g | Not applicable | 4 weeks | None |
| Sun 2019 | Xiaoer Jianpi Kaiwei decoction | Decoction | Atractylodes macrocepha-la Koidz [Asteraceae; Atractylodis Rhizoma Alba], Codonopsis pilosulae (Fr.) Nannf. [Campanulaceae; Codonopsis Pilosulae Radix], Citrus unshiu Markovich [Rutaceae; Citri Unshius Pericarpium], Dioscorea batatas Dacne. [Dioscoreaceae; Dioscoreae Rhizoma] | None | Guǎngxī huān bǎo yào yè yǒuxiàn gōngsī | 30 days | None |
| Sun 2020 | Xingpi Yanger granule | Granule | Emilia sonchifolia (L.) DC., Gerbera piloselloides (Linn. ) Cass., Pittosporum glabratum Lindl., Valeriana jatamansi Jones | None | Guìzhōu jiàn xìng yào yè yǒuxiàn gōngsī shēngchǎn | 1 month | None |
| Tang 2005 | Xingpi Yanger granule | Granule | Emilia sonchifolia (L.) DC., Gerbera piloselloides (Linn. ) Cass., Pittosporum glabratum Lindl., Valeriana jatamansi Jones | None | Guìzhōu jiàn xìng yào yè yǒuxiàn gōngsī shēngchǎn | 4 weeks | None |
| Tao 2011 | Jianpi pill | Pill | Codonopsis pilosulae (Fr.) Nannf. [Campanulaceae; Codonopsis Pilosulae Radix], Atractylodes macrocepha-la Koidz [Asteraceae; Atractylodis Rhizoma Alba], Dioscorea batatas Dacne. [Dioscoreaceae; Dioscoreae Rhizoma], Citrus unshiu Markovich [Rutaceae; Citri Unshius Pericarpium], Poncirus trifoliata Rafin. [Rutaceae; Aurantii Immaturus Fructus], Triticum aestivum L. [Gramineae; Massa Medicata Fermentata], Hordeum vulgare L. [Gramineae; Hordei Fructus Germiniatus], Crataegus pinnatifida Bge [Rosaceae; Crataegii Fructus] | None | Hénán shěng wǎn xī zhìyào gǔfèn yǒuxiàn gōngsī | 2 weeks | None |
| Wang 2010 | tòng xiè yào fāng | Decoction | Paeonia lactiflora Pall. [Paeoniaceae; Paeoniae Radix Alba], Atractylodes macrocepha-la Koidz [Asteraceae; Atractylodis Rhizoma Alba], Saposhnikovia divaricata Schiskin [Apiaceae; Saposhnikovia Radix], Citrus unshiu Markovich [Rutaceae; Citrii Unshiu Immaturi Pericarpium], Atractylodes lancea (Thunb.) DC. [Asteraceae; Atractylodis Rhizoma], Citrus unshiu Markovich [Rutaceae; Citri Unshius Pericarpium]6 g, Hordeum vulgare L. [Gramineae; Hordei Fructus Germiniatus] 9 g, Uncaria sinensis (Oliv.) Havil. [Rubiaceae; Uncariae Ramulus et Uncus], Glycyrrhiza uralensis Fisch. [Leguminosae; Glycyrrhizae Radix] 5 g, Coix lachryma-jobi var. ma-yeun (Roman.) Stapf [Gramineae; Coicis Semen] 12 g | - Yin deficiency and interior heat: Artemisiae apiacea Hance {Asteraceae; Artemisiae Apiaceae Herba] - Qi deficiency: Codonopsis pilosulae (Fr.) Nannf. [Campanulaceae; Codonopsis Pilosulae Radix] - Abdominal pain: Bupleurum falcatum Linne [Apiaceae; Bupleuri Radix] | Not applicable | 4 weeks | None |
| Wang 2012a | Lǐ pí yīn zhèng fāng | Decoction | Pseudostellaria augustifolia Y.N.Lee [Caryophyllaceae; Pseudostellariae Radix] 5~10 g, Homo sapiens L. [Hominidae; Mominis Placenta] 3~5 g, Paeonia lactiflora Pall. [Paeoniaceae; Paeoniae Radix Alba] 6~8 g, Dioscorea batatas Dacne. [Dioscoreaceae; Dioscoreae Rhizoma], Nelumbo nucifera Gaertner [Nymphaceae; Nelumbinis Semen] 8~15 g, Poria cocos (Schw.) Wolf [Polyporaceae; Poria(Hoelen)], Citrus unshiu Markovich [Rutaceae; Citri Unshius Pericarpium]6~10 g, Dolichos lablab L. [Leguminosae; Lablab Semen], Oryza sativa L. [Gramineae; Arida Oryza Distillata] 10~15 g, Nelumbo nucifera Gaertn. 3~6 g, Glycyrrhiza uralensis Fisch. [Leguminosae; Glycyrrhizae Radix] 3 g | - Obvious food injury: Gallus gallus var. domesticus Brisson [Phasianidae; Galli Stomachichum Corium], Crataegus pinnatifida Bge [Rosaceae; Crataegii Fructus], Hordeum vulgare L. [Gramineae; Hordei Fructus Germiniatus], Triticum aestivum L. [Gramineae; Massa Medicata Fermentata] - Yin injury: Rehmannia glutinosa var. purpurea (Makino) Makino et Nemoto [Scrophulariaceae; Rehmanniae Radix], Dendrobium loddigesii Rolfe. [Orchidaceae; Denbrobii Herba], Zizyphus jujuba Mill [Rhamnaceae; Zizyphi Spinosae Semen], Paeonia lactiflora Pall. [Paeoniaceae; Paeoniae Radix Alba] - Obvious abdominal distension: Coix lachryma-jobi var. ma-yeun (Roman.) Stapf [Gramineae; Coicis Semen], Amomum villosum Lour. [Zingiberaceae; Amomi Fuctus], Dolichos lablab L. [Leguminosae; Lablab Semen], Poria cocos (Schw.) Wolf [Polyporaceae; Poria(Hoelen)] | Not applicable | 4 weeks | None |
| Wang 2012b | Jianweibao granule | Granule | Atractylodes lancea (Thunb.) DC. [Asteraceae; Atractylodis Rhizoma], Atractylodes macrocepha-la Koidz [Asteraceae; Atractylodis Rhizoma Alba], Crataegus pinnatifida Bge [Rosaceae; Crataegii Fructus], Gallus gallus var. domesticus Brisson [Phasianidae; Galli Stomachichum Corium], Citrus unshiu Markovich [Rutaceae; Citri Unshius Pericarpium], Pseudostellaria augustifolia Y.N.Lee [Caryophyllaceae; Pseudostellariae Radix] | None | Not applicable | 4 weeks | None |
| Wang 2012c | Lǐ pí ān zhōng tāng | Decoction | Panax ginseng C. A. Mey. [Araliaceae; Ginseng Radix] 9 g, Atractylodes macrocepha-la Koidz [Asteraceae; Atractylodis Rhizoma Alba], Poria cocos (Schw.) Wolf [Polyporaceae; Poria(Hoelen)], Coix lachryma-jobi var. ma-yeun (Roman.) Stapf [Gramineae; Coicis Semen], Dioscorea batatas Dacne. [Dioscoreaceae; Dioscoreae Rhizoma], Atractylodes lancea (Thunb.) DC. [Asteraceae; Atractylodis Rhizoma], Crataegus pinnatifida Bge [Rosaceae; Crataegii Fructus], Gallus gallus var. domesticus Brisson [Phasianidae; Galli Stomachichum Corium] 10 g, Citrus unshiu Markovich [Rutaceae; Citri Unshius Pericarpium], Magnolia officinalis Rehder et Wilson [Magnoliaceae; Magnoliae Cortex], Glycyrrhiza uralensis Fisch. [Leguminosae; Glycyrrhizae Radix] 6 g | None | Not applicable | 4 weeks | None |
| Wang 2012d | Er kāng tángjiāng | Decoction | Pseudostellaria augustifolia Y.N.Lee [Caryophyllaceae; Pseudostellariae Radix], Paeonia lactiflora Pall. [Paeoniaceae; Paeoniae Radix Alba], Poria cocos (Schw.) Wolf [Polyporaceae; Poria(Hoelen)] 5~10 g, Ostrea gigas Thunb. [Ostreidae; Ostreae Concha] 3~9 g, Hordeum vulgare L. [Gramineae; Hordei Fructus Germiniatus], Crataegus pinnatifida Bge [Rosaceae; Crataegii Fructus], Gallus gallus var. domesticus Brisson [Phasianidae; Galli Stomachichum Corium] 6~9 g, Citrus unshiu Markovich [Rutaceae; Citri Unshius Pericarpium], Coix lachryma-jobi var. ma-yeun (Roman.) Stapf [Gramineae; Coicis Semen], Glycyrrhiza uralensis Fisch. [Leguminosae; Glycyrrhizae Radix] 3~5 g | - White slimy fur: Atractylodes lancea (Thunb.) DC. [Asteraceae; Atractylodis Rhizoma], Dolichos lablab L. [Leguminosae; Lablab Semen] 6~9 g - Sloppy stool: Zingiber officinale Rosc. [Zingiberaceae; Zingiberis Rhizoma Siccus], Myristica fragrans Houtt. [Myristicaceae; Myristicae Semen] 6~9 g - Abdominal distention: remove Glycyrrhiza uralensis Fisch [Leguminosae; Glycyrrhizae Radix], add Aucklandia lappa Decne [Asteraceae; Aucklandiae Radix] 5~10 g, Cyperus rotundus L. [Cyperaceae; Cyperi Rhizoma] 6~9 g - Profuse sweating and easy to catch a cold: increase Ostrea gigas Thunb. [Ostreidae; Ostreae Concha], add Astragalus membranaceus Bunge [Leguminosae; Astragali Radix], Saposhnikovia divaricata Schiskin [Apiaceae; Saposhnikovia Radix] 3~9 g - Emotional depression: Bupleurum falcatum Linne [Apiaceae; Bupleuri Radix] 5~10 g, Selaginella tamariscina (Beauv.) Spring [Selaginellaceae; Selaginelliae Herba] 3~9 g | Not applicable | 30 days | None |
| Wang 2014 | Jianpi qinghua decoction | Decoction | Poria cocos (Schw.) Wolf [Polyporaceae; Poria(Hoelen)], Atractylodes macrocepha-la Koidz [Asteraceae; Atractylodis Rhizoma Alba] 10 g, Gallus gallus var. domesticus Brisson [Phasianidae; Galli Stomachichum Corium], Crataegus pinnatifida Bge [Rosaceae; Crataegii Fructus], Codonopsis pilosulae (Fr.) Nannf. [Campanulaceae; Codonopsis Pilosulae Radix], Coix lachryma-jobi var. ma-yeun (Roman.) Stapf [Gramineae; Coicis Semen], Artemisia capillaris Thunb. [Asteraceae; Artemisiae Capillaris Herba] 5 g, Whole grain 3 g, Picrorrhiza scrophulariiflora Pennell [Scrophulariaceae; Picrorrhozae Rhizoma], Glycyrrhiza uralensis Fisch. [Leguminosae; Glycyrrhizae Radix] 2 g | None | Not applicable | 2 weeks | None |
| Wang 2015a | No name | Decoction | Picrorrhiza scrophulariiflora Pennell [Scrophulariaceae; Picrorrhozae Rhizoma] 20 g, Aucklandia lappa Decne [Asteraceae; Aucklandiae Radix], Citrus aurantium L. [Rutaceae; Aurantii Fructus Pericarpium], Citrus unshiu Markovich [Rutaceae; Citri Unshius Pericarpium], Citrus unshiu Markovich [Rutaceae; Citrii Unshiu Immaturi Pericarpium] 15 g, Curcuma zedoaria Rocs. [Zingiberaceae; Zedoariae Rhizoma], Scirpus flaviatilis (Torr.) A. Gray [Sparganiaceae; Scirpi Rhizoma], Triticum aestivum L. [Gramineae; Massa Medicata Fermentata], Hordeum vulgare L. [Gramineae; Hordei Fructus Germiniatus], Oryza sativa L. [Gramineae; Oryzae Fructus Germinatus], Raphanus sativus var. hortensis for. acanthiformis Makino [Brassicaceae; Raphani Semen] 10 g | None | Not applicable | 2 weeks | None |
| Wang 2015b | Children Xiangju pill | Pill | Aucklandia lappa Decne [Asteraceae; Aucklandiae Radix], Citrus unshiu Markovich [Rutaceae; Citri Unshius Pericarpium], Atractylodes lancea (Thunb.) DC. [Asteraceae; Atractylodis Rhizoma], Atractylodes macrocepha-la Koidz [Asteraceae; Atractylodis Rhizoma Alba], Poria cocos (Schw.) Wolf [Polyporaceae; Poria(Hoelen)], Glycyrrhiza uralensis Fisch. [Leguminosae; Glycyrrhizae Radix], Dolichos lablab L. [Leguminosae; Lablab Semen], Dioscorea batatas Dacne. [Dioscoreaceae; Dioscoreae Rhizoma], Nelumbo nucifera Gaertner [Nymphaceae; Nelumbinis Semen], Coix lachryma-jobi var. ma-yeun (Roman.) Stapf [Gramineae; Coicis Semen], Crataegus pinnatifida Bge [Rosaceae; Crataegii Fructus], Hordeum vulgare L. [Gramineae; Hordei Fructus Germiniatus], Triticum aestivum L. [Gramineae; Massa Medicata Fermentata], Magnolia officinalis Rehder et Wilson [Magnoliaceae; Magnoliae Cortex], Poncirus trifoliata Rafin. [Rutaceae; Aurantii Immaturus Fructus], Cyperus rotundus L. [Cyperaceae; Cyperi Rhizoma], Amomum villosum Lour. [Zingiberaceae; Amomi Fuctus], Pinellia ternata (Thunb.) Breit. [Araceae; Pinelliae Rhizoma], Alisma orientalis (Sam) Juzep [Alismataceae; Alismatis Rhizoma] | None | Běijīng tóngréntáng gǔfèn yǒuxiàn gōngsī tóngréntáng zhìyào chǎng, guóyào zhǔn zì  (Z11020224) | 4 weeks | None |
| Wang 2015c | Xiè bái sàn hé tòng xiè yào fāng | Decoction | Crataegus pinnatifida Bge [Rosaceae; Crataegii Fructus], Triticum aestivum L. [Gramineae; Massa Medicata Fermentata], Hordeum vulgare L. [Gramineae; Hordei Fructus Germiniatus], Gallus gallus var. domesticus Brisson [Phasianidae; Galli Stomachichum Corium] 15 g, Lycium chinese Mill. [Solanaceae; Lycii Radicis Cortex], Morus alba L. [Moraceae; Mori Radicis Cortex], Dioscorea batatas Dacne. [Dioscoreaceae; Dioscoreae Rhizoma], Atractylodes macrocepha-la Koidz [Asteraceae; Atractylodis Rhizoma Alba], Paeonia lactiflora Pall. [Paeoniaceae; Paeoniae Radix Alba], Citrus unshiu Markovich [Rutaceae; Citri Unshius Pericarpium], Raphanus sativus var. hortensis for. acanthiformis Makino [Brassicaceae; Raphani Semen], Dolichos lablab L. [Leguminosae; Lablab Semen] 10 g, Saposhnikovia divaricata Schiskin [Apiaceae; Saposhnikovia Radix] 5 g, Pinellia ternata (Thunb.) Breit. [Araceae; Pinelliae Rhizoma], Amomum villosum Lour. [Zingiberaceae; Amomi Fuctus], Glycyrrhiza uralensis Fisch. [Leguminosae; Glycyrrhizae Radix] 3 g | None | Not applicable | 4 weeks | 1 year |
| Wang 2016 | No name | Powder | - Shenling Baizhu powder (Codonopsis pilosulae (Fr.) Nannf. [Campanulaceae; Codonopsis Pilosulae Radix], Atractylodes macrocepha-la Koidz [Asteraceae; Atractylodis Rhizoma Alba], Poria cocos (Schw.) Wolf [Polyporaceae; Poria(Hoelen)]) 6 g  - Xiāo dǎo sàn (Triticum aestivum L. [Gramineae; Massa Medicata Fermentata], Crataegus pinnatifida Bge [Rosaceae; Crataegii Fructus], Hordeum vulgare L. [Gramineae; Hordei Fructus Germiniatus]) 6 g - Wèi líng sàn (Atractylodes lancea (Thunb.) DC. [Asteraceae; Atractylodis Rhizoma], Amomum villosum Lour. [Zingiberaceae; Amomi Fuctus], Citrus unshiu Markovich [Rutaceae; Citri Unshius Pericarpium]) 6 g, Raphanus sativus var. hortensis for. acanthiformis Makino [Brassicaceae; Raphani Semen] 5 g | - Night sweating: Schizandra chinensis (Turcz.) Baill. [Magnoliaceae; Schizandrae Fructus], Rhus chinensis Mill. [Anacardiaceae; Galla Rhois] - Dry stool: Trichosanthes kirilowii Maxim. [Cucurbitaceae; Trichosanthis Semen] | Not applicable | 4 weeks | None |
| Wang 2017a | Jianpi Xiaoshi decoction | Decoction | Codonopsis pilosulae (Fr.) Nannf. [Campanulaceae; Codonopsis Pilosulae Radix] 12 g, Poria cocos (Schw.) Wolf [Polyporaceae; Poria(Hoelen)], Atractylodes macrocepha-la Koidz [Asteraceae; Atractylodis Rhizoma Alba], Dioscorea batatas Dacne. [Dioscoreaceae; Dioscoreae Rhizoma], Dolichos lablab L. [Leguminosae; Lablab Semen], Triticum aestivum L. [Gramineae; Massa Medicata Fermentata] 9 g, Citrus unshiu Markovich [Rutaceae; Citri Unshius Pericarpium], Triticum aestivum L. [Gramineae; Massa Medicata Fermentata], Crataegus pinnatifida Bge [Rosaceae; Crataegii Fructus], Hordeum vulgare L. [Gramineae; Hordei Fructus Germiniatus], Glycyrrhiza uralensis Fisch. [Leguminosae; Glycyrrhizae Radix] 6 g | None | Not applicable | NR | None |
| Wang 2017b | Xingpi Yanger granule | Granule | Emilia sonchifolia (L.) DC., Gerbera piloselloides (Linn. ) Cass., Pittosporum glabratum Lindl., Valeriana jatamansi Jones | None | Guìzhōu jiàn xìng yào yè yǒuxiàn gōngsī shēngchǎn | 2 months | None |
| Wang 2017c | Xiaoer Piweile granule | Granule | Dioscorea batatas Dacne. [Dioscoreaceae; Dioscoreae Rhizoma], Gallus gallus var. domesticus Brisson [Phasianidae; Galli Stomachichum Corium], Rheum palmatum L. [Polygonaceae; Rhei Rhizoma], Amomum krabanh Pierre ex Cagnep [Zingiberaceae; Amomi Rotundus Fructus], Hordeum vulgare L. [Gramineae; Hordei Fructus Germiniatus] | None | Guìzhōu kē dùn zhìyào yǒuxiàn zérèn gōngsī, pīzhǔn wén hào  (Z20025749) | 4 weeks | None |
| Wang 2017d | Shenling Baizhu powder | Decoction | Atractylodes lancea (Thunb.) DC. [Asteraceae; Atractylodis Rhizoma], Codonopsis pilosulae (Fr.) Nannf. [Campanulaceae; Codonopsis Pilosulae Radix], Poria cocos (Schw.) Wolf [Polyporaceae; Poria(Hoelen)], Atractylodes macrocepha-la Koidz [Asteraceae; Atractylodis Rhizoma Alba] 10 g, Agastache rugosa (Fisch. et Meyer) O. Kuntze [Labiatae; Agastachis Herba], Magnolia officinalis Rehder et Wilson [Magnoliaceae; Magnoliae Cortex] 8 g, Saposhnikovia divaricata Schiskin [Apiaceae; Saposhnikovia Radix] 5 g, Glycyrrhiza uralensis Fisch. [Leguminosae; Glycyrrhizae Radix] 3 g | None | Not applicable | 2 weeks | None |
| Wang 2018a | Children's compound Jineijin chewable tablet | Tablet | Gallus gallus var. domesticus Brisson [Phasianidae; Galli Stomachichum Corium], Triticum aestivum L. [Gramineae; Massa Medicata Fermentata] | None | Hénán tài lóng yào yè gǔfèn yǒuxiàn gōngsī | 8 weeks | None |
| Wang 2018b | Xingpi Yanger granule | Granule | Emilia sonchifolia (L.) DC., Gerbera piloselloides (Linn. ) Cass., Pittosporum glabratum Lindl., Valeriana jatamansi Jones | None | Guìzhōu jiàn xìng yào yè yǒuxiàn gōngsī shēngchǎn | 4 weeks | None |
| Wang 2018c | Children's compound Jineijin chewable tablet | Tablet | Gallus gallus var. domesticus Brisson [Phasianidae; Galli Stomachichum Corium], Triticum aestivum L. [Gramineae; Massa Medicata Fermentata] | None | Hénán tài lóng yào yè gǔfèn yǒuxiàn gōngsī | 8 weeks | None |
| Wang 2018d | Jiàn pí hé wèi tāng | Decoction | Poria cocos (Schw.) Wolf [Polyporaceae; Poria(Hoelen)], Atractylodes macrocepha-la Koidz [Asteraceae; Atractylodis Rhizoma Alba] 10 g, Gallus gallus var. domesticus Brisson [Phasianidae; Galli Stomachichum Corium], Crataegus pinnatifida Bge [Rosaceae; Crataegii Fructus], Codonopsis pilosulae (Fr.) Nannf. [Campanulaceae; Codonopsis Pilosulae Radix], Coix lachryma-jobi var. ma-yeun (Roman.) Stapf [Gramineae; Coicis Semen], Artemisia capillaris Thunb. [Asteraceae; Artemisiae Capillaris Herba] 5 g, Chrysomyia megacephala Fabricius [Calliphoridae; Chrysomyiae] 3 g, Picrorrhiza scrophulariiflora Pennell [Scrophulariaceae; Picrorrhozae Rhizoma], Glycyrrhiza uralensis Fisch. [Leguminosae; Glycyrrhizae Radix] 2 g | -Severe dampness: decrease Codonopsis pilosulae (Fr.) Nannf. [Campanulaceae; Codonopsis Pilosulae Radix] until 3 g, add Coix lachryma-jobi var. ma-yeun (Roman.) Stapf [Gramineae; Coicis Semen] until 10 g, Liriope platyphylla Wang et Tang [Liliaceae; Liriopes Radix] 3 g  - Severe qi deficiency: decrease Crataegus pinnatifida Bge [Rosaceae; Crataegii Fructus] until 3 g, add Dioscorea batatas Decne. [Dioscoreaceae; Dioscoreae Rhizoma] 5 g | Not applicable | 2 weeks | None |
| Wang 2019a | Jianpi Xiaoji decoction | Decoction | Aucklandia lappa Decne [Asteraceae; Aucklandiae Radix] 3 g, Astragalus membranaceus Bunge [Leguminosae; Astragali Radix], Atractylodes macrocepha-la Koidz [Asteraceae; Atractylodis Rhizoma Alba], Pseudostellaria augustifolia Y.N.Lee [Caryophyllaceae; Pseudostellariae Radix], Poria cocos (Schw.) Wolf [Polyporaceae; Poria(Hoelen)] 10 g, Coix lachryma-jobi var. ma-yeun (Roman.) Stapf [Gramineae; Coicis Semen], Poncirus trifoliata Rafin. [Rutaceae; Aurantii Immaturus Fructus], Crataegus pinnatifida Bge [Rosaceae; Crataegii Fructus], Magnolia officinalis Rehder et Wilson [Magnoliaceae; Magnoliae Cortex] 6 g | None | Not applicable | 2 weeks | None |
| Wang 2019b | Erbao granula | Granule | Pseudostellaria augustifolia Y.N.Lee [Caryophyllaceae; Pseudostellariae Radix], Glehnia littoralis Fr. Schm. [Apiaceae; Glehniae Radix], Poria cocos (Schw.) Wolf [Polyporaceae; Poria(Hoelen)], Dioscorea batatas Dacne. [Dioscoreaceae; Dioscoreae Rhizoma], Hordeum vulgare L. [Gramineae; Hordei Fructus Germiniatus], Citrus unshiu Markovich [Rutaceae; Citri Unshius Pericarpium], Paeonia lactiflora Pall. [Paeoniaceae; Paeoniae Radix Alba], Crataegus pinnatifida Bge [Rosaceae; Crataegii Fructus], Dolichos lablab L. [Leguminosae; Lablab Semen], Liriope platyphylla Wang et Tang [Liliaceae; Liriopes Radix], Pueraria thunbergiana Benth. [Leguminosae; Puerariae Radix] | None | Jiāngxī yào dū zhāngshù zhìyào yǒuxiàn gōngsī | 4 weeks | None |
| Wang 2020a | Sanren decoction and Xiehuang powder | Decoction | Coix lachryma-jobi var. ma-yeun (Roman.) Stapf [Gramineae; Coicis Semen] 30 g, Talc 15 g, Scutellaria baicalensis Georgi [Labiatae; Scutellariae Radix], Prunus armeniaca L. var. ansu Maxim. [Rosaceae; Armeniacae Semen], Agastache rugosa (Fisch. et Meyer) O. Kuntze [Labiatae; Agastachis Herba], Saposhnikovia divaricata Schiskin [Apiaceae; Saposhnikovia Radix], Pinellia ternata (Thunb.) Breit. [Araceae; Pinelliae Rhizoma], Lophatherum gracile Brongn. [Gramineae; Lophatheri Herba] 10 g, Gardenia jasminoides var. grandiflora (Lour.) Nakai [Rubiaceae; Gardeniae Fructus], Magnolia officinalis Rehder et Wilson [Magnoliaceae; Magnoliae Cortex] 9 g, Amomum krabanh Pierre ex Cagnep [Zingiberaceae; Amomi Rotundus Fructus], Tetrapanax papyriferus K. Koch [Araliaceae; Tetrapanacis Medulla], Glycyrrhiza uralensis Fisch. [Leguminosae; Glycyrrhizae Radix] 5 g | - Thirst, Irritability: Prunus mume Sieb. et Zucc [Rosaceae; Mume Fructus], Chaenomeles sinensis Koehne [Rosaceae; Chaenomelis Fuctus] 10 g - Eat less: Triticum aestivum L. [Gramineae; Massa Medicata Fermentata], Crataegus pinnatifida Bge [Rosaceae; Crataegii Fructus], Gallus gallus var. domesticus Brisson [Phasianidae; Galli Stomachichum Corium], Oryza sativa L. [Gramineae; Oryzae Semen Germinatus] 10 g - Shortage of qi, unwilling to talk: Dioscorea batatas Dacne. [Dioscoreaceae; Dioscoreae Rhizoma], Nelumbo nucifera Gaertner [Nymphaceae; Nelumbinis Semen], Codonopsis pilosulae (Fr.) Nannf. [Campanulaceae; Codonopsis Pilosulae Radix], Poria cocos (Schw.) Wolf [Polyporaceae; Poria(Hoelen)] 10 g -abdominal distension: Rheum palmatum L. [Polygonaceae; Rhei Rhizoma], Areca catechu L. [Arecaceae; Arecae Semen] 10 g - Fetid mouth odor and profuse sweating: Coptis deltoidea C.Y. Cheng et Hsiao [Ranunculaceae; Coptidis Rhizoma] 5 g | Not applicable | 3 weeks | None |
| Wang 2020b | Zī péi tāng | Decoction | Dioscorea batatas Dacne. [Dioscoreaceae; Dioscoreae Rhizoma] 24 g, Coix lachryma-jobi var. ma-yeun (Roman.) Stapf [Gramineae; Coicis Semen], Oryza sativa L. [Gramineae; Oryzae Fructus Germinatus], Hordeum vulgare L. [Gramineae; Hordei Fructus Germiniatus] 15 g, Scrophularia buergeriana Miq. [Scrophulariaceae; Scrophulariae Radix], Pseudostellaria augustifolia Y.N.Lee [Caryophyllaceae; Pseudostellariae Radix], Poria cocos (Schw.) Wolf [Polyporaceae; Poria(Hoelen)], Dendrobium loddigesii Rolfe. [Orchidaceae; Denbrobii Herba], Liriope platyphylla Wang et Tang [Liliaceae; Liriopes Radix] 9 g, Atractylodes macrocepha-la Koidz [Asteraceae; Atractylodis Rhizoma Alba], Gallus gallus var. domesticus Brisson [Phasianidae; Galli Stomachichum Corium], Paeonia lactiflora Pall. [Paeoniaceae; Paeoniae Radix Alba], Citrus unshiu Markovich [Rutaceae; Citri Unshius Pericarpium], Glycyrrhiza uralensis Fisch. [Leguminosae; Glycyrrhizae Radix] 6 g | None | Not applicable | 6 weeks | None |
| Wang 2020c | Shenling Baizhu powder | Decoction | Atractylodes lancea (Thunb.) DC. [Asteraceae; Atractylodis Rhizoma], Poria cocos (Schw.) Wolf [Polyporaceae; Poria(Hoelen)] 12 g, Codonopsis pilosulae (Fr.) Nannf. [Campanulaceae; Codonopsis Pilosulae Radix], Atractylodes macrocepha-la Koidz [Asteraceae; Atractylodis Rhizoma Alba] 10 g, Agastache rugosa (Fisch. et Meyer) O. Kuntze [Labiatae; Agastachis Herba], Magnolia officinalis Rehder et Wilson [Magnoliaceae; Magnoliae Cortex] 9 g, Saposhnikovia divaricata Schiskin [Apiaceae; Saposhnikovia Radix], Glycyrrhiza uralensis Fisch. [Leguminosae; Glycyrrhizae Radix] 4 g | None | Not applicable | 2 weeks | None |
| Wei 2015 | Xingpi Yanger granule | Granule | Emilia sonchifolia (L.) DC., Gerbera piloselloides (Linn. ) Cass., Pittosporum glabratum Lindl., Valeriana jatamansi Jones | None | Guìzhōu jiàn xìng yào yè yǒuxiàn gōngsī shēngchǎn | 4 weeks | 6 month |
| Wu 2001 | Kàng yànshí héjì | Decoction | Codonopsis pilosulae (Fr.) Nannf. [Campanulaceae; Codonopsis Pilosulae Radix], Atractylodes macrocepha-la Koidz [Asteraceae; Atractylodis Rhizoma Alba], Citrus unshiu Markovich [Rutaceae; Citri Unshius Pericarpium], Magnolia officinalis Rehder et Wilson [Magnoliaceae; Magnoliae Cortex], Ligusticum chuanxiong Hort [Apiaceae; Ligustici Rhizoma], Oryza sativa L. [Gramineae; Oryzae Fructus Germinatus], Hordeum vulgare L. [Gramineae; Hordei Fructus Germiniatus] | None | Not applicable | 4 weeks | None |
| Wu 2003 | Sijunzi decoction | Decoction | Codonopsis pilosulae (Fr.) Nannf. [Campanulaceae; Codonopsis Pilosulae Radix], Atractylodes macrocepha-la Koidz [Asteraceae; Atractylodis Rhizoma Alba] 10 g, Dioscorea batatas Dacne. [Dioscoreaceae; Dioscoreae Rhizoma], Crataegus pinnatifida Bge [Rosaceae; Crataegii Fructus], Hordeum vulgare L. [Gramineae; Hordei Fructus Germiniatus], Oryza sativa L. [Gramineae; Oryzae Fructus Germinatus], Citrus unshiu Markovich [Rutaceae; Citri Unshius Pericarpium], Forsythia suspensa (Thunb.) Vahl [Oleaceae; Forsythiae Fructus] 5 g, Amomum krabanh Pierre ex Cagnep [Zingiberaceae; Amomi Rotundus Fructus], Amomum villosum Lour. [Zingiberaceae; Amomi Fuctus], Glycyrrhiza uralensis Fisch. [Leguminosae; Glycyrrhizae Radix] 3 g | None | Not applicable | 2 weeks | 6 month |
| Wu 2007 | Huà jī kǒufú yè | Decoction | Poria cocos (Schw.) Wolf [Polyporaceae; Poria(Hoelen)], Sepia esculenta Hoyle [Sepiolidae; Sepiae Os], Gallus gallus var. domesticus Brisson [Phasianidae; Galli Stomachichum Corium], Areca catechu L. [Arecaceae; Arecae Semen], Omphalia lapidescens Schoret. [Polyporaceae; Omphalia Polyporus], Carpesium abrotanoides L. [Asteraceae; Carpesii Fructus], Quisqualis indica L. [Combretaceae; Quisqualis Fuctus], Scirpus flaviatilis (Torr.) A. Gray [Sparganiaceae; Scirpi Rhizoma], Curcuma zedoaria Rocs. [Zingiberaceae; Zedoariae Rhizoma], Carthamus tinctorius L. [Asteraceae; Carthami Flos] | None | Jiāngxī de wēi yào yè yǒuxiàn gōngsī | 4 weeks | None |
| Wu 2009 | Kaiwei Jianpi decoction | Decoction | Pseudostellaria augustifolia Y.N.Lee [Caryophyllaceae; Pseudostellariae Radix] 10~15 g, Poria cocos (Schw.) Wolf [Polyporaceae; Poria(Hoelen)], Oryza sativa L. [Gramineae; Oryzae Fructus Germinatus], Hordeum vulgare L. [Gramineae; Hordei Fructus Germiniatus], Crataegus pinnatifida Bge [Rosaceae; Crataegii Fructus], Bù zhā yè 5~10 g, Atractylodes macrocepha-la Koidz [Asteraceae; Atractylodis Rhizoma Alba], Glycyrrhiza uralensis Fisch. [Leguminosae; Glycyrrhizae Radix], Gallus gallus var. domesticus Brisson [Phasianidae; Galli Stomachichum Corium] 3~5 g | - Abdominal distention, physically weak, pale complexion, pale red tongue, white thin fur or white slimy fur, red fingerprint, slippery pulse: Fraxinus rhynchophylla Hance [Oleacae; Fraxini Cortex], Triticum aestivum L. [Gramineae; Massa Medicata Fermentata], Eupatorium chinese for. tripartitum H. Hara [Asteraceae; Eupatorii Herba] - Unwilling to talk lack of strength, chlorosis, sloopy or undigested stool, pale tongue, thin white fur, weak and relaxed pulse: Dioscorea batatas Dacne. [Dioscoreaceae; Dioscoreae Rhizoma], Dolichos lablab L. [Leguminosae; Lablab Semen], Nelumbo nucifera Gaertner [Nymphaceae; Nelumbinis Semen], Coix lachryma-jobi var. ma-yeun (Roman.) Stapf [Gramineae; Coicis Semen] - Dry tongue, matt complexion and chlorosis, dry stool, dry red tongue, fine pulse: Dendrobium loddigesii Rolfe. [Orchidaceae; Denbrobii Herba], Adenophora triphylla var. japonica Hara [Campanulaceae; Adenophorae Radix], Liriope platyphylla Wang et Tang [Liliaceae; Liriopes Radix], Polygonatum odoratum var. pluriflorum Ohwi [Liliaceae; Polygonati Dodrati Rhizoma] | Not applicable | 10 days | None |
| Wu 2011 | No name | Decoction | Crataegus pinnatifida Bge [Rosaceae; Crataegii Fructus], Triticum aestivum L. [Gramineae; Massa Medicata Fermentata], Hordeum vulgare L. [Gramineae; Hordei Fructus Germiniatus], Paeonia lactiflora Pall. [Paeoniaceae; Paeoniae Radix Alba], Codonopsis pilosulae (Fr.) Nannf. [Campanulaceae; Codonopsis Pilosulae Radix] 10 g, Poria cocos (Schw.) Wolf [Polyporaceae; Poria(Hoelen)] 9 g, Aucklandia lappa Decne [Asteraceae; Aucklandiae Radix], Amomum villosum Lour. [Zingiberaceae; Amomi Fuctus] 5 g, Corydalis ternata Nakai [Papaveraceae; Corydalis (Tuber) Rhizoma] 6 g, Glycyrrhiza uralensis Fisch. [Leguminosae; Glycyrrhizae Radix] 3 g | None | Not applicable | 15 days | None |
| Wu 2016 | Sijunzi decoction | Decoction | Poria cocos (Schw.) Wolf [Polyporaceae; Poria(Hoelen)] 10 g, Gallus gallus var. domesticus Brisson [Phasianidae; Galli Stomachichum Corium], Triticum aestivum L. [Gramineae; Massa Medicata Fermentata], Crataegus pinnatifida Bge [Rosaceae; Crataegii Fructus] 8 g, Pseudostellaria augustifolia Y.N.Lee [Caryophyllaceae; Pseudostellariae Radix], Atractylodes macrocepha-la Koidz [Asteraceae; Atractylodis Rhizoma Alba], Glycyrrhiza uralensis Fisch. [Leguminosae; Glycyrrhizae Radix], Atractylodes lancea (Thunb.) DC. [Asteraceae; Atractylodis Rhizoma], Magnolia officinalis Rehder et Wilson [Magnoliaceae; Magnoliae Cortex], Citrus unshiu Markovich [Rutaceae; Citri Unshius Pericarpium], Striga asiatica (L.) O. Kuntze 5 g | None | Not applicable | 10 days | None |
| Wu 2018 | Xiǎo'ér xiāoshí kēlì | Granule | Gallus gallus var. domesticus Brisson [Phasianidae; Galli Stomachichum Corium], Crataegus pinnatifida Bge [Rosaceae; Crataegii Fructus], Triticum aestivum L. [Gramineae; Massa Medicata Fermentata], Hordeum vulgare L. [Gramineae; Hordei Fructus Germiniatus], Citrus unshiu Markovich [Rutaceae; Citri Unshius Pericarpium] | None | Dàtóng dà yuán yào yè yǒuxiàn zérèn gōngsī, pīzhǔn wén hào | 4 weeks | None |
| Xia 2016 | Zengshi decoction | Decoction | Pseudostellaria augustifolia Y.N.Lee [Caryophyllaceae; Pseudostellariae Radix] 12 g, Dioscorea batatas Dacne. [Dioscoreaceae; Dioscoreae Rhizoma], Bupleurum falcatum Linne [Apiaceae; Bupleuri Radix], Peucedanum decursivum (Miq.) Maxim. [Apiaceae; Peucedani Radix], Agastache rugosa (Fisch. et Meyer) O. Kuntze [Labiatae; Agastachis Herba], Nelumbo nucifera Gaertner [Nymphaceae; Nelumbinis Foluim], Prunus mume Sieb. et Zucc [Rosaceae; Mume Fructus], Forsythia suspensa (Thunb.) Vahl [Oleaceae; Forsythiae Fructus], Gallus gallus var. domesticus Brisson [Phasianidae; Galli Stomachichum Corium] 6 g, Glycyrrhiza uralensis Fisch. [Leguminosae; Glycyrrhizae Radix] 4 g, Hordeum vulgare L. [Gramineae; Hordei Fructus Germiniatus] 15 g | - Nausea and vomiting: Phragmites communis Trin. [Gramineae; Phragmitis Rhizoma] 6 g, Phyllostachys nigra var. henonis (Bean.) Stapf [Gramineae; Bambusae Caulis In Taeniam] 4 g - Abdominal pain and distension: Aucklandia lappa Decne [Asteraceae; Aucklandiae Radix], Perilla frutescens (L.) Brit. [Labiatae; Perillae Caulis] 6 g - Dry stool: Rehmannia glutinosa var. purpurea (Makino) Makino et Nemoto [Scrophulariaceae; Rehmanniae Radix] 6 g, Cannabis sativa L. [Cannabinaceae; Cannabis Fructus] 10 g | Not applicable | 2 weeks | None |
| Xing 2012 | Yunpi powder | Decoction | Atractylodes lancea (Thunb.) DC. [Asteraceae; Atractylodis Rhizoma], Atractylodes macrocepha-la Koidz [Asteraceae; Atractylodis Rhizoma Alba], Poria cocos (Schw.) Wolf [Polyporaceae; Poria(Hoelen)], Dioscorea batatas Dacne. [Dioscoreaceae; Dioscoreae Rhizoma], Coix lachryma-jobi var. ma-yeun (Roman.) Stapf [Gramineae; Coicis Semen], Triticum aestivum L. [Gramineae; Massa Medicata Fermentata] 5~10 g, Citrus unshiu Markovich [Rutaceae; Citri Unshius Pericarpium], Citrus aurantium L. [Rutaceae; Aurantii Fructus Pericarpium], Glycyrrhiza uralensis Fisch. [Leguminosae; Glycyrrhizae Radix] 3~8 g | None | Not applicable | 15 days | 1 month |
| Xiong 2013 | Qí hú zhā kēlì | Granule | Astragalus membranaceus Bunge [Leguminosae; Astragali Radix], Dioscorea batatas Dacne. [Dioscoreaceae; Dioscoreae Rhizoma], Glycyrrhiza uralensis Fisch. [Leguminosae; Glycyrrhizae Radix], Poria cocos (Schw.) Wolf [Polyporaceae; Poria(Hoelen)], Dendrobium loddigesii Rolfe. [Orchidaceae; Denbrobii Herba], Hordeum vulgare L. [Gramineae; Hordei Fructus Germiniatus], Crataegus pinnatifida Bge [Rosaceae; Crataegii Fructus], Chaenomeles sinensis Koehne [Rosaceae; Chaenomelis Fuctus], Codonopsis pilosulae (Fr.) Nannf. [Campanulaceae; Codonopsis Pilosulae Radix], Atractylodes macrocepha-la Koidz [Asteraceae; Atractylodis Rhizoma Alba] | None | Guìzhōu hóng qí yào yè yǒuxiàn gōngsī shēngchǎn | 2 weeks | None |
| Xu 2005 | Shān mài jiàn pí kǒufú yè | Decoction | Crataegus pinnatifida Bge [Rosaceae; Crataegii Fructus], Hordeum vulgare L. [Gramineae; Hordei Fructus Germiniatus], Amomum villosum Lour. [Zingiberaceae; Amomi Fuctus], Citrus unshiu Markovich [Rutaceae; Citri Unshius Pericarpium], Alpinia officinarum Hance [Zingiberaceae; Alpiniae Officinarum Rhizoma], Zingiber officinale Rosc. [Zingiberaceae; Zingiberis Rhizoma Siccus], Gardenia jasminoides var. grandiflora (Lour.) Nakai [Rubiaceae; Gardeniae Fructus] | None | Chéngdū ēn wēi bǎojiàn zhìyào gōngsī shēngchǎn | 2 weeks | None |
| Xu 2008 | Jiàn pí zēng shí kēlì | Granule | Pseudostellaria augustifolia Y.N.Lee [Caryophyllaceae; Pseudostellariae Radix], Sophora flavescens Ait. [Leguminosae; Sophorae Radix], Polygonum orientale L. [Polygonaceae; Polygoni Orientalis Fructus], Crataegus pinnatifida Bge [Rosaceae; Crataegii Fructus], Gallus gallus var. domesticus Brisson [Phasianidae; Galli Stomachichum Corium], Prunus mume Sieb. et Zucc [Rosaceae; Mume Fructus], Manis pentadactyla L. [Manidae; Manititis Squama] | None | Not applicable | 2 weeks | None |
| Xu 2015 | Jianpi Xiaoshi decoction | Decoction | Crataegus pinnatifida Bge [Rosaceae; Crataegii Fructus], Triticum aestivum L. [Gramineae; Massa Medicata Fermentata], Hordeum vulgare L. [Gramineae; Hordei Fructus Germiniatus] 18~30 g, Pseudostellaria augustifolia Y.N.Lee [Caryophyllaceae; Pseudostellariae Radix], Citrus aurantium L. [Rutaceae; Aurantii Fructus Pericarpium], Glycyrrhiza uralensis Fisch. [Leguminosae; Glycyrrhizae Radix], Citrus unshiu Markovich [Rutaceae; Citri Unshius Pericarpium], Atractylodes macrocepha-la Koidz [Asteraceae; Atractylodis Rhizoma Alba], Poria cocos (Schw.) Wolf [Polyporaceae; Poria(Hoelen)], Selaginella tamariscina (Beauv.) Spring [Selaginellaceae; Selaginelliae Herba] 3~6 g | None | Not applicable | 8 weeks | 6 month, 1 year |
| Xue 2016 | Jianpi decoction | Decoction | Pseudostellaria augustifolia Y.N.Lee [Caryophyllaceae; Pseudostellariae Radix] 10~15 g, Poria cocos (Schw.) Wolf [Polyporaceae; Poria(Hoelen)], Oryza sativa L. [Gramineae; Oryzae Fructus Germinatus], Hordeum vulgare L. [Gramineae; Hordei Fructus Germiniatus], Crataegus pinnatifida Bge [Rosaceae; Crataegii Fructus], Bù zhā yè 5~10 g, Atractylodes macrocepha-la Koidz [Asteraceae; Atractylodis Rhizoma Alba], Glycyrrhiza uralensis Fisch. [Leguminosae; Glycyrrhizae Radix], Gallus gallus var. domesticus Brisson [Phasianidae; Galli Stomachichum Corium] 3~5 g | - Slippery pulse, white thin fur or white slimy fur, pale red tongue, pale complexion, physically weak, abdominal distention: Eupatorium chinese for. tripartitum H. Hara [Asteraceae; Eupatorii Herba], Triticum aestivum L. [Gramineae; Massa Medicata Fermentata], Citrus unshiu Markovich [Rutaceae; Citri Unshius Pericarpium] - Weak and relaxed pulse, thin white fur, pale tongue, Sloopy or Undigested stool, chlorosis, unwilling to talk, lack of strength : Coix lachryma-jobi var. ma-yeun (Roman.) Stapf [Gramineae; Coicis Semen], Nelumbo nucifera Gaertner [Nymphaceae; Nelumbinis Semen], Dolichos lablab L. [Leguminosae; Lablab Semen], Dioscorea batatas Dacne. [Dioscoreaceae; Dioscoreae Rhizoma]  - Fine pulse, dry red tongue, dry stool, matt complexion and chlorosis, dry mouth and tongue: Polygonatum odoratum var. pluriflorum Ohwi [Liliaceae; Polygonati Dodrati Rhizoma], Liriope platyphylla Wang et Tang [Liliaceae; Liriopes Radix], Adenophora triphylla var. japonica Hara [Campanulaceae; Adenophorae Radix], Dendrobium loddigesii Rolfe. [Orchidaceae; Denbrobii Herba] | Not applicable | 4 weeks | None |
| Yan 2020 | Cāng shān jiàn pí tāng | Decoction | Dioscorea batatas Dacne. [Dioscoreaceae; Dioscoreae Rhizoma] 15 g, Crataegus pinnatifida Bge [Rosaceae; Crataegii Fructus] 12 g, Atractylodes lancea (Thunb.) DC. [Asteraceae; Atractylodis Rhizoma], Dolichos lablab L. [Leguminosae; Lablab Semen], Triticum aestivum L. [Gramineae; Massa Medicata Fermentata], Gallus gallus var. domesticus Brisson [Phasianidae; Galli Stomachichum Corium], Picrorrhiza scrophulariiflora Pennell [Scrophulariaceae; Picrorrhozae Rhizoma] 10 g, Aucklandia lappa Decne [Asteraceae; Aucklandiae Radix], Citrus unshiu Markovich [Rutaceae; Citri Unshius Pericarpium]6 g, Amomum villosum Lour. [Zingiberaceae; Amomi Fuctus] 3 g | - Obvious qi deficiency: Astragalus membranaceus Bunge [Leguminosae; Astragali Radix], Codonopsis pilosulae (Fr.) Nannf. [Campanulaceae; Codonopsis Pilosulae Radix] - Stomach yin deficiency: Polygonatum odoratum var. pluriflorum Ohwi [Liliaceae; Polygonati Dodrati Rhizoma], Dendrobium loddigesii Rolfe. [Orchidaceae; Denbrobii Herba], Liriope platyphylla Wang et Tang [Liliaceae; Liriopes Radix] - Blood stasis: Prunus percisa (L.) Batsch [Rosaceae; Persicae Semen] - Dry stool: Areca catechu L. [Arecaceae; Arecae Semen] - Frightened child: Uncaria sinensis (Oliv.) Havil. [Rubiaceae; Uncariae Ramulus et Uncus], Cryptotympana pustulata Fabricius [Cicadidae; Cicadae Periostracum] | Not applicable | 10 days | None |
| Yan 2021 | Xiaoer Yanshi decoction | Decoction | Dioscorea batatas Dacne. [Dioscoreaceae; Dioscoreae Rhizoma] 8 g, Dolichos lablab L. [Leguminosae; Lablab Semen], Gallus gallus var. domesticus Brisson [Phasianidae; Galli Stomachichum Corium], Crataegus pinnatifida Bge [Rosaceae; Crataegii Fructus] 6 g, Paeonia lactiflora Pall. [Paeoniaceae; Paeoniae Radix Alba], Prunus mume Sieb. et Zucc [Rosaceae; Mume Fructus], Adenophora triphylla var. japonica Hara [Campanulaceae; Adenophorae Radix] 5 g, Glycyrrhiza uralensis Fisch. [Leguminosae; Glycyrrhizae Radix] 3 g | None | Not applicable | 12 weeks | None |
| Yang 2004 | Xingpi Yanger granule | Granule | Emilia sonchifolia (L.) DC., Gerbera piloselloides (Linn. ) Cass., Pittosporum glabratum Lindl., Valeriana jatamansi Jones | None | Guìzhōu jiàn xìng yào yè yǒuxiàn gōngsī shēngchǎn | 3 weeks | None |
| Yang 2006 | Xiǎo'ér chángwèi kāng kēlì | Granule | Kummerowia stipulacea (Max.) Makino [Leguminosae; Kummerowiae Herba], Elephantopus scaber L. [Asteraceae; Elephantopi Herba], Eriocaulon sieboldianum S. Etz. [Eriocaulaceae; Eriocauli Herba], Vespertilio superans Thomas [Bespertilionidae; Vespertilii Excrementum], Stool, Cryptotympana pustulata Fabricius [Cicadidae; Cicadae Periostracum], Oryza sativa L. [Gramineae; Oryzae Fructus Germinatus], Aucklandia lappa Decne [Asteraceae; Aucklandiae Radix], Codonopsis pilosulae (Fr.) Nannf. [Campanulaceae; Codonopsis Pilosulae Radix] | None | Jiāngnán xīnyú zhìyào yǒuxiàn gōngsī | 2 weeks | None |
| Yang 2008 | Xiāo pǐ jiàn wèisàn | Powder | Pseudostellaria augustifolia Y.N.Lee [Caryophyllaceae; Pseudostellariae Radix], Atractylodes macrocepha-la Koidz [Asteraceae; Atractylodis Rhizoma Alba], Citrus unshiu Markovich [Rutaceae; Citri Unshius Pericarpium], Syzygium aromaticum Merr et Perry [Myrtaceac; Syzygii Flos], Amomum villosum Lour. [Zingiberaceae; Amomi Fuctus], Alpinia katsumadai Hayata [Zingiberaceae; Alpiniae Katsumadai Semen], Coptis deltoidea C.Y. Cheng et Hsiao [Ranunculaceae; Coptidis Rhizoma], Gallus gallus var. domesticus Brisson [Phasianidae; Galli Stomachichum Corium], Crataegus pinnatifida Bge [Rosaceae; Crataegii Fructus], Triticum aestivum L. [Gramineae; Massa Medicata Fermentata], Hordeum vulgare L. [Gramineae; Hordei Fructus Germiniatus] | None | Not applicable | 1 month | None |
| Yang 2010 | Xiehuang powder | Decoction | Saposhnikovia divaricata Schiskin [Apiaceae; Saposhnikovia Radix] 10-15 g, Gypsum Fibrosum 12-1 5g, Gardenia jasminoides var. grandiflora (Lour.) Nakai [Rubiaceae; Gardeniae Fructus], Agastache rugosa (Fisch. et Meyer) O. Kuntze [Labiatae; Agastachis Herba] 6-9 g, Glycyrrhiza uralensis Fisch. [Leguminosae; Glycyrrhizae Radix] 3 g | - Yin damage: Dendrobium loddigesii Rolfe. [Orchidaceae; Denbrobii Herba], Rehmannia glutinosa var. purpurea (Makino) Makino et Nemoto [Scrophulariaceae; Rehmanniae Radix], Prunus mume Sieb. et Zucc [Rosaceae; Mume Fructus], Glycyrrhiza uralensis Fisch. [Leguminosae; Glycyrrhizae Radix] - Spleen qi deficiency: Codonopsis pilosulae (Fr.) Nannf. [Campanulaceae; Codonopsis Pilosulae Radix], Atractylodes macrocepha-la Koidz [Asteraceae; Atractylodis Rhizoma Alba], Citrus unshiu Markovich [Rutaceae; Citri Unshius Pericarpium] - To communicate six bowels: Magnolia officinalis Rehder et Wilson [Magnoliaceae; Magnoliae Cortex], Areca catechu L. [Arecaceae; Arecae Semen], Poncirus trifoliata Rafin. [Rutaceae; Aurantii Immaturus Fructus] | Not applicable | 1 month | None |
| Yang 2013a | Tiaozhong decoction | Decoction | Poria cocos (Schw.) Wolf [Polyporaceae; Poria(Hoelen)], Atractylodes macrocepha-la Koidz [Asteraceae; Atractylodis Rhizoma Alba] 15 g, Codonopsis pilosulae (Fr.) Nannf. [Campanulaceae; Codonopsis Pilosulae Radix], Citrus unshiu Markovich [Rutaceae; Citri Unshius Pericarpium], Crataegus pinnatifida Bge [Rosaceae; Crataegii Fructus], Triticum aestivum L. [Gramineae; Massa Medicata Fermentata] 10 g, Citrus aurantium L. [Rutaceae; Aurantii Fructus Pericarpium] 8 g, Glycyrrhiza uralensis Fisch. [Leguminosae; Glycyrrhizae Radix] 3 g | None | Not applicable | 4 weeks | None |
| Yang 2013b | Shenling Baizhu granule | Granule | Atractylodes macrocepha-la Koidz [Asteraceae; Atractylodis Rhizoma Alba], Dioscorea batatas Dacne. [Dioscoreaceae; Dioscoreae Rhizoma], Dolichos lablab L. [Leguminosae; Lablab Semen], Amomum villosum Lour. [Zingiberaceae; Amomi Fuctus], Coix lachryma-jobi var. ma-yeun (Roman.) Stapf [Gramineae; Coicis Semen], Nelumbo nucifera Gaertner [Nymphaceae; Nelumbinis Semen], Panax ginseng C. A. Mey. [Araliaceae; Ginseng Radix], Poria cocos (Schw.) Wolf [Polyporaceae; Poria(Hoelen)] | None | Yúnnán shěng téngchōng zhìyào chǎng | 7 days | None |
| Yang 2013c | Jianpi Xiaoji pill | Decoction | Pseudostellaria augustifolia Y.N.Lee [Caryophyllaceae; Pseudostellariae Radix], Atractylodes macrocepha-la Koidz [Asteraceae; Atractylodis Rhizoma Alba], Dioscorea batatas Dacne. [Dioscoreaceae; Dioscoreae Rhizoma], Dolichos lablab L. [Leguminosae; Lablab Semen], Areca catechu L. [Arecaceae; Arecae Semen], Citrus unshiu Markovich [Rutaceae; Citri Unshius Pericarpium], Gallus gallus var. domesticus Brisson [Phasianidae; Galli Stomachichum Corium], Crataegus pinnatifida Bge [Rosaceae; Crataegii Fructus], Triticum aestivum L. [Gramineae; Massa Medicata Fermentata], Hordeum vulgare L. [Gramineae; Hordei Fructus Germiniatus], Poria cocos (Schw.) Wolf [Polyporaceae; Poria(Hoelen)] 5-10 g, Amomum villosum Lour. [Zingiberaceae; Amomi Fuctus], Picrorrhiza scrophulariiflora Pennell [Scrophulariaceae; Picrorrhozae Rhizoma], Glycyrrhiza uralensis Fisch. [Leguminosae; Glycyrrhizae Radix] 3-6 g | - Nausea and vomiting: Pinellia ternata (Thunb.) Breit. [Araceae; Pinelliae Rhizoma], Zingiber officinale Rosc. [Zingiberaceae; Zingiberis Rhizoma Recens] - Irritation, anxiety, restless sleep at night: Gardenia jasminoides var. grandiflora (Lour.) Nakai [Rubiaceae; Gardeniae Fructus], Lilium lancifolium Thunb. [Liliaceae; Lili Bulbus] - Enlarged tongue, white slimy fur: Amomum krabanh Pierre ex Cagnep [Zingiberaceae; Amomi Rotundus Fructus] - Physically weak, profuse sweating, easy to catch a cold: Astragalus membranaceus Bunge [Leguminosae; Astragali Radix], Saposhnikovia divaricata Schiskin [Apiaceae; Saposhnikovia Radix] - Abdominal distension: Aucklandia lappa Decne [Asteraceae; Aucklandiae Radix], Magnolia officinalis Rehder et Wilson [Magnoliaceae; Magnoliae Cortex] - Dry stool: Poncirus trifoliata Rafin. [Rutaceae; Aurantii Immaturus Fructus], Raphanus sativus var. hortensis for. acanthiformis Makino [Brassicaceae; Raphani Semen] - Loose stool: remove Areca catechu L. [Arecaceae; Arecae Semen], change Atractylodes macrocepha-la Koidz [Asteraceae; Atractylodis Rhizoma Alba] to Atractylodes lancea (Thunb.) DC. [Asteraceae; Atractylodis Rhizoma], add Nelumbo nucifera Gaertner [Nymphaceae; Nelumbinis Semen], Coix lachryma-jobi var. ma-yeun (Roman.) Stapf [Gramineae; Coicis Semen] | Not applicable | 14 days | None |
| Yang 2014 | Xiaoer Piweile granule | Granule | Dioscorea batatas Dacne. [Dioscoreaceae; Dioscoreae Rhizoma], Rheum palmatum L. [Polygonaceae; Rhei Rhizoma], Gallus gallus var. domesticus Brisson [Phasianidae; Galli Stomachichum Corium], Myristica fragrans Houtt. [Myristicaceae; Myristicae Semen], Hordeum vulgare L. [Gramineae; Hordei Fructus Germiniatus] | None | Kūnmíng bāng yǔ zhìyào yǒuxiàn gōngsī shēngchǎn | 4 weeks | None |
| Yang 2020 | Jianpi Fei'er tangjiang | Syrup | Dioscorea batatas Dacne. [Dioscoreaceae; Dioscoreae Rhizoma], Atractylodes macrocepha-la Koidz [Asteraceae; Atractylodis Rhizoma Alba], Aspongopus chinensis Dallas, Gallus gallus var. domesticus Brisson [Phasianidae; Galli Stomachichum Corium], Crataegus pinnatifida Bge [Rosaceae; Crataegii Fructus], Hordeum vulgare L. [Gramineae; Hordei Fructus Germiniatus], Oryza sativa L. [Gramineae; Oryzae Fructus Germinatus], Adenophora triphylla var. japonica Hara [Campanulaceae; Adenophorae Radix] | None | Sū yào zhì zì  (Z04001512) | 1 month | None |
| Yao 2008 | Xiāo gān lǐ pí tāng | Decoction | Hordeum vulgare L. [Gramineae; Hordei Fructus Germiniatus], Triticum aestivum L. [Gramineae; Massa Medicata Fermentata] 15 g, Citrus unshiu Markovich [Rutaceae; Citrii Unshiu Immaturi Pericarpium], Citrus unshiu Markovich [Rutaceae; Citri Unshius Pericarpium]10 g, Aloe vere L. [Liliaceae; Aloe], Areca catechu L. [Arecaceae; Arecae Semen] 8 g, Ulmus macrocarpa Hance [Ulmaceae; Ulmi Pasta Semen], Scirpus flaviatilis (Torr.) A. Gray [Sparganiaceae; Scirpi Rhizoma], Quisqualis indica L. [Combretaceae; Quisqualis Fuctus], Glycyrrhiza uralensis Fisch. [Leguminosae; Glycyrrhizae Radix] 5 g, Coptis deltoidea C.Y. Cheng et Hsiao [Ranunculaceae; Coptidis Rhizoma], Picrorrhiza scrophulariiflora Pennell [Scrophulariaceae; Picrorrhozae Rhizoma] 3 g | - Physically weak: remove Ulmus macrocarpa Hance [Ulmaceae; Ulmi Pasta Semen], Scirpus flaviatilis (Torr.) A. Gray [Sparganiaceae; Scirpi Rhizoma], Quisqualis indica L. [Combretaceae; Quisqualis Fuctus] - Thick slimy fur : Amomum tsaoko Crevost et Lemaire [Zingiberaceae; Amomi Tsao-Ko Fructus] 3-5 g, Agastache rugosa (Fisch. et Meyer) O. Kuntze [Labiatae; Agastachis Herba] 10 g - Obvious abdominal distention: Raphanus sativus var. hortensis for. acanthiformis Makino [Brassicaceae; Raphani Semen] 10 g - Loose stool: Atractylodes lancea (Thunb.) DC. [Asteraceae; Atractylodis Rhizoma] 10 g | Not applicable | 10-30 days | None |
| Yi 2018 | Fuzheng Jianpi decoction | Decoction | Dioscorea batatas Dacne. [Dioscoreaceae; Dioscoreae Rhizoma] 6 g, Atractylodes macrocepha-la Koidz [Asteraceae; Atractylodis Rhizoma Alba], Poria cocos (Schw.) Wolf [Polyporaceae; Poria(Hoelen)], Aucklandia lappa Decne [Asteraceae; Aucklandiae Radix] 5 g, Panax ginseng C. A. Mey. [Araliaceae; Ginseng Radix], Crataegus pinnatifida Bge [Rosaceae; Crataegii Fructus], Bupleurum falcatum Linne [Apiaceae; Bupleuri Radix], Hordeum vulgare L. [Gramineae; Hordei Fructus Germiniatus], Triticum aestivum L. [Gramineae; Massa Medicata Fermentata], Myristica fragrans Houtt. [Myristicaceae; Myristicae Semen], Paeonia lactiflora Pall. [Paeoniaceae; Paeoniae Radix Alba] 3 g, Citrus unshiu Markovich [Rutaceae; Citri Unshius Pericarpium], Glycyrrhiza uralensis Fisch. [Leguminosae; Glycyrrhizae Radix] 2 g | None | Not applicable | 3 weeks | None |
| You 2020 | Jianpi decoction | Decoction | Astragalus membranaceus Bunge [Leguminosae; Astragali Radix], Gallus gallus var. domesticus Brisson [Phasianidae; Galli Stomachichum Corium], Areca catechu L. [Arecaceae; Arecae Semen], Atractylodes lancea (Thunb.) DC. [Asteraceae; Atractylodis Rhizoma], Atractylodes macrocepha-la Koidz [Asteraceae; Atractylodis Rhizoma Alba], Prunus mume Sieb. et Zucc [Rosaceae; Mume Fructus], Poria cocos (Schw.) Wolf [Polyporaceae; Poria(Hoelen)] 10 g, Glycyrrhiza uralensis Fisch. [Leguminosae; Glycyrrhizae Radix] 6 g, Citrus unshiu Markovich [Rutaceae; Citri Unshius Pericarpium]5 g | None | Not applicable | 4 weeks | None |
| Yu 2009a | Jianpi Xiaoshi decoction | Decoction | Crataegus pinnatifida Bge [Rosaceae; Crataegii Fructus], Triticum aestivum L. [Gramineae; Massa Medicata Fermentata], Hordeum vulgare L. [Gramineae; Hordei Fructus Germiniatus] 18-30 g, Pseudostellaria augustifolia Y.N.Lee [Caryophyllaceae; Pseudostellariae Radix], Atractylodes macrocepha-la Koidz [Asteraceae; Atractylodis Rhizoma Alba], Poria cocos (Schw.) Wolf [Polyporaceae; Poria(Hoelen)], Glycyrrhiza uralensis Fisch. [Leguminosae; Glycyrrhizae Radix], Citrus unshiu Markovich [Rutaceae; Citri Unshius Pericarpium], Selaginella tamariscina (Beauv.) Spring [Selaginellaceae; Selaginelliae Herba], Citrus aurantium L. [Rutaceae; Aurantii Fructus Pericarpium] 3-6 g | None | Not applicable | 4-6 weeks | None |
| Yu 2009b | Xiehuang powder | Powder | Agastache rugosa (Fisch. et Meyer) O. Kuntze [Labiatae; Agastachis Herba], Gardenia jasminoides var. grandiflora (Lour.) Nakai [Rubiaceae; Gardeniae Fructus] 6-9 g, Gypsum Fibrosum 12-15 g, Saposhnikovia divaricata Schiskin [Apiaceae; Saposhnikovia Radix] 10-15 g, Glycyrrhiza uralensis Fisch. [Leguminosae; Glycyrrhizae Radix] 3 g | - Yin damage: Dendrobium loddigesii Rolfe. [Orchidaceae; Denbrobii Herba], Rehmannia glutinosa var. purpurea (Makino) Makino et Nemoto [Scrophulariaceae; Rehmanniae Radix], Prunus mume Sieb. et Zucc [Rosaceae; Mume Fructus] - Spleen qi deficiency: Codonopsis pilosulae (Fr.) Nannf. [Campanulaceae; Codonopsis Pilosulae Radix], Atractylodes macrocepha-la Koidz [Asteraceae; Atractylodis Rhizoma Alba], Citrus unshiu Markovich [Rutaceae; Citri Unshius Pericarpium] - To communicate six bowels: Magnolia officinalis Rehder et Wilson [Magnoliaceae; Magnoliae Cortex], Areca catechu L. [Arecaceae; Arecae Semen], Poncirus trifoliata Rafin. [Rutaceae; Aurantii Immaturus Fructus] | Not applicable | 1 month | None |
| Yu 2014 | Yànshí decoction | Decoction | Triticum aestivum L. [Gramineae; Massa Medicata Fermentata], Crataegus pinnatifida Bge [Rosaceae; Crataegii Fructus], Hordeum vulgare L. [Gramineae; Hordei Fructus Germiniatus] 15 g, Pseudostellaria augustifolia Y.N.Lee [Caryophyllaceae; Pseudostellariae Radix], Poria cocos (Schw.) Wolf [Polyporaceae; Poria(Hoelen)], Atractylodes macrocepha-la Koidz [Asteraceae; Atractylodis Rhizoma Alba], Citrus unshiu Markovich [Rutaceae; Citri Unshius Pericarpium], Gallus gallus var. domesticus Brisson [Phasianidae; Galli Stomachichum Corium], Magnolia officinalis Rehder et Wilson [Magnoliaceae; Magnoliae Cortex], Poncirus trifoliata Rafin. [Rutaceae; Aurantii Immaturus Fructus], Raphanus sativus var. hortensis for. acanthiformis Makino [Brassicaceae; Raphani Semen] 10 g, Glycyrrhiza uralensis Fisch. [Leguminosae; Glycyrrhizae Radix], Amomum villosum Lour. [Zingiberaceae; Amomi Fuctus] 6 g | - Obvious abdominal pain Aucklandia lappa Decne [Asteraceae; Aucklandiae Radix], Paeonia lactiflora Pall. [Paeoniaceae; Paeoniae Radix Alba], Corydalis ternata Nakai [Papaveraceae; Corydalis (Tuber) Rhizoma] 10 g - Lack of strength , profuse sweating: Astragalus membranaceus Bunge [Leguminosae; Astragali Radix] 20 g, Ephedra sinica Stapf. [Ephedraceae; Ephedrae Radix] 10 g - White slimy fur: Pinellia ternata (Thunb.) Breit. [Araceae; Pinelliae Rhizoma], Eupatorium chinese for. tripartitum H. Hara [Asteraceae; Eupatorii Herba] 9 g - Nausea and vomiting: Pinellia ternata (Thunb.) Breit. [Araceae; Pinelliae Rhizoma], Phyllostachys nigra var. henonis (Bean.) Stapf [Gramineae; Bambusae Caulis In Taeniam] 5 g | Not applicable | 20 days | None |
| Yu 2016 | Xingpi Yanger granule | Granule | Emilia sonchifolia (L.) DC., Gerbera piloselloides (Linn. ) Cass., Pittosporum glabratum Lindl., Valeriana jatamansi Jones | None | NR | 4 weeks | None |
| Yu 2017 | Xiaoer Yanshi granule | Granule | Panax ginseng C. A. Mey. [Araliaceae; Ginseng Radix], Dioscorea batatas Dacne. [Dioscoreaceae; Dioscoreae Rhizoma], Atractylodes macrocepha-la Koidz [Asteraceae; Atractylodis Rhizoma Alba], Crataegus pinnatifida Bge [Rosaceae; Crataegii Fructus], Zingiber officinale Rosc. [Zingiberaceae; Zingiberis Rhizoma Siccus], Amomum villosum Lour. [Zingiberaceae; Amomi Fuctus], Picrorrhiza scrophulariiflora Pennell [Scrophulariaceae; Picrorrhozae Rhizoma], Areca catechu L. [Arecaceae; Arecae Semen] | None | Nèiménggǔ huì fēng yào yè yǒuxiàn gōngsī | NR | None |
| Yuan 2008 | Yigong powder | Decoction | Codonopsis pilosulae (Fr.) Nannf. [Campanulaceae; Codonopsis Pilosulae Radix], Atractylodes macrocepha-la Koidz [Asteraceae; Atractylodis Rhizoma Alba], Poria cocos (Schw.) Wolf [Polyporaceae; Poria(Hoelen)], Citrus unshiu Markovich [Rutaceae; Citri Unshius Pericarpium], Citrus aurantium L. [Rutaceae; Aurantii Fructus Pericarpium] 6 g, Selaginella tamariscina (Beauv.) Spring [Selaginellaceae; Selaginelliae Herba], Eupatorium chinese for. tripartitum H. Hara [Asteraceae; Eupatorii Herba] 5 g, Forsythia suspensa (Thunb.) Vahl [Oleaceae; Forsythiae Fructus], Hordeum vulgare L. [Gramineae; Hordei Fructus Germiniatus], Oryza sativa L. [Gramineae; Oryzae Fructus Germinatus] 8 g, Glycyrrhiza uralensis Fisch. [Leguminosae; Glycyrrhizae Radix] 4 g | None | Not applicable | 1 week | None |
| Yuan 2009 | Zhuangerling decoction | Decoction | Atractylodes lancea (Thunb.) DC. [Asteraceae; Atractylodis Rhizoma], Codonopsis pilosulae (Fr.) Nannf. [Campanulaceae; Codonopsis Pilosulae Radix] 96 g, Crataegus pinnatifida Bge [Rosaceae; Crataegii Fructus], Astragalus membranaceus Bunge [Leguminosae; Astragali Radix], Hordeum vulgare L. [Gramineae; Hordei Fructus Germiniatus], Cassia tora L. [Leguminosae; Cassiae Semen], Picrorrhiza scrophulariiflora Pennell [Scrophulariaceae; Picrorrhozae Rhizoma] 160 g, Citrus unshiu Markovich [Rutaceae; Citri Unshius Pericarpium]48 g, sucrose 100 g | None | Jiāngsū shěng zhōng yīyuàn zhìjì | 8 weeks | None |
| Yuan 2010 | Er shí xiāo tāng | Decoction | Atractylodes macrocepha-la Koidz [Asteraceae; Atractylodis Rhizoma Alba], Dioscorea batatas Dacne. [Dioscoreaceae; Dioscoreae Rhizoma], Magnolia officinalis Rehder et Wilson [Magnoliaceae; Magnoliae Cortex], Poncirus trifoliata Rafin. [Rutaceae; Aurantii Immaturus Fructus], Crataegus pinnatifida Bge [Rosaceae; Crataegii Fructus], Triticum aestivum L. [Gramineae; Massa Medicata Fermentata] 10 g, Pseudostellaria augustifolia Y.N.Lee [Caryophyllaceae; Pseudostellariae Radix], Glycyrrhiza uralensis Fisch. [Leguminosae; Glycyrrhizae Radix] 5 g | - Obvious abdominal pain: Aucklandia lappa Decne [Asteraceae; Aucklandiae Radix], Paeonia lactiflora Pall. [Paeoniaceae; Paeoniae Radix Alba], Corydalis ternata Nakai [Papaveraceae; Corydalis (Tuber) Rhizoma] 10 g - Lack of strength , profuse sweating: Astragalus membranaceus Bunge [Leguminosae; Astragali Radix] 20 g, Ephedra sinica Stapf. [Ephedraceae; Ephedrae Radix] 10 g - White slimy fur: Pinellia ternata (Thunb.) Breit. [Araceae; Pinelliae Rhizoma], Eupatorium chinese for. tripartitum H. Hara [Asteraceae; Eupatorii Herba] 9 g - Nausea and vomiting: Pinellia ternata (Thunb.) Breit. [Araceae; Pinelliae Rhizoma], Phyllostachys nigra var. henonis (Bean.) Stapf [Gramineae; Bambusae Caulis In Taeniam] 5 g - Dry stool: Rheum palmatum L. [Polygonaceae; Rhei Rhizoma] 5 g, Raphanus sativus var. hortensis for. acanthiformis Makino [Brassicaceae; Raphani Semen] 10 g | Not applicable | 20 days | None |
| Yuan 2011 | Kaiwei yin | Decoction | Perilla frutescens var. crispa (Thunb.) Decne. [Labiatae; Perillae Caulis], Cyperus rotundus L. [Cyperaceae; Cyperi Rhizoma], Citrus unshiu Markovich [Rutaceae; Citri Unshius Pericarpium], Phyllostachys nigra var. henonis (Bean.) Stapf [Gramineae; Bambusae Caulis In Taeniam], Citrus aurantium L. [Rutaceae; Aurantii Fructus Pericarpium], Poria cocos (Schw.) Wolf [Polyporaceae; Poria(Hoelen)], Areca catechu L. [Arecaceae; Arecae Pericarpium], Crataegus pinnatifida Bge [Rosaceae; Crataegii Fructus], Triticum aestivum L. [Gramineae; Massa Medicata Fermentata], Hordeum vulgare L. [Gramineae; Hordei Fructus Germiniatus], Gallus gallus var. domesticus Brisson [Phasianidae; Galli Stomachichum Corium] | None | Not applicable | 4 weeks | None |
| Yuan 2019 | Daozhi decoction | Decoction | Crataegus pinnatifida Bge [Rosaceae; Crataegii Fructus], Triticum aestivum L. [Gramineae; Massa Medicata Fermentata], Raphanus sativus var. hortensis for. acanthiformis Makino [Brassicaceae; Raphani Semen], Areca catechu L. [Arecaceae; Arecae Semen], Forsythia suspensa (Thunb.) Vahl [Oleaceae; Forsythiae Fructus], Cyperus rotundus L. [Cyperaceae; Cyperi Rhizoma] 10 g, Citrus unshiu Markovich [Rutaceae; Citri Unshius Pericarpium], Pinellia ternata (Thunb.) Breit. [Araceae; Pinelliae Rhizoma], Glycyrrhiza uralensis Fisch. [Leguminosae; Glycyrrhizae Radix] 5 g | - Obvious nausea and vomiting: Phyllostachys nigra var. henonis (Bean.) Stapf [Gramineae; Bambusae Caulis In Taeniam] 6 g, Agastache rugosa (Fisch. et Meyer) O. Kuntze [Labiatae; Agastachis Herba] 10 g - Constipation: Rheum palmatum L. [Polygonaceae; Rhei Rhizoma] 1 g - Mouth and tongue sore: Phyllostachys nigra var. henonis (Bean.) Stapf [Gramineae; Phyllostachydis Folium] 5 g, Gypsum Fibrosum 20 g - External contraction symptoms(nasal congestion, cough): Perilla frutescens var. acuta Kudo [Labiatae; Perilla Folium] 6 g, Prunus armeniaca L. var. ansu Maxim. [Rosaceae; Armeniacae Semen] 10 g - Pale complexion, lean body, lack of strength , sloppy stool: Atractylodes macrocepha-la Koidz [Asteraceae; Atractylodis Rhizoma Alba], Poria cocos (Schw.) Wolf [Polyporaceae; Poria(Hoelen)] 10 g, Atractylodes lancea (Thunb.) DC. [Asteraceae; Atractylodis Rhizoma] 6 g | Not applicable | 4 weeks | None |
| Yuan 2021 | Yangwei Zengye decoction | Decoction | Glehnia littoralis Fr. Schm. [Apiaceae; Glehniae Radix], Polygonatum odoratum var. pluriflorum Ohwi [Liliaceae; Polygonati Dodrati Rhizoma], Dendrobium loddigesii Rolfe. [Orchidaceae; Denbrobii Herba], Paeonia lactiflora Pall. [Paeoniaceae; Paeoniae Radix Alba], Prunus mume Sieb. et Zucc [Rosaceae; Mume Fructus] 5 g, Crataegus pinnatifida Bge [Rosaceae; Crataegii Fructus], Triticum aestivum L. [Gramineae; Massa Medicata Fermentata], Hordeum vulgare L. [Gramineae; Hordei Fructus Germiniatus], Dolichos lablab L. [Leguminosae; Lablab Semen], Dioscorea batatas Dacne. [Dioscoreaceae; Dioscoreae Rhizoma], Oryza sativa L. [Gramineae; Oryzae Semen Germinatus] 10 g, Glycyrrhiza uralensis Fisch. [Leguminosae; Glycyrrhizae Radix] 6 g | None | Not applicable | 4 weeks | 3 month |
| Zeng 2010 | Pinggan Xiaoshi decoction | Decoction | Bupleurum falcatum Linne [Apiaceae; Bupleuri Radix] 6-10 g, Paeonia lactiflora Pall. [Paeoniaceae; Paeoniae Radix Alba], Poncirus trifoliata Rafin. [Rutaceae; Aurantii Immaturus Fructus], Atractylodes macrocepha-la Koidz [Asteraceae; Atractylodis Rhizoma Alba], Citrus unshiu Markovich [Rutaceae; Citri Unshius Pericarpium], Poria cocos (Schw.) Wolf [Polyporaceae; Poria(Hoelen)], Triticum aestivum L. [Gramineae; Massa Medicata Fermentata], Hordeum vulgare L. [Gramineae; Hordei Fructus Germiniatus], Crataegus pinnatifida Bge [Rosaceae; Crataegii Fructus] 3-10 g, Glycyrrhiza uralensis Fisch. [Leguminosae; Glycyrrhizae Radix] 3-6 g | None | Not applicable | 1 month | None |
| Zeng 2012 | Yùn pí huà shī fāng | Decoction | Triticum aestivum L. [Gramineae; Massa Medicata Fermentata], Poria cocos (Schw.) Wolf [Polyporaceae; Poria(Hoelen)], Crataegus pinnatifida Bge [Rosaceae; Crataegii Fructus] 9 g, Atractylodes macrocepha-la Koidz [Asteraceae; Atractylodis Rhizoma Alba], Poncirus trifoliata Rafin. [Rutaceae; Aurantii Immaturus Fructus], Magnolia officinalis Rehder et Wilson [Magnoliaceae; Magnoliae Cortex], Hordeum vulgare L. [Gramineae; Hordei Fructus Germiniatus] 6 g, Areca catechu L. [Arecaceae; Arecae Pericarpium], Curcuma aromatica Salisb. [Zingiberaceae; Curcumae Radix] 5 g, Scutellaria baicalensis Georgi [Labiatae; Scutellariae Radix] 2 g | None | Not applicable | 7 days | None |
| Zhan 2019 | Yigong powder | Granule | Pseudostellaria augustifolia Y.N.Lee [Caryophyllaceae; Pseudostellariae Radix] 8 g, Atractylodes macrocepha-la Koidz [Asteraceae; Atractylodis Rhizoma Alba], Poria cocos (Schw.) Wolf [Polyporaceae; Poria(Hoelen)], Citrus unshiu Markovich [Rutaceae; Citri Unshius Pericarpium], Atractylodes lancea (Thunb.) DC. [Asteraceae; Atractylodis Rhizoma], Gallus gallus var. domesticus Brisson [Phasianidae; Galli Stomachichum Corium] 5 g, Zizyphus jujuba ver. inermis Rehder [Rhamnaceae; Zizyphi Fructus], Zingiber officinale Rosc. [Zingiberaceae; Zingiberis Rhizoma Recens] 3 g, Glycyrrhiza uralensis Fisch. [Leguminosae; Glycyrrhizae Radix] 2 g | - Slimy fur, loose stool: remove Atractylodes macrocepha-la Koidz [Asteraceae; Atractylodis Rhizoma Alba], add Coix lachryma-jobi var. ma-yeun (Roman.) Stapf [Gramineae; Coicis Semen] - Indigestion: Crataegus pinnatifida Bge [Rosaceae; Crataegii Fructus], Oryza sativa L. [Gramineae; Oryzae Fructus Germinatus], Hordeum vulgare L. [Gramineae; Hordei Fructus Germiniatus] | Běijīng kāngrén táng yào yè yǒuxiàn gōngsī | 4 weeks | None |
| Zhang 2004 | Yì pí tāng | Decoction | Pseudostellaria augustifolia Y.N.Lee [Caryophyllaceae; Pseudostellariae Radix] 30 g, Dioscorea batatas Dacne. [Dioscoreaceae; Dioscoreae Rhizoma] 24 g, Dolichos lablab L. [Leguminosae; Lablab Semen], Oryza sativa L. [Gramineae; Oryzae Fructus Germinatus], Hordeum vulgare L. [Gramineae; Hordei Fructus Germiniatus] 15 g, Nelumbo nucifera Gaertner [Nymphaceae; Nelumbinis Semen], Crataegus pinnatifida Bge [Rosaceae; Crataegii Fructus], Atractylodes macrocepha-la Koidz [Asteraceae; Atractylodis Rhizoma Alba], Poria cocos (Schw.) Wolf [Polyporaceae; Poria(Hoelen)] 10 g, Platycodon grandiflorum (Jacq.) A. DC. [Campanulaceae; Platycodi Radix] 6 g, Picrorrhiza scrophulariiflora Pennell [Scrophulariaceae; Picrorrhozae Rhizoma] 5 g, Glycyrrhiza uralensis Fisch. [Leguminosae; Glycyrrhizae Radix] 3 g | None | Not applicable | 2 weeks | None |
| Zhang 2005 | No name | Powder | Poria cocos (Schw.) Wolf [Polyporaceae; Poria(Hoelen)], Crataegus pinnatifida Bge [Rosaceae; Crataegii Fructus] 25 g, Codonopsis pilosulae (Fr.) Nannf. [Campanulaceae; Codonopsis Pilosulae Radix], Atractylodes macrocepha-la Koidz [Asteraceae; Atractylodis Rhizoma Alba], Cyperus rotundus L. [Cyperaceae; Cyperi Rhizoma], Raphanus sativus var. hortensis for. acanthiformis Makino [Brassicaceae; Raphani Semen] 15 g, Triticum aestivum L. [Gramineae; Massa Medicata Fermentata], Citrus unshiu Markovich [Rutaceae; Citri Unshius Pericarpium], Gallus gallus var. domesticus Brisson [Phasianidae; Galli Stomachichum Corium] 15 g, Scirpus flaviatilis (Torr.) A. Gray [Sparganiaceae; Scirpi Rhizoma] 5 g | None | Not applicable | 15 days | None |
| Zhang 2006 | Tiaopi decoction | Decoction | Atractylodes lancea (Thunb.) DC. [Asteraceae; Atractylodis Rhizoma], Citrus unshiu Markovich [Rutaceae; Citri Unshius Pericarpium], Crataegus pinnatifida Bge [Rosaceae; Crataegii Fructus], Gallus gallus var. domesticus Brisson [Phasianidae; Galli Stomachichum Corium] | None | Not applicable | 4 weeks | None |
| Zhang 2007 | Yùn pí huà shí tāng | Decoction | Amomum villosum Lour. [Zingiberaceae; Amomi Fuctus], Aucklandia lappa Decne [Asteraceae; Aucklandiae Radix], Astagalus membranaceus Bunge [Leguminosae; Astragali Radix] 6 g, Astragalus membranaceus Bunge [Leguminosae; Astragali Radix], Pseudostellaria augustifolia Y.N.Lee [Caryophyllaceae; Pseudostellariae Radix], Poria cocos (Schw.) Wolf [Polyporaceae; Poria(Hoelen)], Areca catechu L. [Arecaceae; Arecae Semen], Pinellia ternata (Thunb.) Breit. [Araceae; Pinelliae Rhizoma Fermentata], Raphanus sativus var. hortensis for. acanthiformis Makino [Brassicaceae; Raphani Semen] 5 g, Atractylodes macrocepha-la Koidz [Asteraceae; Atractylodis Rhizoma Alba], Citrus unshiu Markovich [Rutaceae; Citri Unshius Pericarpium]4 g, Glycyrrhiza uralensis Fisch. [Leguminosae; Glycyrrhizae Radix] 3 g | - Low fever, palm and plantar fever: Picrorrhiza scrophulariiflora Pennell [Scrophulariaceae; Picrorrhozae Rhizoma], Lycium chinese Mill. [Solanaceae; Lycii Radicis Cortex] - Dry mouth, dry throat: Liriope platyphylla Wang et Tang [Liliaceae; Liriopes Radix], Adenophora triphylla var. japonica Hara [Campanulaceae; Adenophorae Radix] - Weakness: Dioscorea batatas Dacne. [Dioscoreaceae; Dioscoreae Rhizoma], Astragalus membranaceus Bunge [Leguminosae; Astragali Radix] - Dampness stagnation: Coix lachryma-jobi var. ma-yeun (Roman.) Stapf [Gramineae; Coicis Semen], Dolichos lablab L. [Leguminosae; Lablab Semen] - Abdominal distension: Areca catechu L. [Arecaceae; Arecae Pericarpium], Magnolia officinalis Rehder et Wilson [Magnoliaceae; Magnoliae Cortex] - Loose stool: Myristica fragrans Houtt. [Myristicaceae; Myristicae Semen], Terminalia chebula (Gaertner) Retz. [Combretaceae; Terminaliae Fructus] - Constipation: Trichosanthes kirilowii Maxim. [Cucurbitaceae; Trichosanthis Semen], Cannabis sativa L. [Cannabinaceae; Cannabis Fructus] | Not applicable | 30 days | None |
| Zhang 2009 | Qīngrè huà shī yǎng yīn kēlì | Granule | Artemisiae apiacea Hance {Asteraceae; Artemisiae Apiaceae Herba], Atractylodes lancea (Thunb.) DC. [Asteraceae; Atractylodis Rhizoma], Fritillaria thunbergii Miq. [Liliaceae; Fritillariae Thunbergii Bulbus], Paeonia lactiflora Pall. [Paeoniaceae; Paeoniae Radix Rubra], Raphanus sativus var. hortensis for. acanthiformis Makino [Brassicaceae; Raphani Semen] | None | Nányáng yīxué gāoděng zhuānkē xuéxiào zhōngyào shíyàn shì zhìbèi | 15 days | None |
| Zhang 2011 | Xingpi Yanger granule | Granule | Gerbera piloselloides (Linn. ) Cass., Pittosporum glabratum Lindl., Emilia sonchifolia (L.) DC., Valeriana jatamansi Jones | None | Guìzhōu jiàn xìng yào yè yǒuxiàn gōngsī shēngchǎn | 4 weeks | None |
| Zhang 2013a | Simo decoction | Decoction | Aucklandia lappa Decne [Asteraceae; Aucklandiae Radix], Citrus aurantium L. [Rutaceae; Aurantii Fructus Pericarpium], Lindera srtychnifolium [Lauraceae; Linderae Radix], Areca catechu L. [Arecaceae; Arecae Semen] | None | Húnán zhōng dá wù mǎ zhìyào gōngsī shēngchǎn | 14 days | None |
| Zhang 2013b | Jianweibao granule | Granule | Atractylodes lancea (Thunb.) DC. [Asteraceae; Atractylodis Rhizoma], Atractylodes macrocepha-la Koidz [Asteraceae; Atractylodis Rhizoma Alba], Crataegus pinnatifida Bge [Rosaceae; Crataegii Fructus], Gallus gallus var. domesticus Brisson [Phasianidae; Galli Stomachichum Corium], Citrus unshiu Markovich [Rutaceae; Citri Unshius Pericarpium] | None | Not applicable | 30 days | None |
| Zhang 2014a | Yì huáng sàn | Powder | Citrus unshiu Markovich [Rutaceae; Citri Unshius Pericarpium], Aucklandia lappa Decne [Asteraceae; Aucklandiae Radix], Citrus unshiu Markovich [Rutaceae; Citrii Unshiu Immaturi Pericarpium], Glycyrrhiza uralensis Fisch. [Leguminosae; Glycyrrhizae Radix], Crataegus pinnatifida Bge [Rosaceae; Crataegii Fructus], Triticum aestivum L. [Gramineae; Massa Medicata Fermentata], Hordeum vulgare L. [Gramineae; Hordei Fructus Germiniatus] | - With heat, Sloppy smelly stool, slimy fur: Agastache rugosa (Fisch. et Meyer) O. Kuntze [Labiatae; Agastachis Herba], Lonicera joponica Thunb. [Caprifoliaceae; Lonicerae Flos] - Profuse sweating: Oryza sativa var. glutinosa Matsum. [Gramineae; Oryzae Radix], Ephedra sinica Stapf. [Ephedraceae; Ephedrae Radix] - Yang deficiency with pale tongue: Dàn fù piàn - Peeling fur: Dendrobium loddigesii Rolfe. [Orchidaceae; Denbrobii Herba], Trichosanthes kirilowii Maxim. [Cucurbitaceae; Trichosanthis Fructus], Oryza sativa L. [Gramineae; Oryzae Fructus Germinatus] - Thirst, peeling fur: Adenophora triphylla var. japonica Hara [Campanulaceae; Adenophorae Radix] | Not applicable | 2-3 months | None |
| Zhang 2014b | Xingpi Yanger granule | Granule | Gerbera piloselloides (Linn. ) Cass., Pittosporum glabratum Lindl., Emilia sonchifolia (L.) DC.,Valeriana jatamansi Jones | None | Guìzhōu jiàn xìng yào yè yǒuxiàn gōngsī | 4 weeks | None |
| Zhang 2015 | Yunpi Kaiwei decoction | Decoction | Poria cocos (Schw.) Wolf [Polyporaceae; Poria(Hoelen)], Triticum aestivum L. [Gramineae; Massa Medicata Fermentata], Gallus gallus var. domesticus Brisson [Phasianidae; Galli Stomachichum Corium], Citrus unshiu Markovich [Rutaceae; Citri Unshius Pericarpium], Agastache rugosa (Fisch. et Meyer) O. Kuntze [Labiatae; Agastachis Herba] 6-10 g, Coix lachryma-jobi var. ma-yeun (Roman.) Stapf [Gramineae; Coicis Semen] 6-12 g, Dioscorea batatas Dacne. [Dioscoreaceae; Dioscoreae Rhizoma], Amomum villosum Lour. [Zingiberaceae; Amomi Fuctus], Raphanus sativus var. hortensis for. acanthiformis Makino [Brassicaceae; Raphani Semen], Citrus aurantium L. [Rutaceae; Aurantii Fructus Pericarpium], Magnolia officinalis Rehder et Wilson [Magnoliaceae; Magnoliae Cortex] 6-8 g, Crataegus pinnatifida Bge [Rosaceae; Crataegii Fructus] 8-10 g, Hordeum vulgare L. [Gramineae; Hordei Fructus Germiniatus] 8-12 g, Glycyrrhiza uralensis Fisch. [Leguminosae; Glycyrrhizae Radix] 3-6 g | - Palm and plantar fever, thirst, irritation: Forsythia suspensa (Thunb.) Vahl [Oleaceae; Forsythiae Fructus], Phyllostachys nigra var. henosis (Bean.) Stapf [Gramineae; Phyllostachydis Folium], Lú gēn - Constipation: Cannabis sativa L. [Cannabinaceae; Cannabis Fructus], Rheum palmatum L. [Polygonaceae; Rhei Rhizoma] - White slimy fur: Dolichos lablab L. [Leguminosae; Lablab Semen], Atractylodes lancea (Thunb.) DC. [Asteraceae; Atractylodis Rhizoma], Eupatorium chinese for. tripartitum H. Hara [Asteraceae; Eupatorii Herba] | Sìchuān xīn lǜsè yào yè kējì fāzhǎn gǔfèn yǒuxiàn gōngsī | 4 weeks | None |
| Zhang 2019 | Tiaogan Lipi granule | Granule | Atractylodes lancea (Thunb.) DC. [Asteraceae; Atractylodis Rhizoma], Atractylodes macrocepha-la Koidz [Asteraceae; Atractylodis Rhizoma Alba], Poria cocos (Schw.) Wolf [Polyporaceae; Poria(Hoelen)], Dolichos lablab L. [Leguminosae; Lablab Semen], Syzygium aromaticum Merr et Perry [Myrtaceac; Syzygii Flos], Aucklandia lappa Decne [Asteraceae; Aucklandiae Radix], Citrus unshiu Markovich [Rutaceae; Citri Unshius Pericarpium], Paeonia lactiflora Pall. [Paeoniaceae; Paeoniae Radix Alba], Bupleurum falcatum Linne [Apiaceae; Bupleuri Radix], Astagalus membranaceus Bunge [Leguminosae; Astragali Radix], Uncaria sinensis (Oliv.) Havil. [Rubiaceae; Uncariae Ramulus et Uncus], Forsythia suspensa (Thunb.) Vahl [Oleaceae; Forsythiae Fructus], Crataegus pinnatifida Bge [Rosaceae; Crataegii Fructus], Citrus aurantium L. [Rutaceae; Aurantii Fructus Pericarpium], Selaginella tamariscina (Beauv.) Spring [Selaginellaceae; Selaginelliae Herba] 6~10 g | None | Not applicable | 4 weeks | 4 week |
| Zhang 2020 | Jiàn pí hé wèi tāng | Decoction | Atractylodes macrocepha-la Koidz [Asteraceae; Atractylodis Rhizoma Alba], Poria cocos (Schw.) Wolf [Polyporaceae; Poria(Hoelen)] 10 g, Artemisia capillaris Thunb. [Asteraceae; Artemisiae Capillaris Herba], Gallus gallus var. domesticus Brisson [Phasianidae; Galli Stomachichum Corium], Codonopsis pilosulae (Fr.) Nannf. [Campanulaceae; Codonopsis Pilosulae Radix], Coix lachryma-jobi var. ma-yeun (Roman.) Stapf [Gramineae; Coicis Semen], Crataegus pinnatifida Bge [Rosaceae; Crataegii Fructus] 5 g, Chrysomyia megacephala Fabricius [Calliphoridae; Chrysomyiae] 3 g, Glycyrrhiza uralensis Fisch. [Leguminosae; Glycyrrhizae Radix], Picrorrhiza scrophulariiflora Pennell [Scrophulariaceae; Picrorrhozae Rhizoma] 2 g | - Severe dampness: remove Codonopsis pilosulae (Fr.) Nannf. [Campanulaceae; Codonopsis Pilosulae Radix] to 3 g, add Coix lachryma-jobi var. ma-yeun (Roman.) Stapf [Gramineae; Coicis Semen] to 10 g, Liriope platyphylla Wang et Tang [Liliaceae; Liriopes Radix] 3 g - Severe qi deficiency: remove Crataegus pinnatifida Bge [Rosaceae; Crataegii Fructus] to 3 g, add Dioscorea batatas Dacne. [Dioscoreaceae; Dioscoreae Rhizoma] 5 g | Not applicable | 4 weeks | None |
| Zhao 2004 | No name | Decoction | Panax ginseng C. A. Mey. [Araliaceae; Ginseng Radix], Atractylodes macrocepha-la Koidz [Asteraceae; Atractylodis Rhizoma Alba], Crataegus pinnatifida Bge [Rosaceae; Crataegii Fructus], Triticum aestivum L. [Gramineae; Massa Medicata Fermentata], Hordeum vulgare L. [Gramineae; Hordei Fructus Germiniatus] 5-10 g, Poria cocos (Schw.) Wolf [Polyporaceae; Poria(Hoelen)] 7-12 g, Amomum villosum Lour. [Zingiberaceae; Amomi Fuctus] 4-7 g, Bupleurum falcatum Linne [Apiaceae; Bupleuri Radix] 4-8 g, Paeonia lactiflora Pall. [Paeoniaceae; Paeoniae Radix Alba] 4-10 g, Astagalus membranaceus Bunge [Leguminosae; Astragali Radix] 5-8 g, Uncaria rhynchophylla (Miq.) Jacks. [Rubiaceae; Uncariae Ramulus cum Uncis] 7-10 g, Poncirus trifoliata Rafin. [Rutaceae; Aurantii Immaturus Fructus] 5-8 g, Liriope platyphylla Wang et Tang [Liliaceae; Liriopes Radix] 6-10 g, Areca catechu L. [Arecaceae; Arecae Semen] 4-7 g, Zingiber officinale Rosc. [Zingiberaceae; Zingiberis Rhizoma Siccus] 3-6 g | None | Not applicable | 10 days | 30 day |
| Zhao 2012 | Xiaoer Piweile granule | Granule | Dioscorea batatas Dacne. [Dioscoreaceae; Dioscoreae Rhizoma], Gallus gallus var. domesticus Brisson [Phasianidae; Galli Stomachichum Corium], Amomum krabanh Pierre ex Cagnep [Zingiberaceae; Amomi Rotundus Fructus], Rheum palmatum L. [Polygonaceae; Rhei Rhizoma], Hordeum vulgare L. [Gramineae; Hordei Fructus Germiniatus] | None | Not applicable | 4 weeks | None |
| Zhao 2015 | Yunpi Xiaoshi decoction | Decoction | Atractylodes lancea (Thunb.) DC. [Asteraceae; Atractylodis Rhizoma] 10 g, Agastache rugosa (Fisch. et Meyer) O. Kuntze [Labiatae; Agastachis Herba], Citrus aurantium L. [Rutaceae; Aurantii Fructus Pericarpium], Crataegus pinnatifida Bge [Rosaceae; Crataegii Fructus], Triticum aestivum L. [Gramineae; Massa Medicata Fermentata], Hordeum vulgare L. [Gramineae; Hordei Fructus Germiniatus] 9 g, Citrus unshiu Markovich [Rutaceae; Citri Unshius Pericarpium], Amomum villosum Lour. [Zingiberaceae; Amomi Fuctus], Amomum krabanh Pierre ex Cagnep [Zingiberaceae; Amomi Rotundus Fructus], Raphanus sativus var. hortensis for. acanthiformis Makino [Brassicaceae; Raphani Semen] 6 g, Glycyrrhiza uralensis Fisch. [Leguminosae; Glycyrrhizae Radix] 3 g | None | Not applicable | 30 days | None |
| Zhao 2020a | Xingpi Xiaoshi decoction | Decoction | Crataegus pinnatifida Bge [Rosaceae; Crataegii Fructus], Poncirus trifoliata Rafin. [Rutaceae; Aurantii Immaturus Fructus], Poria cocos (Schw.) Wolf [Polyporaceae; Poria(Hoelen)], Gallus gallus var. domesticus Brisson [Phasianidae; Galli Stomachichum Corium], Pseudostellaria augustifolia Y.N.Lee [Caryophyllaceae; Pseudostellariae Radix], Bù zhā yè, Tadehagi triquetrum (L.) Ohashi 10 g, Citrus unshiu Markovich [Rutaceae; Citri Unshius Pericarpium], Amomum villosum Lour. [Zingiberaceae; Amomi Fuctus] 5 g | None | Not applicable | 1 month | None |
| Zhao 2020b | Jianpi Xiaoshi decoction | Decoction | Atractylodes macrocepha-la Koidz [Asteraceae; Atractylodis Rhizoma Alba] 20 g, Poria cocos (Schw.) Wolf [Polyporaceae; Poria(Hoelen)] 12 g, Codonopsis pilosulae (Fr.) Nannf. [Campanulaceae; Codonopsis Pilosulae Radix], Amomum villosum Lour. [Zingiberaceae; Amomi Fuctus], Crataegus pinnatifida Bge [Rosaceae; Crataegii Fructus], Bupleurum falcatum Linne [Apiaceae; Bupleuri Radix] 10 g, Hordeum vulgare L. [Gramineae; Hordei Fructus Germiniatus], Dioscorea batatas Dacne. [Dioscoreaceae; Dioscoreae Rhizoma] 6 g, Triticum aestivum L. [Gramineae; Massa Medicata Fermentata], Magnolia officinalis Rehder et Wilson [Magnoliaceae; Magnoliae Cortex], Gallus gallus var. domesticus Brisson [Phasianidae; Galli Stomachichum Corium] 5 g, Raphanus sativus var. hortensis for. acanthiformis Makino [Brassicaceae; Raphani Semen], Glycyrrhiza uralensis Fisch. [Leguminosae; Glycyrrhizae Radix] 2 g | None | Not applicable | 1 month | None |
| Zheng 2012 | HM1: Qīwèi báizhú sàn HM2: Jiàn pí yì qì héjì, Xǐngpí kāiwèi héjì | HM1: Powder HM2: Decoction | - Qīwèi báizhú sàn: Poria cocos (Schw.) Wolf [Polyporaceae; Poria(Hoelen)], Citrus unshiu Markovich [Rutaceae; Citri Unshius Pericarpium], Nelumbo nucifera Gaertner [Nymphaceae; Nelumbinis Foluim], Perilla frutescens var. acuta Kudo [Labiatae; Perillae Semen], Oryza sativa L. [Gramineae; Oryzae Fructus Germinatus], Dolichos lablab L. [Leguminosae; Lablab Semen], Amomum villosum Lour. [Zingiberaceae; Amomi Fuctus], Selaginella tamariscina (Beauv.) Spring [Selaginellaceae; Selaginelliae Herba], Triticum aestivum L. [Gramineae; Massa Medicata Fermentata], Aucklandia lappa Decne [Asteraceae; Aucklandiae Radix], Coix lachryma-jobi var. ma-yeun (Roman.) Stapf [Gramineae; Coicis Semen], Nelumbo nucifera Gaertner [Nymphaceae; Nelumbinis Semen] - Jiàn pí yì qì héjì, Xǐngpí kāiwèi héjì: NR | None | NR | 1 month | None |
| Zhong 2020 | Shenling Baizhu powder | Decoction | Poria cocos (Schw.) Wolf [Polyporaceae; Poria(Hoelen)], Atractylodes lancea (Thunb.) DC. [Asteraceae; Atractylodis Rhizoma] 12 g, Codonopsis pilosulae (Fr.) Nannf. [Campanulaceae; Codonopsis Pilosulae Radix], Atractylodes macrocepha-la Koidz [Asteraceae; Atractylodis Rhizoma Alba] 10 g, Magnolia officinalis Rehder et Wilson [Magnoliaceae; Magnoliae Cortex] 9 g, Glycyrrhiza uralensis Fisch. [Leguminosae; Glycyrrhizae Radix] 6 g, Saposhnikovia divaricata Schiskin [Apiaceae; Saposhnikovia Radix] 3 g | None | Not applicable | 12 weeks | None |
| Zhou 2005 | Shugan Lipi decoction | Decoction | Bupleurum falcatum Linne [Apiaceae; Bupleuri Radix], Curcuma aromatica Salisb. [Zingiberaceae; Curcumae Radix], Agastache rugosa (Fisch. et Meyer) O. Kuntze [Labiatae; Agastachis Herba], Atractylodes macrocepha-la Koidz [Asteraceae; Atractylodis Rhizoma Alba], Codonopsis pilosulae (Fr.) Nannf. [Campanulaceae; Codonopsis Pilosulae Radix] 6-9 g, Dioscorea batatas Dacne. [Dioscoreaceae; Dioscoreae Rhizoma], Striga asiatica (L.) O. Kuntze, Gallus gallus var. domesticus Brisson [Phasianidae; Galli Stomachichum Corium], Crataegus pinnatifida Bge [Rosaceae; Crataegii Fructus], Prunus mume Sieb. et Zucc [Rosaceae; Mume Fructus], Triticum aestivum L. [Gramineae; Massa Medicata Fermentata] 9-12 g, Citrus aurantium L. [Rutaceae; Aurantii Fructus Pericarpium] 5-6 g, Glycyrrhiza uralensis Fisch. [Leguminosae; Glycyrrhizae Radix] 6 g | - Obvious qi deficiency: Astragalus membranaceus Bunge [Leguminosae; Astragali Radix] 9-12 g - Obvious yin deficiency: Dendrobium loddigesii Rolfe. [Orchidaceae; Denbrobii Herba] 6-9 g | Not applicable | 20 days | None |
| Zhou 2012 | Jianpi Xiaoshi decoction | Decoction | Crataegus pinnatifida Bge [Rosaceae; Crataegii Fructus], Triticum aestivum L. [Gramineae; Massa Medicata Fermentata], Hordeum vulgare L. [Gramineae; Hordei Fructus Germiniatus] 25 g, Atractylodes macrocepha-la Koidz [Asteraceae; Atractylodis Rhizoma Alba], Pseudostellaria augustifolia Y.N.Lee [Caryophyllaceae; Pseudostellariae Radix], Poria cocos (Schw.) Wolf [Polyporaceae; Poria(Hoelen)] 6 g, Citrus aurantium L. [Rutaceae; Aurantii Fructus Pericarpium], Citrus unshiu Markovich [Rutaceae; Citri Unshius Pericarpium], Selaginella tamariscina (Beauv.) Spring [Selaginellaceae; Selaginelliae Herba], Glycyrrhiza uralensis Fisch. [Leguminosae; Glycyrrhizae Radix] 3 g | None | Not applicable | 2 months | None |
| Zhou 2015 | Sijunzi decoction | Decoction | Crataegus pinnatifida Bge [Rosaceae; Crataegii Fructus], Triticum aestivum L. [Gramineae; Massa Medicata Fermentata], Hordeum vulgare L. [Gramineae; Hordei Fructus Germiniatus] 3-8 g, Codonopsis pilosulae (Fr.) Nannf. [Campanulaceae; Codonopsis Pilosulae Radix], Atractylodes macrocepha-la Koidz [Asteraceae; Atractylodis Rhizoma Alba], Poncirus trifoliata Rafin. [Rutaceae; Aurantii Immaturus Fructus], Gallus gallus var. domesticus Brisson [Phasianidae; Galli Stomachichum Corium] 3-6 g, Poria cocos (Schw.) Wolf [Polyporaceae; Poria(Hoelen)] 3-5 g, Paeonia lactiflora Pall. [Paeoniaceae; Paeoniae Radix Alba] 2-5 g, Citrus unshiu Markovich [Rutaceae; Citri Unshius Pericarpium]2-4 g, Glycyrrhiza uralensis Fisch. [Leguminosae; Glycyrrhizae Radix], Bupleurum falcatum Linne [Apiaceae; Bupleuri Radix] 1-3 g | - Diarrhea: Zingiber officinale Rosc. [Zingiberaceae; Zingiberis Rhizoma Siccus] 3-5 g, Amomum villosum Lour. [Zingiberaceae; Amomi Fuctus] 1-3 g - Constipation: Rheum palmatum L. [Polygonaceae; Rhei Rhizoma] 2-4 g - Effulgent liver fire with red tongue, thirst: Gardenia jasminoides var. grandiflora (Lour.) Nakai [Rubiaceae; Gardeniae Fructus], Scutellaria baicalensis Georgi [Labiatae; Scutellariae Radix] 3-5 g - Hiccups frequently: Raphanus sativus var. hortensis for. acanthiformis Makino [Brassicaceae; Raphani Semen] 3-6 g, Zingiber officinale Rosc. [Zingiberaceae; Zingiberis Rhizoma Recens] 3-5 g - Night cry, easy to startle: Cryptotympana pustulata Fabricius [Cicadidae; Cicadae Periostracum] 1-3 g - Abdominal pain with parasitic diseases: Quisqualis indica L. [Combretaceae; Quisqualis Fuctus] 1-3 g, Aloe vere L. [Liliaceae; Aloe] 1 g - Middle energizer dampness: Agastache rugosa (Fisch. et Meyer) O. Kuntze [Labiatae; Agastachis Herba] 1-3 g, Pinellia ternata (Thunb.) Breit. [Araceae; Pinelliae Rhizoma] 1-2 g | Not applicable | 4 weeks | None |
| Zhou 2018 | Zisheng decoction | Decoction | Dioscorea batatas Dacne. [Dioscoreaceae; Dioscoreae Rhizoma] 10-30 g, Atractylodes macrocepha-la Koidz [Asteraceae; Atractylodis Rhizoma Alba] 10-20 g, Dendrobium loddigesii Rolfe. [Orchidaceae; Denbrobii Herba], Dimocarpus longan Lour. [Sapindaceae; Longanae Arillus], Paeonia lactiflora Pall. [Paeoniaceae; Paeoniae Radix Alba] 10-15 g, Gallus gallus var. domesticus Brisson [Phasianidae; Galli Stomachichum Corium] 3-9 g, Polygonatum sibiricum Redoute [Liliaceae; Polygonati Rhizoma] 5-10 g, Citrus unshiu Markovich [Rutaceae; Citri Unshius Pericarpium]6-12 g | - Food stagnance: Crataegus pinnatifida Bge [Rosaceae; Crataegii Fructus], Raphanus sativus var. hortensis for. acanthiformis Makino [Brassicaceae; Raphani Semen], Hordeum vulgare L. [Gramineae; Hordei Fructus Germiniatus] - Abdominal pain: Corydalis ternata Nakai [Papaveraceae; Corydalis (Tuber) Rhizoma], Citrus aurantium L. [Rutaceae; Aurantii Fructus Pericarpium] | Jiāngsū shěng jiāngyīn shì tiān jiāng yào yè yǒuxiàn gōngsī | 4 weeks | None |
| Zhou 2020 | Shenling Baizhu powder | Powder | Panax ginseng C. A. Mey. [Araliaceae; Ginseng Radix], Poria cocos (Schw.) Wolf [Polyporaceae; Poria(Hoelen)], Atractylodes macrocepha-la Koidz [Asteraceae; Atractylodis Rhizoma Alba], Coix lachryma-jobi var. ma-yeun (Roman.) Stapf [Gramineae; Coicis Semen], Amomum villosum Lour. [Zingiberaceae; Amomi Fuctus], Dolichos lablab L. [Leguminosae; Lablab Semen], Platycodon grandiflorum (Jacq.) A. DC. [Campanulaceae; Platycodi Radix], Dioscorea batatas Dacne. [Dioscoreaceae; Dioscoreae Rhizoma], Nelumbo nucifera Gaertner [Nymphaceae; Nelumbinis Semen], Glycyrrhiza uralensis Fisch. [Leguminosae; Glycyrrhizae Radix] | None | Shānxī huá kāng yào yè gǔfèn yǒuxiàn gōngsī | 14 days | None |
| Zhu 2000 | Jianpi pill | Pill | Codonopsis pilosulae (Fr.) Nannf. [Campanulaceae; Codonopsis Pilosulae Radix], Triticum aestivum L. [Gramineae; Massa Medicata Fermentata], Hordeum vulgare L. [Gramineae; Hordei Fructus Germiniatus], Crataegus pinnatifida Bge [Rosaceae; Crataegii Fructus], Dioscorea batatas Dacne. [Dioscoreaceae; Dioscoreae Rhizoma] 10 g, Atractylodes macrocepha-la Koidz [Asteraceae; Atractylodis Rhizoma Alba], Poria cocos (Schw.) Wolf [Polyporaceae; Poria(Hoelen)] 6 g, Poncirus trifoliata Rafin. [Rutaceae; Aurantii Immaturus Fructus], Citrus unshiu Markovich [Rutaceae; Citri Unshius Pericarpium]4 g | - Spleen yang deficiency, middle energizer dampness, thick slimy fur : Atractylodes lancea (Thunb.) DC. [Asteraceae; Atractylodis Rhizoma], Agastache rugosa (Fisch. et Meyer) O. Kuntze [Labiatae; Agastachis Herba] - Spleen stomach qi deficiency: Astragalus membranaceus Bunge [Leguminosae; Astragali Radix], Nelumbo nucifera Gaertner [Nymphaceae; Nelumbinis Semen] - Spleen stomach yin deficiency: Prunus mume Sieb. et Zucc [Rosaceae; Mume Fructus], Polygonatum odoratum var. pluriflorum Ohwi [Liliaceae; Polygonati Dodrati Rhizoma] - Liver depression and spleen deficiency: Paeonia lactiflora Pall. [Paeoniaceae; Paeoniae Radix Alba], Elephas Species [Elephantidae; Fossilia Ossis Mastodi] | NR | 1 month | None |
| Zhu 2002 | Zengshi decoction | Decoction | Codonopsis pilosulae (Fr.) Nannf. [Campanulaceae; Codonopsis Pilosulae Radix] 12 g, Atractylodes lancea (Thunb.) DC. [Asteraceae; Atractylodis Rhizoma], Dioscorea batatas Dacne. [Dioscoreaceae; Dioscoreae Rhizoma], Poria cocos (Schw.) Wolf [Polyporaceae; Poria(Hoelen)] 9 g, Citrus unshiu Markovich [Rutaceae; Citri Unshius Pericarpium], Gallus gallus var. domesticus Brisson [Phasianidae; Galli Stomachichum Corium], Prunus mume Sieb. et Zucc [Rosaceae; Mume Fructus] 7 g, Glycyrrhiza uralensis Fisch. [Leguminosae; Glycyrrhizae Radix] 5 g | -The course of the disease is more than 3 years, severe anorexia: Amomum villosum Lour. [Zingiberaceae; Amomi Fuctus] 5 g - Visit in summer: Agastache rugosa (Fisch. et Meyer) O. Kuntze [Labiatae; Agastachis Herba] 6 g - Stagnation: Triticum aestivum L. [Gramineae; Massa Medicata Fermentata] 12 g, Oryza sativa L. [Gramineae; Oryzae Fructus Germinatus], Hordeum vulgare L. [Gramineae; Hordei Fructus Germiniatus] 9 g - Constipation: Liriope platyphylla Wang et Tang [Liliaceae; Liriopes Radix] 9 g, (severe) Rheum palmatum L. [Polygonaceae; Rhei Rhizoma] 6 g - Diarrhea: Atractylodes macrocepha-la Koidz [Asteraceae; Atractylodis Rhizoma Alba] 9 g, Plantago asiatica L. [Plantaginaceae; Plantaginis Semen] 6 g - Profuse sweating and easy to catch a cold: Ostrea gigas Thunb. [Ostreidae; Ostreae Concha] 15 g, Astragalus membranaceus Bunge [Leguminosae; Astragali Radix] 9 g, Saposhnikovia divaricata Schiskin [Apiaceae; Saposhnikovia Radix] 6 g - Night sweating: Ostrea gigas Thunb. [Ostreidae; Ostreae Concha] 15 g, Artemisiae apiacea Hance {Asteraceae; Artemisiae Apiaceae Herba] 6 g - Nausea and vomiting: Pinellia ternata (Thunb.) Breit. [Araceae; Pinelliae Rhizoma] 5 g | NR | 4 weeks | None |
| Zhu 2011 | bǎo hé wán | Pill | Triticum aestivum L. [Gramineae; Massa Medicata Fermentata], Crataegus pinnatifida Bge [Rosaceae; Crataegii Fructus], Forsythia suspensa (Thunb.) Vahl [Oleaceae; Forsythiae Fructus], Raphanus sativus var. hortensis for. acanthiformis Makino [Brassicaceae; Raphani Semen], Pseudostellaria augustifolia Y.N.Lee [Caryophyllaceae; Pseudostellariae Radix], Poria cocos (Schw.) Wolf [Polyporaceae; Poria(Hoelen)] 10 g | None | NR | 4-6 weeks | None |
| Zhu 2018 | Children's compound Jineijin chewable tablet | Tablet | Gallus gallus var. domesticus Brisson [Phasianidae; Galli Stomachichum Corium], Artemisiae apiacea Hance {Asteraceae; Artemisiae Apiaceae Herba], Polygonum hydropiper L., Xanthium strumarium L. [Asteraceae; Xanthii Herba], Phaseolus angularis W. f. Wight [Leguminosae; Phaseoli Semen], Prunus armeniaca L. var. ansu Maxim. [Rosaceae; Armeniacae Semen], Triticum aestivum L. [Gramineae; Tritici Testa] | None | Hénán tài lóng yào yè gǔfèn yǒuxiàn gōngsī shēngchǎn | 6 weeks | None |
| Zou 2014 | Jianwei Xiaoshi decoction | Decoction or Capsule | Pseudostellaria augustifolia Y.N.Lee [Caryophyllaceae; Pseudostellariae Radix] 12 g, Atractylodes lancea (Thunb.) DC. [Asteraceae; Atractylodis Rhizoma], Crataegus pinnatifida Bge [Rosaceae; Crataegii Fructus], Citrus unshiu Markovich [Rutaceae; Citri Unshius Pericarpium]10 g, Dolichos lablab L. [Leguminosae; Lablab Semen] 9 g, Atractylodes macrocepha-la Koidz [Asteraceae; Atractylodis Rhizoma Alba], Dioscorea batatas Dacne. [Dioscoreaceae; Dioscoreae Rhizoma], Gallus gallus var. domesticus Brisson [Phasianidae; Galli Stomachichum Corium], Amomum villosum Lour. [Zingiberaceae; Amomi Fuctus], Hordeum vulgare L. [Gramineae; Hordei Fructus Germiniatus] 8 g, Coix lachryma-jobi var. ma-yeun (Roman.) Stapf [Gramineae; Coicis Semen] 5 g | - Abdominal distension, yellow slimy fur: Raphanus sativus var. hortensis for. acanthiformis Makino [Brassicaceae; Raphani Semen], Agastache rugosa (Fisch. et Meyer) O. Kuntze [Labiatae; Agastachis Herba] - Sweat easily: Astragalus membranaceus Bunge [Leguminosae; Astragali Radix], Saposhnikovia divaricata Schiskin [Apiaceae; Saposhnikovia Radix] - Constipation: Cannabis sativa L. [Cannabinaceae; Cannabis Fructus] | NR | 2 weeks | None |
| Zou 2016 | Xiaoer Yanshi granule | Granule | Panax ginseng C. A. Mey. [Araliaceae; Ginseng Radix], Dioscorea batatas Dacne. [Dioscoreaceae; Dioscoreae Rhizoma], Atractylodes macrocepha-la Koidz [Asteraceae; Atractylodis Rhizoma Alba], Crataegus pinnatifida Bge [Rosaceae; Crataegii Fructus], Areca catechu L. [Arecaceae; Arecae Semen], Zingiber officinale Rosc. [Zingiberaceae; Zingiberis Rhizoma Siccus], Picrorrhiza scrophulariiflora Pennell [Scrophulariaceae; Picrorrhozae Rhizoma], Amomum villosum Lour. [Zingiberaceae; Amomi Fuctus] | None | Nèiménggǔ huì fēng yào yè yǒuxiàn gōngsī shēngchǎn | 4 weeks | 6 month |

Abbreviation. TG, treatment group
